# Entropy Transfer between Residue Pairs and Allostery in Proteins: Quantifying Allosteric Communication in Ubiquitin

**DOI:** 10.1371/journal.pcbi.1005319

**Published:** 2017-01-17

**Authors:** Aysima Hacisuleyman, Burak Erman

**Affiliations:** Department of Chemical and Biological Engineering, Koç University, Sariyer, Istanbul, Turkey; University of Houston, UNITED STATES

## Abstract

It has recently been proposed by Gunasakaran et al. that allostery may be an intrinsic property of all proteins. Here, we develop a computational method that can determine and quantify allosteric activity in any given protein. Based on Schreiber's transfer entropy formulation, our approach leads to an information transfer landscape for the protein that shows the presence of entropy sinks and sources and explains how pairs of residues communicate with each other using entropy transfer. The model can identify the residues that drive the fluctuations of others. We apply the model to Ubiquitin, whose allosteric activity has not been emphasized until recently, and show that there are indeed systematic pathways of entropy and information transfer between residues that correlate well with the activities of the protein. We use 600 nanosecond molecular dynamics trajectories for Ubiquitin and its complex with human polymerase iota and evaluate entropy transfer between all pairs of residues of Ubiquitin and quantify the binding susceptibility changes upon complex formation. We explain the complex formation propensities of Ubiquitin in terms of entropy transfer. Important residues taking part in allosteric communication in Ubiquitin predicted by our approach are in agreement with results of NMR relaxation dispersion experiments. Finally, we show that time delayed correlation of fluctuations of two interacting residues possesses an intrinsic causality that tells which residue controls the interaction and which one is controlled. Our work shows that time delayed correlations, entropy transfer and causality are the required new concepts for explaining allosteric communication in proteins.

## Author Summary

Allosteric communication is essential for the function of proteins. Recent work shows that allostery results from dynamic processes in the protein associated with atomic fluctuations leading to entropic interactions that involve ensemble of pathways rather than discrete two state transitions. Based on this new picture of allostery, it was proposed that allostery may indeed be an intrinsic property of all proteins. In order to test this hypothesis, we derive the computational tools for quantifying allosteric communication, and explain allostery in terms of entropy transfer, a new concept based on information theory. We use molecular dynamics simulations from which we calculate the transfer of entropy between pairs of residues. Results show that certain residues act as entropy sources while others as entropy sinks. Evaluation of time delayed correlations shows the presence of causality of interactions that allow us to differentiate between residues that are drivers in allosteric activity and those that are driven. Identification of driver-driven relations is important for drug design. Using the example of Ubiquitin, we identify paths of information transfer that control its binding to diverse partners in the Ubiquitin-Proteasome System. The proposed model can determine and quantify allosteric activity in any given protein.

## Introduction

Allosteric communication describes the process in which action at one site of a protein is transmitted to another site at which the protein performs its activity. The importance of allostery in biological systems has generated significant experimental and computational research. The basic problem is to identify residues that participate in allosteric communication in the hope of controlling their behavior related to protein function. Allosteric communication first requires the identification of two sites, the effector site, i.e., the site that is acted upon, and the regulatory site where protein's activity is regulated. Although more than 1000 allosteric sites are known [1] many more need to be characterized. In fact several pairs of allosteric endpoints may exist in a protein [2] which increases the number of candidate pairs that communicate. This problem becomes even more important when one considers the fact that most known cancers result from disruption of allosteric communication as a result of single mutations [3, 4] and the number of proteins associated with this phenomenon is very large. Expressed in simple terms, the solution of the problem reduces to finding whether two given residues communicate with each other, and if so what the consequences of this communication are. Various approaches to solve the problem may be found in References [5–16]. The specific aim of the present paper is to develop a rapid computational technique that identifies interaction of residue pairs based on concepts of information transfer and entropy, to scan a given protein and identify pairs of sites that communicate and to determine whether these communicating pairs may be candidates for allosteric activity.

The present work departs from the approaches outlined in the preceding paragraph. We do not focus either on single allosteric sites or on allosteric paths. We consider the time trajectory of the fluctuations of two residues, which may be spatially distant, and search for information transfer from the trajectory of one residue to that of the other. The trajectories are obtained from long molecular dynamics (MD) equilibrium simulations that give the fluctuation of each atom at constant temperature. The first requirement for information to be transferred from an atom *i* to another atom, *j*, is that their trajectories should be correlated. The second requirement is that this transfer should be asymmetric, i.e., information going from *i* to *j* should not be equal to information from *j* to *i*. This requires the use of time delayed correlations of fluctuations which may be asymmetric in contrast to time independent correlations which are symmetric by definition and therefore lack information on directionality. If *C*_*ij*_*(t*,*t+τ)* denotes the correlation of fluctuations of *i* at time *t* with those of *j* at time *t+τ*, then asymmetry requires that *C*_*ij*_*(t*,*t+τ)≠ C*_*ji*_*(t*,*t+τ)*. This introduces directionality and therefore causality into the problem. If time delayed correlations are asymmetric, then can we quantify the net information that is transferred? The answer is yes if we pose the problem in terms of entropy transfer.

Before going into the discussion of entropy, it is worth pointing out that information transfer is exclusively based on the changes in the amplitudes and frequencies of fluctuations in the system. This was first suggested and modelled by Cooper and Dryden (CD) [17] and reached larger dimensions by the work of Gunasekaran [18] which suggests that since allosteric communication is a result of correlated fluctuations then allostery should be an intrinsic dynamic property of all proteins. The dynamics aspect of proteins resides in the fluctuations of atoms which may be evaluated by experimentally measuring the B-factors of the atoms. The B-factor of the ith atom is related to its time independent autocorrelation of fluctuations, ⟨(*ΔR*_*i*_)^*2*^⟩, by $B_{i}=\frac{{8\pi }^{2}}{3}\langle {\left({\Delta R}_{i}\right)}^{2}\rangle$. However, knowledge of them is not sufficient for predicting allosteric communication and cross correlations ⟨Δ*R*_*i*_Δ*R*_*j*_⟩ between the fluctuations of different atoms are needed. Allosteric activity requires not only the modulation of the cross correlations in the system but also on time delayed cross correlations, ⟨*ΔR*_*i*_(*0*)*ΔR*_*j*_(*t*)⟩, as will be described below in detail. The CD model is referred to as 'Allostery without conformational change'. In this respect, it goes beyond the classical Monod-Wyman-Changeux (MWC) [19] model and its relative, the Koshland Nemethy Wyman (KNW) model [20] both of which relate allostery to discrete conformational changes at the regulatory site. Sending information by changing the amplitude and frequencies of fluctuations is entropic [21] and depends not only on the value of the entropy but also on the transfer of entropy from residue to residue during communication. Entropy as a source of information transfer is widely used in information theory [22] which is only very recently used for a protein-DNA complex by Kamberaj and van der Vaart [23]. Through analysis of entropy transfer, they determined residues that act as drivers of the fluctuations of other residues, thereby determining causality that is inherent in the correlations. Determining residues that act as drivers and those that are driven is important especially from the point of view of drug design. Entropy transfer and causality is a new paradigm for studying allosteric communication in proteins, which we elaborate in detail in the present paper. On a broader scale, our findings show that all proteins may indeed exhibit allosteric communication and therefore supports the hypothesis by Gunasakaran, [18] which states that allostery is an intrinsic property of all dynamic proteins.

The quantitative measure of information flow between two correlated processes is introduced by Schreiber [22] in 2000. In the present work, the processes are generated in the form of trajectories of atomic coordinates using MD simulations from which probabilities of atomic coordinate fluctuations required for evaluating transfer entropy are calculated. We calculate the entropies based on atoms and identify the entropy of a residue with the entropy of its alpha carbon. Denoting the probability of fluctuation of atom *i* by *p*_*i*_, Callen showed [24] that the Shannon measure of disorder, ${\text{-}k}_{B}\sum_{i=1}^{N}p_{i}{lnp}_{i}$ with *N* and *k*_*B*_ denoting the number of elements of the system and the Boltzmann constant, is the entropy of the system which is maximized at constant energy (See Callen [24], Chapter 17. Entropy and disorder: Generalized canonical formulations). At this point we give here a qualitative explanation of the relationship between information flow and a physical event such as fluctuations of atoms, and continue this discussion on a quantitative way after we introduce the statistical mechanical basis of the model. Suppose we have two trajectories, one of atom *i* and the other of atom *j*. If the fluctuations of *i* and *j* are independent of each other, then knowledge of the fluctuations *of i* will not give us information on the fluctuations of *j* and the uncertainty associated with the two events will be a maximum. The total entropy *of i* and *j* will be the sum of the singlet entropies, *S*_*i*_*+S*_*j*_. If, on the other hand, *i and j* move in a correlated way, the fluctuations of *i* controlling the fluctuations of *j*, then we will have more information on the fluctuations of *j* than if they were uncorrelated. For example, if *i* and *j* were perfectly correlated, then we would know exactly what *j* will do if we know *what i* is doing. This extra information *I*_*ij*_ that we gain because of the physical coupling of *i* and *j* is obtained by the Shannon equation and is termed as the mutual information and is always positive. The total entropy, *S*_*ij*_ of *i* and *j* in this case is written as *S*_*şj*_ *= S*_*i*_*+S*_*j*_*-I*_*ij*_ (see Eqs 12 and 13 and also Ref. [25]). Thus, correlation of fluctuations, irrespective of whether they are negative or positive, always decreases the sum of the singlet entropies of *i* and *j*. These arguments and the Shannon equation have been used to obtain entropy changes in proteins at different levels of approximation [21, 26–38]. However, we need to go beyond the Shannon equation in order to quantify allosteric communication in proteins which requires, as shown by Schreiber in 2000 [22], the knowledge of time delayed conditional probabilities of two trajectories. In the interest of determining which residue drives the correlated motions and which residue responds, van der Vaart applied the Schreiber equation to determine information flow between Ets-1 transcription factor and its binding partner DNA [23] (Also see references [39] and [40] in similar context). Since this first work on entropy transfer in proteins there has been a limited number of studies on information transfer in proteins. Barr et al. [41] quantified entropy transfer among several residues in a molecular dynamics analysis of mutation effects on autophosphorylation of ERK2. Corrada et al. [42] analyzed entropy transfer in antibody antigen interactions. Perilla et al. [43] used the transfer entropy method to analyze barrier crossing transitions in epidermal growth factor receptors. Qi and Im [44] quantified drive-response relations between residues during folding. Jo et al. [45] obtained a causality relationship between intramolecular hydrogen bonds and the conformational states of *N*-glycan core in glycoproteins. Zhang et al. [46] applied the method to understand changes in the correlated motions in the Rho GTPase binding domain during dimerization. An extensive overview of similar techniques is given in reference [47].

In the following section, we define the model on which we build the information theoretical basis of entropy. We then study the problem of time delayed correlation of fluctuations in proteins. Despite its importance in pointing to directionality of events in proteins, as has been shown recently for the allosteric activity of K-Ras [48], time delayed functions have not been studied in detail in the past. We then present a fast and accurate method of calculating entropy changes in proteins subject to pairwise interactions. Calculation of entropy of proteins is not new and has already been investigated by several authors [26–28, 49, 50] at different levels of approximation. Our method of entropy calculation is motivated by the recent finding that the distribution functions for the magnitude of fluctuations of residues in globular proteins can be derived from a universal function [51]. The method that we use for calculating the entropy is fast and accurate, based on histogramming the magnitude of fluctuations of each atom in a protein where the bin number is chosen according to the Sturges' rule of determining the widths of class intervals [52]. We show that the use of Sturges' rule in our computational method leads to results that agree with earlier entropy calculations. We benchmark our method with calculations of Ubiquitin by Fleck et al [38]. The entropy change of Ubiquitin upon binding to human polymerase iota that we calculate agrees with the value obtained in reference [38] using a different method of entropy estimation. The computational method that we adopt is efficient and plausible, and may directly be applied for evaluating entropy transfer in proteins.

The association of Shannon equation with statistical mechanical definition of entropy and quantifying transfer entropy using the Schreiber equation allows us to interpret a wide range of events in proteins. If entropy transfer is considered in terms of changes in mobility, then transfer of entropy from *i* to *j* implies decrease in the mobility of *i* due to its correlation with *j*. Stated in another way, residue *j* extracts entropy from *i*. If binding is considered, one could then say that transfer of entropy from *i* to *j* would facilitate binding at *i* due to lowered mobility of *i*, although this may not be a general trend and may depend on several other factors. We use the model to study the directionality of information flow and entropy transfer in the 76 amino acid protein Ubiquitin which is known to propagate signals allosterically in the cell by binding to a vast number of substrates [53]. Until the recent work of Smith et al., [54], the allosteric mechanisms of Ubiquitin were not widely recognized and it was not generally classified as an allosteric protein. Using NMR relaxation dispersion measurements, Smith et al., identified a collective global motion that originates from a conformational switch spatially distant from the site where Ubiquitin binds to other proteins. The predictions of the model we present agree with observations of Smith et al. The model goes one step further and predicts the direction of information transfer and therefore explain the underlying causes of the observed phenomenon. We discuss this in detail in the Discussion Section. In order to identify communication patterns leading to allosteric effects, we scanned the full Ubiquitin and identified the pairs of residues whose time delayed correlation functions are asymmetric and we quantified the amount of entropy transferred between residue pairs. In order to have a feeling of the effects of entropy transfer on protein-protein interactions, we analyzed the behavior of Ubiquitin when complexed with the binding partner human polymerase iota, 2KTF.pdb. We observed that binding of Ubiquitin to iota modifies the fluctuation patterns on another site that may affect the binding of a third protein which may possibly affect the formation of a ternary complex [55].

## Results

### Structure of Ubiquitin

**Fig 1:**
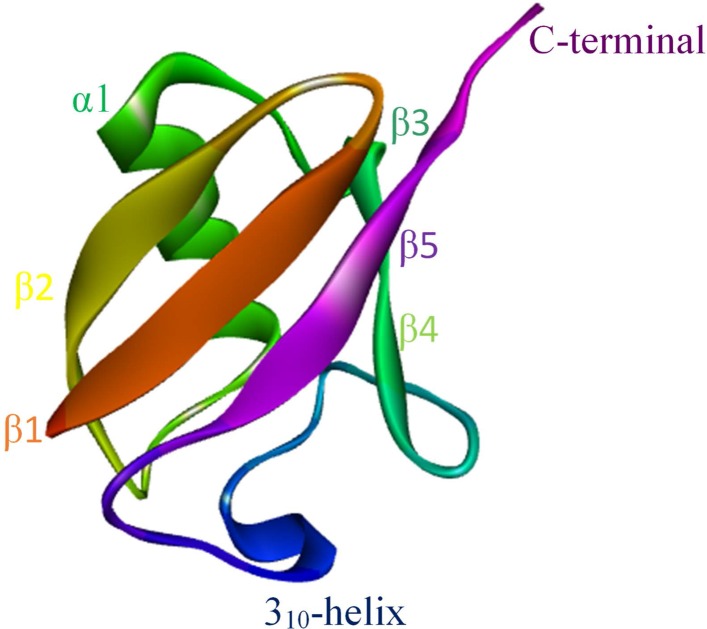
Structure of ubiquitin (1UBQ).

Ubiquitin is a 76 amino acid protein as shown in Fig 1. It consists of 8 distinct secondary structures that actively take part in its interactions with a large number of proteins.

**Fig 2:**
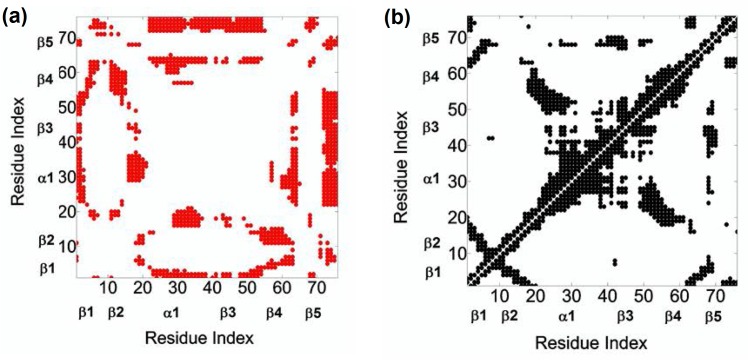
**a.** The left panel shows the negative Pearson correlations in the range (-1, -0.25), **b.** the right panel shows positive Pearson correlations in the range (0.25, 1.0). Pearson correlations are calculated from Eq 9.

The interactions of the secondary structures are strictly coordinated by the correlations in the protein. In Fig 2. we present the results of Pearson correlations of fluctuations, where the negative and positive correlations are shown in the left and right panels, respectively. The correlations with amplitudes (-1,-0.25) and (0.25, 1.0) are shown in the figure. The strongest negative correlation is between LEU73 and the three residues PRO37, PRO38 and ASP39. The next level of negative correlations are among distant pairs that are situated approximately diagonally opposite in the structure (examples are correlations between pairs LEU8-GLN31, ILE30-LYS63, GLU18-LYS33). The negative correlations between these pairs are expected to confer a breathing type of motion to the protein, which was indeed observed experimentally[54]. We elaborate on this point in detail in the Discussion Section. Fig 2B shows that positive correlations are mostly along the diagonal, indicating that neighboring residues along the primary sequence are positively correlated. However, there are off-diagonal regions in Fig 2B showing positive correlations among residues that are not close along the primary structure. The strongest off-diagonal positive correlation in Fig 2B is between GLU24 and GLY53.

### Transfer Entropy in Ubiquitin

We present the results of entropy transfer between all residue pairs of Ubiquitin. We consider only the alpha carbons, and the values given are divided by the Boltzmann constant. Results presented below are based on a trajectory of 600 ns. Entropy transfer values calculated from a duplicate trajectory of 600 ns gave similar results. Results of entropy transfer calculations for Ubiquitin and its complex presented below showed that convergence is established after 400 ns.

**Fig 3:**
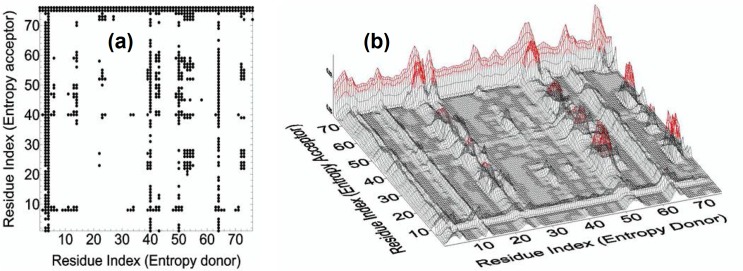
**a**. Entropy transfer from residue *i* to residue *j*. Abscissa represents residues which provide entropy to residues shown along the ordinate. Entropy transferred from residue i to residue *j* is obtained from Eq 16. Values between 0.0035–0.35 are recorded. Values below 0.0035 are not shown in order not to crowd the figure. *T*_*i*→*j*_(*τ*) values are calculated from Eq 16 with *τ = 5 ns*., **b.** Three dimensional description of entropy transfer in Ubiquitin. Red regions denote the residues with large contributions to transfer entropy. The figure is a 3-D version of Fig 3A.

Using Eq 16 we evaluated the values of entropy transfer from alpha carbon *i* to *j*, *T*_*i*→*j*_(*τ*), for all pairs of *i* and *j* for *τ = 5 ns*. Calculations averaged over several time stations between 0 and 5 ns gave approximately the same values for entropy transfer. In the remaining parts we present *τ = 5 ns* results only. The characteristic decay time of correlations of fluctuations of alpha carbons, which will be discussed in the following section, is on the average between 1 to 10 ns. The entropy transfer function T_i→j_(τ) that we obtain from fluctuation trajectories of alpha carbons depends on the correlation of two events that are *τ* ns apart in time. If *τ* is taken very small, i. e., around zero, then the difference between *T*_*i*→*j*_(*τ*) and *T*_*j*→*i*_(*τ*) will be very small because *T*_*i*→*j*_(0) = *T*_*j*→*i*_(0). If *τ* is taken much larger than the characteristic decay time, then the correlations will have decayed to small values and the differences will be vanishingly small. In agreement with this reasoning, we took *τ = 5 ns* and calculated entropy transfer at this time. The results are shown in Fig 3. The abscissa, named as entropy donor, denotes the indices of residues that act like entropy reservoirs to other residues. The ordinate, named as entropy acceptor, denotes the indices of residues that act like entropy sinks that absorb entropy from the system.

The columns of black points in the figure show that specific residues, such as ILE3 and PHE4, ILE13, ILE23, LYS27, GLY53, GLU64, ARG72 provide entropy to several residues of the protein. The rows of black circles indicate residues such as LEU8, THR9, GLY75 and 76, that absorb entropy from several residues of the protein. Residues ILE3 and PHE4, ILE13 and GLU64 form a spatial cluster. Also, the residues ILE23, LYS27 and GLY53 form a spatial cluster. If the allosteric path description is adopted, then we can say that these two spatial clusters lie on the allosteric path.

**Fig 4:**
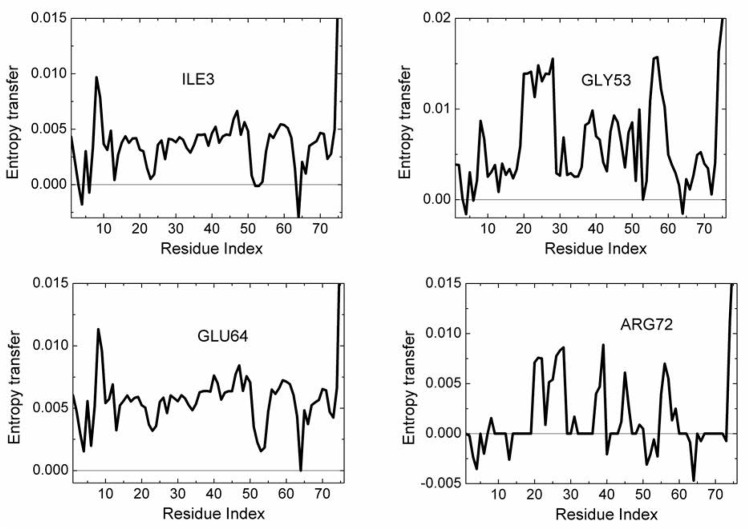
Entropy transfer from a given residue to other residues of the protein. The residue from which entropy is transferred is marked in each panel. Calculations are based on the relation *T*_*i*→*j*_(*5*)-*T*_*j*→*i*_(*5*).

**Fig 5:**
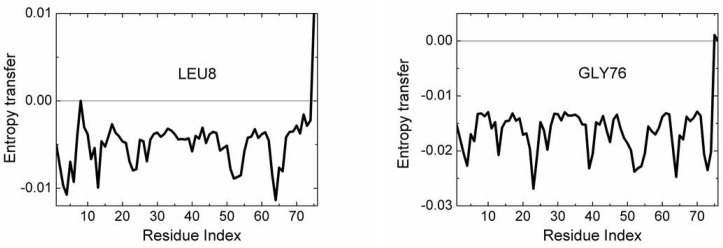
Entropy transfer from residues of the protein into LEU8 and GLY76. The residue from which entropy is transferred is marked in each panel. Calculations are based on the relation *T*_*i*→*j*_(*5*)-*T*_*j*→*i*_(*5*).

In order to have an idea on the mechanism of communication in the system, one needs to know the transfer of entropy among specific pairs of residues. From the data of Fig 3, we can find with which residues a given amino acid interacts entropywise. Figs 4 and 5 summarize the net entropy exchange, *T*_*i*→*j*_(*5*)-*T*_*j*→*i*_(*5*), between the labeled residue in each panel and the *j*^*th*^ residue of the protein. Fig 4 shows some examples with mostly positive entropy transfer from the labeled residue to others. The top left panel in Fig 4 shows entropy transfer from ILE3 to other residues. Specifically it transfers the largest entropy to LEU8 and GLY75 and GLY76. Both ILE3 and LEU8 are at the opposite extremities of β_1_. ILE3 is a spatial neighbor of GLU64. GLU64 is hydrogen bonded to GLN2, and the entropy of GLU64 is transferred to ILE3 via the stated hydrogen bond. ILE3 contributes entropy to several other residues of the protein as may be seen from the figure. Entropic interactions of residues PHE4, ILE13, ILE23 and LYS27 are very similar to those of ILE3 and are not given as a separate figure. The top right panel in Fig 4 shows the interactions of GLY53 with the rest of the protein. GLY53 is situated on the long loop between β_3_ and β_4_, and is hydrogen bonded to the main alpha carbon of GLU24 which is at the end of α_1_. Fig 4 shows that GLY53 contributes to the entropy of the segment between VAL17 and LYS29. It also transfers entropy along the chain to LEU56. GLU64 contributes entropy to several residues, in a way similar to that of ILE3. ARG72 has a unique pattern of contribution, specifically to ASP39 which is its spatial neighbor, to the loop between α_1_ and β_2_, to PHE45 and LEU56, both of which are spatially distant from ARG72. It also contributes to the mobility of the C-terminal. Fig 5 gives two examples for mostly negative values of *T*_*i*→*j*_(*5*)-*T*_*j*→*i*_(*5*). The left panel in Fig 5 shows that LEU8 and GLY76 absorb entropy from most of the residues of the protein.

**Fig 6:**
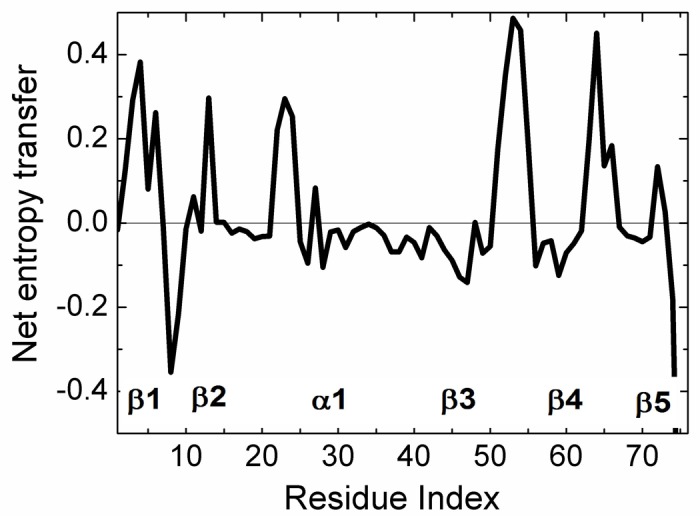
Net entropy transfer from one residue to the rest of the protein, calculated by Eq 17. A residue with a positive (negative) value of net entropy transfer is an entropy source (sink).

The net transfer of entropy from residue i, defined by Eq 17 is presented in Fig 6. Positive values denote net entropy transfer out from a residue, and negative values denote net entropy into a residue. Similar to the pattern observed in Fig 3, we see that certain residues behave as entropy sources for the rest of the protein and some behave as entropy sinks.

**Fig 7:**
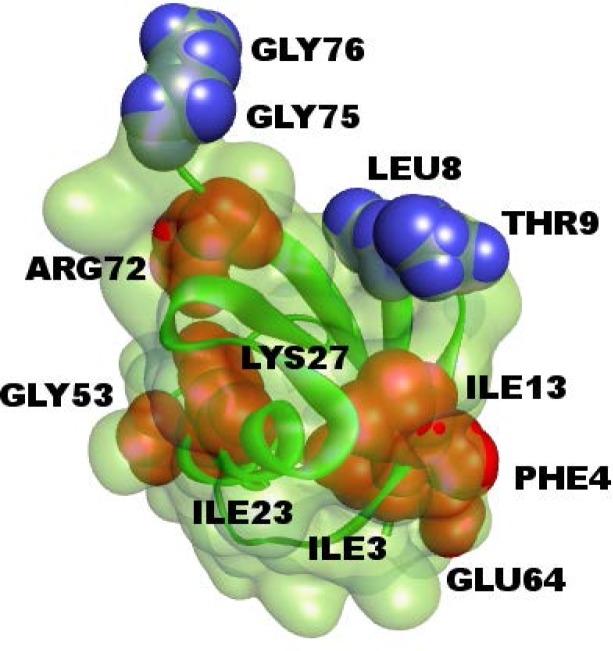
Structure of ubiquitin, residues that are colored in red are entropy acceptors and residues that are colored in blue are the entropy donors.

We see that β_1_ and β_2_ act as an entropy sources as well as part of the helix α_1_. The largest amount of entropy is provided by the loops between β_3_β_4_ and β_4_β_5_. The two major entropy sinks are the loop between β_1_ and β_2_ and the last two residues of the C-terminal. Entropy sources are located mostly at secondary structures or at their extremities. The three residues PHE4, THR14, GLU64 are spatial neighbors. Similarly, LEU43, LEU50, are spatial neighbors. The entropy source and sink residues are shown in three dimensions in Fig 7.

### Time Delayed Correlations of Ubiquitin

**Fig 8:**
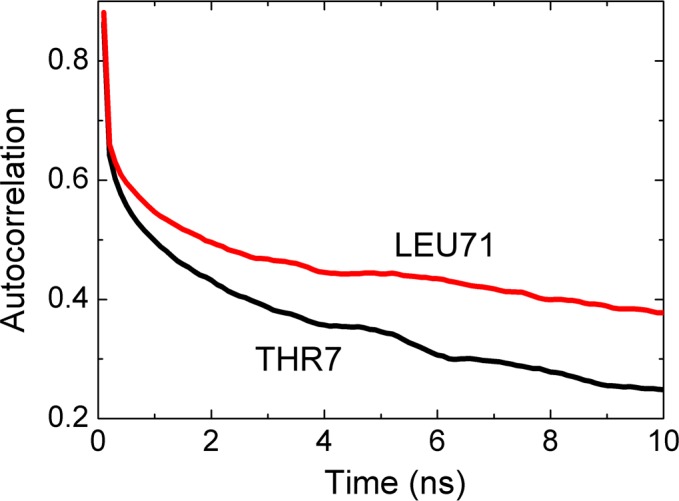
Autocorrelations functions for THR7 and LEU71 calculated from Eq 7 for *i* = *j*. The abscissa denotes the time delay parameter, i.e., the time between two observations, one at time zero the other at the indicated time on the axis.

Fluctuations of amino acids in Ubiquitin display characteristic decay times that are in the order of 1 to 10 ns as may be observed from the decay of the curves to 1/e of their original values. Differences arise from the unique conformational features of the amino acid and its environment. In Fig 8, we show the autocorrelations of THR7 and LEU71.

The autocorrelation function for THR7, i.e., the time required to decay to 1/e of the original value is 5 ns. LEU71 decays slightly slower with a decay time of 10 ns.

The time delayed cross correlations of the fluctuations of two amino acids are of interest because they yield information on the causality of events. The static correlations presented in Fig 2 are symmetric, i.e., *C*_*ij*_(*τ*) = *C*_*ji*_(*τ*). However, time delayed cross correlations of fluctuations of two amino acids show asymmetries which we discuss in this section.

**Fig 9:**
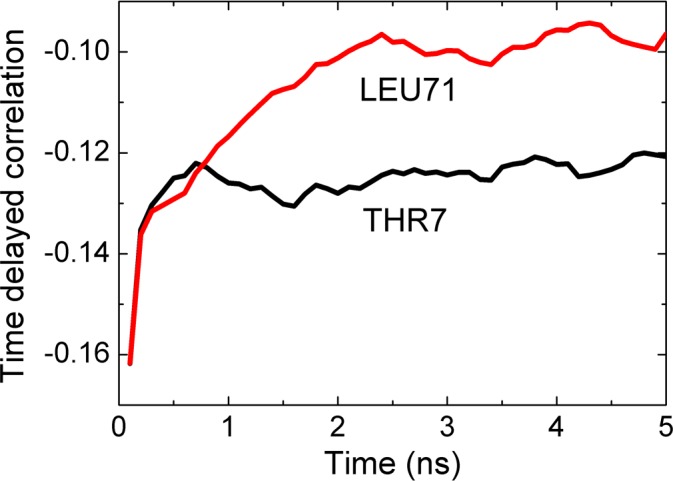
Cross correlation of fluctuations of THR7 and LEU71. Black line is for correlations where THR7 precedes LEU71. The red line is for correlations where LEU71 precedes THR7. The abscissa denotes the time delay parameter, i.e., the time between two observations, one at time zero the other at the indicated time on the axis. The curves are calculated from Eq 7.

**Fig 10:**
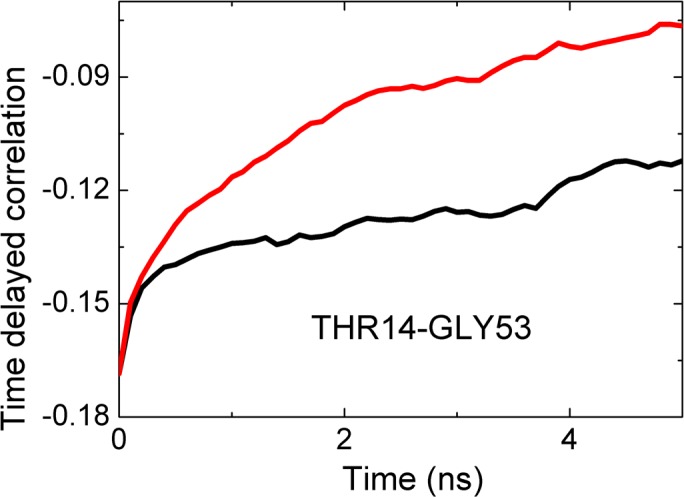
Cross correlation of fluctuations of THR14 and GLY53. Black line is for correlations where THR14 precedes GLY53. The red line is for correlations whereGLY53 precedes THR14. The abscissa denotes the time delay parameter, i.e., the time between two observations, one at time zero the other at the indicated time on the axis. The curves are calculated from Eq 7.

In Figs 9 and 10, we present two cases that show significant causality. The strongest asymmetry is between LEU7 and THR71, shown in Fig 9.

In this figure, the black curve is for correlation of THR7 at time *t* and LEU71 at *t+τ*. The red curve is for LEU71 at *t* and THR7 at *t+τ*. The black curve decays significantly slower than the red curve, indicating that the effect of the fluctuations of THR7 on later fluctuations of LEU71 persists for longer times whereas the converse is not true. We therefore say that the motions of THR7 drive the motions of, LEU71 i.e., THR7 is the driver and is LEU71 driven. Since LEU71 is located on the C-terminal segment, and THR7 is at the end of β_1_, we can say that the β_1_ strand controls the fluctuations of the C-terminal. We see that the black curve remains approximately constant after a rapid initial decay. This shows that the driver action of THR7 on LEU71 persists for longer times.

In Fig 10, the black curve is for the correlation of the fluctuations of THR14 with later fluctuations of GLY53. The red curve is for the reverse case, fluctuations of GLY53 affecting later fluctuations of THR14. This figure shows that THR14 is the driver and GLY53 is driven. THR14 is on the β_2_ strand and GLY53 is on the long loop connecting the α3_10_ helix to β_4_.

## Discussion

The entropy transfer model that we developed for understanding allosteric communication in proteins measures the amount of information transfer between the trajectories of two atoms, *i* and *j*. Knowing the fluctuations of atoms *i* and *j* at time *t*, the model evaluates the amount of uncertainty reduced in the future fluctuations of atom *j*. One extreme case is where the fluctuations of *i* have no effect on the fluctuations of *j*, i.e., their trajectories are uncorrelated. In this case, Eq 16 equates to zero, and no entropy will be transferred to residue *j* from *i*. The other extreme case is where the fluctuations of *i* ar time *t* are perfectly locked into those of *j* at time *t* and the knowledge of the present values of *i* and *j* fluctuations will reduce the uncertainty of the future fluctuations of *j*. In this case, the second term *S*(*ΔR*_*j*_(*t*+*τ*)|*ΔR*_*i*_(*t*),*ΔR*_*j*_(*t*)) in Eq 15 will be modified as *S*(*ΔR*_*j*_(*t*+*τ*)|*ΔR*_*j*_(*t*))-*S*(*ΔR*_*i*_(*t*)), which states that the reduction in uncertainty is due to the locking in of the fluctuations of *i*. Substituting these into Eq 16 leads to *T*_*i*→*j*_(*τ*) = *S*(*ΔR*_*i*_). The range of values of entropy that may be transferred from *i* to *j* will lie within the interval *0* ≤ *T*_*i*→*j*_(*τ*) ≤ *S*(*ΔR*_*i*_). In the absence of symmetry, *T*_*i*→*j*_(*t*) ≠ *T*_*j*→*i*_(*t*), we talk of a net transfer of entropy from *i* to *j* which will lie in the interval -*S*(*ΔR*_*j*_) ≤ *T*_*i*→*j*_(*τ*)-*T*_*j*→*i*_(*τ*) ≤ *S*(*ΔR*_*i*_). If (*T*_*i*→*j*_(*τ*)-*T*_*j*→*i*_(*τ*)) is greater than zero, we say that the fluctuations of atom *i* drive those of atom *j*.

Based on these explanations, we now compare the predictions of the model with experimental data. Progress in NMR spectroscopy and Relaxation Dispersion measurements allows for reliable experimental determination of correlations of fluctuations of residues that may be spatially distant[54, 56, 57]. Such long range correlations are candidate mechanisms that require information transfer, and hence may be seen as suitable indicators of entropy transfer. We show that measured correlations and patterns of entropy transfer that we calculate are complementary to each other.

### Comparison with Experiment

Strong correlations between two residues, ILE23 and ASN25 have been observed by NMR studies of Ubiquitin [58, 59]. A more detaild investigation by Ban et al., [57] using the recently developed NMR Relaxation Dispersion technique showed a strong correlation between ILE23, ASN25 and THR55. Later work by Smith et al., [54] using Relaxation Dispersion measurements showed that the two residues GLU24 and GLY53 act as a conformational switch and their correlated fluctuations induce breathing-like motions in the overall protein which affect the substrate binding region of Ubiquitin. Our Pearson correlation analysis shows that GLU24 and GLY53 are strongly positively correlated and there is significant entropy transfer from GLY53, which is located on a loop, to GLU24, located on the helix, as may be seen from Fig 3B and the top right panel of Fig 4. According to these figures, GLY53 is a strong entropy source for GLU24. Thus, we see a strong directionality in the interactions of the two residues that form a conformational switch that controls the overall motions of the protein: GLY53 drives the fluctuations of GLU24. In turn, GLU24 drives residues LEU8, GLN40 and the C-terminal residues.

Secondly, based on the predictive features of our model, we discuss the possible consequences of ligand binding and mutation, both of which are of significant relevance to drug design.

### Changes upon Complex Formation of Ubiquitin

**Fig 11:**
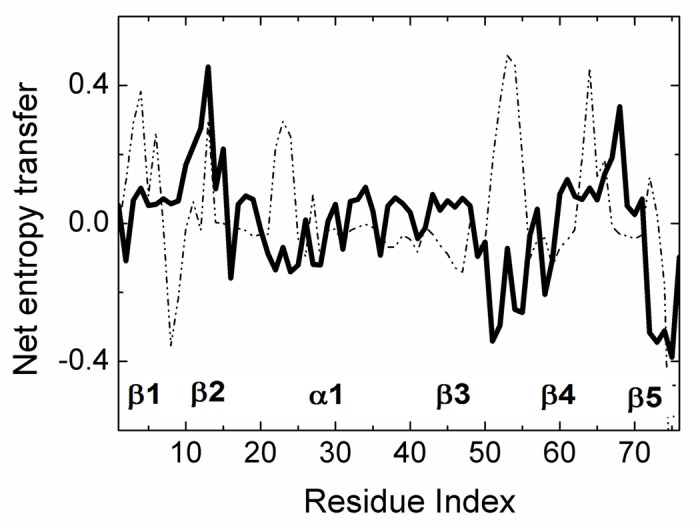
Net entropy transfer of residues in unbound Ubiquitin (dashed curve) and in the complex (solid curve) Curves are obtained by using Eq 17.

Ubiquitin forms complexes with a multitude of proteins. Here we studied its complex with Human Polymerase Iota which is a small protein of 28 amino acids, 2L0G.pdb, it binds to the following residues of Ubiquitin: LEU8, THR9, GLY10, GLY47, ARG42, ILE44 and the C-terminal. Net entropy transfer in Ubiquitin in the bound and free states is compared in Fig 11. The solid and dashed curves are for the bound and free states, respectively. Entropy transfer characteristics of four residues of Ubiquitin show significant changes upon complex formation. LEU8 which was and entropy sink in the unbound Ubiquitin is no longer a sink. GLU24, GLY53 and GLU64 which were strong entropy sources in the unbound state cease to be so in the complex. A possible interpretation of this observation is that GLY53 no longer drives GLU24 which in turn does not excite the breathing like motions of Ubiquitin, and the entropy transfer characteristics of Ubiquitin is now completely changes in favor of conformations that prefer complexation.

Mutations, although we have not performed simulation on such systems, are expected to significantly modify the entropy transfer characteristics of those residues that exhibit strong entropy transfer in the wild type. For example, mutation of GLU24 or GLY53 would abolish the driver-driven relations and change the functional dynamics of Ubiquitin significantly. In fact Smith et al. [54]performed mutations experimentally and observed that the affinity toward ubiquitins binding partner weakened twofold in both mutants. Mutation of residues LEU8 and GLN40 should also lead to strong changes in allosteric behavior of Ubiquitin because these resudues also have important role on entropy transfer, as was discussed in the preceding paragraphs.

Entropy transfer is computed only for alpha carbons in the present work. In principle, the calculations may be extended to include sidechains also since molecular dynamics trajectories are performed for all atoms of the system and the information for sidechain entropies is present in the trajectories. In the interest of brevity and clarity of presentation, only alpha carbons were treated at this preliminary analysis of entropy transfer in proteins. It is worth stating, however, that NMR measurements on backbone carbon and nitrogen were sufficient to characterize the allosteric dynamics of Ubiquitin [54, 58]. Sidechains and amino acid types will undoubtedly affect residue-residue communication, especially when considering differences between the bound and unbound states, and should be included in a more detailed analysis. Binding of iota to Ubiquitin modifies the fluctuation patterns of residues ILE3, ILE23, GLY53 and GLU64 which are on a surface that is susceptible to the binding of a third protein, a case which may possibly affect the formation of a ternary complex [55].

In conclusion, we used the Schreiber's model of entropy transfer and presented a detailed analysis of allosteric communication in Ubiquitin. Based on the analysis of time delayed events, we showed that information may be transferred between pairs of residues. The allosteric mechanisms of Ubiquitin have been understood only very recently. Our work shows that there is significant information transfer between residue pairs in this system. From the entropy transfer point of view, all proteins may exhibit allosteric communication. This observation supports the recent hypothesis by Gunasekaran et al [18] that allostery is indeed an intrinsic property of proteins. Our work shows that the knowledge of time delayed correlations and entropy transfer is needed in order to quantify allosteric communication in proteins. Time delayed events have not been widely used in studies of protein function and allosteric communication. Recently, it was shown that causality introduced by time delayed correlations plays significant role on allosteric communications in K-Ras. In this respect, time delayed correlation functions may be viewed as a new tool for studying allosteric communication in proteins. A three dimensional map of entropy transfer, as shown in Fig 3B may be useful for visualizing allosteric communication between pairs of residues more easily. Based on Fig 3B and the entropy transfer propensities of residue, the model serves as a suitable tool for explaining the basis of allosteric mechanisms in proteins.

Finally, it is worth noting that the present approach which maps the causality, driver-driven relations, and entropy exchange into pairs of residues, as seen in Fig 3B, should be of great significance for allosteric drug design because it tells us which residues to manipulate. In this respect, a driver residue is more critical than the driven residue and manipulating the driver will be perturb the existing correlations more efficiently. The effects of mutation on allosteric communication may be quantified by calculating the changes in entropy transfer. As we showed in the UBQ-Human Polymerase Iota complex, binding may result in entropy changes in the exposed residues of the complex and change the binding propensities of the complex to other molecules such as another protein, a small molecule ligand or a DNA segment.

## Methods

### Molecular Dynamics Trajectories

We perform molecular dynamics simulations for a protein in equilibrium and extract stationary trajectories for each atom. The trajectories for the atoms are expressed as

$$R\left(t_{k}\right)=R\left(R_{1}\left(t_{k}\right),R_{2}\left(t_{k}\right),R_{3}\left(t_{k}\right),..,R_{N}\left(t_{k}\right)\right)\;k=1,2,..,n_{T}$$ (1)

Here, *R*_*i*_*(t*_*k*_*)* is the position vector of the ith atom at the *k*^*th*^ time *t*_*k*_, expressed in terms of its Cartesian coordinates, *X*_*i*_*(t*_*k*_*)*, *Y*_*i*_*(t*_*k*_*)* and *Z*_*i*_*(t*_*k*_*)*, *N* is the total number of atoms and *t*_*k*_ is the time in the kth step.*k* ranges from 1 to *n*_*T*_, the total number of steps in the simulation. If the total time is*T*, then the length *ξ* of each time step is *ξ = T/n*_*T*_. Each atom has a unique equilibrium mean position defined by

$$\bar{R}=R\left({\bar{R}}_{1},{\bar{R}}_{2},{\bar{R}}_{3},..,{\bar{R}}_{N}\right)$$ (2)

We denote the instantaneous state of fluctuation of a protein at time *t*_*k*_ by the vector

$$\Delta R\left(t_{k}\right)=R\left(t_{k}\right)-\bar{R}$$ (3)

which reads in vector form as

$$\Delta R\left(t_{k}\right)=\Delta R\left(\Delta R_{1}\left(t_{k}\right),\Delta R_{2}\left(t_{k}\right),\Delta R_{3}\left(t_{k}\right),\ldots ,\Delta R_{N}\left(t_{k}\right)\right)$$ (4)

For each *t*_*k*_, Eq 4 has *N* entries. For the purposes of the present study, we need the magnitude of the fluctuations only. In the following, we will let *ΔR*_*i*_*(t*_*k*_*)* represent the magnitude of the fluctuation at time *t*_*k*_.

### Evaluation of Probabilities

The most general expression for the probability of fluctuation *ΔR* is the joint probability *p(ΔR) p(ΔR*_*1*_, *ΔR*_*2*,_ *ΔR*_*3*_,.. *ΔR*_*N*_*)*. This expression contains information on all orders of dependence between atoms and is too general for use. In the other extreme, the simplest expression is the singlet probability function *p*_*i*_*(ΔR*_*i*_*)* which is obtained from the most general expression by

$$p\left(\Delta R_{i}\right)=\int_{0}^{\infty }\cdots \int_{0}^{\infty }\cdots \int_{0}^{\infty }\cdots \int_{0}^{\infty }p\left(\Delta R_{1},\Delta R_{2},\Delta R_{3},..,\Delta R_{N}\right)d\Delta R_{1},\ldots ..,\Delta R_{i-1},..,\Delta R_{i+1},..,\Delta R_{N}$$ (5)

*N* such functions define the probability of fluctuations of the *N* residues within the singlet approximation.

The next simplest probability is the pair probability *p(ΔR*_*i*_, *ΔR*_*j*_*)* obtained from the most general expression by

$$\begin{array}{l} p\left(\Delta R_{i},\Delta R_{j}\right)=\int_{0}^{\infty }\cdots \int_{0}^{\infty }p\left(\Delta R_{1},\Delta R_{2},\Delta R_{3},..,\Delta R_{N}\right)\\ \;d\Delta R_{1},..,\Delta R_{i-1},..,\Delta R_{i+1},..,\Delta R_{j-1},..,\Delta R_{j+1},..,\Delta R_{N} \end{array}$$ (6)

For *N* atoms, there are $\frac{N\left(N\text{-}1\right)}{2}$ equations for pair probabilities.

In Eqs 5 and 6, *ΔR*’s are treated as continuous. In the remaining of the paper, we will adopt a discrete representation for them in terms of histograms. The histograms will be expressed in terms of *n* bins. We refer to each bin as a state. The variables in the probabilities will then be functions of state variables. Thus we write *p(ΔR*_*i*_*(k))* where *k* goes from 1 to *n* where *n* is the number of states that define *ΔR*_*i*_. Similarly, *p(ΔR*_*i*_*(k)*, *ΔR*_*j*_*(l))*. In order to simplify the notation, we will suppress the state index, and write *p(ΔR*_*i*_*(k))* as *p(ΔR*_*i*_*)*. Similarly, *p(ΔR*_*i*_*(k)*, *ΔR*_*j*_*(l))≡ p(ΔR*_*i*_, *ΔR*_*j*_*)*.

### Time Delayed Correlation Functions

We let *p(ΔR*_*i*_*(t)*, *ΔR*_*j*_*(t+τ))* denote the joint probability of observing the fluctuation *ΔR*_*i*_ at time *t* and *ΔR*_*j*_ at time *t+τ*. In this simplified notation, *ΔR*_*i*_*(t)* represents the value of *ΔR*_*i*_ in state *k* at time *t*, which is identical to *ΔR*_*i*_*(k*,*t)*. *ΔR*_*j*_*(t+ τ)* may be affected by the earlier fluctuations of *ΔR*_*i*_*(t)*. The extent of this effect may be quantified by the time delayed correlation function

$$C_{ij}\left(\tau \right)=\frac{\left[\sum_{k=1}^{n_{T}-\tau /\xi }\Delta R_{i}\left(t_{k}\right)\Delta R_{j}\left(t_{k}+\tau \right)/\left(n_{T}-\tau /\xi \right)\right]}{{\langle {\left(\Delta R_{i}\right)}^{2}\rangle }^{1/2}{\langle {\left(\Delta R_{j}\right)}^{2}\rangle }^{1/2}}$$ (7)

This is a conditional correlation where *ΔR*_*i*_ comes before *ΔR*_*j*_. In general, *p(ΔR*_*i*_*(t)*, *ΔR*_*j*_*(t+τ))≠ p(ΔR*_*j*_*(t)*, *ΔR*_*i*_*(t+τ))*. This leads to directionality in the structure, known as causality, and consequently,

$$C_{ij}\left(\tau \right)\neq C_{ji}\left(\tau \right)$$ (8)

If the fluctuations of residue *i* control the fluctuations of residue *j*, i.e., if residue *j* is driven by *i*, then the decay time for *C*_*ij*_*(τ)* will be larger than that of *C*_*ji*_*(τ)*.

When *τ = 0*, time independent Pearson correlation function is obtained as

$$C_{ij}\left(0\right)=\frac{\langle \Delta R_{i}\Delta R_{j}\rangle }{{\langle {\left(\Delta R_{i}\right)}^{2}\rangle }^{1/2}{\langle {\left(\Delta R_{j}\right)}^{2}\rangle }^{1/2}}$$ (9)

### Entropy

The entropy for a pair of subsytems, *i* and *j*, the entropy *S*_*2*,*ij*_ is given as

$$S_{2,ij}=-k_{B}\sum_{k}\sum_{l}p_{ij}\left(k,l\right)\ln\;p_{ij}\;\left(k,l\right)=-k_{B}\langle \ln\;p_{ij}\rangle$$ (10)

In Eq 10, the indices *k* and *l* denote the indices for the states of the respective subsystems, the states being obtained from the histograms described in the preceding section. For the case of pairwise interactions, the expression *p*_*ij*_*(k*,*l)* represents the joint probability where subsystem *i* is in state *k* and the subsystem *j* is in state *l*. We used the notation *p*_*ij*_*(k*,*l) = p(ΔR*_*i*_*(k)*, *ΔR*_*j*_*(l))* for brevity of presentation.

In Eq 10, *S*_*2*,*ij*_ signifies the joint entropy for two subsystems with pair probabilities.

We now divide and multiply the entropy expression by the singlet probabilities:

$$S_{2,ij}=-k_{B}\left\langle \ln\left(\frac{p_{ij}}{p_{i}p_{j}}p_{i}p_{j}\right)\right\rangle$$ (11)

which leads to the expression

$$S_{2,ij}=-k_{B}\left\langle \ln\;p_{i}\right\rangle -k_{B}\left\langle \ln\;p_{j}\right\rangle -k_{B}\left\langle \ln\left(\frac{p_{ij}}{p_{i}p_{j}}\right)\right\rangle$$ (12)

$$S_{2,ij}=S_{1,i}+S_{1,j}-I_{2,ij}$$ (13)

where, *S*_*1*,*i*_ = -*k*_*B*_⟨*lnp*_*i*_⟩ is the singlet entropy and $I_{2,ij}{=k}_{B}\left\langle ln\left(\frac{p_{ij}}{p_{i}p_{j}}\right)\right\rangle$ is the mutual information of the system.

**Fig 12:**
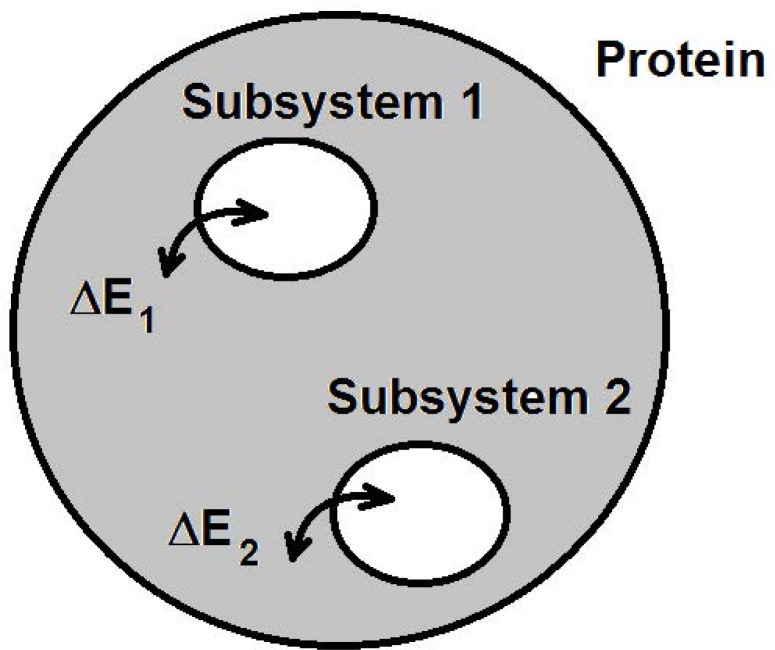
Energy exchange between protein and its subsystems.

Using statistical mechanics arguments given by Callen [24], each subsystem may be treated as a canonical ensemble that exchanges energy with its surroundings, represented by the cartoon in Fig 12. The surroundings of Subsystem 1 for example is the protein which contains Subsystem 2 also. We may choose the subsystems arbitrarily, an atom, an amino acid, or a secondary structure such as a helix, beta strand, loop or a tail. The subsystem may also be in contact with the surroundings of the protein. Mutual information is zero if the fluctuations of *i* are independent of the fluctuations of *j*. Otherwise, mutual information is always greater than zero. This leads to the conclusion that correlations always decrease the sum of the individual entropies in a system.

### Conditional Entropy

We consider two trajectories, *ΔR*_*i*_*(t)* and *ΔR*_*j*_*(t)*. We now consider two events separated in time by *τ*, with the condition that *ΔR*_*i*_ coming before *ΔR*_*j*_. The conditional entropy for these two events is defined by

$$\begin{array}{l} S\left(\Delta R_{j}\left(t+\tau \right)|\Delta R_{i}\left(t\right)\right)=-\sum_{}p\left(\Delta R_{i}\left(t\right),\Delta R_{j}\left(t+\tau \right)\right)\ln p\left(\Delta R_{j}\left(t+\tau \right)|\Delta R_{i}\left(t\right)\right)\\ \;=\left\langle \ln p\left(\Delta R_{j}\left(t+\tau \right)|\Delta R_{i}\left(t\right)\right)\right\rangle \\ \;=\left\langle \ln\frac{p\left(\Delta R_{i}\left(t\right),\Delta R_{j}\left(t+\tau \right)\right)}{p\left(\Delta R_{i}\left(t\right)\right)}\right\rangle \\ \;=\left\langle \ln p\left(\Delta R_{i}\left(0\right),\Delta R_{j}\left(\tau \right)\right)\right\rangle -\left\langle \ln p\left(\Delta R_{i}\left(0\right)\right)\right\rangle \end{array}$$ (14)

where, the summation is over all states for *i* and *j*, and the condition of stationarity is used in the last equation.

### Transfer Entropy

Following Schreiber's work [22], we write the transfer entropy *T*_*i*→*j*_(*τ*) from trajectory *i* to *j* at time *τ* as

$$T_{i\to j}\left(\tau \right)=S\left(\Delta R_{i}\left(t+\tau \right)|\Delta R_{i}\left(t\right)\right)-S\left(\Delta R_{j}\left(t+\tau \right)|\Delta R_{i}\left(t\right),\Delta R_{j}\left(t\right)\right)$$ (15)

Using the last of Eq 14, this may be written as

$$\begin{array}{l} T_{i\to j}\left(\tau \right)=-\left\langle \ln\;p\left(\Delta R_{j}\left(0\right),\Delta R_{j}\left(\tau \right)\right)\right\rangle +\left\langle \ln\;p\left(\Delta R_{i}\left(0\right),\Delta R_{j}\left(0\right),\Delta R_{j}\left(\tau \right)\right)\right\rangle \\ \;+\left\langle \ln\;p\left(\Delta R_{j}\left(0\right)\right)\right\rangle -\left\langle \ln\;p\left(\Delta R_{i}\left(0\right),\Delta R_{j}\left(0\right)\right)\right\rangle \end{array}$$ (16)

Through the term *p(ΔR*_*i*_*(0)*, *ΔR*_*j*_*(0)*, *ΔR*_*j*_*(τ))*,, Eq 16 requires the evaluation of triple probabilities. If trajectories *i* and *j* are independent, then *T*_*i*→*j*_(*τ*) = *0* entropy transfer from *i* to *j* will be zero. In general, *T*_*i*→*j*_(*τ*) ≠ *T*_*j*→*i*_(*τ*) and this will determine the net transfer of entropy from one event to another separated in time by *τ*. Different values of *τ* shows how entropy transfer depends on prior interactions. In this study, we will take *τ = 5 ns* as the representative correlation time of cross correlations.

### Net Entropy Transfer from an Atom

Eq 16 gives the entropy transferred, *T*_*i*→*j*_(*τ*), from atom *i* to *j*. The net entropy transferred from to all other atoms is obtained by summing over all *j* as

$$Net\;transfer\;from\;residue\;i=\sum_{j=1}^{N}\left(T_{i\to j}\left(\tau \right)-T_{j\to i}\left(\tau \right)\right)$$ (17)

### Molecular Dynamics Simulation

All-atom Molecular Dynamics simulations were performed for unbound (PDB ID: 1UBQ) and bound states (PDB ID: 2KTF) of Ubiquitin, using NAMD 2.11 simulation program with CHARMM22 All-Hydrogen Parameter set for Proteins and Lipids. TIP3P water model was used to represent water molecules. Counter ions are placed to neutralize the system. Time step of simulations were 2 fs and periodic boundary conditions were applied in an isothermal-isobaric NPT ensemble with constant temperature of 300 K and constant pressure of 1 bar. Temperature and pressure are controlled by Langevin thermostat and Langevin piston barostat, respectively. System coordinates were saved every 1 ps. 1–4 scaling is applied to van der Waals interactions with a cutoff of 12.0 Å. Energy of the system was minimized and system is heated to 300 K for 50 ps and further subjected to MD production run for 600 ns. Frames in trajectories were aligned to the first frame of the simulation by using VMD 1.9.2 to eliminate all rotational and translational degrees of freedom and the analysis is done with the aligned Cartesian coordinates.

### Entropy Calculations

Amplitude of fluctuations were calculated for each atom from Cartesian coordinates, *R*_*i*_*(t*_*k*_*)*, of the trajectory and the mean amplitude of fluctuations, $\bar{R_{i}}$ was subtracted from each *R*_*i*_*(t*_*k*_*)* and the *ΔR*_*i*..*N*_*(t*_*k*_*)* matrix was generated with *(t*_*k*_,*N)* dimensions, where *N* is the number of atoms and *t*_*k*_ is the number of frames selected for calculations. All of the calculations in this study will be based on the alpha carbon of each residue unless otherwise stated. Initial data up to equilibration was excluded from the calculations as equilibration. A binning approach was used to calculate configurational entropy, for individual and pairwise dependent atoms. Calculations were performed using MATLAB R2015b. Histogram function of MATLAB was used to cluster data into 8 bins with specified widths and partitioning of data is adaptive according to the maximum and minimum of data. Calculations were performed using 8 discrete bins. Number of bins were selected according to the Sturges’ rule. The optimum number, *n*_*opt*_, of bins is calculated from the Sturges' rule according to

$$n_{opt}=mean\;fluctuation\cdot \left(1+\log_{2}N\right)$$ (18)

Here, the mean of fluctuations, i.e., the average fluctuation of the *N* alpha carbons divided by maximum fluctuation is calculated from the trajectories and is equal to 0.4. For a trajectory of 600,000 time steps, the optimum number of bins is obtained as 8 which is used throughout the calculations. After partitioning the fluctuation of each atom into 8 discrete bins, the probabilities were calculated from the frequency of occurrences and entropy was expressed in individual and pairwise mutual information terms. For comparison with benchmark calculations, the change of configurational entropy was calculated for all heavy atoms by subtracting pairwise mutual information term from individual entropy term as given by Eq 12. By using Eq 16, transfer entropy from atom *i* to *j* was calculated with a delay value of 5 ns for alpha carbons by subtracting the triple conditional entropies from pairwise conditional entropies. Result of configurational entropy calculations were compared with benchmark data and transfer entropy results were used to study changes in entropy transfer patterns when Ubiquitin forms a complex.

Amount of mutual entropy depends on the distribution of the individual entropies and it is bounded by individual entropy terms.

$$I_{2}\left(i,j\right)\leq \min\left\{S\left(i\right),S\left(j\right)\right\}$$ (19)

Estimated entropy from a finite sample may be affected by some systematic errors and a correction term is required to get rid of this error [60]. Corrections were applied according to the previous studies [61]

$$S_{1}^{true}\approx S_{1}^{estimated}+\frac{M-1}{2N}$$ (20)

Where $S_{1}^{estimated}$ is the raw entropy, *M* is the number of histogram bins with non-zero probability. Since mutual entropy is the sum of entropies, this formula can also be used to correct *I*_*2*_*(i*,*j)* terms.

$$I_{2}^{true}\approx I_{2}^{estimated}+\frac{M_{ij}-M_{i}-M_{j}+1}{2N}$$ (21)

Where *M*_*ij*_, *M*_*i*_ and *M*_*j*_ represent the numbers of the corresponding histogram bins with non-zero probabilities.

### Benchmark for Entropy Calculations

Our method of configurational entropy calculations which we need for calculating transfer entropy are based on a histograming method using Sturges’ rule. We compare the results of our configurational entropy calculations with those of MIST(Mutual Information Spanning Trees) method of PARENT [38]. The mean entropy change result of Ubiquitin upon complex formation with human polymerase iota, 2KTF.pdb was obtained by our method as -47.64 (standard deviation of 12.33) calculated from 5 different portions from a simulation of 1200 ns. For the same system, MIST gave a mean of -40.59 (standard deviation of 28.99) for 5 different MD simulation sets for Ubiquitin and its complex. The error between the means of our result and of MIST is 17.4%, which is within 1 standard deviation of PARENT results.

## Supporting Information

S1 Text Data for figures. (DOCX)

## References

[1] Huang Z, Zhu L, Cao Y, Wu G, Liu X, Chen Y, et al ASD: a comprehensive database of allosteric proteins and modulators. Nucleic acids research. 2011;39(suppl 1):D663–D9. 21051350 10.1093/nar/gkq1022 PMC3013650

[2] Barbany M, Meyer T, Faustino I, D'Abramo M, Morata J, Orozco M, et al Molecular dynamics study of naturally existing cavity couplings in proteins. PloS one. 2015;10(3):e0119978 10.1371/journal.pone.0119978 25816327 PMC4376744

[3] Cerami E, Gao J, Dogrusoz U, Gross BE, Sumer SO, Aksoy BA, et al The cBio cancer genomics portal: an open platform for exploring multidimensional cancer genomics data. Cancer discovery. 2012;2(5):401–4. PubMed Central PMCID: PMC3956037. 10.1158/2159-8290.CD-12-0095 22588877 PMC3956037

[4] Henry VJ, Bandrowski AE, Pepin AS, Gonzalez BJ, Desfeux A. OMICtools: an informative directory for multi-omic data analysis. Database: the journal of biological databases and curation. 2014;2014. PubMed Central PMCID: PMC4095679. 10.1093/database/bau069 PMC4095679 25024350

[5] Dror RO, Green HF, Valant C, Borhani DW, Valcourt JR, Pan AC, et al Structural basis for modulation of a G-protein-coupled receptor by allosteric drugs. Nature. 2013;503(7475):295–9. 10.1038/nature12595 24121438

[6] Novinec M, Korenč M, Caflisch A, Ranganathan R, Lenarčič B, Baici A. A novel allosteric mechanism in the cysteine peptidase cathepsin K discovered by computational methods. Nature communications. 2014;5. 10.1038/ncomms4287 24518821

[7] Lu S, Huang W, Zhang J. Recent computational advances in the identification of allosteric sites in proteins. Drug discovery today. 2014;19(10):1595–600. 10.1016/j.drudis.2014.07.012 25107670

[8] Hardy JA, Wells JA. Searching for new allosteric sites in enzymes. Current opinion in structural biology. 2004;14(6):706–15. 10.1016/j.sbi.2004.10.009 15582395

[9] Panjkovich A, Daura X. PARS: a web server for the prediction of protein allosteric and regulatory sites. Bioinformatics. 2014;30(9):1314–5. 10.1093/bioinformatics/btu002 24413526

[10] Collier G, Ortiz V. Emerging computational approaches for the study of protein allostery. Archives of biochemistry and biophysics. 2013;538(1):6–15. 10.1016/j.abb.2013.07.025 23933229

[11] Erman B. A fast approximate method of identifying paths of allosteric communication in proteins. Proteins: Structure, Function, and Bioinformatics. 2013;81(7):1097–101. 10.1002/prot.24284 23508936

[12] Shukla D, Meng Y, Roux B, Pande VS. Activation pathway of Src kinase reveals intermediate states as targets for drug design. Nature communications. 2014;5. 10.1038/ncomms4397 PMC4465921 24584478

[13] Tang S, Liao J-C, Dunn AR, Altman RB, Spudich JA, Schmidt JP. Predicting allosteric communication in myosin via a pathway of conserved residues. Journal of molecular biology. 2007;373(5):1361–73. 10.1016/j.jmb.2007.08.059 17900617 PMC2128046

[14] Kaya C, Armutlulu A, Ekesan S, Haliloglu T. MCPath: Monte Carlo path generation approach to predict likely allosteric pathways and functional residues. Nucleic acids research. 2013;41(W1):W249–W55. 23742907 10.1093/nar/gkt284 PMC3692092

[15] McClendon CL, Friedland G, Mobley DL, Amirkhani H, Jacobson MP. Quantifying correlations between allosteric sites in thermodynamic ensembles. J Chem Theory Comput. 2009;5(9):2486–502. 10.1021/ct9001812 20161451 PMC2790287

[16] Tsai C-J, Nussinov R. A unified view of “how allostery works”. PLoS Comput Biol. 2014;10(2):e1003394 10.1371/journal.pcbi.1003394 24516370 PMC3916236

[17] Cooper A, Dryden D. Allostery without conformational change. European Biophysics Journal. 1984;11(2):103–9. 6544679 10.1007/BF00276625

[18] Gunasekaran K, Ma B, Nussinov R. Is allostery an intrinsic property of all dynamic proteins? Proteins: Structure, Function, and Bioinformatics. 2004;57(3):433–43. 10.1002/prot.20232 15382234

[19] Monod J, Wyman J, Changeux J-P. On the nature of allosteric transitions: a plausible model. Journal of molecular biology. 1965;12(1):88–118. 14343300 10.1016/s0022-2836(65)80285-6

[20] Koshland D Jr, Nemethy G, Filmer D. Comparison of experimental binding data and theoretical models in proteins containing subunits*. Biochemistry. 1966;5(1):365–85. 5938952 10.1021/bi00865a047

[21] Motlagh HN, Wrabl JO, Li J, Hilser VJ. The ensemble nature of allostery. Nature. 2014;508(7496):331–9. 10.1038/nature13001 24740064 PMC4224315

[22] Schreiber T. Measuring information transfer. Physical Review Letters. 2000;85(2):461–4. 10.1103/PhysRevLett.85.461 10991308

[23] Kamberaj H, van der Vaart A. Extracting the Causality of Correlated Motions from Molecular Dynamics Simulations. Biophysical Journal. 2009;97(6):1747–55. 10.1016/j.bpj.2009.07.019 19751680 PMC2749796

[24] Callen HB. Thermodynamics and an introduction to thermostatistics. Second ed: Wiley; 1985.

[25] MacKay DJ. Information theory, inference and learning algorithms: Cambridge university press; 2003.

[26] Karplus, Kushick. Method for Estimating the Configurational Entropy of Macromolecules.

[27] Karplus M, Ichiye T, Pettitt BM. Configurational Entropy of Native Proteins. Biophysical Journal. 1987;52(6):1083–5. 10.1016/S0006-3495(87)83303-9 3427197 PMC1330109

[28] Suarez E, Diaz N, Mendez J, Suarez D. CENCALC: A Computational Tool for Conformational Entropy Calculations from Molecular Simulations. Journal of Computational Chemistry. 2013;34(23):2041–54. 24046838 10.1002/jcc.23350

[29] Suarez E, Diaz N, Suarez D. Entropy Calculations of Single Molecules by Combining the Rigid-Rotor and Harmonic-Oscillator Approximations with Conformational Entropy Estimations from Molecular Dynamics Simulations. J Chem Theory Comput. 2011;7(8):2638–53. 10.1021/ct200216n 26606637

[30] King BM, Silver NW, Tidor B. Efficient calculation of molecular configurational entropies using an information theoretic approximation. The Journal of Physical Chemistry B. 2012;116(9):2891–904. 10.1021/jp2068123 22229789 PMC3465721

[31] Killian BJ, Yundenfreund Kravitz J, Gilson MK. Extraction of configurational entropy from molecular simulations via an expansion approximation. J Chem Phys. 2007;127(2):024107 PubMed Central PMCID: PMCPMC2707031. 10.1063/1.2746329 17640119 PMC2707031

[32] Killian BJ, Kravitz JY, Somani S, Dasgupta P, Pang YP, Gilson MK. Configurational Entropy in Protein-Peptide Binding: Computational Study of Tsg101 Ubiquitin E2 Variant Domain with an HIV-Derived PTAP Nonapeptide. Journal of Molecular Biology. 2009;389(2):315–35. 10.1016/j.jmb.2009.04.003 19362095 PMC2758778

[33] Numata J, Knapp EW. Balanced and Bias-Corrected Computation of Conformational Entropy Differences for Molecular Trajectories. J Chem Theory Comput. 2012;8(4):1235–45. 10.1021/ct200910z 26596740

[34] Hnizdo V, Tan J, Killian BJ, Gilson MK. Efficient calculation of configurational entropy from molecular simulations by combining the mutual-information expansion and nearest-neighbor methods. J Comput Chem. 2008;29(10):1605–14. PubMed Central PMCID: PMCPMC2620139. 10.1002/jcc.20919 18293293 PMC2620139

[35] Zídek L, Novotny MV, Stone MJ. Increased protein backbone conformational entropy upon hydrophobic ligand binding. Nature Structural & Molecular Biology. 1999;6(12):1118–21. 10.1038/70057 10581552

[36] Suarez E, Suarez D. Multibody local approximation: Application to conformational entropy calculations on biomolecules. Journal of Chemical Physics. 2012;137(8). Artn 084115. 10.1063/1.4748104 22938226

[37] Ben-Naim A. A farewell to entropy: statistical thermodynamics based on information: S = logW. Hackensack, N.J.: World Scientific; 2008 xxv, 384 p. p.

[38] Fleck M, Polyansky AA, Zagrovic B. PARENT: A Parallel Software Suite for the Calculation of Configurational Entropy in Biomolecular Systems. J Chem Theory Comput. 2016;12(4):2055–65. 10.1021/acs.jctc.5b01217 26989950

[39] van der Vaart A. Coupled binding–bending–folding: The complex conformational dynamics of protein-DNA binding studied by atomistic molecular dynamics simulations. Biochimica et Biophysica Acta (BBA)-General Subjects. 2015;1850(5):1091–8. 25161164 10.1016/j.bbagen.2014.08.009

[40] Karolak A, van der Vaart A. Importance of local interactions for the stability of inhibitory helix 1 in apo Ets-1. Biophysical chemistry. 2012;165:74–8. 10.1016/j.bpc.2012.03.007 22494801

[41] Barr D, Oashi T, Burkhard K, Lucius S, Samadani R, Zhang J, et al Importance of domain closure for the autoactivation of ERK2. Biochemistry. 2011;50(37):8038–48. 10.1021/bi200503a 21842857 PMC3172398

[42] Corrada D, Morra G, Colombo G. Investigating Allostery in Molecular Recognition: Insights from a Computational Study of Multiple Antibody–Antigen Complexes. The Journal of Physical Chemistry B. 2013;117(2):535–52. 10.1021/jp310753z 23240736

[43] Perilla JR, Leahy DJ, Woolf TB. Molecular dynamics simulations of transitions for ECD epidermal growth factor receptors show key differences between human and drosophila forms of the receptors. Proteins: Structure, Function, and Bioinformatics. 2013;81(7):1113–26. 10.1002/prot.24257 PMC3968921 23348956

[44] Qi YF, Im W. Quantification of Drive-Response Relationships Between Residues During Protein Folding. J Chem Theory Comput. 2013;9(8):3799–805. 10.1021/ct4002784 PMC3819712 24223527

[45] Jo S, Qi Y, Im W. Preferred conformations of N-glycan core pentasaccharide in solution and in glycoproteins. Glycobiology. 2015:cwv083. 10.1093/glycob/cwv083 PMC4672150 26405106

[46] Zhang L, Centa T, Buck M. Structure and Dynamics Analysis on Plexin-B1 Rho GTPase Binding Domain as a Monomer and Dimer. The Journal of Physical Chemistry B. 2014;118(26):7302–11. 10.1021/jp503668k 24901636 PMC4096216

[47] Karolak A. Application and Development of Computational Methods in Conformational Studies of Bio-molecules. 2015.

[48] Vatansever S, Gümüş ZH, Erman B. Intrinsic K-Ras dynamics: A novel molecular dynamics data analysis method shows causality between residue pair motions. Scientific Reports. 2016;6. 10.1038/srep37012 PMC5109477 27845397

[49] Kassem S, Ahmed M, El-Sheikh S, Barakat KH. Entropy in bimolecular simulations: A comprehensive review of atomic fluctuations-based methods. J Mol Graph Model. 2015;62:105–17. 10.1016/j.jmgm.2015.09.010 26407139

[50] Numata J, Wan M, Knapp EW. Conformational entropy of biomolecules: beyond the quasi-harmonic approximation. Genome Inform. 2007;18:192–205. 18546487

[51] Erman B. Universal features of fluctuations in globular proteins. Proteins: Structure, Function, and Bioinformatics. 2016;84(6):721–5. 10.1002/prot.25032 26971570

[52] Legg PA, Rosin PL, Marshall D, Morgan JE. Improving accuracy and efficiency of registration by mutual information using Sturges’ histogram rule. Proc Med Image Understand Anal. 2007:26–30.

[53] Liu J, Nussinov R. The role of allostery in the ubiquitin–proteasome system. Critical reviews in biochemistry and molecular biology. 2013;48(2):89–97. 10.3109/10409238.2012.742856 23234564 PMC3609921

[54] Smith CA, Ban D, Pratihar S, Giller K, Paulat M, Becker S, et al Allosteric switch regulates protein–protein binding through collective motion. Proceedings of the National Academy of Sciences. 2016:201519609. 10.1073/pnas.1519609113 PMC4812760 26961002

[55] Garner TP, Strachan J, Shedden EC, Long JE, Cavey JR, Shaw B, et al Independent interactions of ubiquitin-binding domains in a ubiquitin-mediated ternary complex. Biochemistry. 2011;50(42):9076–87. 10.1021/bi201137e 21923101

[56] Fenwick RB, Esteban-Martín S, Richter B, Lee D, Walter KF, Milovanovic D, et al Weak long-range correlated motions in a surface patch of ubiquitin involved in molecular recognition. Journal of the American Chemical Society. 2011;133(27):10336–9. 10.1021/ja200461n 21634390 PMC3686050

[57] Ban D, Funk M, Gulich R, Egger D, Sabo TM, Walter KF, et al Kinetics of conformational sampling in ubiquitin. Angewandte Chemie International Edition. 2011;50(48):11437–40. 10.1002/anie.201105086 22113802

[58] Massi F, Grey MJ, Palmer AG. Microsecond timescale backbone conformational dynamics in ubiquitin studied with NMR R1ρ relaxation experiments. Protein science. 2005;14(3):735–42. 10.1110/ps.041139505 15722448 PMC2279275

[59] Dittmer J, Bodenhausen G. Evidence for slow motion in proteins by multiple refocusing of heteronuclear nitrogen/proton multiple quantum coherences in NMR. Journal of the American Chemical Society. 2004;126(5):1314–5. 10.1021/ja0386243 14759169

[60] Scarabelli G, Morra G, Colombo G. Predicting interaction sites from the energetics of isolated proteins: a new approach to epitope mapping. Biophysical journal. 2010;98(9):1966–75. 10.1016/j.bpj.2010.01.014 20441761 PMC2862194

[61] Paninski L. Estimation of entropy and mutual information. Neural computation. 2003;15(6):1191–253.

