# Supplementary material for: Entropy Transfer between Residue Pairs and Allostery in Proteins: Quantifying Allosteric Communication in Ubiquitin
Source: PLoS Comput Biol. 2017 Jan 17;13(1):e1005319. doi: 10.1371/journal.pcbi.1005319 (PMC5283753; doi:10.1371/journal.pcbi.1005319)
Supplement: S1 Text — (DOCX) [file pcbi.1005319.s001.docx]

**Supplementary data for the paper:**

Entropy Transfer between Residue Pairs Shows that Allostery is an Intrinsic Property of Proteins:

Quantifying Allosteric Communication in Ubiquitin

by

Aysima Hacisuleyman, Burak Erman

The data for the trajectories generated from the simulations is available upon request from the authors, email: [berman@ku.edu.tr](mailto:berman@ku.edu.tr) and [ahacisuleyman@ku.edu.tr](mailto:ahacisuleyman@ku.edu.tr)

**Contents (Each data set is separated from the previous and following sets by page breaks)**

Data for Figure 2a and b

Data for Figure 3a and b

Data for Figures 4 and 5

Data for Figure 6

Data for Figure 7

Data for Figure 8

Data for Figure 9

Data for Figure 10

Data for Figure 11

**Data for Figure 2:** The following is the data used for drawing Fig. 2. The first two columns are residue indices, the third column is the Pearson correlation value. Fig. 2 is drawn using the commercial software Surfer, which makes it possible to plot the negative and positive correlations in separate panels.

1 1 1.00000000000000

1 2 0.694915764613744

1 3 0.404787721168124

1 4 0.109677197898175

1 5 -0.201007176636395

1 6 -0.306463474858114

1 7 -0.262163319154705

1 8 -0.203528452677179

1 9 -0.237097387399938

1 10 -0.167816385303759

1 11 -0.258215489125227

1 12 -0.174124010551221

1 13 -0.132749567448177

1 14 0.110650097674548

1 15 0.205471672507949

1 16 0.585247160264942

1 17 0.571829993976819

1 18 0.574522273921016

1 19 0.410692552983945

1 20 0.291978857180327

1 21 0.205472782416032

1 22 -0.0666006648511683

1 23 -0.247017465782482

1 24 -0.294962427606561

1 25 -0.191015904326312

1 26 -0.275463760274303

1 27 -0.381846437186712

1 28 -0.373756596521663

1 29 -0.398015080937856

1 30 -0.447424757287630

1 31 -0.429894742443516

1 32 -0.425689081683833

1 33 -0.393235700788526

1 34 -0.371376436692300

1 35 -0.375175528971758

1 36 -0.360019011139020

1 37 -0.320651658225510

1 38 -0.311272524053436

1 39 -0.208117387639577

1 40 -0.259978060430438

1 41 -0.333481407154462

1 42 -0.238982475167763

1 43 -0.324351958299296

1 44 -0.363668778103790

1 45 -0.339392458626044

1 46 -0.241273852211821

1 47 -0.238612126082988

1 48 -0.288459974785120

1 49 -0.343371575030456

1 50 -0.357434843135184

1 51 -0.336863834655617

1 52 -0.312930592945855

1 53 -0.263323324790404

1 54 -0.187306716192574

1 55 0.00161975121459823

1 56 0.106354915769619

1 57 0.188181824696145

1 58 0.0376796638934549

1 59 -0.0160547176727609

1 60 0.125971993152701

1 61 0.167641580652979

1 62 0.311458962757144

1 63 0.523864476864804

1 64 0.406974482749447

1 65 0.166230911135789

1 66 -0.0834506195366055

1 67 -0.229677588303832

1 68 -0.378391502109759

1 69 -0.307003216291648

1 70 -0.155490371870077

1 71 0.0784434542165613

1 72 0.262599997801985

1 73 0.399877569526604

1 74 0.419872618637050

1 75 0.382063211096171

1 76 0.345559441033392

2 1 0.694915764613744

2 2 0.999999999999997

2 3 0.685019566181355

2 4 0.328549888832655

2 5 -0.0205964312847704

2 6 -0.176763758632188

2 7 -0.185262230733978

2 8 -0.186560745341532

2 9 -0.213414006807204

2 10 -0.141749532049375

2 11 -0.156188202764014

2 12 -0.0139117357144907

2 13 0.0141204347155036

2 14 0.322429560524904

2 15 0.439907058245763

2 16 0.594758695396943

2 17 0.441757692345019

2 18 0.358206548344779

2 19 0.216046007089258

2 20 0.110255421308234

2 21 0.00509914093121652

2 22 -0.248414724946459

2 23 -0.336236578402397

2 24 -0.351954137983526

2 25 -0.273012266020904

2 26 -0.312708500539056

2 27 -0.367001708701519

2 28 -0.356660358695621

2 29 -0.370127276762385

2 30 -0.389275012683159

2 31 -0.374233980955825

2 32 -0.375358036172988

2 33 -0.343007738916783

2 34 -0.319224567833957

2 35 -0.328791847857410

2 36 -0.318569254200542

2 37 -0.294619878606963

2 38 -0.282201168745666

2 39 -0.187254357838571

2 40 -0.243264611639638

2 41 -0.305478672697405

2 42 -0.197719938109935

2 43 -0.297992187610777

2 44 -0.349827537451651

2 45 -0.336902421893128

2 46 -0.237253882470968

2 47 -0.235745454221533

2 48 -0.271687376845305

2 49 -0.334358559218230

2 50 -0.366089278004245

2 51 -0.396291609145687

2 52 -0.394365884751820

2 53 -0.385852043075419

2 54 -0.347220724838992

2 55 -0.185888618549342

2 56 -0.0654586241962497

2 57 0.0215873353210165

2 58 -0.123506426601523

2 59 -0.122029212189385

2 60 0.0366690512777581

2 61 0.118634806044839

2 62 0.264107898983485

2 63 0.514414424223435

2 64 0.525437930286194

2 65 0.231289013165045

2 66 0.0236545498989181

2 67 -0.139487355681989

2 68 -0.306943533183460

2 69 -0.294763293909597

2 70 -0.152489834494178

2 71 0.0207992246948877

2 72 0.300289497303322

2 73 0.365754622146734

2 74 0.423218359567439

2 75 0.369500529198289

2 76 0.337418423723743

3 1 0.404787721168124

3 2 0.685019566181355

3 3 1.00000000000000

3 4 0.615717542709657

3 5 0.233679383919388

3 6 0.0151588127614624

3 7 -0.108634682421774

3 8 -0.160963936579643

3 9 -0.188647475123891

3 10 -0.122770263570183

3 11 -0.0589487263676278

3 12 0.131124318414594

3 13 0.168937761263459

3 14 0.431108128631040

3 15 0.491581212522213

3 16 0.411565598874237

3 17 0.306793231970123

3 18 0.192086825880913

3 19 0.0799326376361603

3 20 -0.00370540737094004

3 21 -0.0790742526726077

3 22 -0.247730042917143

3 23 -0.279677600418929

3 24 -0.264493373018982

3 25 -0.253256409020796

3 26 -0.264390562924653

3 27 -0.252136005465287

3 28 -0.247508680844451

3 29 -0.279252076993169

3 30 -0.267761131639392

3 31 -0.236269127676237

3 32 -0.253603098125204

3 33 -0.238990599280122

3 34 -0.216226716077449

3 35 -0.217882434312016

3 36 -0.207262148766091

3 37 -0.178908736121468

3 38 -0.177367726560218

3 39 -0.113169892039849

3 40 -0.155615652046401

3 41 -0.213260797882060

3 42 -0.158862335503836

3 43 -0.210881975902035

3 44 -0.230485326002816

3 45 -0.216364634272688

3 46 -0.154877802019344

3 47 -0.165721312799190

3 48 -0.187622499642693

3 49 -0.220319213954468

3 50 -0.241735443859260

3 51 -0.260151434118674

3 52 -0.275039819629480

3 53 -0.269094822820421

3 54 -0.258148837495862

3 55 -0.171618639525314

3 56 -0.0976954343520798

3 57 -0.0483866074542978

3 58 -0.118762572691842

3 59 -0.0935865131770527

3 60 -0.00812714645261742

3 61 0.0670453458538874

3 62 0.175261650828467

3 63 0.359211820009578

3 64 0.456262744491631

3 65 0.340967311968212

3 66 0.253883943786878

3 67 0.103379837151305

3 68 -0.132644802498409

3 69 -0.206959799950345

3 70 -0.138437473909594

3 71 -0.0323621992296731

3 72 0.153802148411551

3 73 0.207940927738745

3 74 0.263914716349675

3 75 0.229129345121530

3 76 0.213077320078042

4 1 0.109677197898175

4 2 0.328549888832655

4 3 0.615717542709657

4 4 1.00000000000000

4 5 0.626706886120025

4 6 0.344749032674708

4 7 0.132234480555929

4 8 -0.0462098977717844

4 9 -0.0275960473982381

4 10 0.00349404206419043

4 11 0.255196961841991

4 12 0.422432062046629

4 13 0.481960291713178

4 14 0.591843477355679

4 15 0.471453186227749

4 16 0.241563774594008

4 17 0.113173065514683

4 18 -0.0487926533481302

4 19 -0.156080925458852

4 20 -0.212353436247528

4 21 -0.214542312637246

4 22 -0.305099757560429

4 23 -0.245238349032680

4 24 -0.175648077169164

4 25 -0.199094186707750

4 26 -0.189459381834294

4 27 -0.113363172223059

4 28 -0.102797995272874

4 29 -0.140159331951942

4 30 -0.107785239849451

4 31 -0.0795799204981665

4 32 -0.106812799066177

4 33 -0.105440419247061

4 34 -0.0838331068723361

4 35 -0.0864318816754557

4 36 -0.0871012710543748

4 37 -0.0713762889548722

4 38 -0.0623160967263961

4 39 -0.0301718031402131

4 40 -0.0674169870639001

4 41 -0.0873623902243927

4 42 -0.0249155898032938

4 43 -0.0468510775184187

4 44 -0.0681939658954173

4 45 -0.0962550119308787

4 46 -0.0674582042403757

4 47 -0.0927218849017192

4 48 -0.0628414300200551

4 49 -0.0798807263026438

4 50 -0.141152447657373

4 51 -0.198127432619954

4 52 -0.222940200952871

4 53 -0.254810337951852

4 54 -0.299774471649710

4 55 -0.290751264377431

4 56 -0.264494650620492

4 57 -0.259043206754195

4 58 -0.275438905322787

4 59 -0.225349123305456

4 60 -0.191056166119653

4 61 -0.142138560746488

4 62 -0.0790638583386400

4 63 0.0506504457184348

4 64 0.279611877057860

4 65 0.309469141018632

4 66 0.510433179021048

4 67 0.371464012878895

4 68 0.118214455241313

4 69 -0.0590512835590032

4 70 -0.0850647455188047

4 71 -0.165576592728672

4 72 0.0768834199668973

4 73 0.0116800741317465

4 74 0.0882060877966419

4 75 0.0750597399637932

4 76 0.0931191720011855

5 1 -0.201007176636395

5 2 -0.0205964312847704

5 3 0.233679383919388

5 4 0.626706886120025

5 5 1.00000000000000

5 6 0.695945671905614

5 7 0.378499084952799

5 8 0.154231447145079

5 9 0.168702115746772

5 10 0.147512639835372

5 11 0.422932595005868

5 12 0.494173811647374

5 13 0.579133275174031

5 14 0.402726299637542

5 15 0.308302017185341

5 16 0.0135049683137422

5 17 -0.0869990263855602

5 18 -0.231170102680147

5 19 -0.298158260490308

5 20 -0.291840832742681

5 21 -0.219886178641454

5 22 -0.173032421885750

5 23 -0.0444064902543858

5 24 0.0530818877862531

5 25 -0.00116780884348275

5 26 0.0259265784238789

5 27 0.149428095773244

5 28 0.161410143698846

5 29 0.112298476616554

5 30 0.137815389482785

5 31 0.150469307005218

5 32 0.115744259155435

5 33 0.0814089002415942

5 34 0.0774254847512447

5 35 0.0869295533856632

5 36 0.0754968753917542

5 37 0.0910462668736993

5 38 0.122551834848013

5 39 0.0920139082111529

5 40 0.0626570309130611

5 41 0.131282733672467

5 42 0.168980857550469

5 43 0.193526210495431

5 44 0.167523959190606

5 45 0.0941522415801021

5 46 0.0521769982495882

5 47 0.0416922762381286

5 48 0.0796871643706093

5 49 0.0975672743814395

5 50 0.0505965917774069

5 51 -0.000136213143166742

5 52 -0.0227185290185540

5 53 -0.0665821997875255

5 54 -0.155297736830575

5 55 -0.241283854309956

5 56 -0.298274878987262

5 57 -0.348942861244660

5 58 -0.297063042509064

5 59 -0.254353306572235

5 60 -0.298898784707803

5 61 -0.277498229383877

5 62 -0.267885739596285

5 63 -0.250016985360942

5 64 -0.0577330685436099

5 65 0.0771284349476222

5 66 0.368634432105665

5 67 0.512832206473162

5 68 0.415372490701421

5 69 0.257102576478082

5 70 0.0839589413004064

5 71 -0.201459557104158

5 72 -0.110918030698283

5 73 -0.257354749519812

5 74 -0.226003658416131

5 75 -0.187099774468439

5 76 -0.150948455973533

6 1 -0.306463474858114

6 2 -0.176763758632188

6 3 0.0151588127614624

6 4 0.344749032674708

6 5 0.695945671905614

6 6 0.999999999999995

6 7 0.626987034984111

6 8 0.441329005782121

6 9 0.390658280267609

6 10 0.363303154271784

6 11 0.518567979934232

6 12 0.459638024538444

6 13 0.383163056074227

6 14 0.180613301856258

6 15 0.133506793785132

6 16 -0.0978539667134716

6 17 -0.153192121899141

6 18 -0.274689970331893

6 19 -0.306192260765587

6 20 -0.258131019223686

6 21 -0.155833866549945

6 22 -0.0383874050444743

6 23 0.0843929322776651

6 24 0.174290473374360

6 25 0.102417811702511

6 26 0.111207829615984

6 27 0.231769059678190

6 28 0.227057595173839

6 29 0.161979704930306

6 30 0.153159425394540

6 31 0.158083001545737

6 32 0.122418188931772

6 33 0.0752075564712551

6 34 0.0573002287474190

6 35 0.0617003675624616

6 36 0.0491055848021803

6 37 0.113027277636953

6 38 0.199936500588730

6 39 0.166953589293290

6 40 0.0946805506009658

6 41 0.196683060438676

6 42 0.234881282578235

6 43 0.227162701079510

6 44 0.202686579733856

6 45 0.0929848110534830

6 46 0.0416354686692751

6 47 0.0301562035766109

6 48 0.0479172454005287

6 49 0.0846959491506250

6 50 0.0787685020052290

6 51 0.0700736217416641

6 52 0.0703096309701512

6 53 0.0700773166255978

6 54 -0.0117621950934719

6 55 -0.132889115908474

6 56 -0.231928349619694

6 57 -0.291546205109951

6 58 -0.212252478123496

6 59 -0.214888119723245

6 60 -0.278736361728735

6 61 -0.307567017329528

6 62 -0.335058912284400

6 63 -0.336130001022111

6 64 -0.187831930691736

6 65 -0.0150792355818286

6 66 0.279408884259847

6 67 0.404364637428578

6 68 0.508901861291459

6 69 0.349185906652356

6 70 0.105589668894192

6 71 -0.234271183505370

6 72 -0.197869639675652

6 73 -0.380943144122360

6 74 -0.302422107042951

6 75 -0.264766052064126

6 76 -0.241312292598353

7 1 -0.262163319154705

7 2 -0.185262230733978

7 3 -0.108634682421774

7 4 0.132234480555929

7 5 0.378499084952799

7 6 0.626987034984111

7 7 1.00000000000001

7 8 0.869314206883172

7 9 0.780443747096530

7 10 0.486794893366930

7 11 0.498709335422628

7 12 0.212592407276957

7 13 0.232682960325197

7 14 0.0888089036593038

7 15 0.0245591535816585

7 16 -0.120108298494329

7 17 -0.167041819957901

7 18 -0.243843652158710

7 19 -0.266079357816142

7 20 -0.232341873988010

7 21 -0.156222270805604

7 22 -0.0738385802035917

7 23 0.0212948704036284

7 24 0.104969048737220

7 25 0.0485505480243076

7 26 0.0477864710149721

7 27 0.154993768295841

7 28 0.153253811920676

7 29 0.0767813854370499

7 30 0.0536766842201580

7 31 0.0644345282838046

7 32 0.0385677385741031

7 33 -0.0146595731254904

7 34 -0.0295009133833166

7 35 -0.0257541348870594

7 36 -0.0309678544220381

7 37 0.0392793430807221

7 38 0.176631066026327

7 39 0.180757746717007

7 40 0.0282089075191866

7 41 0.163146758085739

7 42 0.315350323436901

7 43 0.209801628502481

7 44 0.150689710646651

7 45 0.0186484980719762

7 46 -0.00587568719953713

7 47 0.00533784138878882

7 48 -0.00106341693814065

7 49 -0.0270990682316091

7 50 -0.0245647959864775

7 51 -0.0658308445011280

7 52 -0.0800945043023872

7 53 -0.0251944943159643

7 54 -0.0695713827248708

7 55 -0.160171759837404

7 56 -0.233857711762147

7 57 -0.245228721320913

7 58 -0.206366044348667

7 59 -0.218958849130159

7 60 -0.213703577293040

7 61 -0.269963816525498

7 62 -0.286871210209610

7 63 -0.293266379561740

7 64 -0.202897789925781

7 65 -0.0875014018000768

7 66 0.0853182234968921

7 67 0.170123687646175

7 68 0.470849021183741

7 69 0.280156886806839

7 70 0.0344068064332221

7 71 -0.381626158576023

7 72 -0.134671260712336

7 73 -0.382347388520186

7 74 -0.313483013846974

7 75 -0.255910790979713

7 76 -0.219704542547352

8 1 -0.203528452677179

8 2 -0.186560745341532

8 3 -0.160963936579643

8 4 -0.0462098977717844

8 5 0.154231447145079

8 6 0.441329005782121

8 7 0.869314206883172

8 8 1.00000000000002

8 9 0.816829753851585

8 10 0.564821305001739

8 11 0.265883648426403

8 12 -0.0522356706663771

8 13 -0.0513872772302014

8 14 -0.132010141430711

8 15 -0.0953581387846691

8 16 -0.143364722195746

8 17 -0.125342231512671

8 18 -0.135268011320274

8 19 -0.118202701805832

8 20 -0.0620075600234671

8 21 -0.0244782681628445

8 22 0.0564912250707822

8 23 0.0920940994319766

8 24 0.133446606384875

8 25 0.0725375174861271

8 26 0.0552509666301330

8 27 0.126290624942890

8 28 0.104332193523483

8 29 0.0212640522523256

8 30 -0.0242194293114669

8 31 -0.0145643686646280

8 32 -0.0375019042651696

8 33 -0.0876806601737419

8 34 -0.105385676186647

8 35 -0.101314158526662

8 36 -0.0944234624618153

8 37 0.00882238202160917

8 38 0.159172897291155

8 39 0.175493127640315

8 40 0.00832973827440960

8 41 0.132040759460338

8 42 0.254511519860383

8 43 0.143402425232402

8 44 0.104618923478915

8 45 -0.0109885130435843

8 46 -0.0222138881221130

8 47 -0.00854607907797244

8 48 -0.0538457068907684

8 49 -0.0761229764985935

8 50 -0.0279164757650555

8 51 -0.0172433651116073

8 52 -0.0164419872987444

8 53 0.0858269943815118

8 54 0.0829137548339379

8 55 0.0119070711071081

8 56 -0.0730494602286688

8 57 -0.0599566479956144

8 58 -0.0267126668040945

8 59 -0.0886275648678257

8 60 -0.0790633290590613

8 61 -0.167541895226546

8 62 -0.204628743114408

8 63 -0.216322771593439

8 64 -0.196860916973120

8 65 -0.0772353446804318

8 66 -0.0118769240248881

8 67 0.0746864864453519

8 68 0.383053624566501

8 69 0.247065133458111

8 70 0.000489556794605003

8 71 -0.302493128472257

8 72 -0.177840423648168

8 73 -0.349278122356397

8 74 -0.268518653079965

8 75 -0.221790195908086

8 76 -0.208377081966586

9 1 -0.237097387399938

9 2 -0.213414006807204

9 3 -0.188647475123891

9 4 -0.0275960473982381

9 5 0.168702115746772

9 6 0.390658280267609

9 7 0.780443747096530

9 8 0.816829753851585

9 9 0.999999999999997

9 10 0.706564307973629

9 11 0.528105827996173

9 12 0.144420110902588

9 13 0.0810995856667437

9 14 -0.0396347317690274

9 15 -0.0573360125420879

9 16 -0.126626466683442

9 17 -0.136260813102943

9 18 -0.176102396728374

9 19 -0.182724802731652

9 20 -0.139057429222339

9 21 -0.0828390445270875

9 22 -0.00542151838498811

9 23 0.0587283074048232

9 24 0.121748517774907

9 25 0.0712023824632018

9 26 0.0513170367199328

9 27 0.130335130782352

9 28 0.126610798192509

9 29 0.0553047112204582

9 30 0.0227274996819244

9 31 0.0342728177182626

9 32 0.0157375604676797

9 33 -0.0309458875219354

9 34 -0.0428876531915549

9 35 -0.0412648472264909

9 36 -0.0511569721082901

9 37 0.0252984111826687

9 38 0.147823463031005

9 39 0.153275550023854

9 40 0.00809183010486669

9 41 0.113875771686096

9 42 0.228211341953098

9 43 0.141881735699209

9 44 0.0962546479453331

9 45 -0.00356419078863286

9 46 -0.0122856302804550

9 47 -0.00225412876026185

9 48 -0.0230410472410585

9 49 -0.0394943478087960

9 50 -0.0185762876843169

9 51 -0.0305275619314293

9 52 -0.0300134407966494

9 53 0.0293428506583785

9 54 -0.000375292477385943

9 55 -0.0728945320737099

9 56 -0.144957877855534

9 57 -0.151367646014858

9 58 -0.112939503490429

9 59 -0.143298261497118

9 60 -0.150460060943839

9 61 -0.215637293231493

9 62 -0.239498300528107

9 63 -0.268948336978053

9 64 -0.228211655520833

9 65 -0.126880494061276

9 66 -0.0298737540068605

9 67 0.0377343521328912

9 68 0.326026452876261

9 69 0.204622189978389

9 70 -0.00643161080905931

9 71 -0.337844262872113

9 72 -0.153501735866093

9 73 -0.350585875927812

9 74 -0.270266037981862

9 75 -0.216546490437348

9 76 -0.189335441453008

10 1 -0.167816385303759

10 2 -0.141749532049375

10 3 -0.122770263570183

10 4 0.00349404206419043

10 5 0.147512639835372

10 6 0.363303154271784

10 7 0.486794893366930

10 8 0.564821305001739

10 9 0.706564307973629

10 10 1.00000000000001

10 11 0.567432207024461

10 12 0.256177371609145

10 13 0.0562119104114016

10 14 -0.0151291667590529

10 15 0.00231657182795344

10 16 -0.0492933374881567

10 17 -0.0555812257397665

10 18 -0.0998976197004881

10 19 -0.125335685609229

10 20 -0.0819841870301035

10 21 -0.0390586811225638

10 22 0.00483720021771166

10 23 0.0368140273063111

10 24 0.0826294786096462

10 25 0.0484870811300269

10 26 0.0170021414274479

10 27 0.0601433622061883

10 28 0.0492289378827158

10 29 0.00449493010196424

10 30 -0.0234326702958154

10 31 -0.0320415714665830

10 32 -0.0345680732508993

10 33 -0.0468878736110222

10 34 -0.0625169029360133

10 35 -0.0794779733003334

10 36 -0.100108843783055

10 37 -0.0426835403645432

10 38 0.0479812869899542

10 39 0.0511739315044925

10 40 -0.0260506246995999

10 41 0.0614647606680488

10 42 0.139565124125904

10 43 0.0732621074614936

10 44 0.0162263497472655

10 45 -0.0797372212895828

10 46 -0.0673524129904643

10 47 -0.0529222337295284

10 48 -0.0623166764738033

10 49 -0.0572263263050114

10 50 -0.0311197170401487

10 51 -0.0109732067953942

10 52 -0.00365135644766335

10 53 0.0292032481984742

10 54 0.00258718746140266

10 55 -0.0514928241455779

10 56 -0.111114565129284

10 57 -0.114791784885550

10 58 -0.0949396451829267

10 59 -0.135466145076814

10 60 -0.144105953406041

10 61 -0.193750689562569

10 62 -0.208152797000215

10 63 -0.221728819587507

10 64 -0.185619399027195

10 65 -0.104561782758636

10 66 -0.0540596846564171

10 67 -0.00769686709595837

10 68 0.162716416966411

10 69 0.168461452728832

10 70 0.0434923955188294

10 71 -0.146866733918188

10 72 -0.0427778157988669

10 73 -0.182810100980987

10 74 -0.124313255123062

10 75 -0.109892939739919

10 76 -0.119724478541315

11 1 -0.258215489125227

11 2 -0.156188202764014

11 3 -0.0589487263676278

11 4 0.255196961841991

11 5 0.422932595005868

11 6 0.518567979934232

11 7 0.498709335422628

11 8 0.265883648426403

11 9 0.528105827996173

11 10 0.567432207024461

11 11 0.999999999999992

11 12 0.726532701230431

11 13 0.552861659300645

11 14 0.360595898493754

11 15 0.201459186166468

11 16 1.13555121412390e-05

11 17 -0.105086885978813

11 18 -0.254013892124429

11 19 -0.334423603548888

11 20 -0.328096569220802

11 21 -0.215823230132680

11 22 -0.162518384277448

11 23 -0.0357695884438525

11 24 0.0747511182212782

11 25 0.0466150405479170

11 26 0.0393526860270639

11 27 0.136347458058645

11 28 0.165258930139613

11 29 0.130164009121799

11 30 0.135068468629995

11 31 0.141501747831080

11 32 0.133423895714071

11 33 0.108181788892065

11 34 0.0974643853860983

11 35 0.0889606259187863

11 36 0.0466202455972743

11 37 0.0521359689975567

11 38 0.0989738856931152

11 39 0.0714293264423932

11 40 0.0166845996858476

11 41 0.0804708741151445

11 42 0.151092515929084

11 43 0.133591662520087

11 44 0.0761019878835547

11 45 0.00138398628784897

11 46 -0.000712302471094494

11 47 -0.00522680329043755

11 48 0.0434309856111962

11 49 0.0459061552259240

11 50 -0.000153169851429767

11 51 -0.0560811076105973

11 52 -0.0534083538598795

11 53 -0.105912842554103

11 54 -0.204151237345295

11 55 -0.285961251623551

11 56 -0.328155145522425

11 57 -0.387848782217181

11 58 -0.351999835952509

11 59 -0.315689845451895

11 60 -0.348554403001546

11 61 -0.336650827650494

11 62 -0.321006007564857

11 63 -0.340346172769131

11 64 -0.201505727696206

11 65 -0.137465190128512

11 66 0.0816172225191489

11 67 0.100341577740619

11 68 0.230342068799503

11 69 0.135741313945653

11 70 0.0238325660301289

11 71 -0.305206380558169

11 72 -0.0572132080547358

11 73 -0.268191930741332

11 74 -0.206883188516317

11 75 -0.161926345208274

11 76 -0.113190260194091

12 1 -0.174124010551221

12 2 -0.0139117357144907

12 3 0.131124318414594

12 4 0.422432062046629

12 5 0.494173811647374

12 6 0.459638024538444

12 7 0.212592407276957

12 8 -0.0522356706663771

12 9 0.144420110902588

12 10 0.256177371609145

12 11 0.726532701230431

12 12 1.00000000000000

12 13 0.708024260002319

12 14 0.545965784967897

12 15 0.354313007462273

12 16 0.101800404530680

12 17 -0.0386072964978973

12 18 -0.215634586422310

12 19 -0.314974102924817

12 20 -0.333486616379730

12 21 -0.244115031918174

12 22 -0.235777716629275

12 23 -0.121079381080112

12 24 -0.0168043755803985

12 25 -0.0307098786660949

12 26 -0.0349383358999136

12 27 0.0437065433275091

12 28 0.0785223328011769

12 29 0.0704469866782385

12 30 0.0891332541247470

12 31 0.0902099114084912

12 32 0.0908697772572996

12 33 0.0961240833551550

12 34 0.0880687998182369

12 35 0.0773378254838564

12 36 0.0390834809335888

12 37 0.0120300723412113

12 38 0.00808448139219226

12 39 -0.0163729046068477

12 40 -0.00144983980482993

12 41 0.0216481996165506

12 42 0.0581721045930726

12 43 0.0577780064644119

12 44 0.000779433529814264

12 45 -0.0446682571897375

12 46 -0.0359173230453875

12 47 -0.0442790540756840

12 48 0.0181339207519091

12 49 0.0285316847971507

12 50 -0.0300668604430926

12 51 -0.0878261452650863

12 52 -0.0938505766610064

12 53 -0.176691348511413

12 54 -0.273715042252644

12 55 -0.330539562473142

12 56 -0.340735367765571

12 57 -0.401950037036803

12 58 -0.385015058748720

12 59 -0.325797300006535

12 60 -0.346597260001729

12 61 -0.295130486437865

12 62 -0.260048017924388

12 63 -0.244315861178220

12 64 -0.0735998405814080

12 65 -0.0435226146200891

12 66 0.149735748633964

12 67 0.120265819638336

12 68 0.112599495348414

12 69 0.0486985039130619

12 70 0.0209676416438523

12 71 -0.185489894709651

12 72 0.0491457845391648

12 73 -0.108618392004671

12 74 -0.0551981193885046

12 75 -0.0379409132272376

12 76 0.00118467232102547

13 1 -0.132749567448177

13 2 0.0141204347155036

13 3 0.168937761263459

13 4 0.481960291713178

13 5 0.579133275174031

13 6 0.383163056074227

13 7 0.232682960325197

13 8 -0.0513872772302014

13 9 0.0810995856667437

13 10 0.0562119104114016

13 11 0.552861659300645

13 12 0.708024260002319

13 13 1.00000000000000

13 14 0.713410466997312

13 15 0.426418612308288

13 16 0.131904684636645

13 17 -0.0181174493014572

13 18 -0.193564235471580

13 19 -0.296641226779635

13 20 -0.323678153640599

13 21 -0.234753133787452

13 22 -0.246550935886173

13 23 -0.122509562861239

13 24 -0.0135352296765905

13 25 -0.0353407414951204

13 26 -0.0271499303512438

13 27 0.0729052293278508

13 28 0.119527953901730

13 29 0.0811688019980081

13 30 0.110299544777560

13 31 0.140622577783771

13 32 0.122087501900423

13 33 0.0980403760411711

13 34 0.110089893388487

13 35 0.119999841679231

13 36 0.0932795178312974

13 37 0.0651384361880125

13 38 0.0552034604893422

13 39 0.0315132466155207

13 40 0.00937244833172256

13 41 0.0178053886828896

13 42 0.0596058079762999

13 43 0.0764135318505758

13 44 0.0416868099329541

13 45 0.0279400978101689

13 46 0.0346354074815275

13 47 0.0134903859971955

13 48 0.0810285030503207

13 49 0.0683987588110633

13 50 -0.0122873615870891

13 51 -0.0995565236328177

13 52 -0.116819048982305

13 53 -0.192255640928905

13 54 -0.286167277268263

13 55 -0.331436332292700

13 56 -0.336369711768487

13 57 -0.407270989144545

13 58 -0.397250930855526

13 59 -0.324591782199784

13 60 -0.341682917392304

13 61 -0.281166358559638

13 62 -0.240422264762972

13 63 -0.232467324688231

13 64 -0.0515983953466460

13 65 -0.0445475994820903

13 66 0.163392157096818

13 67 0.160824534145214

13 68 0.160680820727156

13 69 0.0152237305870807

13 70 -0.0351298054502622

13 71 -0.274407470309025

13 72 -0.0114271127673640

13 73 -0.154469008285481

13 74 -0.123856449295456

13 75 -0.0849731780136107

13 76 -0.0204111540213531

14 1 0.110650097674548

14 2 0.322429560524904

14 3 0.431108128631040

14 4 0.591843477355679

14 5 0.402726299637542

14 6 0.180613301856258

14 7 0.0888089036593038

14 8 -0.132010141430711

14 9 -0.0396347317690274

14 10 -0.0151291667590529

14 11 0.360595898493754

14 12 0.545965784967897

14 13 0.713410466997312

14 14 0.999999999999999

14 15 0.694211704674484

14 16 0.387032942294046

14 17 0.189558659330452

14 18 -0.0245561069065945

14 19 -0.171050220605081

14 20 -0.246553218682800

14 21 -0.222625372254666

14 22 -0.336395067902052

14 23 -0.258425393158704

14 24 -0.164829157038599

14 25 -0.163032499077102

14 26 -0.166944664114472

14 27 -0.0933813115337207

14 28 -0.0662048939056276

14 29 -0.108728571938027

14 30 -0.0702630326151692

14 31 -0.0446655789349506

14 32 -0.0750428461535030

14 33 -0.0699746433496547

14 34 -0.0419398958183925

14 35 -0.0396348281416773

14 36 -0.0453135075026612

14 37 -0.0701069474123406

14 38 -0.0775857864021485

14 39 -0.0501438639582860

14 40 -0.0720485359003425

14 41 -0.101491325594376

14 42 -0.0275737140289690

14 43 -0.0736030459646048

14 44 -0.137827373912132

14 45 -0.151351429400180

14 46 -0.0893677548305347

14 47 -0.0902295949168377

14 48 -0.0418749881309828

14 49 -0.0957815190829245

14 50 -0.177290994440649

14 51 -0.265704401788561

14 52 -0.264364302545700

14 53 -0.336299508127195

14 54 -0.410692136005659

14 55 -0.389368923904673

14 56 -0.340230291520777

14 57 -0.354717990892345

14 58 -0.414767467577902

14 59 -0.356121586369667

14 60 -0.295885974595362

14 61 -0.212682056623568

14 62 -0.115368319204551

14 63 -0.0129026497099316

14 64 0.162413136425149

14 65 0.0632619199446873

14 66 0.161975819193277

14 67 0.0417640148207787

14 68 -0.0507164852138570

14 69 -0.137204573269290

14 70 -0.0776320360106196

14 71 -0.178536334741417

14 72 0.182400390132313

14 73 0.0565955916061417

14 74 0.0939727491058876

14 75 0.107363423604941

14 76 0.151171038604455

15 1 0.205471672507949

15 2 0.439907058245763

15 3 0.491581212522213

15 4 0.471453186227749

15 5 0.308302017185341

15 6 0.133506793785132

15 7 0.0245591535816585

15 8 -0.0953581387846691

15 9 -0.0573360125420879

15 10 0.00231657182795344

15 11 0.201459186166468

15 12 0.354313007462273

15 13 0.426418612308288

15 14 0.694211704674484

15 15 0.999999999999998

15 16 0.657075352230026

15 17 0.418438716581898

15 18 0.150488553615083

15 19 -0.0387475794355452

15 20 -0.100089000006528

15 21 -0.0593063306276198

15 22 -0.185546279733735

15 23 -0.140335797192287

15 24 -0.0643172971584770

15 25 -0.0701068661693916

15 26 -0.0861935900655475

15 27 -0.0359847299068221

15 28 -0.0337864430593122

15 29 -0.131080740670204

15 30 -0.127575500255701

15 31 -0.0991949471360018

15 32 -0.151849996014795

15 33 -0.169257520577108

15 34 -0.148891109158852

15 35 -0.128205391913821

15 36 -0.108117670453807

15 37 -0.0788637010171298

15 38 -0.0551956637825089

15 39 -0.0167383728326960

15 40 -0.0645578777970124

15 41 -0.0912983059749021

15 42 -0.0613508468142954

15 43 -0.104541691270775

15 44 -0.150344506719344

15 45 -0.159399685244621

15 46 -0.0759320902951405

15 47 -0.0691605160939374

15 48 -0.0650705539743671

15 49 -0.123412531196502

15 50 -0.177897443730812

15 51 -0.219696096999181

15 52 -0.177629788505407

15 53 -0.218059739325985

15 54 -0.290300655553292

15 55 -0.251650802549309

15 56 -0.227940641179067

15 57 -0.256802379119630

15 58 -0.325945005522618

15 59 -0.310707755952353

15 60 -0.267713159062334

15 61 -0.179116698113568

15 62 -0.0820870957068049

15 63 0.0702893764356490

15 64 0.169529588324651

15 65 0.0500919920697234

15 66 0.0859311881875174

15 67 0.00954372890591192

15 68 -0.0747039893479449

15 69 -0.132555921793973

15 70 -0.0891894672215930

15 71 -0.119287321069909

15 72 0.118622358715248

15 73 0.0488964482539580

15 74 0.0730692787116598

15 75 0.0964701206149820

15 76 0.127647388930710

16 1 0.585247160264942

16 2 0.594758695396943

16 3 0.411565598874237

16 4 0.241563774594008

16 5 0.0135049683137422

16 6 -0.0978539667134716

16 7 -0.120108298494329

16 8 -0.143364722195746

16 9 -0.126626466683442

16 10 -0.0492933374881567

16 11 1.13555121412390e-05

16 12 0.101800404530680

16 13 0.131904684636645

16 14 0.387032942294046

16 15 0.657075352230026

16 16 1

16 17 0.722993067443087

16 18 0.458510277282837

16 19 0.241970358546202

16 20 0.159566586682669

16 21 0.147363728825728

16 22 -0.0809126497841367

16 23 -0.154997427458648

16 24 -0.143087500813241

16 25 -0.0847072003528855

16 26 -0.161917380682427

16 27 -0.191060721659167

16 28 -0.195316079209083

16 29 -0.288044794949019

16 30 -0.328698697005226

16 31 -0.307141226093305

16 32 -0.343595561132973

16 33 -0.340735113695422

16 34 -0.319819497566268

16 35 -0.307644948118979

16 36 -0.279364359346101

16 37 -0.228068209198210

16 38 -0.192765048617536

16 39 -0.114799881009829

16 40 -0.181728942138213

16 41 -0.230827698209262

16 42 -0.166523220657569

16 43 -0.229409827642594

16 44 -0.282058673094612

16 45 -0.272712043069888

16 46 -0.167888874799495

16 47 -0.160989722810242

16 48 -0.178706778678077

16 49 -0.242726174050365

16 50 -0.280643567442670

16 51 -0.296217470049655

16 52 -0.237610704180043

16 53 -0.243636013992326

16 54 -0.246677582722690

16 55 -0.107177290580992

16 56 -0.0466288465665676

16 57 -0.0321631985865295

16 58 -0.170905719975078

16 59 -0.212826644651397

16 60 -0.121353301119025

16 61 -0.0335647998909061

16 62 0.102335378759584

16 63 0.314789416730528

16 64 0.272981145565360

16 65 0.0497634119905790

16 66 -0.0589710376347938

16 67 -0.169152281003441

16 68 -0.266286041769471

16 69 -0.239611726292246

16 70 -0.130799286266176

16 71 -0.0379226682402305

16 72 0.194749327024355

16 73 0.230304233673194

16 74 0.241877813060940

16 75 0.246450267539318

16 76 0.257294768274865

17 1 0.571829993976819

17 2 0.441757692345019

17 3 0.306793231970123

17 4 0.113173065514683

17 5 -0.0869990263855602

17 6 -0.153192121899141

17 7 -0.167041819957901

17 8 -0.125342231512671

17 9 -0.136260813102943

17 10 -0.0555812257397665

17 11 -0.105086885978813

17 12 -0.0386072964978973

17 13 -0.0181174493014572

17 14 0.189558659330452

17 15 0.418438716581898

17 16 0.722993067443087

17 17 0.999999999999999

17 18 0.747566756464599

17 19 0.471948798452306

17 20 0.364173039406935

17 21 0.391825375565801

17 22 0.144094005428534

17 23 -0.00792363230208744

17 24 -0.0201622541352432

17 25 0.0205586460313993

17 26 -0.102498147122041

17 27 -0.147676675795573

17 28 -0.165674033530541

17 29 -0.289317088697746

17 30 -0.355303696219890

17 31 -0.322608904339820

17 32 -0.369070959652132

17 33 -0.382133448338976

17 34 -0.368021239200574

17 35 -0.340216629595950

17 36 -0.299848104546783

17 37 -0.214957029627248

17 38 -0.176770545042085

17 39 -0.0920334130131226

17 40 -0.168361907921924

17 41 -0.216764203718179

17 42 -0.189974053778188

17 43 -0.229196698632338

17 44 -0.253530609940193

17 45 -0.244408281672575

17 46 -0.146246425982879

17 47 -0.131070928249911

17 48 -0.178206439676509

17 49 -0.220518300528820

17 50 -0.227426352885942

17 51 -0.178684981012587

17 52 -0.0980685573902540

17 53 -0.0444016512895811

17 54 -0.0100671613253973

17 55 0.145059715038444

17 56 0.182644128149903

17 57 0.171577327399390

17 58 0.0395914158074279

17 59 -0.0495168815369556

17 60 -0.00157054061629202

17 61 0.0422517676292272

17 62 0.119069935775373

17 63 0.278297238245613

17 64 0.167048071985887

17 65 0.0170650503716018

17 66 -0.107447620925034

17 67 -0.184907922913075

17 68 -0.272096143169662

17 69 -0.211498313847438

17 70 -0.118824803176950

17 71 0.0275795224139962

17 72 0.107028031855384

17 73 0.181394451821128

17 74 0.161457379731408

17 75 0.186872323684644

17 76 0.161028278979868

18 1 0.574522273921016

18 2 0.358206548344779

18 3 0.192086825880913

18 4 -0.0487926533481302

18 5 -0.231170102680147

18 6 -0.274689970331893

18 7 -0.243843652158710

18 8 -0.135268011320274

18 9 -0.176102396728374

18 10 -0.0998976197004881

18 11 -0.254013892124429

18 12 -0.215634586422310

18 13 -0.193564235471580

18 14 -0.0245561069065945

18 15 0.150488553615083

18 16 0.458510277282837

18 17 0.747566756464599

18 18 1.00000000000000

18 19 0.737078602374398

18 20 0.568303158633475

18 21 0.525075939235191

18 22 0.246374762720997

18 23 0.00993882337165615

18 24 -0.0603427572073602

18 25 0.0251975646163091

18 26 -0.113411430105125

18 27 -0.220841100716195

18 28 -0.235955632853906

18 29 -0.338397477791136

18 30 -0.413723270473641

18 31 -0.381523969150136

18 32 -0.410878365435310

18 33 -0.417923267198299

18 34 -0.402626544367947

18 35 -0.378190823452128

18 36 -0.340137328906767

18 37 -0.250407232395354

18 38 -0.220523542764027

18 39 -0.121456312253284

18 40 -0.196508960984020

18 41 -0.260307134895567

18 42 -0.228526565852430

18 43 -0.271549832558913

18 44 -0.277570827512710

18 45 -0.252796644434065

18 46 -0.161310498990145

18 47 -0.146464482644840

18 48 -0.227471567726841

18 49 -0.256272021482412

18 50 -0.246674763503752

18 51 -0.174583959208093

18 52 -0.100902030831909

18 53 0.00271807557738989

18 54 0.0994548314189555

18 55 0.308732450606063

18 56 0.366379996278892

18 57 0.405560503541565

18 58 0.260674840991399

18 59 0.139286505528416

18 60 0.197159333965133

18 61 0.174556660213795

18 62 0.193443592141294

18 63 0.310231474469525

18 64 0.151779573178539

18 65 0.0336421815136299

18 66 -0.172670499107002

18 67 -0.233407218563698

18 68 -0.325684504643051

18 69 -0.222555492932657

18 70 -0.121669804197314

18 71 0.112438793325404

18 72 0.0936211638764671

18 73 0.258610762689793

18 74 0.222348609486491

18 75 0.227203424595051

18 76 0.175644459592233

19 1 0.410692552983945

19 2 0.216046007089258

19 3 0.0799326376361603

19 4 -0.156080925458852

19 5 -0.298158260490308

19 6 -0.306192260765587

19 7 -0.266079357816142

19 8 -0.118202701805832

19 9 -0.182724802731652

19 10 -0.125335685609229

19 11 -0.334423603548888

19 12 -0.314974102924817

19 13 -0.296641226779635

19 14 -0.171050220605081

19 15 -0.0387475794355452

19 16 0.241970358546202

19 17 0.471948798452306

19 18 0.737078602374398

19 19 1.00000000000000

19 20 0.722353959974826

19 21 0.505282968314579

19 22 0.294611797746911

19 23 0.0588310352717697

19 24 -0.0545968218986693

19 25 -0.00677579586532289

19 26 -0.115968167389730

19 27 -0.221574254218629

19 28 -0.237531157417950

19 29 -0.308560388833218

19 30 -0.373417405850607

19 31 -0.334936503050867

19 32 -0.347822520099438

19 33 -0.355286707770827

19 34 -0.339807355594865

19 35 -0.312970752382445

19 36 -0.285608674595554

19 37 -0.199587007015105

19 38 -0.185559542949001

19 39 -0.102801993525018

19 40 -0.166784738717257

19 41 -0.228815532845865

19 42 -0.233839991068047

19 43 -0.260214254902903

19 44 -0.247584077913076

19 45 -0.209947232768689

19 46 -0.138425199603699

19 47 -0.129867451555889

19 48 -0.231946385522452

19 49 -0.220083798007458

19 50 -0.177936683191951

19 51 -0.0719481861743629

19 52 -0.0169599082384034

19 53 0.120360001879666

19 54 0.271008396822406

19 55 0.499066893699061

19 56 0.588711877624062

19 57 0.641052655571153

19 58 0.501010786826243

19 59 0.356269627633663

19 60 0.393236749133896

19 61 0.316361799061105

19 62 0.236663513096446

19 63 0.267804951391960

19 64 0.102123109331591

19 65 0.0658561397867725

19 66 -0.169589696770550

19 67 -0.221255535675832

19 68 -0.306253607624683

19 69 -0.188953805317887

19 70 -0.109705436006144

19 71 0.157458870884151

19 72 0.00418560334829195

19 73 0.217887984079161

19 74 0.188018186112703

19 75 0.177845130802452

19 76 0.111096504471596

20 1 0.291978857180327

20 2 0.110255421308234

20 3 -0.00370540737094004

20 4 -0.212353436247528

20 5 -0.291840832742681

20 6 -0.258131019223686

20 7 -0.232341873988010

20 8 -0.0620075600234671

20 9 -0.139057429222339

20 10 -0.0819841870301035

20 11 -0.328096569220802

20 12 -0.333486616379730

20 13 -0.323678153640599

20 14 -0.246553218682800

20 15 -0.100089000006528

20 16 0.159566586682669

20 17 0.364173039406935

20 18 0.568303158633475

20 19 0.722353959974826

20 20 1.00000000000001

20 21 0.739111120351749

20 22 0.489959778195909

20 23 0.246033448247306

20 24 0.114264409145372

20 25 0.137357648808928

20 26 0.0131834134699772

20 27 -0.0851984477585932

20 28 -0.126781337401428

20 29 -0.229178936945467

20 30 -0.299662255216395

20 31 -0.269885731690680

20 32 -0.302417150451330

20 33 -0.322818268098051

20 34 -0.313404972600679

20 35 -0.277451530931778

20 36 -0.240320948078402

20 37 -0.134789033486879

20 38 -0.107087332302609

20 39 -0.0278212070303214

20 40 -0.111768213068709

20 41 -0.169158185504835

20 42 -0.205506525250335

20 43 -0.196967754310593

20 44 -0.165293672923876

20 45 -0.139950538534117

20 46 -0.0924616083219983

20 47 -0.0815065888024994

20 48 -0.171353912589847

20 49 -0.139594390121234

20 50 -0.0846787484416835

20 51 0.0381236674514135

20 52 0.124226754938881

20 53 0.278836561245545

20 54 0.425886850134223

20 55 0.631933198594477

20 56 0.617256901139366

20 57 0.662360674416824

20 58 0.554679319623439

20 59 0.394746431185262

20 60 0.390947126283999

20 61 0.279352877316805

20 62 0.158558149468442

20 63 0.176734529771753

20 64 0.00819459456355705

20 65 0.0142855706153346

20 66 -0.190157613806288

20 67 -0.189161454963075

20 68 -0.239354741699562

20 69 -0.104785302906728

20 70 -0.0825612912725899

20 71 0.162925826185504

20 72 -0.0945008660782621

20 73 0.115595879877636

20 74 0.0540473428950319

20 75 0.0703007081987268

20 76 0.0149559382090580

21 1 0.205472782416032

21 2 0.00509914093121652

21 3 -0.0790742526726077

21 4 -0.214542312637246

21 5 -0.219886178641454

21 6 -0.155833866549945

21 7 -0.156222270805604

21 8 -0.0244782681628445

21 9 -0.0828390445270875

21 10 -0.0390586811225638

21 11 -0.215823230132680

21 12 -0.244115031918174

21 13 -0.234753133787452

21 14 -0.222625372254666

21 15 -0.0593063306276198

21 16 0.147363728825728

21 17 0.391825375565801

21 18 0.525075939235191

21 19 0.505282968314579

21 20 0.739111120351749

21 21 0.999999999999994

21 22 0.733476001428277

21 23 0.471755258974544

21 24 0.348198006624852

21 25 0.372560822484044

21 26 0.219938106859422

21 27 0.123499433201245

21 28 0.0724082636711057

21 29 -0.0817619223315707

21 30 -0.178696004356491

21 31 -0.165727060123104

21 32 -0.227202495269460

21 33 -0.273640649862316

21 34 -0.278469251152545

21 35 -0.228843231894362

21 36 -0.173554329364481

21 37 -0.0529340656993631

21 38 -0.00260024197284340

21 39 0.0497887796921828

21 40 -0.0102283400786511

21 41 -0.0630470860947595

21 42 -0.139406503341548

21 43 -0.0871760009332903

21 44 -0.0579074610040779

21 45 -0.0495056060070213

21 46 -0.0333565450822110

21 47 -0.0206890855218813

21 48 -0.0654309689254546

21 49 -0.0216144931148336

21 50 0.0445276262404399

21 51 0.172201879986832

21 52 0.300257336805718

21 53 0.420397439495774

21 54 0.493951261122026

21 55 0.605351772413063

21 56 0.512813052928819

21 57 0.433759229807823

21 58 0.365199180727984

21 59 0.208026797388547

21 60 0.150139899302295

21 61 0.103025744914019

21 62 0.0129604869942424

21 63 0.0287560266221486

21 64 -0.104223950774917

21 65 -0.0835105352151812

21 66 -0.184774503784311

21 67 -0.142123324153424

21 68 -0.137587852051995

21 69 -0.0250449289470036

21 70 -0.0357044751056152

21 71 0.114775392701083

21 72 -0.172396410313346

21 73 -0.0370085057286659

21 74 -0.126794034067669

21 75 -0.0509311207818949

21 76 -0.0787430804533212

22 1 -0.0666006648511683

22 2 -0.248414724946459

22 3 -0.247730042917143

22 4 -0.305099757560429

22 5 -0.173032421885750

22 6 -0.0383874050444743

22 7 -0.0738385802035917

22 8 0.0564912250707822

22 9 -0.00542151838498811

22 10 0.00483720021771166

22 11 -0.162518384277448

22 12 -0.235777716629275

22 13 -0.246550935886173

22 14 -0.336395067902052

22 15 -0.185546279733735

22 16 -0.0809126497841367

22 17 0.144094005428534

22 18 0.246374762720997

22 19 0.294611797746911

22 20 0.489959778195909

22 21 0.733476001428277

22 22 1.00000000000001

22 23 0.801404658984911

22 24 0.678361331520610

22 25 0.659160342406123

22 26 0.538269650538512

22 27 0.406078208090688

22 28 0.337566953043307

22 29 0.206335740250741

22 30 0.0688351552931894

22 31 0.0358201640306269

22 32 -0.0132936229850689

22 33 -0.0839935838061430

22 34 -0.132418175283688

22 35 -0.0859323773480562

22 36 -0.0494330552226605

22 37 0.0731834487524842

22 38 0.142347163683406

22 39 0.119748450066111

22 40 0.0809222486951082

22 41 0.0958842204578312

22 42 -0.0458601251193630

22 43 0.0917631615261095

22 44 0.136147025219801

22 45 0.127845960861509

22 46 0.0732542600533559

22 47 0.0891175747358189

22 48 0.0805631364167909

22 49 0.192837192033124

22 50 0.318786937504185

22 51 0.485271768198624

22 52 0.646259454793745

22 53 0.728351736212643

22 54 0.736956092030741

22 55 0.673336995688111

22 56 0.497893244858710

22 57 0.349119794470117

22 58 0.399945699263533

22 59 0.247727899393994

22 60 0.103011164427958

22 61 0.0405910436273335

22 62 -0.0935033241661838

22 63 -0.167914920996779

22 64 -0.287183228154176

22 65 -0.176600144354669

22 66 -0.165404556179174

22 67 -0.0345537698097479

22 68 0.0396502374125482

22 69 0.130864281675678

22 70 0.0332888446318207

22 71 0.0772948310224640

22 72 -0.388752129235186

22 73 -0.270631774496430

22 74 -0.386813287590730

22 75 -0.287807924164011

22 76 -0.300147141735252

23 1 -0.247017465782482

23 2 -0.336236578402397

23 3 -0.279677600418929

23 4 -0.245238349032680

23 5 -0.0444064902543858

23 6 0.0843929322776651

23 7 0.0212948704036284

23 8 0.0920940994319766

23 9 0.0587283074048232

23 10 0.0368140273063111

23 11 -0.0357695884438525

23 12 -0.121079381080112

23 13 -0.122509562861239

23 14 -0.258425393158704

23 15 -0.140335797192287

23 16 -0.154997427458648

23 17 -0.00792363230208744

23 18 0.00993882337165615

23 19 0.0588310352717697

23 20 0.246033448247306

23 21 0.471755258974544

23 22 0.801404658984911

23 23 0.999999999999995

23 24 0.852672408682209

23 25 0.730512393150999

23 26 0.684205542789733

23 27 0.635909021831687

23 28 0.540606309218648

23 29 0.370731905143358

23 30 0.245415958142413

23 31 0.224161504396851

23 32 0.146671183128171

23 33 0.0319359619303885

23 34 -0.0195679449081069

23 35 0.0415945128475936

23 36 0.0800318708935129

23 37 0.230478107201674

23 38 0.330748243276479

23 39 0.257901848553980

23 40 0.195836746236499

23 41 0.239562326636992

23 42 0.0684263106562496

23 43 0.238311400135789

23 44 0.275124300472577

23 45 0.252559378902303

23 46 0.165640040846088

23 47 0.182337008185451

23 48 0.199235669875434

23 49 0.342002200542565

23 50 0.470046316745618

23 51 0.584268196758109

23 52 0.743748052443339

23 53 0.718885152347385

23 54 0.650709853927539

23 55 0.499422562128675

23 56 0.333395604972596

23 57 0.134612870281996

23 58 0.234805552535994

23 59 0.153696281993018

23 60 -0.0338331209679561

23 61 -0.0607846085646331

23 62 -0.179800499809903

23 63 -0.291276013283811

23 64 -0.355962821553829

23 65 -0.217718729703193

23 66 -0.0995671943756740

23 67 0.0655926779024701

23 68 0.185947304339208

23 69 0.219561303211018

23 70 0.0696750916329347

23 71 -0.0107054369609165

23 72 -0.476555420032722

23 73 -0.454861492335585

23 74 -0.547075675655626

23 75 -0.431303455238524

23 76 -0.406878607918758

24 1 -0.294962427606561

24 2 -0.351954137983526

24 3 -0.264493373018982

24 4 -0.175648077169164

24 5 0.0530818877862531

24 6 0.174290473374360

24 7 0.104969048737220

24 8 0.133446606384875

24 9 0.121748517774907

24 10 0.0826294786096462

24 11 0.0747511182212782

24 12 -0.0168043755803985

24 13 -0.0135352296765905

24 14 -0.164829157038599

24 15 -0.0643172971584770

24 16 -0.143087500813241

24 17 -0.0201622541352432

24 18 -0.0603427572073602

24 19 -0.0545968218986693

24 20 0.114264409145372

24 21 0.348198006624852

24 22 0.678361331520610

24 23 0.852672408682209

24 24 1.00000000000000

24 25 0.838456025504351

24 26 0.704572387567289

24 27 0.730011138549028

24 28 0.671878487301596

24 29 0.482888155807027

24 30 0.355318622220692

24 31 0.325536150665432

24 32 0.250871852318620

24 33 0.142158651668715

24 34 0.0731131112983565

24 35 0.117151062755226

24 36 0.136232664153432

24 37 0.238729897914150

24 38 0.338273973933676

24 39 0.245075192755908

24 40 0.193960821436575

24 41 0.284464306960744

24 42 0.104200171906897

24 43 0.261013212505045

24 44 0.297756012051326

24 45 0.274396118669478

24 46 0.191319803600412

24 47 0.197744202732692

24 48 0.228531375013350

24 49 0.334784821484472

24 50 0.433173259992748

24 51 0.547064166415950

24 52 0.718570228529600

24 53 0.687192034125363

24 54 0.545173682857018

24 55 0.371233979238221

24 56 0.175552626901817

24 57 -0.0370697406636623

24 58 0.0788570290366298

24 59 0.0202014969704312

24 60 -0.181836959584122

24 61 -0.178282044577170

24 62 -0.250202282469765

24 63 -0.352353178086665

24 64 -0.385929364913907

24 65 -0.234373066798925

24 66 -0.0590774857483694

24 67 0.111116995975054

24 68 0.254411516635297

24 69 0.249374863739258

24 70 0.0691057547704728

24 71 -0.0867318659306034

24 72 -0.453637881449400

24 73 -0.493977167753139

24 74 -0.602635886131525

24 75 -0.462939550530311

24 76 -0.422724875212364

25 1 -0.191015904326312

25 2 -0.273012266020904

25 3 -0.253256409020796

25 4 -0.199094186707750

25 5 -0.00116780884348275

25 6 0.102417811702511

25 7 0.0485505480243076

25 8 0.0725375174861271

25 9 0.0712023824632018

25 10 0.0484870811300269

25 11 0.0466150405479170

25 12 -0.0307098786660949

25 13 -0.0353407414951204

25 14 -0.163032499077102

25 15 -0.0701068661693916

25 16 -0.0847072003528855

25 17 0.0205586460313993

25 18 0.0251975646163091

25 19 -0.00677579586532289

25 20 0.137357648808928

25 21 0.372560822484044

25 22 0.659160342406123

25 23 0.730512393150999

25 24 0.838456025504351

25 25 0.999999999999998

25 26 0.817617044196740

25 27 0.674278119954920

25 28 0.670021592342622

25 29 0.540048859575697

25 30 0.350182799443863

25 31 0.277646038183258

25 32 0.227672662650538

25 33 0.125252399038039

25 34 0.0403331483977892

25 35 0.0722264678068311

25 36 0.0831875560377190

25 37 0.169365801976414

25 38 0.259066364737345

25 39 0.187348368737778

25 40 0.141498517227567

25 41 0.225572763562594

25 42 0.0773498220121048

25 43 0.204139142505856

25 44 0.201714001255952

25 45 0.170085835613529

25 46 0.0997541473179889

25 47 0.104303680590819

25 48 0.131710046259046

25 49 0.219636857886538

25 50 0.299950204839159

25 51 0.398142114460690

25 52 0.566493849496130

25 53 0.540004325971773

25 54 0.410953695521712

25 55 0.298229339638018

25 56 0.142946386804073

25 57 -0.0390844723888981

25 58 0.0258233327584440

25 59 -0.0322449959108062

25 60 -0.183485563964521

25 61 -0.173392478411657

25 62 -0.228799909327696

25 63 -0.293635605840543

25 64 -0.335896967792202

25 65 -0.246915762370679

25 66 -0.102302265575810

25 67 0.0400218596615111

25 68 0.161442712520656

25 69 0.187804588772713

25 70 0.0599717895949661

25 71 -0.0691924464898569

25 72 -0.347825998445293

25 73 -0.367531349499776

25 74 -0.475637731707361

25 75 -0.350371299272865

25 76 -0.313613414235443

26 1 -0.275463760274303

26 2 -0.312708500539056

26 3 -0.264390562924653

26 4 -0.189459381834294

26 5 0.0259265784238789

26 6 0.111207829615984

26 7 0.0477864710149721

26 8 0.0552509666301330

26 9 0.0513170367199328

26 10 0.0170021414274479

26 11 0.0393526860270639

26 12 -0.0349383358999136

26 13 -0.0271499303512438

26 14 -0.166944664114472

26 15 -0.0861935900655475

26 16 -0.161917380682427

26 17 -0.102498147122041

26 18 -0.113411430105125

26 19 -0.115968167389730

26 20 0.0131834134699772

26 21 0.219938106859422

26 22 0.538269650538512

26 23 0.684205542789733

26 24 0.704572387567289

26 25 0.817617044196740

26 26 1.00000000000000

26 27 0.791661732934236

26 28 0.691431050375375

26 29 0.649424709193852

26 30 0.505581862291088

26 31 0.379768812605457

26 32 0.331405467848894

26 33 0.224076653842265

26 34 0.128263131445840

26 35 0.144448413159761

26 36 0.146098990903788

26 37 0.225053313870709

26 38 0.325696251308153

26 39 0.233605094512994

26 40 0.175244151086455

26 41 0.263438750305722

26 42 0.109945092618937

26 43 0.246485162212957

26 44 0.242279664203016

26 45 0.206100719370421

26 46 0.121614232626319

26 47 0.126983139731755

26 48 0.153382922456228

26 49 0.248011297230893

26 50 0.303420758190199

26 51 0.353571028126993

26 52 0.503978309369873

26 53 0.461178620814244

26 54 0.329591281812336

26 55 0.187710074769586

26 56 0.0652817915371818

26 57 -0.0946431249037705

26 58 -0.0136252738269834

26 59 -0.0400810679462125

26 60 -0.179746000901200

26 61 -0.177286137732194

26 62 -0.245333781370702

26 63 -0.337022449243922

26 64 -0.336163374252965

26 65 -0.241297859279174

26 66 -0.0723716672955232

26 67 0.0730451972213870

26 68 0.199984069225918

26 69 0.199988801939665

26 70 0.0553555071972352

26 71 -0.0876190285806787

26 72 -0.363035432648226

26 73 -0.397991705666661

26 74 -0.462706544043890

26 75 -0.365704900601254

26 76 -0.322008803657194

27 1 -0.381846437186712

27 2 -0.367001708701519

27 3 -0.252136005465287

27 4 -0.113363172223059

27 5 0.149428095773244

27 6 0.231769059678190

27 7 0.154993768295841

27 8 0.126290624942890

27 9 0.130335130782352

27 10 0.0601433622061883

27 11 0.136347458058645

27 12 0.0437065433275091

27 13 0.0729052293278508

27 14 -0.0933813115337207

27 15 -0.0359847299068221

27 16 -0.191060721659167

27 17 -0.147676675795573

27 18 -0.220841100716195

27 19 -0.221574254218629

27 20 -0.0851984477585932

27 21 0.123499433201245

27 22 0.406078208090688

27 23 0.635909021831687

27 24 0.730011138549028

27 25 0.674278119954920

27 26 0.791661732934236

27 27 0.999999999999992

27 28 0.861556506989892

27 29 0.662479681002366

27 30 0.631097825168951

27 31 0.602360975270672

27 32 0.468091469810707

27 33 0.321433450752903

27 34 0.279347248922872

27 35 0.332096284135090

27 36 0.356368372208446

27 37 0.440440239109640

27 38 0.545287291780526

27 39 0.420666032885635

27 40 0.333060132647154

27 41 0.420328363384996

27 42 0.206894788928977

27 43 0.320652534097423

27 44 0.334930943542038

27 45 0.291743394512984

27 46 0.212251236905449

27 47 0.220945727140230

27 48 0.237574721622034

27 49 0.316798612934825

27 50 0.349796577148796

27 51 0.379197982609277

27 52 0.515217445409856

27 53 0.463217174233836

27 54 0.303061142560720

27 55 0.127067438312924

27 56 -0.0218628651822969

27 57 -0.199056109441328

27 58 -0.0863439000446589

27 59 -0.0927869496496677

27 60 -0.265622479091877

27 61 -0.255689287364284

27 62 -0.307611593744518

27 63 -0.414478151019219

27 64 -0.381822244099503

27 65 -0.245332479740221

27 66 -0.00261561683630440

27 67 0.175336835382723

27 68 0.344655501289415

27 69 0.276944719576107

27 70 0.0746531612102820

27 71 -0.144061594832386

27 72 -0.423228852898357

27 73 -0.566003427637744

27 74 -0.638276606218651

27 75 -0.515401921429093

27 76 -0.444717428531649

28 1 -0.373756596521663

28 2 -0.356660358695621

28 3 -0.247508680844451

28 4 -0.102797995272874

28 5 0.161410143698846

28 6 0.227057595173839

28 7 0.153253811920676

28 8 0.104332193523483

28 9 0.126610798192509

28 10 0.0492289378827158

28 11 0.165258930139613

28 12 0.0785223328011769

28 13 0.119527953901730

28 14 -0.0662048939056276

28 15 -0.0337864430593122

28 16 -0.195316079209083

28 17 -0.165674033530541

28 18 -0.235955632853906

28 19 -0.237531157417950

28 20 -0.126781337401428

28 21 0.0724082636711057

28 22 0.337566953043307

28 23 0.540606309218648

28 24 0.671878487301596

28 25 0.670021592342622

28 26 0.691431050375375

28 27 0.861556506989892

28 28 0.999999999999997

28 29 0.787151948192235

28 30 0.663887053411707

28 31 0.682808159671696

28 32 0.592055972100126

28 33 0.419732099771511

28 34 0.348178802617623

28 35 0.411141208746969

28 36 0.395123987039932

28 37 0.437501034780114

28 38 0.497527607648559

28 39 0.373517846383783

28 40 0.313575324952458

28 41 0.394325161163040

28 42 0.190269725441674

28 43 0.290000437765707

28 44 0.299284947524514

28 45 0.258390212254983

28 46 0.192702931601302

28 47 0.195855491622012

28 48 0.219492988820487

28 49 0.284515685222322

28 50 0.306584799864000

28 51 0.338414841524761

28 52 0.451988881731006

28 53 0.403383858678547

28 54 0.243996444834219

28 55 0.0811850738267566

28 56 -0.0582483570639615

28 57 -0.227057944356788

28 58 -0.115909780889583

28 59 -0.111253615546749

28 60 -0.278708018489905

28 61 -0.265858846003129

28 62 -0.313785793846355

28 63 -0.407649516463129

28 64 -0.376786633562594

28 65 -0.254582239464280

28 66 -0.00340261183588792

28 67 0.167065356522748

28 68 0.327568212226708

28 69 0.248345595756556

28 70 0.0696000469566635

28 71 -0.168872645964311

28 72 -0.393473869197198

28 73 -0.536233092785483

28 74 -0.615892989407330

28 75 -0.481422302052785

28 76 -0.400227186159009

29 1 -0.398015080937856

29 2 -0.370127276762385

29 3 -0.279252076993169

29 4 -0.140159331951942

29 5 0.112298476616554

29 6 0.161979704930306

29 7 0.0767813854370499

29 8 0.0212640522523256

29 9 0.0553047112204582

29 10 0.00449493010196424

29 11 0.130164009121799

29 12 0.0704469866782385

29 13 0.0811688019980081

29 14 -0.108728571938027

29 15 -0.131080740670204

29 16 -0.288044794949019

29 17 -0.289317088697746

29 18 -0.338397477791136

29 19 -0.308560388833218

29 20 -0.229178936945467

29 21 -0.0817619223315707

29 22 0.206335740250741

29 23 0.370731905143358

29 24 0.482888155807027

29 25 0.540048859575697

29 26 0.649424709193852

29 27 0.662479681002366

29 28 0.787151948192235

29 29 0.999999999999998

29 30 0.830378711177107

29 31 0.674847309986613

29 32 0.730023320618530

29 33 0.644894373422217

29 34 0.482475314685686

29 35 0.450278861247825

29 36 0.354702504327867

29 37 0.271546500740973

29 38 0.282798085804462

29 39 0.143575528646912

29 40 0.161181922569382

29 41 0.293777019326644

29 42 0.137572028348574

29 43 0.237967987142176

29 44 0.230166288518548

29 45 0.191586232635324

29 46 0.124222681568798

29 47 0.116694650463598

29 48 0.156825770862357

29 49 0.228487965043374

29 50 0.238915940005258

29 51 0.273279590658531

29 52 0.348146687667263

29 53 0.286031548029565

29 54 0.141750907710745

29 55 -0.0225100796050307

29 56 -0.132740823854637

29 57 -0.257210274067920

29 58 -0.134756737029293

29 59 -0.109288406462961

29 60 -0.255136274796739

29 61 -0.256971219207662

29 62 -0.304553966991581

29 63 -0.406146724695539

29 64 -0.350920887113012

29 65 -0.240504744507453

29 66 -0.0134305073300547

29 67 0.125751232547976

29 68 0.250570118423323

29 69 0.194998716756830

29 70 0.0609901270767567

29 71 -0.160595435162264

29 72 -0.298530660078405

29 73 -0.379356357694027

29 74 -0.432948533262929

29 75 -0.330909105360602

29 76 -0.266774570614938

30 1 -0.447424757287630

30 2 -0.389275012683159

30 3 -0.267761131639392

30 4 -0.107785239849451

30 5 0.137815389482785

30 6 0.153159425394540

30 7 0.0536766842201580

30 8 -0.0242194293114669

30 9 0.0227274996819244

30 10 -0.0234326702958154

30 11 0.135068468629995

30 12 0.0891332541247470

30 13 0.110299544777560

30 14 -0.0702630326151692

30 15 -0.127575500255701

30 16 -0.328698697005226

30 17 -0.355303696219890

30 18 -0.413723270473641

30 19 -0.373417405850607

30 20 -0.299662255216395

30 21 -0.178696004356491

30 22 0.0688351552931894

30 23 0.245415958142413

30 24 0.355318622220692

30 25 0.350182799443863

30 26 0.505581862291088

30 27 0.631097825168951

30 28 0.663887053411707

30 29 0.830378711177107

30 30 0.999999999999997

30 31 0.844088671636666

30 32 0.794299237688868

30 33 0.750403015108095

30 34 0.693267534672459

30 35 0.646636080582981

30 36 0.584761929371183

30 37 0.442066858981826

30 38 0.390777544114068

30 39 0.258442725825243

30 40 0.302518637324405

30 41 0.367394145013815

30 42 0.154027347123146

30 43 0.236197385511865

30 44 0.250478064047892

30 45 0.221038371846411

30 46 0.168927702030811

30 47 0.171824560613835

30 48 0.196981858481256

30 49 0.247650393697616

30 50 0.217554636281735

30 51 0.215785783758508

30 52 0.273125104845380

30 53 0.207732732942776

30 54 0.0656776514749720

30 55 -0.103806637042299

30 56 -0.203407682711983

30 57 -0.296939526850533

30 58 -0.167465340726109

30 59 -0.124907538768950

30 60 -0.265418347992999

30 61 -0.270113982984004

30 62 -0.307713731155243

30 63 -0.418080409153968

30 64 -0.346173326764697

30 65 -0.233942614164689

30 66 0.00894549063923464

30 67 0.151699229131910

30 68 0.273856649102763

30 69 0.193602883031852

30 70 0.0634728806263709

30 71 -0.146686207055315

30 72 -0.268959990792777

30 73 -0.424432884423213

30 74 -0.465065792235904

30 75 -0.380959537259701

30 76 -0.299485036975049

31 1 -0.429894742443516

31 2 -0.374233980955825

31 3 -0.236269127676237

31 4 -0.0795799204981665

31 5 0.150469307005218

31 6 0.158083001545737

31 7 0.0644345282838046

31 8 -0.0145643686646280

31 9 0.0342728177182626

31 10 -0.0320415714665830

31 11 0.141501747831080

31 12 0.0902099114084912

31 13 0.140622577783771

31 14 -0.0446655789349506

31 15 -0.0991949471360018

31 16 -0.307141226093305

31 17 -0.322608904339820

31 18 -0.381523969150136

31 19 -0.334936503050867

31 20 -0.269885731690680

31 21 -0.165727060123104

31 22 0.0358201640306269

31 23 0.224161504396851

31 24 0.325536150665432

31 25 0.277646038183258

31 26 0.379768812605457

31 27 0.602360975270672

31 28 0.682808159671696

31 29 0.674847309986613

31 30 0.844088671636666

31 31 1.00000000000000

31 32 0.853822296018795

31 33 0.708786594992642

31 34 0.734338472044115

31 35 0.783417271361316

31 36 0.739048816441011

31 37 0.646990507893161

31 38 0.560644430423741

31 39 0.438391001699954

31 40 0.437352666436554

31 41 0.397164013538332

31 42 0.151492936735487

31 43 0.220671921736873

31 44 0.268111701998746

31 45 0.252196858907791

31 46 0.215918268922189

31 47 0.226677090533988

31 48 0.230558716122665

31 49 0.259108452021594

31 50 0.215696068340618

31 51 0.203571156184957

31 52 0.256819988350278

31 53 0.202120248482667

31 54 0.0746484761750143

31 55 -0.0798581654473464

31 56 -0.172412261617457

31 57 -0.260757594233014

31 58 -0.135795251525050

31 59 -0.0927758854818175

31 60 -0.236569711964026

31 61 -0.241236465982160

31 62 -0.278545923045734

31 63 -0.384410780825311

31 64 -0.331155551068222

31 65 -0.223261671798581

31 66 0.0304427903914681

31 67 0.172355996244615

31 68 0.292325927303398

31 69 0.190590205005083

31 70 0.0627958287556572

31 71 -0.146668771251618

31 72 -0.306687345708735

31 73 -0.507015836710536

31 74 -0.549924896331721

31 75 -0.449255227427557

31 76 -0.349375612394482

32 1 -0.425689081683833

32 2 -0.375358036172988

32 3 -0.253603098125204

32 4 -0.106812799066177

32 5 0.115744259155435

32 6 0.122418188931772

32 7 0.0385677385741031

32 8 -0.0375019042651696

32 9 0.0157375604676797

32 10 -0.0345680732508993

32 11 0.133423895714071

32 12 0.0908697772572996

32 13 0.122087501900423

32 14 -0.0750428461535030

32 15 -0.151849996014795

32 16 -0.343595561132973

32 17 -0.369070959652132

32 18 -0.410878365435310

32 19 -0.347822520099438

32 20 -0.302417150451330

32 21 -0.227202495269460

32 22 -0.0132936229850689

32 23 0.146671183128171

32 24 0.250871852318620

32 25 0.227672662650538

32 26 0.331405467848894

32 27 0.468091469810707

32 28 0.592055972100126

32 29 0.730023320618530

32 30 0.794299237688868

32 31 0.853822296018795

32 32 1.00000000000001

32 33 0.864070209693835

32 34 0.752771044571592

32 35 0.747313735700315

32 36 0.597318879786044

32 37 0.426655167157805

32 38 0.339058935760984

32 39 0.194004483250729

32 40 0.232049500288094

32 41 0.272009131606425

32 42 0.0973845443714618

32 43 0.169841843480612

32 44 0.211057407837940

32 45 0.194009571043440

32 46 0.164691844718728

32 47 0.170182231407929

32 48 0.186909812446692

32 49 0.215578086924068

32 50 0.179029767261787

32 51 0.188586530830110

32 52 0.212908813925337

32 53 0.151303189025129

32 54 0.0395678264831561

32 55 -0.110195037144326

32 56 -0.188862405278907

32 57 -0.258574285679571

32 58 -0.132577342915679

32 59 -0.0859328981734868

32 60 -0.214528658708388

32 61 -0.225368675509274

32 62 -0.264171031485963

32 63 -0.374681229584094

32 64 -0.315882679920005

32 65 -0.212260561190090

32 66 0.0137057493324553

32 67 0.133797305398026

32 68 0.230200147559856

32 69 0.143559050412200

32 70 0.0391970339592866

32 71 -0.159510110941039

32 72 -0.248221116308445

32 73 -0.370806877381930

32 74 -0.406130117571241

32 75 -0.320875121766732

32 76 -0.240957579930860

33 1 -0.393235700788526

33 2 -0.343007738916783

33 3 -0.238990599280122

33 4 -0.105440419247061

33 5 0.0814089002415942

33 6 0.0752075564712551

33 7 -0.0146595731254904

33 8 -0.0876806601737419

33 9 -0.0309458875219354

33 10 -0.0468878736110222

33 11 0.108181788892065

33 12 0.0961240833551550

33 13 0.0980403760411711

33 14 -0.0699746433496547

33 15 -0.169257520577108

33 16 -0.340735113695422

33 17 -0.382133448338976

33 18 -0.417923267198299

33 19 -0.355286707770827

33 20 -0.322818268098051

33 21 -0.273640649862316

33 22 -0.0839935838061430

33 23 0.0319359619303885

33 24 0.142158651668715

33 25 0.125252399038039

33 26 0.224076653842265

33 27 0.321433450752903

33 28 0.419732099771511

33 29 0.644894373422217

33 30 0.750403015108095

33 31 0.708786594992642

33 32 0.864070209693835

33 33 1.00000000000001

33 34 0.862062773923477

33 35 0.727513070484577

33 36 0.566082548477506

33 37 0.273912989540785

33 38 0.146669249686053

33 39 0.0149253068454543

33 40 0.118864476455681

33 41 0.183090860671673

33 42 0.0383430810911380

33 43 0.100299837859189

33 44 0.142475755130853

33 45 0.130198344812923

33 46 0.110627773134894

33 47 0.112170160021960

33 48 0.137036155017400

33 49 0.155464221049474

33 50 0.112963536855859

33 51 0.135214486309198

33 52 0.138860121997463

33 53 0.0762476464260727

33 54 -0.0206962238663561

33 55 -0.157447791923796

33 56 -0.225462481767968

33 57 -0.265528664233838

33 58 -0.147160704398690

33 59 -0.0980123497604654

33 60 -0.202720922538424

33 61 -0.213641866298583

33 62 -0.233853329477765

33 63 -0.335820293443006

33 64 -0.274814310939128

33 65 -0.181660323649639

33 66 0.00805641876225787

33 67 0.0975453665444460

33 68 0.160363745283457

33 69 0.0779416589750166

33 70 0.00515985620736785

33 71 -0.156973712984313

33 72 -0.152414322173148

33 73 -0.248203014171326

33 74 -0.268435468923779

33 75 -0.198347054412937

33 76 -0.136296666863710

34 1 -0.371376436692300

34 2 -0.319224567833957

34 3 -0.216226716077449

34 4 -0.0838331068723361

34 5 0.0774254847512447

34 6 0.0573002287474190

34 7 -0.0295009133833166

34 8 -0.105385676186647

34 9 -0.0428876531915549

34 10 -0.0625169029360133

34 11 0.0974643853860983

34 12 0.0880687998182369

34 13 0.110089893388487

34 14 -0.0419398958183925

34 15 -0.148891109158852

34 16 -0.319819497566268

34 17 -0.368021239200574

34 18 -0.402626544367947

34 19 -0.339807355594865

34 20 -0.313404972600679

34 21 -0.278469251152545

34 22 -0.132418175283688

34 23 -0.0195679449081069

34 24 0.0731131112983565

34 25 0.0403331483977892

34 26 0.128263131445840

34 27 0.279347248922872

34 28 0.348178802617623

34 29 0.482475314685686

34 30 0.693267534672459

34 31 0.734338472044115

34 32 0.752771044571592

34 33 0.862062773923477

34 34 1.00000000000000

34 35 0.890995189006268

34 36 0.775090889196928

34 37 0.472212821631695

34 38 0.285697045933451

34 39 0.183292545866649

34 40 0.287454313245692

34 41 0.253488583102562

34 42 0.0506834004484274

34 43 0.0876150623218382

34 44 0.140590209282563

34 45 0.141971711885764

34 46 0.134609502349536

34 47 0.139021433966244

34 48 0.151535867140108

34 49 0.156830938243977

34 50 0.0918325658175992

34 51 0.0867499131122163

34 52 0.0939486967536529

34 53 0.0330327016292039

34 54 -0.0542851112683376

34 55 -0.175114279297449

34 56 -0.231015000947799

34 57 -0.254181218881941

34 58 -0.145850026858779

34 59 -0.0914166512160889

34 60 -0.185769118408748

34 61 -0.193913650640666

34 62 -0.210081334437904

34 63 -0.303023221673046

34 64 -0.253054862885615

34 65 -0.177218302802421

34 66 0.0218485409762073

34 67 0.107858475547527

34 68 0.166267150945987

34 69 0.0693125926853708

34 70 0.0129856597364904

34 71 -0.126483100336880

34 72 -0.136430476429279

34 73 -0.299211649686437

34 74 -0.315163862918776

34 75 -0.251120385044360

34 76 -0.171206107471085

35 1 -0.375175528971758

35 2 -0.328791847857410

35 3 -0.217882434312016

35 4 -0.0864318816754557

35 5 0.0869295533856632

35 6 0.0617003675624616

35 7 -0.0257541348870594

35 8 -0.101314158526662

35 9 -0.0412648472264909

35 10 -0.0794779733003334

35 11 0.0889606259187863

35 12 0.0773378254838564

35 13 0.119999841679231

35 14 -0.0396348281416773

35 15 -0.128205391913821

35 16 -0.307644948118979

35 17 -0.340216629595950

35 18 -0.378190823452128

35 19 -0.312970752382445

35 20 -0.277451530931778

35 21 -0.228843231894362

35 22 -0.0859323773480562

35 23 0.0415945128475936

35 24 0.117151062755226

35 25 0.0722264678068311

35 26 0.144448413159761

35 27 0.332096284135090

35 28 0.411141208746969

35 29 0.450278861247825

35 30 0.646636080582981

35 31 0.783417271361316

35 32 0.747313735700315

35 33 0.727513070484577

35 34 0.890995189006268

35 35 1.00000000000000

35 36 0.888879280150827

35 37 0.650967662968771

35 38 0.450781088559462

35 39 0.343893100742006

35 40 0.436274137222926

35 41 0.350715305813227

35 42 0.102430776032575

35 43 0.138017676364870

35 44 0.189770869992683

35 45 0.184653752834959

35 46 0.175182640057593

35 47 0.192552461042156

35 48 0.200830778498876

35 49 0.214722268388949

35 50 0.146718945603603

35 51 0.133374624161164

35 52 0.155694298737312

35 53 0.0895058698640992

35 54 -0.00336488274169568

35 55 -0.127422800067267

35 56 -0.186802459125537

35 57 -0.229275345555709

35 58 -0.117353820209460

35 59 -0.0626726752595107

35 60 -0.179743355121872

35 61 -0.186008606622787

35 62 -0.221087230948412

35 63 -0.315615238610815

35 64 -0.274711003616016

35 65 -0.203178257162292

35 66 0.0150841831224817

35 67 0.127813601813422

35 68 0.204718570164481

35 69 0.142214892792974

35 70 0.0874532286447295

35 71 -0.0511348332987561

35 72 -0.172985308236961

35 73 -0.373777847780611

35 74 -0.418319608421383

35 75 -0.346792303137252

35 76 -0.260452289108000

36 1 -0.360019011139020

36 2 -0.318569254200542

36 3 -0.207262148766091

36 4 -0.0871012710543748

36 5 0.0754968753917542

36 6 0.0491055848021803

36 7 -0.0309678544220381

36 8 -0.0944234624618153

36 9 -0.0511569721082901

36 10 -0.100108843783055

36 11 0.0466202455972743

36 12 0.0390834809335888

36 13 0.0932795178312974

36 14 -0.0453135075026612

36 15 -0.108117670453807

36 16 -0.279364359346101

36 17 -0.299848104546783

36 18 -0.340137328906767

36 19 -0.285608674595554

36 20 -0.240320948078402

36 21 -0.173554329364481

36 22 -0.0494330552226605

36 23 0.0800318708935129

36 24 0.136232664153432

36 25 0.0831875560377190

36 26 0.146098990903788

36 27 0.356368372208446

36 28 0.395123987039932

36 29 0.354702504327867

36 30 0.584761929371183

36 31 0.739048816441011

36 32 0.597318879786044

36 33 0.566082548477506

36 34 0.775090889196928

36 35 0.888879280150827

36 36 0.999999999999999

36 37 0.807554325391650

36 38 0.585004193526927

36 39 0.476495878690431

36 40 0.568375482275565

36 41 0.433211871288981

36 42 0.148355260739698

36 43 0.180819667205133

36 44 0.228747527085798

36 45 0.221867986912651

36 46 0.201300937853471

36 47 0.222824596047032

36 48 0.230837971272422

36 49 0.253469721745123

36 50 0.183849071395049

36 51 0.151090716894076

36 52 0.177555402927229

36 53 0.117148387802487

36 54 0.0252751621512333

36 55 -0.0953301103457450

36 56 -0.158285841401695

36 57 -0.209736959472802

36 58 -0.100908550079968

36 59 -0.0443813748834433

36 60 -0.167706080358953

36 61 -0.173654524340932

36 62 -0.215816079659353

36 63 -0.304541421727446

36 64 -0.270516749592933

36 65 -0.197169012176684

36 66 0.0177103036652216

36 67 0.140534035133030

36 68 0.227834419687160

36 69 0.165132396148443

36 70 0.120216377148698

36 71 -0.00979275331132751

36 72 -0.195381940839282

36 73 -0.423693043705553

36 74 -0.476357297692809

36 75 -0.405990605064888

36 76 -0.318613490924674

37 1 -0.320651658225510

37 2 -0.294619878606963

37 3 -0.178908736121468

37 4 -0.0713762889548722

37 5 0.0910462668736993

37 6 0.113027277636953

37 7 0.0392793430807221

37 8 0.00882238202160917

37 9 0.0252984111826687

37 10 -0.0426835403645432

37 11 0.0521359689975567

37 12 0.0120300723412113

37 13 0.0651384361880125

37 14 -0.0701069474123406

37 15 -0.0788637010171298

37 16 -0.228068209198210

37 17 -0.214957029627248

37 18 -0.250407232395354

37 19 -0.199587007015105

37 20 -0.134789033486879

37 21 -0.0529340656993631

37 22 0.0731834487524842

37 23 0.230478107201674

37 24 0.238729897914150

37 25 0.169365801976414

37 26 0.225053313870709

37 27 0.440440239109640

37 28 0.437501034780114

37 29 0.271546500740973

37 30 0.442066858981826

37 31 0.646990507893161

37 32 0.426655167157805

37 33 0.273912989540785

37 34 0.472212821631695

37 35 0.650967662968771

37 36 0.807554325391650

37 37 0.999999999999995

37 38 0.830252637065095

37 39 0.699038313667531

37 40 0.674644249638085

37 41 0.467448242943712

37 42 0.190005796381016

37 43 0.223219524364025

37 44 0.282654640241989

37 45 0.278885809682739

37 46 0.244898132970053

37 47 0.262495784818356

37 48 0.242838701276551

37 49 0.278522018428579

37 50 0.235728478704629

37 51 0.197156680239684

37 52 0.242773170880872

37 53 0.211228093115801

37 54 0.132673259484736

37 55 0.0216137713318580

37 56 -0.0464134971634152

37 57 -0.119212749727555

37 58 -0.0158563445630664

37 59 0.0185133117225431

37 60 -0.114234054029289

37 61 -0.126193298066723

37 62 -0.184956954471162

37 63 -0.270727420637224

37 64 -0.258616608916818

37 65 -0.170634798565934

37 66 0.0362023523930931

37 67 0.167081386384830

37 68 0.281297533610163

37 69 0.209402780289304

37 70 0.112794456996240

37 71 -0.0131421188611691

37 72 -0.292046433719086

37 73 -0.525102823455134

37 74 -0.564979601408302

37 75 -0.479460917174077

37 76 -0.388249759440332

38 1 -0.311272524053436

38 2 -0.282201168745666

38 3 -0.177367726560218

38 4 -0.0623160967263961

38 5 0.122551834848013

38 6 0.199936500588730

38 7 0.176631066026327

38 8 0.159172897291155

38 9 0.147823463031005

38 10 0.0479812869899542

38 11 0.0989738856931152

38 12 0.00808448139219226

38 13 0.0552034604893422

38 14 -0.0775857864021485

38 15 -0.0551956637825089

38 16 -0.192765048617536

38 17 -0.176770545042085

38 18 -0.220523542764027

38 19 -0.185559542949001

38 20 -0.107087332302609

38 21 -0.00260024197284340

38 22 0.142347163683406

38 23 0.330748243276479

38 24 0.338273973933676

38 25 0.259066364737345

38 26 0.325696251308153

38 27 0.545287291780526

38 28 0.497527607648559

38 29 0.282798085804462

38 30 0.390777544114068

38 31 0.560644430423741

38 32 0.339058935760984

38 33 0.146669249686053

38 34 0.285697045933451

38 35 0.450781088559462

38 36 0.585004193526927

38 37 0.830252637065095

38 38 1.00000000000000

38 39 0.829677314208949

38 40 0.624049613769260

38 41 0.525687562647960

38 42 0.315340040938639

38 43 0.306853764175964

38 44 0.322059658241218

38 45 0.281301323028899

38 46 0.231695518735598

38 47 0.256182491179194

38 48 0.226578419539726

38 49 0.268042899433471

38 50 0.251631889878619

38 51 0.200182734784397

38 52 0.257721983540373

38 53 0.245172117128037

38 54 0.163979888485314

38 55 0.0478631096783052

38 56 -0.0328039077245513

38 57 -0.114700941414388

38 58 -0.0245707519217449

38 59 -0.0117050407643463

38 60 -0.127603363816872

38 61 -0.144643668207900

38 62 -0.199154842331156

38 63 -0.281657431956191

38 64 -0.264465043545828

38 65 -0.164330016526923

38 66 0.0330416748210204

38 67 0.166155107721515

38 68 0.333013746552119

38 69 0.261846167693757

38 70 0.121037183242287

38 71 -0.0530553046782482

38 72 -0.336010189641966

38 73 -0.582119632072821

38 74 -0.608706064337309

38 75 -0.516901230436706

38 76 -0.437782884635517

39 1 -0.208117387639577

39 2 -0.187254357838571

39 3 -0.113169892039849

39 4 -0.0301718031402131

39 5 0.0920139082111529

39 6 0.166953589293290

39 7 0.180757746717007

39 8 0.175493127640315

39 9 0.153275550023854

39 10 0.0511739315044925

39 11 0.0714293264423932

39 12 -0.0163729046068477

39 13 0.0315132466155207

39 14 -0.0501438639582860

39 15 -0.0167383728326960

39 16 -0.114799881009829

39 17 -0.0920334130131226

39 18 -0.121456312253284

39 19 -0.102801993525018

39 20 -0.0278212070303214

39 21 0.0497887796921828

39 22 0.119748450066111

39 23 0.257901848553980

39 24 0.245075192755908

39 25 0.187348368737778

39 26 0.233605094512994

39 27 0.420666032885635

39 28 0.373517846383783

39 29 0.143575528646912

39 30 0.258442725825243

39 31 0.438391001699954

39 32 0.194004483250729

39 33 0.0149253068454543

39 34 0.183292545866649

39 35 0.343893100742006

39 36 0.476495878690431

39 37 0.699038313667531

39 38 0.829677314208949

39 39 1.00000000000000

39 40 0.742809063941691

39 41 0.525921350270985

39 42 0.324024675117597

39 43 0.264793816421933

39 44 0.260502918965852

39 45 0.226435004999466

39 46 0.197907637625541

39 47 0.217330778094621

39 48 0.155715697655483

39 49 0.176150203750048

39 50 0.156839060721653

39 51 0.0952650460374991

39 52 0.156344407595124

39 53 0.181474206912657

39 54 0.126889244745911

39 55 0.0560998628358327

39 56 -0.000644205651995644

39 57 -0.0524973344918280

39 58 0.00315100215686104

39 59 0.00681204362551347

39 60 -0.0710960516226592

39 61 -0.0914869605376868

39 62 -0.133957849324211

39 63 -0.188926358635535

39 64 -0.185261107901256

39 65 -0.121087121679245

39 66 0.0267432263477542

39 67 0.127949584793789

39 68 0.287220918288495

39 69 0.221896654176097

39 70 0.111945031833912

39 71 0.0170784948921016

39 72 -0.252693520177268

39 73 -0.525396654459772

39 74 -0.544202094200032

39 75 -0.470031403635882

39 76 -0.401923096303141

40 1 -0.259978060430438

40 2 -0.243264611639638

40 3 -0.155615652046401

40 4 -0.0674169870639001

40 5 0.0626570309130611

40 6 0.0946805506009658

40 7 0.0282089075191866

40 8 0.00832973827440960

40 9 0.00809183010486669

40 10 -0.0260506246995999

40 11 0.0166845996858476

40 12 -0.00144983980482993

40 13 0.00937244833172256

40 14 -0.0720485359003425

40 15 -0.0645578777970124

40 16 -0.181728942138213

40 17 -0.168361907921924

40 18 -0.196508960984020

40 19 -0.166784738717257

40 20 -0.111768213068709

40 21 -0.0102283400786511

40 22 0.0809222486951082

40 23 0.195836746236499

40 24 0.193960821436575

40 25 0.141498517227567

40 26 0.175244151086455

40 27 0.333060132647154

40 28 0.313575324952458

40 29 0.161181922569382

40 30 0.302518637324405

40 31 0.437352666436554

40 32 0.232049500288094

40 33 0.118864476455681

40 34 0.287454313245692

40 35 0.436274137222926

40 36 0.568375482275565

40 37 0.674644249638085

40 38 0.624049613769260

40 39 0.742809063941691

40 40 0.999999999999995

40 41 0.699834157759752

40 42 0.394901107789753

40 43 0.328863688939438

40 44 0.293383113605139

40 45 0.242763857185765

40 46 0.197766823444963

40 47 0.230947988054376

40 48 0.219476657780010

40 49 0.263419025414801

40 50 0.234154636105367

40 51 0.177005005891840

40 52 0.210798427960952

40 53 0.178215714353255

40 54 0.115465976312608

40 55 0.0176999661098944

40 56 -0.0417792972226716

40 57 -0.109336340848237

40 58 -0.0219840331592581

40 59 0.0172411308831053

40 60 -0.0922158926645226

40 61 -0.100650650199134

40 62 -0.159965531052400

40 63 -0.228842008536651

40 64 -0.219930184323751

40 65 -0.145896394199504

40 66 0.00459926908987819

40 67 0.135446931835951

40 68 0.251541996944905

40 69 0.286702886640803

40 70 0.293538792040585

40 71 0.248661374188834

40 72 -0.0767149930271502

40 73 -0.416441990082385

40 74 -0.524348329256186

40 75 -0.454413075297964

40 76 -0.399487736062934

41 1 -0.333481407154462

41 2 -0.305478672697405

41 3 -0.213260797882060

41 4 -0.0873623902243927

41 5 0.131282733672467

41 6 0.196683060438676

41 7 0.163146758085739

41 8 0.132040759460338

41 9 0.113875771686096

41 10 0.0614647606680488

41 11 0.0804708741151445

41 12 0.0216481996165506

41 13 0.0178053886828896

41 14 -0.101491325594376

41 15 -0.0912983059749021

41 16 -0.230827698209262

41 17 -0.216764203718179

41 18 -0.260307134895567

41 19 -0.228815532845865

41 20 -0.169158185504835

41 21 -0.0630470860947595

41 22 0.0958842204578312

41 23 0.239562326636992

41 24 0.284464306960744

41 25 0.225572763562594

41 26 0.263438750305722

41 27 0.420328363384996

41 28 0.394325161163040

41 29 0.293777019326644

41 30 0.367394145013815

41 31 0.397164013538332

41 32 0.272009131606425

41 33 0.183090860671673

41 34 0.253488583102562

41 35 0.350715305813227

41 36 0.433211871288981

41 37 0.467448242943712

41 38 0.525687562647960

41 39 0.525921350270985

41 40 0.699834157759752

41 41 1.00000000000000

41 42 0.651395612103793

41 43 0.536079864148489

41 44 0.397297665456251

41 45 0.265233265547079

41 46 0.158196374639991

41 47 0.204289689571830

41 48 0.219433616451332

41 49 0.309714824181340

41 50 0.308136330445591

41 51 0.271209817008286

41 52 0.264519984145851

41 53 0.204688916026123

41 54 0.134167872323175

41 55 0.00124737222319550

41 56 -0.0891462167198205

41 57 -0.177779215742426

41 58 -0.0626651421432072

41 59 -0.0222466189716516

41 60 -0.157268858252425

41 61 -0.169653682199992

41 62 -0.230981004126467

41 63 -0.322998167695646

41 64 -0.290940053125525

41 65 -0.169155420902817

41 66 -0.000192743286376390

41 67 0.184670313529397

41 68 0.370286738349616

41 69 0.507732060677229

41 70 0.479555572372178

41 71 0.241716620887159

41 72 -0.0814743650731793

41 73 -0.408468547761672

41 74 -0.562787681886922

41 75 -0.478009471190971

41 76 -0.459075605451169

42 1 -0.238982475167763

42 2 -0.197719938109935

42 3 -0.158862335503836

42 4 -0.0249155898032938

42 5 0.168980857550469

42 6 0.234881282578235

42 7 0.315350323436901

42 8 0.254511519860383

42 9 0.228211341953098

42 10 0.139565124125904

42 11 0.151092515929084

42 12 0.0581721045930726

42 13 0.0596058079762999

42 14 -0.0275737140289690

42 15 -0.0613508468142954

42 16 -0.166523220657569

42 17 -0.189974053778188

42 18 -0.228526565852430

42 19 -0.233839991068047

42 20 -0.205506525250335

42 21 -0.139406503341548

42 22 -0.0458601251193630

42 23 0.0684263106562496

42 24 0.104200171906897

42 25 0.0773498220121048

42 26 0.109945092618937

42 27 0.206894788928977

42 28 0.190269725441674

42 29 0.137572028348574

42 30 0.154027347123146

42 31 0.151492936735487

42 32 0.0973845443714618

42 33 0.0383430810911380

42 34 0.0506834004484274

42 35 0.102430776032575

42 36 0.148355260739698

42 37 0.190005796381016

42 38 0.315340040938639

42 39 0.324024675117597

42 40 0.394901107789753

42 41 0.651395612103793

42 42 0.999999999999990

42 43 0.694075937798146

42 44 0.402280950830432

42 45 0.154578778219986

42 46 0.0319502435651978

42 47 0.105744086006501

42 48 0.146971482513496

42 49 0.228118732027566

42 50 0.228777023267560

42 51 0.114413041729659

42 52 0.0364620792741350

42 53 0.00683684549753752

42 54 -0.0256296858750736

42 55 -0.123754273856407

42 56 -0.170914069669773

42 57 -0.196045443189848

42 58 -0.144445121556232

42 59 -0.0996888619987405

42 60 -0.127701798202939

42 61 -0.138953793827494

42 62 -0.186590749167887

42 63 -0.248272063643332

42 64 -0.190739692170995

42 65 -0.113770157169213

42 66 -0.000617034352731147

42 67 0.147892861383327

42 68 0.376632943556655

42 69 0.530502497263961

42 70 0.570685550400857

42 71 0.176617742715676

42 72 0.0700579036442376

42 73 -0.275505691574189

42 74 -0.424528682593624

42 75 -0.368040306889995

42 76 -0.394079137566316

43 1 -0.324351958299296

43 2 -0.297992187610777

43 3 -0.210881975902035

43 4 -0.0468510775184187

43 5 0.193526210495431

43 6 0.227162701079510

43 7 0.209801628502481

43 8 0.143402425232402

43 9 0.141881735699209

43 10 0.0732621074614936

43 11 0.133591662520087

43 12 0.0577780064644119

43 13 0.0764135318505758

43 14 -0.0736030459646048

43 15 -0.104541691270775

43 16 -0.229409827642594

43 17 -0.229196698632338

43 18 -0.271549832558913

43 19 -0.260214254902903

43 20 -0.196967754310593

43 21 -0.0871760009332903

43 22 0.0917631615261095

43 23 0.238311400135789

43 24 0.261013212505045

43 25 0.204139142505856

43 26 0.246485162212957

43 27 0.320652534097423

43 28 0.290000437765707

43 29 0.237967987142176

43 30 0.236197385511865

43 31 0.220671921736873

43 32 0.169841843480612

43 33 0.100299837859189

43 34 0.0876150623218382

43 35 0.138017676364870

43 36 0.180819667205133

43 37 0.223219524364025

43 38 0.306853764175964

43 39 0.264793816421933

43 40 0.328863688939438

43 41 0.536079864148489

43 42 0.694075937798146

43 43 1.00000000000001

43 44 0.741732878195900

43 45 0.437403736074775

43 46 0.184288404011943

43 47 0.263947729653912

43 48 0.324958674975204

43 49 0.496690891370101

43 50 0.478803831089908

43 51 0.347482961899283

43 52 0.280153806563272

43 53 0.193706770384449

43 54 0.121815904897006

43 55 -0.0273156736970159

43 56 -0.115034816583208

43 57 -0.205485264894241

43 58 -0.0982496566280113

43 59 -0.0314748619902927

43 60 -0.149298000788379

43 61 -0.148284804594710

43 62 -0.228323150668737

43 63 -0.316725675513029

43 64 -0.248670592313454

43 65 -0.139393481225874

43 66 0.0450817139591625

43 67 0.280834708396338

43 68 0.515666637069568

43 69 0.556567910129294

43 70 0.471980967807838

43 71 0.117774737133830

43 72 -0.161814273709263

43 73 -0.357249600459721

43 74 -0.500628187645780

43 75 -0.424197633417169

43 76 -0.424161510601670

44 1 -0.363668778103790

44 2 -0.349827537451651

44 3 -0.230485326002816

44 4 -0.0681939658954173

44 5 0.167523959190606

44 6 0.202686579733856

44 7 0.150689710646651

44 8 0.104618923478915

44 9 0.0962546479453331

44 10 0.0162263497472655

44 11 0.0761019878835547

44 12 0.000779433529814264

44 13 0.0416868099329541

44 14 -0.137827373912132

44 15 -0.150344506719344

44 16 -0.282058673094612

44 17 -0.253530609940193

44 18 -0.277570827512710

44 19 -0.247584077913076

44 20 -0.165293672923876

44 21 -0.0579074610040779

44 22 0.136147025219801

44 23 0.275124300472577

44 24 0.297756012051326

44 25 0.201714001255952

44 26 0.242279664203016

44 27 0.334930943542038

44 28 0.299284947524514

44 29 0.230166288518548

44 30 0.250478064047892

44 31 0.268111701998746

44 32 0.211057407837940

44 33 0.142475755130853

44 34 0.140590209282563

44 35 0.189770869992683

44 36 0.228747527085798

44 37 0.282654640241989

44 38 0.322059658241218

44 39 0.260502918965852

44 40 0.293383113605139

44 41 0.397297665456251

44 42 0.402280950830432

44 43 0.741732878195900

44 44 0.999999999999994

44 45 0.741806814955840

44 46 0.424239191188244

44 47 0.501664001056702

44 48 0.543857493971171

44 49 0.590291456601465

44 50 0.474491098743508

44 51 0.379527181425727

44 52 0.350433698842628

44 53 0.278916763718784

44 54 0.193348988846565

44 55 0.0248259462688504

44 56 -0.0875139301909092

44 57 -0.182032828393057

44 58 -0.0599179255862576

44 59 -0.0126373048250788

44 60 -0.156887945920053

44 61 -0.152533887029116

44 62 -0.218728016801569

44 63 -0.303999559168597

44 64 -0.250415699143625

44 65 -0.114467065808787

44 66 0.0833895756957986

44 67 0.346302154979126

44 68 0.562464089785479

44 69 0.457323036064344

44 70 0.309611775637534

44 71 0.0243365590518978

44 72 -0.318858143251214

44 73 -0.425258666908881

44 74 -0.504012630686129

44 75 -0.442767361249281

44 76 -0.421842819125224

45 1 -0.339392458626044

45 2 -0.336902421893128

45 3 -0.216364634272688

45 4 -0.0962550119308787

45 5 0.0941522415801021

45 6 0.0929848110534830

45 7 0.0186484980719762

45 8 -0.0109885130435843

45 9 -0.00356419078863286

45 10 -0.0797372212895828

45 11 0.00138398628784897

45 12 -0.0446682571897375

45 13 0.0279400978101689

45 14 -0.151351429400180

45 15 -0.159399685244621

45 16 -0.272712043069888

45 17 -0.244408281672575

45 18 -0.252796644434065

45 19 -0.209947232768689

45 20 -0.139950538534117

45 21 -0.0495056060070213

45 22 0.127845960861509

45 23 0.252559378902303

45 24 0.274396118669478

45 25 0.170085835613529

45 26 0.206100719370421

45 27 0.291743394512984

45 28 0.258390212254983

45 29 0.191586232635324

45 30 0.221038371846411

45 31 0.252196858907791

45 32 0.194009571043440

45 33 0.130198344812923

45 34 0.141971711885764

45 35 0.184653752834959

45 36 0.221867986912651

45 37 0.278885809682739

45 38 0.281301323028899

45 39 0.226435004999466

45 40 0.242763857185765

45 41 0.265233265547079

45 42 0.154578778219986

45 43 0.437403736074775

45 44 0.741806814955840

45 45 0.999999999999986

45 46 0.703832185722403

45 47 0.572570928974721

45 48 0.547611695971287

45 49 0.468968635191495

45 50 0.405900046427514

45 51 0.354214063082111

45 52 0.336366194862857

45 53 0.265386031588244

45 54 0.186336860578730

45 55 0.0426306558511595

45 56 -0.0560328771162474

45 57 -0.164396777356483

45 58 -0.0438850808486811

45 59 0.00529691320046817

45 60 -0.147512033815184

45 61 -0.107141662716885

45 62 -0.126044700642521

45 63 -0.235990612144372

45 64 -0.219917028675633

45 65 -0.0735106931735103

45 66 0.108092851688972

45 67 0.314797677931796

45 68 0.396276240700876

45 69 0.262334971687413

45 70 0.102016777215520

45 71 -0.0449681113037973

45 72 -0.362492187175526

45 73 -0.363713824355253

45 74 -0.391823932284394

45 75 -0.359198913699541

45 76 -0.311696320492405

46 1 -0.241273852211821

46 2 -0.237253882470968

46 3 -0.154877802019344

46 4 -0.0674582042403757

46 5 0.0521769982495882

46 6 0.0416354686692751

46 7 -0.00587568719953713

46 8 -0.0222138881221130

46 9 -0.0122856302804550

46 10 -0.0673524129904643

46 11 -0.000712302471094494

46 12 -0.0359173230453875

46 13 0.0346354074815275

46 14 -0.0893677548305347

46 15 -0.0759320902951405

46 16 -0.167888874799495

46 17 -0.146246425982879

46 18 -0.161310498990145

46 19 -0.138425199603699

46 20 -0.0924616083219983

46 21 -0.0333565450822110

46 22 0.0732542600533559

46 23 0.165640040846088

46 24 0.191319803600412

46 25 0.0997541473179889

46 26 0.121614232626319

46 27 0.212251236905449

46 28 0.192702931601302

46 29 0.124222681568798

46 30 0.168927702030811

46 31 0.215918268922189

46 32 0.164691844718728

46 33 0.110627773134894

46 34 0.134609502349536

46 35 0.175182640057593

46 36 0.201300937853471

46 37 0.244898132970053

46 38 0.231695518735598

46 39 0.197907637625541

46 40 0.197766823444963

46 41 0.158196374639991

46 42 0.0319502435651978

46 43 0.184288404011943

46 44 0.424239191188244

46 45 0.703832185722403

46 46 0.999999999999989

46 47 0.752752771176807

46 48 0.472783965282676

46 49 0.322080515758761

46 50 0.229331207065044

46 51 0.221883331578571

46 52 0.231535869041422

46 53 0.186668741284684

46 54 0.119130805829793

46 55 0.0205322806304670

46 56 -0.0497070272748834

46 57 -0.120433701553034

46 58 -0.0365105799265686

46 59 -0.0141670617915576

46 60 -0.121076295219751

46 61 -0.0992245679190379

46 62 -0.111317045814239

46 63 -0.186327644111396

46 64 -0.192349952177306

46 65 -0.0988379779727188

46 66 -0.00485493832314746

46 67 0.156079090440068

46 68 0.225003688970216

46 69 0.110757510754172

46 70 0.000342334026896806

46 71 -0.0813238174549948

46 72 -0.317094565620223

46 73 -0.299865776182131

46 74 -0.290428220171593

46 75 -0.246239896101871

46 76 -0.200320367358091

47 1 -0.238612126082988

47 2 -0.235745454221533

47 3 -0.165721312799190

47 4 -0.0927218849017192

47 5 0.0416922762381286

47 6 0.0301562035766109

47 7 0.00533784138878882

47 8 -0.00854607907797244

47 9 -0.00225412876026185

47 10 -0.0529222337295284

47 11 -0.00522680329043755

47 12 -0.0442790540756840

47 13 0.0134903859971955

47 14 -0.0902295949168377

47 15 -0.0691605160939374

47 16 -0.160989722810242

47 17 -0.131070928249911

47 18 -0.146464482644840

47 19 -0.129867451555889

47 20 -0.0815065888024994

47 21 -0.0206890855218813

47 22 0.0891175747358189

47 23 0.182337008185451

47 24 0.197744202732692

47 25 0.104303680590819

47 26 0.126983139731755

47 27 0.220945727140230

47 28 0.195855491622012

47 29 0.116694650463598

47 30 0.171824560613835

47 31 0.226677090533988

47 32 0.170182231407929

47 33 0.112170160021960

47 34 0.139021433966244

47 35 0.192552461042156

47 36 0.222824596047032

47 37 0.262495784818356

47 38 0.256182491179194

47 39 0.217330778094621

47 40 0.230947988054376

47 41 0.204289689571830

47 42 0.105744086006501

47 43 0.263947729653912

47 44 0.501664001056702

47 45 0.572570928974721

47 46 0.752752771176807

47 47 1.00000000000000

47 48 0.668610068728902

47 49 0.450250377703283

47 50 0.253380011737281

47 51 0.206561681085993

47 52 0.247375884862649

47 53 0.203360714026464

47 54 0.131252349542171

47 55 0.0238484138487898

47 56 -0.0481529786331224

47 57 -0.106786549211475

47 58 -0.0346389349513012

47 59 -0.0192180733884352

47 60 -0.113868596109578

47 61 -0.102803131045169

47 62 -0.128819282119371

47 63 -0.198101469777256

47 64 -0.204697592015909

47 65 -0.134700844565960

47 66 -0.0565109645254161

47 67 0.118948296356714

47 68 0.228582066328218

47 69 0.157750615248664

47 70 0.0801388342066785

47 71 -0.0209552511409071

47 72 -0.281514167088690

47 73 -0.309816274269156

47 74 -0.334848995092675

47 75 -0.292598337499400

47 76 -0.260889392654575

48 1 -0.288459974785120

48 2 -0.271687376845305

48 3 -0.187622499642693

48 4 -0.0628414300200551

48 5 0.0796871643706093

48 6 0.0479172454005287

48 7 -0.00106341693814065

48 8 -0.0538457068907684

48 9 -0.0230410472410585

48 10 -0.0623166764738033

48 11 0.0434309856111962

48 12 0.0181339207519091

48 13 0.0810285030503207

48 14 -0.0418749881309828

48 15 -0.0650705539743671

48 16 -0.178706778678077

48 17 -0.178206439676509

48 18 -0.227471567726841

48 19 -0.231946385522452

48 20 -0.171353912589847

48 21 -0.0654309689254546

48 22 0.0805631364167909

48 23 0.199235669875434

48 24 0.228531375013350

48 25 0.131710046259046

48 26 0.153382922456228

48 27 0.237574721622034

48 28 0.219492988820487

48 29 0.156825770862357

48 30 0.196981858481256

48 31 0.230558716122665

48 32 0.186909812446692

48 33 0.137036155017400

48 34 0.151535867140108

48 35 0.200830778498876

48 36 0.230837971272422

48 37 0.242838701276551

48 38 0.226578419539726

48 39 0.155715697655483

48 40 0.219476657780010

48 41 0.219433616451332

48 42 0.146971482513496

48 43 0.324958674975204

48 44 0.543857493971171

48 45 0.547611695971287

48 46 0.472783965282676

48 47 0.668610068728902

48 48 0.999999999999994

48 49 0.726942496388247

48 50 0.437713834536974

48 51 0.296493576621000

48 52 0.316859101200439

48 53 0.213773263324673

48 54 0.108042892929576

48 55 -0.0415307778786070

48 56 -0.132383064974524

48 57 -0.222687518730347

48 58 -0.133638452831847

48 59 -0.0719457247918226

48 60 -0.187340396680312

48 61 -0.161578757775472

48 62 -0.192061297183140

48 63 -0.255814195829219

48 64 -0.222758961142731

48 65 -0.154153881946913

48 66 -0.0127122348797714

48 67 0.137403765493832

48 68 0.208403728589775

48 69 0.179715615731639

48 70 0.136193671578227

48 71 0.0139201281843830

48 72 -0.253775667954240

48 73 -0.298254691351336

48 74 -0.382504172963010

48 75 -0.351707697609511

48 76 -0.315897884868240

49 1 -0.343371575030456

49 2 -0.334358559218230

49 3 -0.220319213954468

49 4 -0.0798807263026438

49 5 0.0975672743814395

49 6 0.0846959491506250

49 7 -0.0270990682316091

49 8 -0.0761229764985935

49 9 -0.0394943478087960

49 10 -0.0572263263050114

49 11 0.0459061552259240

49 12 0.0285316847971507

49 13 0.0683987588110633

49 14 -0.0957815190829245

49 15 -0.123412531196502

49 16 -0.242726174050365

49 17 -0.220518300528820

49 18 -0.256272021482412

49 19 -0.220083798007458

49 20 -0.139594390121234

49 21 -0.0216144931148336

49 22 0.192837192033124

49 23 0.342002200542565

49 24 0.334784821484472

49 25 0.219636857886538

49 26 0.248011297230893

49 27 0.316798612934825

49 28 0.284515685222322

49 29 0.228487965043374

49 30 0.247650393697616

49 31 0.259108452021594

49 32 0.215578086924068

49 33 0.155464221049474

49 34 0.156830938243977

49 35 0.214722268388949

49 36 0.253469721745123

49 37 0.278522018428579

49 38 0.268042899433471

49 39 0.176150203750048

49 40 0.263419025414801

49 41 0.309714824181340

49 42 0.228118732027566

49 43 0.496690891370101

49 44 0.590291456601465

49 45 0.468968635191495

49 46 0.322080515758761

49 47 0.450250377703283

49 48 0.726942496388247

49 49 0.999999999999996

49 50 0.738132134121829

49 51 0.529228741705334

49 52 0.490368278734120

49 53 0.351790398515543

49 54 0.268604026100588

49 55 0.0914039986247025

49 56 -0.00551499453529164

49 57 -0.133500415230787

49 58 0.00956631242710682

49 59 0.100341662096129

49 60 -0.0761266461281717

49 61 -0.0781083733515905

49 62 -0.197068977649342

49 63 -0.311329874959766

49 64 -0.272916171381225

49 65 -0.163864366928067

49 66 0.0305874749893966

49 67 0.211076365346155

49 68 0.285559200672106

49 69 0.283497531874798

49 70 0.238397258179978

49 71 0.0879930962141399

49 72 -0.344794559192357

49 73 -0.379050018464919

49 74 -0.498323962032473

49 75 -0.421999697703138

49 76 -0.404060714236497

50 1 -0.357434843135184

50 2 -0.366089278004245

50 3 -0.241735443859260

50 4 -0.141152447657373

50 5 0.0505965917774069

50 6 0.0787685020052290

50 7 -0.0245647959864775

50 8 -0.0279164757650555

50 9 -0.0185762876843169

50 10 -0.0311197170401487

50 11 -0.000153169851429767

50 12 -0.0300668604430926

50 13 -0.0122873615870891

50 14 -0.177290994440649

50 15 -0.177897443730812

50 16 -0.280643567442670

50 17 -0.227426352885942

50 18 -0.246674763503752

50 19 -0.177936683191951

50 20 -0.0846787484416835

50 21 0.0445276262404399

50 22 0.318786937504185

50 23 0.470046316745618

50 24 0.433173259992748

50 25 0.299950204839159

50 26 0.303420758190199

50 27 0.349796577148796

50 28 0.306584799864000

50 29 0.238915940005258

50 30 0.217554636281735

50 31 0.215696068340618

50 32 0.179029767261787

50 33 0.112963536855859

50 34 0.0918325658175992

50 35 0.146718945603603

50 36 0.183849071395049

50 37 0.235728478704629

50 38 0.251631889878619

50 39 0.156839060721653

50 40 0.234154636105367

50 41 0.308136330445591

50 42 0.228777023267560

50 43 0.478803831089908

50 44 0.474491098743508

50 45 0.405900046427514

50 46 0.229331207065044

50 47 0.253380011737281

50 48 0.437713834536974

50 49 0.738132134121829

50 50 0.999999999999999

50 51 0.796291207339102

50 52 0.647410036534749

50 53 0.477210154886230

50 54 0.434563311699655

50 55 0.228414081440881

50 56 0.122503819952957

50 57 -0.0412294068879619

50 58 0.126758328177300

50 59 0.216056062688004

50 60 0.0106101157394276

50 61 0.0105764931788558

50 62 -0.141196780233110

50 63 -0.315415146560752

50 64 -0.309390196272611

50 65 -0.165884737584896

50 66 0.0222024476058025

50 67 0.208755484998425

50 68 0.259589663263156

50 69 0.294897152508701

50 70 0.227168963761151

50 71 0.116235169866571

50 72 -0.384069845846595

50 73 -0.376602209367456

50 74 -0.507434477515846

50 75 -0.416296030446046

50 76 -0.421042136380109

51 1 -0.336863834655617

51 2 -0.396291609145687

51 3 -0.260151434118674

51 4 -0.198127432619954

51 5 -0.000136213143166742

51 6 0.0700736217416641

51 7 -0.0658308445011280

51 8 -0.0172433651116073

51 9 -0.0305275619314293

51 10 -0.0109732067953942

51 11 -0.0560811076105973

51 12 -0.0878261452650863

51 13 -0.0995565236328177

51 14 -0.265704401788561

51 15 -0.219696096999181

51 16 -0.296217470049655

51 17 -0.178684981012587

51 18 -0.174583959208093

51 19 -0.0719481861743629

51 20 0.0381236674514135

51 21 0.172201879986832

51 22 0.485271768198624

51 23 0.584268196758109

51 24 0.547064166415950

51 25 0.398142114460690

51 26 0.353571028126993

51 27 0.379197982609277

51 28 0.338414841524761

51 29 0.273279590658531

51 30 0.215785783758508

51 31 0.203571156184957

51 32 0.188586530830110

51 33 0.135214486309198

51 34 0.0867499131122163

51 35 0.133374624161164

51 36 0.151090716894076

51 37 0.197156680239684

51 38 0.200182734784397

51 39 0.0952650460374991

51 40 0.177005005891840

51 41 0.271209817008286

51 42 0.114413041729659

51 43 0.347482961899283

51 44 0.379527181425727

51 45 0.354214063082111

51 46 0.221883331578571

51 47 0.206561681085993

51 48 0.296493576621000

51 49 0.529228741705334

51 50 0.796291207339102

51 51 1.00000000000001

51 52 0.847050596097083

51 53 0.690829476726183

51 54 0.649838598436183

51 55 0.422276102460749

51 56 0.271968600513624

51 57 0.0881576548087832

51 58 0.285245904921500

51 59 0.311704362092816

51 60 0.0619665807992847

51 61 0.0332802953991418

51 62 -0.127447043656247

51 63 -0.306617803350715

51 64 -0.342980619617530

51 65 -0.151226286621975

51 66 -4.74598935370602e-05

51 67 0.185658897679212

51 68 0.218693163385564

51 69 0.299622900657755

51 70 0.209508682383401

51 71 0.149897024423254

51 72 -0.439442058825288

51 73 -0.367229731823876

51 74 -0.524913514459773

51 75 -0.401307741590352

51 76 -0.420036177923829

52 1 -0.312930592945855

52 2 -0.394365884751820

52 3 -0.275039819629480

52 4 -0.222940200952871

52 5 -0.0227185290185540

52 6 0.0703096309701512

52 7 -0.0800945043023872

52 8 -0.0164419872987444

52 9 -0.0300134407966494

52 10 -0.00365135644766335

52 11 -0.0534083538598795

52 12 -0.0938505766610064

52 13 -0.116819048982305

52 14 -0.264364302545700

52 15 -0.177629788505407

52 16 -0.237610704180043

52 17 -0.0980685573902540

52 18 -0.100902030831909

52 19 -0.0169599082384034

52 20 0.124226754938881

52 21 0.300257336805718

52 22 0.646259454793745

52 23 0.743748052443339

52 24 0.718570228529600

52 25 0.566493849496130

52 26 0.503978309369873

52 27 0.515217445409856

52 28 0.451988881731006

52 29 0.348146687667263

52 30 0.273125104845380

52 31 0.256819988350278

52 32 0.212908813925337

52 33 0.138860121997463

52 34 0.0939486967536529

52 35 0.155694298737312

52 36 0.177555402927229

52 37 0.242773170880872

52 38 0.257721983540373

52 39 0.156344407595124

52 40 0.210798427960952

52 41 0.264519984145851

52 42 0.0364620792741350

52 43 0.280153806563272

52 44 0.350433698842628

52 45 0.336366194862857

52 46 0.231535869041422

52 47 0.247375884862649

52 48 0.316859101200439

52 49 0.490368278734120

52 50 0.647410036534749

52 51 0.847050596097083

52 52 1.00000000000000

52 53 0.846676575481500

52 54 0.723544949822727

52 55 0.490941442207316

52 56 0.299527842739005

52 57 0.0866452791746145

52 58 0.263341780297363

52 59 0.219579518922714

52 60 -0.0419823233118192

52 61 -0.0566041536715809

52 62 -0.185180389897634

52 63 -0.324742268855006

52 64 -0.370584883197624

52 65 -0.201390071182073

52 66 -0.0426120013231203

52 67 0.143396093266720

52 68 0.191865501735091

52 69 0.284659711271535

52 70 0.164859534999048

52 71 0.118765765888168

52 72 -0.507049703691096

52 73 -0.428919879711488

52 74 -0.564621415908717

52 75 -0.436025944910655

52 76 -0.427670166682457

53 1 -0.263323324790404

53 2 -0.385852043075419

53 3 -0.269094822820421

53 4 -0.254810337951852

53 5 -0.0665821997875255

53 6 0.0700773166255978

53 7 -0.0251944943159643

53 8 0.0858269943815118

53 9 0.0293428506583785

53 10 0.0292032481984742

53 11 -0.105912842554103

53 12 -0.176691348511413

53 13 -0.192255640928905

53 14 -0.336299508127195

53 15 -0.218059739325985

53 16 -0.243636013992326

53 17 -0.0444016512895811

53 18 0.00271807557738989

53 19 0.120360001879666

53 20 0.278836561245545

53 21 0.420397439495774

53 22 0.728351736212643

53 23 0.718885152347385

53 24 0.687192034125363

53 25 0.540004325971773

53 26 0.461178620814244

53 27 0.463217174233836

53 28 0.403383858678547

53 29 0.286031548029565

53 30 0.207732732942776

53 31 0.202120248482667

53 32 0.151303189025129

53 33 0.0762476464260727

53 34 0.0330327016292039

53 35 0.0895058698640992

53 36 0.117148387802487

53 37 0.211228093115801

53 38 0.245172117128037

53 39 0.181474206912657

53 40 0.178215714353255

53 41 0.204688916026123

53 42 0.00683684549753752

53 43 0.193706770384449

53 44 0.278916763718784

53 45 0.265386031588244

53 46 0.186668741284684

53 47 0.203360714026464

53 48 0.213773263324673

53 49 0.351790398515543

53 50 0.477210154886230

53 51 0.690829476726183

53 52 0.846676575481500

53 53 1.00000000000001

53 54 0.867187960343073

53 55 0.653394199403889

53 56 0.443616024114722

53 57 0.267907879740258

53 58 0.420731283855814

53 59 0.308671980201444

53 60 0.0815956424365240

53 61 0.0160463330831014

53 62 -0.135210575461896

53 63 -0.266683795109613

53 64 -0.352749568302450

53 65 -0.161639559454469

53 66 -0.0669488839198843

53 67 0.109803931242701

53 68 0.182245526310764

53 69 0.240529594341223

53 70 0.0907192952663549

53 71 0.0793198472527346

53 72 -0.520221081884486

53 73 -0.425064242341143

53 74 -0.532170008256818

53 75 -0.421278278297628

53 76 -0.421244341444612

54 1 -0.187306716192574

54 2 -0.347220724838992

54 3 -0.258148837495862

54 4 -0.299774471649710

54 5 -0.155297736830575

54 6 -0.0117621950934719

54 7 -0.0695713827248708

54 8 0.0829137548339379

54 9 -0.000375292477385943

54 10 0.00258718746140266

54 11 -0.204151237345295

54 12 -0.273715042252644

54 13 -0.286167277268263

54 14 -0.410692136005659

54 15 -0.290300655553292

54 16 -0.246677582722690

54 17 -0.0100671613253973

54 18 0.0994548314189555

54 19 0.271008396822406

54 20 0.425886850134223

54 21 0.493951261122026

54 22 0.736956092030741

54 23 0.650709853927539

54 24 0.545173682857018

54 25 0.410953695521712

54 26 0.329591281812336

54 27 0.303061142560720

54 28 0.243996444834219

54 29 0.141750907710745

54 30 0.0656776514749720

54 31 0.0746484761750143

54 32 0.0395678264831561

54 33 -0.0206962238663561

54 34 -0.0542851112683376

54 35 -0.00336488274169568

54 36 0.0252751621512333

54 37 0.132673259484736

54 38 0.163979888485314

54 39 0.126889244745911

54 40 0.115465976312608

54 41 0.134167872323175

54 42 -0.0256296858750736

54 43 0.121815904897006

54 44 0.193348988846565

54 45 0.186336860578730

54 46 0.119130805829793

54 47 0.131252349542171

54 48 0.108042892929576

54 49 0.268604026100588

54 50 0.434563311699655

54 51 0.649838598436183

54 52 0.723544949822727

54 53 0.867187960343073

54 54 0.999999999999996

54 55 0.841698956938794

54 56 0.633935276777295

54 57 0.490312160078665

54 58 0.631560706809302

54 59 0.482760489776309

54 60 0.273039512869664

54 61 0.163015536929640

54 62 -0.0365293149146756

54 63 -0.175726502050269

54 64 -0.294563872209241

54 65 -0.106670551056279

54 66 -0.100051301684531

54 67 0.0486398676439365

54 68 0.0987208730904533

54 69 0.181450192783650

54 70 0.0624739232875274

54 71 0.126364286044901

54 72 -0.473266882240511

54 73 -0.323874706904717

54 74 -0.429025261320511

54 75 -0.341998493505801

54 76 -0.369261350406810

55 1 0.00161975121459823

55 2 -0.185888618549342

55 3 -0.171618639525314

55 4 -0.290751264377431

55 5 -0.241283854309956

55 6 -0.132889115908474

55 7 -0.160171759837404

55 8 0.0119070711071081

55 9 -0.0728945320737099

55 10 -0.0514928241455779

55 11 -0.285961251623551

55 12 -0.330539562473142

55 13 -0.331436332292700

55 14 -0.389368923904673

55 15 -0.251650802549309

55 16 -0.107177290580992

55 17 0.145059715038444

55 18 0.308732450606063

55 19 0.499066893699061

55 20 0.631933198594477

55 21 0.605351772413063

55 22 0.673336995688111

55 23 0.499422562128675

55 24 0.371233979238221

55 25 0.298229339638018

55 26 0.187710074769586

55 27 0.127067438312924

55 28 0.0811850738267566

55 29 -0.0225100796050307

55 30 -0.103806637042299

55 31 -0.0798581654473464

55 32 -0.110195037144326

55 33 -0.157447791923796

55 34 -0.175114279297449

55 35 -0.127422800067267

55 36 -0.0953301103457450

55 37 0.0216137713318580

55 38 0.0478631096783052

55 39 0.0560998628358327

55 40 0.0176999661098944

55 41 0.00124737222319550

55 42 -0.123754273856407

55 43 -0.0273156736970159

55 44 0.0248259462688504

55 45 0.0426306558511595

55 46 0.0205322806304670

55 47 0.0238484138487898

55 48 -0.0415307778786070

55 49 0.0914039986247025

55 50 0.228414081440881

55 51 0.422276102460749

55 52 0.490941442207316

55 53 0.653394199403889

55 54 0.841698956938794

55 55 0.999999999999999

55 56 0.830963387531723

55 57 0.696349664322046

55 58 0.745862813199086

55 59 0.568217189655615

55 60 0.418761934207275

55 61 0.301034305838805

55 62 0.0840319903832538

55 63 -0.0200829091868017

55 64 -0.175196363557710

55 65 -0.0361302757508555

55 66 -0.135090098446377

55 67 -0.0536065074773991

55 68 -0.0526089909055146

55 69 0.0479556334353272

55 70 -0.0147593531057074

55 71 0.147883272805084

55 72 -0.347862458413672

55 73 -0.147555044772679

55 74 -0.233492555385330

55 75 -0.178024820051105

55 76 -0.221314970216677

56 1 0.106354915769619

56 2 -0.0654586241962497

56 3 -0.0976954343520798

56 4 -0.264494650620492

56 5 -0.298274878987262

56 6 -0.231928349619694

56 7 -0.233857711762147

56 8 -0.0730494602286688

56 9 -0.144957877855534

56 10 -0.111114565129284

56 11 -0.328155145522425

56 12 -0.340735367765571

56 13 -0.336369711768487

56 14 -0.340230291520777

56 15 -0.227940641179067

56 16 -0.0466288465665676

56 17 0.182644128149903

56 18 0.366379996278892

56 19 0.588711877624062

56 20 0.617256901139366

56 21 0.512813052928819

56 22 0.497893244858710

56 23 0.333395604972596

56 24 0.175552626901817

56 25 0.142946386804073

56 26 0.0652817915371818

56 27 -0.0218628651822969

56 28 -0.0582483570639615

56 29 -0.132740823854637

56 30 -0.203407682711983

56 31 -0.172412261617457

56 32 -0.188862405278907

56 33 -0.225462481767968

56 34 -0.231015000947799

56 35 -0.186802459125537

56 36 -0.158285841401695

56 37 -0.0464134971634152

56 38 -0.0328039077245513

56 39 -0.000644205651995644

56 40 -0.0417792972226716

56 41 -0.0891462167198205

56 42 -0.170914069669773

56 43 -0.115034816583208

56 44 -0.0875139301909092

56 45 -0.0560328771162474

56 46 -0.0497070272748834

56 47 -0.0481529786331224

56 48 -0.132383064974524

56 49 -0.00551499453529164

56 50 0.122503819952957

56 51 0.271968600513624

56 52 0.299527842739005

56 53 0.443616024114722

56 54 0.633935276777295

56 55 0.830963387531723

56 56 1.00000000000000

56 57 0.818767042223064

56 58 0.754498888877798

56 59 0.651735236790509

56 60 0.582482301768901

56 61 0.513443244089434

56 62 0.236714204711578

56 63 0.114714990754732

56 64 -0.0596032991112392

56 65 0.0168407707579591

56 66 -0.148325616148237

56 67 -0.125497021099054

56 68 -0.168005750136485

56 69 -0.0637384957970534

56 70 -0.0551719987362384

56 71 0.160565074555230

56 72 -0.230338967429810

56 73 -0.0142105569644225

56 74 -0.0712490848006482

56 75 -0.0488405138628397

56 76 -0.106451284087994

57 1 0.188181824696145

57 2 0.0215873353210165

57 3 -0.0483866074542978

57 4 -0.259043206754195

57 5 -0.348942861244660

57 6 -0.291546205109951

57 7 -0.245228721320913

57 8 -0.0599566479956144

57 9 -0.151367646014858

57 10 -0.114791784885550

57 11 -0.387848782217181

57 12 -0.401950037036803

57 13 -0.407270989144545

57 14 -0.354717990892345

57 15 -0.256802379119630

57 16 -0.0321631985865295

57 17 0.171577327399390

57 18 0.405560503541565

57 19 0.641052655571153

57 20 0.662360674416824

57 21 0.433759229807823

57 22 0.349119794470117

57 23 0.134612870281996

57 24 -0.0370697406636623

57 25 -0.0390844723888981

57 26 -0.0946431249037705

57 27 -0.199056109441328

57 28 -0.227057944356788

57 29 -0.257210274067920

57 30 -0.296939526850533

57 31 -0.260757594233014

57 32 -0.258574285679571

57 33 -0.265528664233838

57 34 -0.254181218881941

57 35 -0.229275345555709

57 36 -0.209736959472802

57 37 -0.119212749727555

57 38 -0.114700941414388

57 39 -0.0524973344918280

57 40 -0.109336340848237

57 41 -0.177779215742426

57 42 -0.196045443189848

57 43 -0.205485264894241

57 44 -0.182032828393057

57 45 -0.164396777356483

57 46 -0.120433701553034

57 47 -0.106786549211475

57 48 -0.222687518730347

57 49 -0.133500415230787

57 50 -0.0412294068879619

57 51 0.0881576548087832

57 52 0.0866452791746145

57 53 0.267907879740258

57 54 0.490312160078665

57 55 0.696349664322046

57 56 0.818767042223064

57 57 1.00000000000000

57 58 0.869303805741517

57 59 0.691206219528285

57 60 0.714670449930499

57 61 0.516085172115036

57 62 0.275111530380727

57 63 0.223759749811977

57 64 0.0459465894459026

57 65 0.0815215143946897

57 66 -0.162869358134448

57 67 -0.180572595925184

57 68 -0.242236220594742

57 69 -0.122052352854214

57 70 -0.0800169683662577

57 71 0.187008177017283

57 72 -0.111844652215545

57 73 0.120186609158297

57 74 0.0967155461915984

57 75 0.0816318662569893

57 76 0.0120405505885534

58 1 0.0376796638934549

58 2 -0.123506426601523

58 3 -0.118762572691842

58 4 -0.275438905322787

58 5 -0.297063042509064

58 6 -0.212252478123496

58 7 -0.206366044348667

58 8 -0.0267126668040945

58 9 -0.112939503490429

58 10 -0.0949396451829267

58 11 -0.351999835952509

58 12 -0.385015058748720

58 13 -0.397250930855526

58 14 -0.414767467577902

58 15 -0.325945005522618

58 16 -0.170905719975078

58 17 0.0395914158074279

58 18 0.260674840991399

58 19 0.501010786826243

58 20 0.554679319623439

58 21 0.365199180727984

58 22 0.399945699263533

58 23 0.234805552535994

58 24 0.0788570290366298

58 25 0.0258233327584440

58 26 -0.0136252738269834

58 27 -0.0863439000446589

58 28 -0.115909780889583

58 29 -0.134756737029293

58 30 -0.167465340726109

58 31 -0.135795251525050

58 32 -0.132577342915679

58 33 -0.147160704398690

58 34 -0.145850026858779

58 35 -0.117353820209460

58 36 -0.100908550079968

58 37 -0.0158563445630664

58 38 -0.0245707519217449

58 39 0.00315100215686104

58 40 -0.0219840331592581

58 41 -0.0626651421432072

58 42 -0.144445121556232

58 43 -0.0982496566280113

58 44 -0.0599179255862576

58 45 -0.0438850808486811

58 46 -0.0365105799265686

58 47 -0.0346389349513012

58 48 -0.133638452831847

58 49 0.00956631242710682

58 50 0.126758328177300

58 51 0.285245904921500

58 52 0.263341780297363

58 53 0.420731283855814

58 54 0.631560706809302

58 55 0.745862813199086

58 56 0.754498888877798

58 57 0.869303805741517

58 58 1.00000000000000

58 59 0.826272104899019

58 60 0.691733494099880

58 61 0.451270346824405

58 62 0.196401106345377

58 63 0.121561269090774

58 64 -0.0344584595133307

58 65 0.0556391214639162

58 66 -0.122440380503108

58 67 -0.0887474154063696

58 68 -0.129801950554370

58 69 -0.0190081785795026

58 70 -0.0269833569308683

58 71 0.187864156309492

58 72 -0.244131673711455

58 73 -0.0251463106986951

58 74 -0.0638149659058881

58 75 -0.0428796563458465

58 76 -0.100279425700257

59 1 -0.0160547176727609

59 2 -0.122029212189385

59 3 -0.0935865131770527

59 4 -0.225349123305456

59 5 -0.254353306572235

59 6 -0.214888119723245

59 7 -0.218958849130159

59 8 -0.0886275648678257

59 9 -0.143298261497118

59 10 -0.135466145076814

59 11 -0.315689845451895

59 12 -0.325797300006535

59 13 -0.324591782199784

59 14 -0.356121586369667

59 15 -0.310707755952353

59 16 -0.212826644651397

59 17 -0.0495168815369556

59 18 0.139286505528416

59 19 0.356269627633663

59 20 0.394746431185262

59 21 0.208026797388547

59 22 0.247727899393994

59 23 0.153696281993018

59 24 0.0202014969704312

59 25 -0.0322449959108062

59 26 -0.0400810679462125

59 27 -0.0927869496496677

59 28 -0.111253615546749

59 29 -0.109288406462961

59 30 -0.124907538768950

59 31 -0.0927758854818175

59 32 -0.0859328981734868

59 33 -0.0980123497604654

59 34 -0.0914166512160889

59 35 -0.0626726752595107

59 36 -0.0443813748834433

59 37 0.0185133117225431

59 38 -0.0117050407643463

59 39 0.00681204362551347

59 40 0.0172411308831053

59 41 -0.0222466189716516

59 42 -0.0996888619987405

59 43 -0.0314748619902927

59 44 -0.0126373048250788

59 45 0.00529691320046817

59 46 -0.0141670617915576

59 47 -0.0192180733884352

59 48 -0.0719457247918226

59 49 0.100341662096129

59 50 0.216056062688004

59 51 0.311704362092816

59 52 0.219579518922714

59 53 0.308671980201444

59 54 0.482760489776309

59 55 0.568217189655615

59 56 0.651735236790509

59 57 0.691206219528285

59 58 0.826272104899019

59 59 0.999999999999994

59 60 0.787986572712276

59 61 0.610565704445288

59 62 0.282909825096409

59 63 0.156715534326140

59 64 0.00128932204416753

59 65 0.0743760606850061

59 66 -0.0747726772068586

59 67 -0.0399516448085359

59 68 -0.0988163816360776

59 69 -0.0217054276998655

59 70 0.00165341140489927

59 71 0.171607898163296

59 72 -0.225150216998746

59 73 -0.0368817951533310

59 74 -0.0775044134601471

59 75 -0.0669235490441791

59 76 -0.120679531013528

60 1 0.125971993152701

60 2 0.0366690512777581

60 3 -0.00812714645261742

60 4 -0.191056166119653

60 5 -0.298898784707803

60 6 -0.278736361728735

60 7 -0.213703577293040

60 8 -0.0790633290590613

60 9 -0.150460060943839

60 10 -0.144105953406041

60 11 -0.348554403001546

60 12 -0.346597260001729

60 13 -0.341682917392304

60 14 -0.295885974595362

60 15 -0.267713159062334

60 16 -0.121353301119025

60 17 -0.00157054061629202

60 18 0.197159333965133

60 19 0.393236749133896

60 20 0.390947126283999

60 21 0.150139899302295

60 22 0.103011164427958

60 23 -0.0338331209679561

60 24 -0.181836959584122

60 25 -0.183485563964521

60 26 -0.179746000901200

60 27 -0.265622479091877

60 28 -0.278708018489905

60 29 -0.255136274796739

60 30 -0.265418347992999

60 31 -0.236569711964026

60 32 -0.214528658708388

60 33 -0.202720922538424

60 34 -0.185769118408748

60 35 -0.179743355121872

60 36 -0.167706080358953

60 37 -0.114234054029289

60 38 -0.127603363816872

60 39 -0.0710960516226592

60 40 -0.0922158926645226

60 41 -0.157268858252425

60 42 -0.127701798202939

60 43 -0.149298000788379

60 44 -0.156887945920053

60 45 -0.147512033815184

60 46 -0.121076295219751

60 47 -0.113868596109578

60 48 -0.187340396680312

60 49 -0.0761266461281717

60 50 0.0106101157394276

60 51 0.0619665807992847

60 52 -0.0419823233118192

60 53 0.0815956424365240

60 54 0.273039512869664

60 55 0.418761934207275

60 56 0.582482301768901

60 57 0.714670449930499

60 58 0.691733494099880

60 59 0.787986572712276

60 60 1.00000000000001

60 61 0.771454836702849

60 62 0.416469074220039

60 63 0.306726426327484

60 64 0.136349662668436

60 65 0.140026416262892

60 66 -0.0980490039561699

60 67 -0.128380784380342

60 68 -0.203923279922078

60 69 -0.145781047817564

60 70 -0.0751677734960627

60 71 0.139346939092420

60 72 -0.0532356381325968

60 73 0.140894594192938

60 74 0.133184153738737

60 75 0.0903842064008005

60 76 0.0255409850992461

61 1 0.167641580652979

61 2 0.118634806044839

61 3 0.0670453458538874

61 4 -0.142138560746488

61 5 -0.277498229383877

61 6 -0.307567017329528

61 7 -0.269963816525498

61 8 -0.167541895226546

61 9 -0.215637293231493

61 10 -0.193750689562569

61 11 -0.336650827650494

61 12 -0.295130486437865

61 13 -0.281166358559638

61 14 -0.212682056623568

61 15 -0.179116698113568

61 16 -0.0335647998909061

61 17 0.0422517676292272

61 18 0.174556660213795

61 19 0.316361799061105

61 20 0.279352877316805

61 21 0.103025744914019

61 22 0.0405910436273335

61 23 -0.0607846085646331

61 24 -0.178282044577170

61 25 -0.173392478411657

61 26 -0.177286137732194

61 27 -0.255689287364284

61 28 -0.265858846003129

61 29 -0.256971219207662

61 30 -0.270113982984004

61 31 -0.241236465982160

61 32 -0.225368675509274

61 33 -0.213641866298583

61 34 -0.193913650640666

61 35 -0.186008606622787

61 36 -0.173654524340932

61 37 -0.126193298066723

61 38 -0.144643668207900

61 39 -0.0914869605376868

61 40 -0.100650650199134

61 41 -0.169653682199992

61 42 -0.138953793827494

61 43 -0.148284804594710

61 44 -0.152533887029116

61 45 -0.107141662716885

61 46 -0.0992245679190379

61 47 -0.102803131045169

61 48 -0.161578757775472

61 49 -0.0781083733515905

61 50 0.0105764931788558

61 51 0.0332802953991418

61 52 -0.0566041536715809

61 53 0.0160463330831014

61 54 0.163015536929640

61 55 0.301034305838805

61 56 0.513443244089434

61 57 0.516085172115036

61 58 0.451270346824405

61 59 0.610565704445288

61 60 0.771454836702849

61 61 0.999999999999996

61 62 0.691450821456068

61 63 0.433369019182809

61 64 0.201549002923352

61 65 0.210730826204034

61 66 -0.0431266560735218

61 67 -0.0994430948105630

61 68 -0.213741172029079

61 69 -0.175882194043350

61 70 -0.0805711925140545

61 71 0.127624059181368

61 72 -0.00847291264442932

61 73 0.169640819950550

61 74 0.156258355566074

61 75 0.118648925102809

61 76 0.0591229044152939

62 1 0.311458962757144

62 2 0.264107898983485

62 3 0.175261650828467

62 4 -0.0790638583386400

62 5 -0.267885739596285

62 6 -0.335058912284400

62 7 -0.286871210209610

62 8 -0.204628743114408

62 9 -0.239498300528107

62 10 -0.208152797000215

62 11 -0.321006007564857

62 12 -0.260048017924388

62 13 -0.240422264762972

62 14 -0.115368319204551

62 15 -0.0820870957068049

62 16 0.102335378759584

62 17 0.119069935775373

62 18 0.193443592141294

62 19 0.236663513096446

62 20 0.158558149468442

62 21 0.0129604869942424

62 22 -0.0935033241661838

62 23 -0.179800499809903

62 24 -0.250202282469765

62 25 -0.228799909327696

62 26 -0.245333781370702

62 27 -0.307611593744518

62 28 -0.313785793846355

62 29 -0.304553966991581

62 30 -0.307713731155243

62 31 -0.278545923045734

62 32 -0.264171031485963

62 33 -0.233853329477765

62 34 -0.210081334437904

62 35 -0.221087230948412

62 36 -0.215816079659353

62 37 -0.184956954471162

62 38 -0.199154842331156

62 39 -0.133957849324211

62 40 -0.159965531052400

62 41 -0.230981004126467

62 42 -0.186590749167887

62 43 -0.228323150668737

62 44 -0.218728016801569

62 45 -0.126044700642521

62 46 -0.111317045814239

62 47 -0.128819282119371

62 48 -0.192061297183140

62 49 -0.197068977649342

62 50 -0.141196780233110

62 51 -0.127447043656247

62 52 -0.185180389897634

62 53 -0.135210575461896

62 54 -0.0365293149146756

62 55 0.0840319903832538

62 56 0.236714204711578

62 57 0.275111530380727

62 58 0.196401106345377

62 59 0.282909825096409

62 60 0.416469074220039

62 61 0.691450821456068

62 62 1.00000000000000

62 63 0.655208145276299

62 64 0.318477333209766

62 65 0.326430774169992

62 66 0.0139278231349654

62 67 -0.121080409090253

62 68 -0.264263425824135

62 69 -0.241123548316325

62 70 -0.138282112804547

62 71 0.0878000554555245

62 72 0.116420277972036

62 73 0.275210839228530

62 74 0.287913497256985

62 75 0.252111793297936

62 76 0.208877260323272

63 1 0.523864476864804

63 2 0.514414424223435

63 3 0.359211820009578

63 4 0.0506504457184348

63 5 -0.250016985360942

63 6 -0.336130001022111

63 7 -0.293266379561740

63 8 -0.216322771593439

63 9 -0.268948336978053

63 10 -0.221728819587507

63 11 -0.340346172769131

63 12 -0.244315861178220

63 13 -0.232467324688231

63 14 -0.0129026497099316

63 15 0.0702893764356490

63 16 0.314789416730528

63 17 0.278297238245613

63 18 0.310231474469525

63 19 0.267804951391960

63 20 0.176734529771753

63 21 0.0287560266221486

63 22 -0.167914920996779

63 23 -0.291276013283811

63 24 -0.352353178086665

63 25 -0.293635605840543

63 26 -0.337022449243922

63 27 -0.414478151019219

63 28 -0.407649516463129

63 29 -0.406146724695539

63 30 -0.418080409153968

63 31 -0.384410780825311

63 32 -0.374681229584094

63 33 -0.335820293443006

63 34 -0.303023221673046

63 35 -0.315615238610815

63 36 -0.304541421727446

63 37 -0.270727420637224

63 38 -0.281657431956191

63 39 -0.188926358635535

63 40 -0.228842008536651

63 41 -0.322998167695646

63 42 -0.248272063643332

63 43 -0.316725675513029

63 44 -0.303999559168597

63 45 -0.235990612144372

63 46 -0.186327644111396

63 47 -0.198101469777256

63 48 -0.255814195829219

63 49 -0.311329874959766

63 50 -0.315415146560752

63 51 -0.306617803350715

63 52 -0.324742268855006

63 53 -0.266683795109613

63 54 -0.175726502050269

63 55 -0.0200829091868017

63 56 0.114714990754732

63 57 0.223759749811977

63 58 0.121561269090774

63 59 0.156715534326140

63 60 0.306726426327484

63 61 0.433369019182809

63 62 0.655208145276299

63 63 0.999999999999994

63 64 0.666898291871249

63 65 0.430243200058957

63 66 0.0999258143004004

63 67 -0.141090177094588

63 68 -0.311317272042355

63 69 -0.310142749894871

63 70 -0.187739759847583

63 71 0.0719686910281918

63 72 0.208224417472335

63 73 0.381401632249174

63 74 0.433306586361190

63 75 0.368942367956450

63 76 0.340574699486289

64 1 0.406974482749447

64 2 0.525437930286194

64 3 0.456262744491631

64 4 0.279611877057860

64 5 -0.0577330685436099

64 6 -0.187831930691736

64 7 -0.202897789925781

64 8 -0.196860916973120

64 9 -0.228211655520833

64 10 -0.185619399027195

64 11 -0.201505727696206

64 12 -0.0735998405814080

64 13 -0.0515983953466460

64 14 0.162413136425149

64 15 0.169529588324651

64 16 0.272981145565360

64 17 0.167048071985887

64 18 0.151779573178539

64 19 0.102123109331591

64 20 0.00819459456355705

64 21 -0.104223950774917

64 22 -0.287183228154176

64 23 -0.355962821553829

64 24 -0.385929364913907

64 25 -0.335896967792202

64 26 -0.336163374252965

64 27 -0.381822244099503

64 28 -0.376786633562594

64 29 -0.350920887113012

64 30 -0.346173326764697

64 31 -0.331155551068222

64 32 -0.315882679920005

64 33 -0.274814310939128

64 34 -0.253054862885615

64 35 -0.274711003616016

64 36 -0.270516749592933

64 37 -0.258616608916818

64 38 -0.264465043545828

64 39 -0.185261107901256

64 40 -0.219930184323751

64 41 -0.290940053125525

64 42 -0.190739692170995

64 43 -0.248670592313454

64 44 -0.250415699143625

64 45 -0.219917028675633

64 46 -0.192349952177306

64 47 -0.204697592015909

64 48 -0.222758961142731

64 49 -0.272916171381225

64 50 -0.309390196272611

64 51 -0.342980619617530

64 52 -0.370584883197624

64 53 -0.352749568302450

64 54 -0.294563872209241

64 55 -0.175196363557710

64 56 -0.0596032991112392

64 57 0.0459465894459026

64 58 -0.0344584595133307

64 59 0.00128932204416753

64 60 0.136349662668436

64 61 0.201549002923352

64 62 0.318477333209766

64 63 0.666898291871249

64 64 1.00000000000000

64 65 0.649356730870369

64 66 0.298095484004358

64 67 -0.00209576494292355

64 68 -0.209772596779237

64 69 -0.266631254205127

64 70 -0.168213544478517

64 71 0.0129914165462965

64 72 0.223868906336088

64 73 0.335657075306545

64 74 0.423422826718321

64 75 0.331503346380584

64 76 0.296014705805021

65 1 0.166230911135789

65 2 0.231289013165045

65 3 0.340967311968212

65 4 0.309469141018632

65 5 0.0771284349476222

65 6 -0.0150792355818286

65 7 -0.0875014018000768

65 8 -0.0772353446804318

65 9 -0.126880494061276

65 10 -0.104561782758636

65 11 -0.137465190128512

65 12 -0.0435226146200891

65 13 -0.0445475994820903

65 14 0.0632619199446873

65 15 0.0500919920697234

65 16 0.0497634119905790

65 17 0.0170650503716018

65 18 0.0336421815136299

65 19 0.0658561397867725

65 20 0.0142855706153346

65 21 -0.0835105352151812

65 22 -0.176600144354669

65 23 -0.217718729703193

65 24 -0.234373066798925

65 25 -0.246915762370679

65 26 -0.241297859279174

65 27 -0.245332479740221

65 28 -0.254582239464280

65 29 -0.240504744507453

65 30 -0.233942614164689

65 31 -0.223261671798581

65 32 -0.212260561190090

65 33 -0.181660323649639

65 34 -0.177218302802421

65 35 -0.203178257162292

65 36 -0.197169012176684

65 37 -0.170634798565934

65 38 -0.164330016526923

65 39 -0.121087121679245

65 40 -0.145896394199504

65 41 -0.169155420902817

65 42 -0.113770157169213

65 43 -0.139393481225874

65 44 -0.114467065808787

65 45 -0.0735106931735103

65 46 -0.0988379779727188

65 47 -0.134700844565960

65 48 -0.154153881946913

65 49 -0.163864366928067

65 50 -0.165884737584896

65 51 -0.151226286621975

65 52 -0.201390071182073

65 53 -0.161639559454469

65 54 -0.106670551056279

65 55 -0.0361302757508555

65 56 0.0168407707579591

65 57 0.0815215143946897

65 58 0.0556391214639162

65 59 0.0743760606850061

65 60 0.140026416262892

65 61 0.210730826204034

65 62 0.326430774169992

65 63 0.430243200058957

65 64 0.649356730870369

65 65 1.00000000000000

65 66 0.530737328549279

65 67 0.215628949421452

65 68 -0.0255198643407237

65 69 -0.115859272497135

65 70 -0.109393270090147

65 71 -0.00415864742916292

65 72 0.0690324301778245

65 73 0.166241684107963

65 74 0.222802734661980

65 75 0.218303225826642

65 76 0.176441100521349

66 1 -0.0834506195366055

66 2 0.0236545498989181

66 3 0.253883943786878

66 4 0.510433179021048

66 5 0.368634432105665

66 6 0.279408884259847

66 7 0.0853182234968921

66 8 -0.0118769240248881

66 9 -0.0298737540068605

66 10 -0.0540596846564171

66 11 0.0816172225191489

66 12 0.149735748633964

66 13 0.163392157096818

66 14 0.161975819193277

66 15 0.0859311881875174

66 16 -0.0589710376347938

66 17 -0.107447620925034

66 18 -0.172670499107002

66 19 -0.169589696770550

66 20 -0.190157613806288

66 21 -0.184774503784311

66 22 -0.165404556179174

66 23 -0.0995671943756740

66 24 -0.0590774857483694

66 25 -0.102302265575810

66 26 -0.0723716672955232

66 27 -0.00261561683630440

66 28 -0.00340261183588792

66 29 -0.0134305073300547

66 30 0.00894549063923464

66 31 0.0304427903914681

66 32 0.0137057493324553

66 33 0.00805641876225787

66 34 0.0218485409762073

66 35 0.0150841831224817

66 36 0.0177103036652216

66 37 0.0362023523930931

66 38 0.0330416748210204

66 39 0.0267432263477542

66 40 0.00459926908987819

66 41 -0.000192743286376390

66 42 -0.000617034352731147

66 43 0.0450817139591625

66 44 0.0833895756957986

66 45 0.108092851688972

66 46 -0.00485493832314746

66 47 -0.0565109645254161

66 48 -0.0127122348797714

66 49 0.0305874749893966

66 50 0.0222024476058025

66 51 -4.74598935370602e-05

66 52 -0.0426120013231203

66 53 -0.0669488839198843

66 54 -0.100051301684531

66 55 -0.135090098446377

66 56 -0.148325616148237

66 57 -0.162869358134448

66 58 -0.122440380503108

66 59 -0.0747726772068586

66 60 -0.0980490039561699

66 61 -0.0431266560735218

66 62 0.0139278231349654

66 63 0.0999258143004004

66 64 0.298095484004358

66 65 0.530737328549279

66 66 0.999999999999997

66 67 0.656400900493836

66 68 0.281585945727982

66 69 0.0221491824719933

66 70 -0.0784496928461266

66 71 -0.141784623579797

66 72 -0.0950030845637116

66 73 -0.0960389344248985

66 74 -0.0198045612753851

66 75 -0.0232187494462630

66 76 -0.00212015869134387

67 1 -0.229677588303832

67 2 -0.139487355681989

67 3 0.103379837151305

67 4 0.371464012878895

67 5 0.512832206473162

67 6 0.404364637428578

67 7 0.170123687646175

67 8 0.0746864864453519

67 9 0.0377343521328912

67 10 -0.00769686709595837

67 11 0.100341577740619

67 12 0.120265819638336

67 13 0.160824534145214

67 14 0.0417640148207787

67 15 0.00954372890591192

67 16 -0.169152281003441

67 17 -0.184907922913075

67 18 -0.233407218563698

67 19 -0.221255535675832

67 20 -0.189161454963075

67 21 -0.142123324153424

67 22 -0.0345537698097479

67 23 0.0655926779024701

67 24 0.111116995975054

67 25 0.0400218596615111

67 26 0.0730451972213870

67 27 0.175336835382723

67 28 0.167065356522748

67 29 0.125751232547976

67 30 0.151699229131910

67 31 0.172355996244615

67 32 0.133797305398026

67 33 0.0975453665444460

67 34 0.107858475547527

67 35 0.127813601813422

67 36 0.140534035133030

67 37 0.167081386384830

67 38 0.166155107721515

67 39 0.127949584793789

67 40 0.135446931835951

67 41 0.184670313529397

67 42 0.147892861383327

67 43 0.280834708396338

67 44 0.346302154979126

67 45 0.314797677931796

67 46 0.156079090440068

67 47 0.118948296356714

67 48 0.137403765493832

67 49 0.211076365346155

67 50 0.208755484998425

67 51 0.185658897679212

67 52 0.143396093266720

67 53 0.109803931242701

67 54 0.0486398676439365

67 55 -0.0536065074773991

67 56 -0.125497021099054

67 57 -0.180572595925184

67 58 -0.0887474154063696

67 59 -0.0399516448085359

67 60 -0.128380784380342

67 61 -0.0994430948105630

67 62 -0.121080409090253

67 63 -0.141090177094588

67 64 -0.00209576494292355

67 65 0.215628949421452

67 66 0.656400900493836

67 67 0.999999999999998

67 68 0.614787220921969

67 69 0.314903895475112

67 70 0.110877901421842

67 71 -0.0803590816751026

67 72 -0.241070195762881

67 73 -0.286446451636521

67 74 -0.269021434208789

67 75 -0.240665642656940

67 76 -0.210314686085992

68 1 -0.378391502109759

68 2 -0.306943533183460

68 3 -0.132644802498409

68 4 0.118214455241313

68 5 0.415372490701421

68 6 0.508901861291459

68 7 0.470849021183741

68 8 0.383053624566501

68 9 0.326026452876261

68 10 0.162716416966411

68 11 0.230342068799503

68 12 0.112599495348414

68 13 0.160680820727156

68 14 -0.0507164852138570

68 15 -0.0747039893479449

68 16 -0.266286041769471

68 17 -0.272096143169662

68 18 -0.325684504643051

68 19 -0.306253607624683

68 20 -0.239354741699562

68 21 -0.137587852051995

68 22 0.0396502374125482

68 23 0.185947304339208

68 24 0.254411516635297

68 25 0.161442712520656

68 26 0.199984069225918

68 27 0.344655501289415

68 28 0.327568212226708

68 29 0.250570118423323

68 30 0.273856649102763

68 31 0.292325927303398

68 32 0.230200147559856

68 33 0.160363745283457

68 34 0.166267150945987

68 35 0.204718570164481

68 36 0.227834419687160

68 37 0.281297533610163

68 38 0.333013746552119

68 39 0.287220918288495

68 40 0.251541996944905

68 41 0.370286738349616

68 42 0.376632943556655

68 43 0.515666637069568

68 44 0.562464089785479

68 45 0.396276240700876

68 46 0.225003688970216

68 47 0.228582066328218

68 48 0.208403728589775

68 49 0.285559200672106

68 50 0.259589663263156

68 51 0.218693163385564

68 52 0.191865501735091

68 53 0.182245526310764

68 54 0.0987208730904533

68 55 -0.0526089909055146

68 56 -0.168005750136485

68 57 -0.242236220594742

68 58 -0.129801950554370

68 59 -0.0988163816360776

68 60 -0.203923279922078

68 61 -0.213741172029079

68 62 -0.264263425824135

68 63 -0.311317272042355

68 64 -0.209772596779237

68 65 -0.0255198643407237

68 66 0.281585945727982

68 67 0.614787220921969

68 68 0.999999999999999

68 69 0.591542790412902

68 70 0.224823737720807

68 71 -0.179882357236225

68 72 -0.327896513862204

68 73 -0.497854079381169

68 74 -0.481764749443887

68 75 -0.425169109306286

68 76 -0.370586372128565

69 1 -0.307003216291648

69 2 -0.294763293909597

69 3 -0.206959799950345

69 4 -0.0590512835590032

69 5 0.257102576478082

69 6 0.349185906652356

69 7 0.280156886806839

69 8 0.247065133458111

69 9 0.204622189978389

69 10 0.168461452728832

69 11 0.135741313945653

69 12 0.0486985039130619

69 13 0.0152237305870807

69 14 -0.137204573269290

69 15 -0.132555921793973

69 16 -0.239611726292246

69 17 -0.211498313847438

69 18 -0.222555492932657

69 19 -0.188953805317887

69 20 -0.104785302906728

69 21 -0.0250449289470036

69 22 0.130864281675678

69 23 0.219561303211018

69 24 0.249374863739258

69 25 0.187804588772713

69 26 0.199988801939665

69 27 0.276944719576107

69 28 0.248345595756556

69 29 0.194998716756830

69 30 0.193602883031852

69 31 0.190590205005083

69 32 0.143559050412200

69 33 0.0779416589750166

69 34 0.0693125926853708

69 35 0.142214892792974

69 36 0.165132396148443

69 37 0.209402780289304

69 38 0.261846167693757

69 39 0.221896654176097

69 40 0.286702886640803

69 41 0.507732060677229

69 42 0.530502497263961

69 43 0.556567910129294

69 44 0.457323036064344

69 45 0.262334971687413

69 46 0.110757510754172

69 47 0.157750615248664

69 48 0.179715615731639

69 49 0.283497531874798

69 50 0.294897152508701

69 51 0.299622900657755

69 52 0.284659711271535

69 53 0.240529594341223

69 54 0.181450192783650

69 55 0.0479556334353272

69 56 -0.0637384957970534

69 57 -0.122052352854214

69 58 -0.0190081785795026

69 59 -0.0217054276998655

69 60 -0.145781047817564

69 61 -0.175882194043350

69 62 -0.241123548316325

69 63 -0.310142749894871

69 64 -0.266631254205127

69 65 -0.115859272497135

69 66 0.0221491824719933

69 67 0.314903895475112

69 68 0.591542790412902

69 69 0.999999999999999

69 70 0.684775122582839

69 71 0.255824251221827

69 72 -0.136802069160227

69 73 -0.343610930227433

69 74 -0.450405560555684

69 75 -0.383103716971026

69 76 -0.399724257400343

70 1 -0.155490371870077

70 2 -0.152489834494178

70 3 -0.138437473909594

70 4 -0.0850647455188047

70 5 0.0839589413004064

70 6 0.105589668894192

70 7 0.0344068064332221

70 8 0.000489556794605003

70 9 -0.00643161080905931

70 10 0.0434923955188294

70 11 0.0238325660301289

70 12 0.0209676416438523

70 13 -0.0351298054502622

70 14 -0.0776320360106196

70 15 -0.0891894672215930

70 16 -0.130799286266176

70 17 -0.118824803176950

70 18 -0.121669804197314

70 19 -0.109705436006144

70 20 -0.0825612912725899

70 21 -0.0357044751056152

70 22 0.0332888446318207

70 23 0.0696750916329347

70 24 0.0691057547704728

70 25 0.0599717895949661

70 26 0.0553555071972352

70 27 0.0746531612102820

70 28 0.0696000469566635

70 29 0.0609901270767567

70 30 0.0634728806263709

70 31 0.0627958287556572

70 32 0.0391970339592866

70 33 0.00515985620736785

70 34 0.0129856597364904

70 35 0.0874532286447295

70 36 0.120216377148698

70 37 0.112794456996240

70 38 0.121037183242287

70 39 0.111945031833912

70 40 0.293538792040585

70 41 0.479555572372178

70 42 0.570685550400857

70 43 0.471980967807838

70 44 0.309611775637534

70 45 0.102016777215520

70 46 0.000342334026896806

70 47 0.0801388342066785

70 48 0.136193671578227

70 49 0.238397258179978

70 50 0.227168963761151

70 51 0.209508682383401

70 52 0.164859534999048

70 53 0.0907192952663549

70 54 0.0624739232875274

70 55 -0.0147593531057074

70 56 -0.0551719987362384

70 57 -0.0800169683662577

70 58 -0.0269833569308683

70 59 0.00165341140489927

70 60 -0.0751677734960627

70 61 -0.0805711925140545

70 62 -0.138282112804547

70 63 -0.187739759847583

70 64 -0.168213544478517

70 65 -0.109393270090147

70 66 -0.0784496928461266

70 67 0.110877901421842

70 68 0.224823737720807

70 69 0.684775122582839

70 70 0.999999999999999

70 71 0.562507201082940

70 72 0.132589140554860

70 73 -0.113182480513893

70 74 -0.293268983077672

70 75 -0.249806190703239

70 76 -0.304998875433613

71 1 0.0784434542165613

71 2 0.0207992246948877

71 3 -0.0323621992296731

71 4 -0.165576592728672

71 5 -0.201459557104158

71 6 -0.234271183505370

71 7 -0.381626158576023

71 8 -0.302493128472257

71 9 -0.337844262872113

71 10 -0.146866733918188

71 11 -0.305206380558169

71 12 -0.185489894709651

71 13 -0.274407470309025

71 14 -0.178536334741417

71 15 -0.119287321069909

71 16 -0.0379226682402305

71 17 0.0275795224139962

71 18 0.112438793325404

71 19 0.157458870884151

71 20 0.162925826185504

71 21 0.114775392701083

71 22 0.0772948310224640

71 23 -0.0107054369609165

71 24 -0.0867318659306034

71 25 -0.0691924464898569

71 26 -0.0876190285806787

71 27 -0.144061594832386

71 28 -0.168872645964311

71 29 -0.160595435162264

71 30 -0.146686207055315

71 31 -0.146668771251618

71 32 -0.159510110941039

71 33 -0.156973712984313

71 34 -0.126483100336880

71 35 -0.0511348332987561

71 36 -0.00979275331132751

71 37 -0.0131421188611691

71 38 -0.0530553046782482

71 39 0.0170784948921016

71 40 0.248661374188834

71 41 0.241716620887159

71 42 0.176617742715676

71 43 0.117774737133830

71 44 0.0243365590518978

71 45 -0.0449681113037973

71 46 -0.0813238174549948

71 47 -0.0209552511409071

71 48 0.0139201281843830

71 49 0.0879930962141399

71 50 0.116235169866571

71 51 0.149897024423254

71 52 0.118765765888168

71 53 0.0793198472527346

71 54 0.126364286044901

71 55 0.147883272805084

71 56 0.160565074555230

71 57 0.187008177017283

71 58 0.187864156309492

71 59 0.171607898163296

71 60 0.139346939092420

71 61 0.127624059181368

71 62 0.0878000554555245

71 63 0.0719686910281918

71 64 0.0129914165462965

71 65 -0.00415864742916292

71 66 -0.141784623579797

71 67 -0.0803590816751026

71 68 -0.179882357236225

71 69 0.255824251221827

71 70 0.562507201082940

71 71 0.999999999999996

71 72 0.434262459245783

71 73 0.296200084743668

71 74 0.0570643774365612

71 75 0.0104681703467547

71 76 -0.129515539698986

72 1 0.262599997801985

72 2 0.300289497303322

72 3 0.153802148411551

72 4 0.0768834199668973

72 5 -0.110918030698283

72 6 -0.197869639675652

72 7 -0.134671260712336

72 8 -0.177840423648168

72 9 -0.153501735866093

72 10 -0.0427778157988669

72 11 -0.0572132080547358

72 12 0.0491457845391648

72 13 -0.0114271127673640

72 14 0.182400390132313

72 15 0.118622358715248

72 16 0.194749327024355

72 17 0.107028031855384

72 18 0.0936211638764671

72 19 0.00418560334829195

72 20 -0.0945008660782621

72 21 -0.172396410313346

72 22 -0.388752129235186

72 23 -0.476555420032722

72 24 -0.453637881449400

72 25 -0.347825998445293

72 26 -0.363035432648226

72 27 -0.423228852898357

72 28 -0.393473869197198

72 29 -0.298530660078405

72 30 -0.268959990792777

72 31 -0.306687345708735

72 32 -0.248221116308445

72 33 -0.152414322173148

72 34 -0.136430476429279

72 35 -0.172985308236961

72 36 -0.195381940839282

72 37 -0.292046433719086

72 38 -0.336010189641966

72 39 -0.252693520177268

72 40 -0.0767149930271502

72 41 -0.0814743650731793

72 42 0.0700579036442376

72 43 -0.161814273709263

72 44 -0.318858143251214

72 45 -0.362492187175526

72 46 -0.317094565620223

72 47 -0.281514167088690

72 48 -0.253775667954240

72 49 -0.344794559192357

72 50 -0.384069845846595

72 51 -0.439442058825288

72 52 -0.507049703691096

72 53 -0.520221081884486

72 54 -0.473266882240511

72 55 -0.347862458413672

72 56 -0.230338967429810

72 57 -0.111844652215545

72 58 -0.244131673711455

72 59 -0.225150216998746

72 60 -0.0532356381325968

72 61 -0.00847291264442932

72 62 0.116420277972036

72 63 0.208224417472335

72 64 0.223868906336088

72 65 0.0690324301778245

72 66 -0.0950030845637116

72 67 -0.241070195762881

72 68 -0.327896513862204

72 69 -0.136802069160227

72 70 0.132589140554860

72 71 0.434262459245783

72 72 0.999999999999980

72 73 0.665044353827483

72 74 0.477397036408592

72 75 0.356451425332582

72 76 0.241437455624174

73 1 0.399877569526604

73 2 0.365754622146734

73 3 0.207940927738745

73 4 0.0116800741317465

73 5 -0.257354749519812

73 6 -0.380943144122360

73 7 -0.382347388520186

73 8 -0.349278122356397

73 9 -0.350585875927812

73 10 -0.182810100980987

73 11 -0.268191930741332

73 12 -0.108618392004671

73 13 -0.154469008285481

73 14 0.0565955916061417

73 15 0.0488964482539580

73 16 0.230304233673194

73 17 0.181394451821128

73 18 0.258610762689793

73 19 0.217887984079161

73 20 0.115595879877636

73 21 -0.0370085057286659

73 22 -0.270631774496430

73 23 -0.454861492335585

73 24 -0.493977167753139

73 25 -0.367531349499776

73 26 -0.397991705666661

73 27 -0.566003427637744

73 28 -0.536233092785483

73 29 -0.379356357694027

73 30 -0.424432884423213

73 31 -0.507015836710536

73 32 -0.370806877381930

73 33 -0.248203014171326

73 34 -0.299211649686437

73 35 -0.373777847780611

73 36 -0.423693043705553

73 37 -0.525102823455134

73 38 -0.582119632072821

73 39 -0.525396654459772

73 40 -0.416441990082385

73 41 -0.408468547761672

73 42 -0.275505691574189

73 43 -0.357249600459721

73 44 -0.425258666908881

73 45 -0.363713824355253

73 46 -0.299865776182131

73 47 -0.309816274269156

73 48 -0.298254691351336

73 49 -0.379050018464919

73 50 -0.376602209367456

73 51 -0.367229731823876

73 52 -0.428919879711488

73 53 -0.425064242341143

73 54 -0.323874706904717

73 55 -0.147555044772679

73 56 -0.0142105569644225

73 57 0.120186609158297

73 58 -0.0251463106986951

73 59 -0.0368817951533310

73 60 0.140894594192938

73 61 0.169640819950550

73 62 0.275210839228530

73 63 0.381401632249174

73 64 0.335657075306545

73 65 0.166241684107963

73 66 -0.0960389344248985

73 67 -0.286446451636521

73 68 -0.497854079381169

73 69 -0.343610930227433

73 70 -0.113182480513893

73 71 0.296200084743668

73 72 0.665044353827483

73 73 1.00000000000000

73 74 0.772187617177159

73 75 0.600776734220406

73 76 0.435541854356183

74 1 0.419872618637050

74 2 0.423218359567439

74 3 0.263914716349675

74 4 0.0882060877966419

74 5 -0.226003658416131

74 6 -0.302422107042951

74 7 -0.313483013846974

74 8 -0.268518653079965

74 9 -0.270266037981862

74 10 -0.124313255123062

74 11 -0.206883188516317

74 12 -0.0551981193885046

74 13 -0.123856449295456

74 14 0.0939727491058876

74 15 0.0730692787116598

74 16 0.241877813060940

74 17 0.161457379731408

74 18 0.222348609486491

74 19 0.188018186112703

74 20 0.0540473428950319

74 21 -0.126794034067669

74 22 -0.386813287590730

74 23 -0.547075675655626

74 24 -0.602635886131525

74 25 -0.475637731707361

74 26 -0.462706544043890

74 27 -0.638276606218651

74 28 -0.615892989407330

74 29 -0.432948533262929

74 30 -0.465065792235904

74 31 -0.549924896331721

74 32 -0.406130117571241

74 33 -0.268435468923779

74 34 -0.315163862918776

74 35 -0.418319608421383

74 36 -0.476357297692809

74 37 -0.564979601408302

74 38 -0.608706064337309

74 39 -0.544202094200032

74 40 -0.524348329256186

74 41 -0.562787681886922

74 42 -0.424528682593624

74 43 -0.500628187645780

74 44 -0.504012630686129

74 45 -0.391823932284394

74 46 -0.290428220171593

74 47 -0.334848995092675

74 48 -0.382504172963010

74 49 -0.498323962032473

74 50 -0.507434477515846

74 51 -0.524913514459773

74 52 -0.564621415908717

74 53 -0.532170008256818

74 54 -0.429025261320511

74 55 -0.233492555385330

74 56 -0.0712490848006482

74 57 0.0967155461915984

74 58 -0.0638149659058881

74 59 -0.0775044134601471

74 60 0.133184153738737

74 61 0.156258355566074

74 62 0.287913497256985

74 63 0.433306586361190

74 64 0.423422826718321

74 65 0.222802734661980

74 66 -0.0198045612753851

74 67 -0.269021434208789

74 68 -0.481764749443887

74 69 -0.450405560555684

74 70 -0.293268983077672

74 71 0.0570643774365612

74 72 0.477397036408592

74 73 0.772187617177159

74 74 1.00000000000000

74 75 0.770843611765540

74 76 0.621990176424117

75 1 0.382063211096171

75 2 0.369500529198289

75 3 0.229129345121530

75 4 0.0750597399637932

75 5 -0.187099774468439

75 6 -0.264766052064126

75 7 -0.255910790979713

75 8 -0.221790195908086

75 9 -0.216546490437348

75 10 -0.109892939739919

75 11 -0.161926345208274

75 12 -0.0379409132272376

75 13 -0.0849731780136107

75 14 0.107363423604941

75 15 0.0964701206149820

75 16 0.246450267539318

75 17 0.186872323684644

75 18 0.227203424595051

75 19 0.177845130802452

75 20 0.0703007081987268

75 21 -0.0509311207818949

75 22 -0.287807924164011

75 23 -0.431303455238524

75 24 -0.462939550530311

75 25 -0.350371299272865

75 26 -0.365704900601254

75 27 -0.515401921429093

75 28 -0.481422302052785

75 29 -0.330909105360602

75 30 -0.380959537259701

75 31 -0.449255227427557

75 32 -0.320875121766732

75 33 -0.198347054412937

75 34 -0.251120385044360

75 35 -0.346792303137252

75 36 -0.405990605064888

75 37 -0.479460917174077

75 38 -0.516901230436706

75 39 -0.470031403635882

75 40 -0.454413075297964

75 41 -0.478009471190971

75 42 -0.368040306889995

75 43 -0.424197633417169

75 44 -0.442767361249281

75 45 -0.359198913699541

75 46 -0.246239896101871

75 47 -0.292598337499400

75 48 -0.351707697609511

75 49 -0.421999697703138

75 50 -0.416296030446046

75 51 -0.401307741590352

75 52 -0.436025944910655

75 53 -0.421278278297628

75 54 -0.341998493505801

75 55 -0.178024820051105

75 56 -0.0488405138628397

75 57 0.0816318662569893

75 58 -0.0428796563458465

75 59 -0.0669235490441791

75 60 0.0903842064008005

75 61 0.118648925102809

75 62 0.252111793297936

75 63 0.368942367956450

75 64 0.331503346380584

75 65 0.218303225826642

75 66 -0.0232187494462630

75 67 -0.240665642656940

75 68 -0.425169109306286

75 69 -0.383103716971026

75 70 -0.249806190703239

75 71 0.0104681703467547

75 72 0.356451425332582

75 73 0.600776734220406

75 74 0.770843611765540

75 75 1.00000000000001

75 76 0.838982933661132

76 1 0.345559441033392

76 2 0.337418423723743

76 3 0.213077320078042

76 4 0.0931191720011855

76 5 -0.150948455973533

76 6 -0.241312292598353

76 7 -0.219704542547352

76 8 -0.208377081966586

76 9 -0.189335441453008

76 10 -0.119724478541315

76 11 -0.113190260194091

76 12 0.00118467232102547

76 13 -0.0204111540213531

76 14 0.151171038604455

76 15 0.127647388930710

76 16 0.257294768274865

76 17 0.161028278979868

76 18 0.175644459592233

76 19 0.111096504471596

76 20 0.0149559382090580

76 21 -0.0787430804533212

76 22 -0.300147141735252

76 23 -0.406878607918758

76 24 -0.422724875212364

76 25 -0.313613414235443

76 26 -0.322008803657194

76 27 -0.444717428531649

76 28 -0.400227186159009

76 29 -0.266774570614938

76 30 -0.299485036975049

76 31 -0.349375612394482

76 32 -0.240957579930860

76 33 -0.136296666863710

76 34 -0.171206107471085

76 35 -0.260452289108000

76 36 -0.318613490924674

76 37 -0.388249759440332

76 38 -0.437782884635517

76 39 -0.401923096303141

76 40 -0.399487736062934

76 41 -0.459075605451169

76 42 -0.394079137566316

76 43 -0.424161510601670

76 44 -0.421842819125224

76 45 -0.311696320492405

76 46 -0.200320367358091

76 47 -0.260889392654575

76 48 -0.315897884868240

76 49 -0.404060714236497

76 50 -0.421042136380109

76 51 -0.420036177923829

76 52 -0.427670166682457

76 53 -0.421244341444612

76 54 -0.369261350406810

76 55 -0.221314970216677

76 56 -0.106451284087994

76 57 0.0120405505885534

76 58 -0.100279425700257

76 59 -0.120679531013528

76 60 0.0255409850992461

76 61 0.0591229044152939

76 62 0.208877260323272

76 63 0.340574699486289

76 64 0.296014705805021

76 65 0.176441100521349

76 66 -0.00212015869134387

76 67 -0.210314686085992

76 68 -0.370586372128565

76 69 -0.399724257400343

76 70 -0.304998875433613

76 71 -0.129515539698986

76 72 0.241437455624174

76 73 0.435541854356183

76 74 0.621990176424117

76 75 0.838982933661132

76 76 1.00000000000001

**Data for Figure 3 a and b**

The following data set is for drawing Fig. 3, the first two columns are residue indices, the third column are the values of entropy transfer from residue i to residue j at 5 ns.

1 1 0

1 2 0.000112800289299719

1 3 0.000775176571340408

1 4 0.000812074116184114

1 5 0.000823165564180273

1 6 0

1 7 0

1 8 0.00586818564862568

1 9 0.00403203562968202

1 10 0

1 11 0.000666906920697707

1 12 0.00149487267289927

1 13 0.00125658120423333

1 14 0

1 15 7.81814499241085e-05

1 16 0.000618229794947744

1 17 0

1 18 0.000117457096611484

1 19 0.000323797909951240

1 20 0

1 21 0

1 22 0

1 23 0

1 24 0

1 25 0

1 26 5.89061061905261e-05

1 27 0

1 28 0.000395087060607757

1 29 0.000320686510404733

1 30 0.000126699029303956

1 31 0.000406504669570928

1 32 0.000342747613949479

1 33 0

1 34 0

1 35 0

1 36 0.000725721650640887

1 37 0.000811963721309594

1 38 0.000952047467831452

1 39 0

1 40 0.00427006777845229

1 41 0.00242504695142931

1 42 0.00107810004620512

1 43 0.00188418661547707

1 44 0.00148849859972999

1 45 0.000830857744630764

1 46 0.00267059635185163

1 47 0.00343216551230707

1 48 0.00111736457458889

1 49 0.00318566100920703

1 50 0.00293057720496859

1 51 0.00132212115528874

1 52 0.000823358014741871

1 53 6.23984265915034e-05

1 54 0.000725958304327756

1 55 5.59515524967136e-05

1 56 0.00121037953246828

1 57 0.000753463873836457

1 58 0.00162928819940944

1 59 0.00261506389744870

1 60 0.00214388114875608

1 61 0.00136518263578989

1 62 0.000340218868728370

1 63 0.000796762487063951

1 64 0.000137339347893062

1 65 0.000439854539547868

1 66 9.45855842215959e-06

1 67 0

1 68 0

1 69 0.000211756426245602

1 70 0.00180279585724707

1 71 0.00186239924442233

1 72 0

1 73 0

1 74 0.00100607258752305

1 75 0.0132157848343639

1 76 0.0160818695926435

2 1 0.00224358551225301

2 2 0

2 3 0.00163936186405533

2 4 0.00201171387147436

2 5 0.00266498660365877

2 6 0.00100793296093304

2 7 0.00120317874074816

2 8 0.00773057147586531

2 9 0.00588101931004104

2 10 0.00152992432244670

2 11 0.00245021761413533

2 12 0.00316795621983657

2 13 0.00266555218298559

2 14 0

2 15 0.00187714931857474

2 16 0.00255186567689725

2 17 0.00148594612287034

2 18 0.00197109525780081

2 19 0.00219631989574409

2 20 0.00123946509470363

2 21 0.00119627396927913

2 22 0.00137697313817964

2 23 0.00149437533128038

2 24 0.00175534690830614

2 25 0.00176305171694602

2 26 0.00207655773306670

2 27 0.00157392280641255

2 28 0.00247439474441435

2 29 0.00221199956851026

2 30 0.00202033071809571

2 31 0.00231954200646367

2 32 0.00220054130536262

2 33 0.00150865957720925

2 34 0.00104461327081329

2 35 0.00167393775461921

2 36 0.00258692124286997

2 37 0.00260332298026950

2 38 0.00273247373756302

2 39 0.00179760394275419

2 40 0.00635886673976727

2 41 0.00419531803388196

2 42 0.00288380147921230

2 43 0.00370938158869416

2 44 0.00328902600633041

2 45 0.00280332190045796

2 46 0.00462301040488355

2 47 0.00525932085759751

2 48 0.00289772638883556

2 49 0.00491191257079549

2 50 0.00455316810361361

2 51 0.00286587997430854

2 52 0.00294193869072590

2 53 0.00190133042644902

2 54 0.00256572515951814

2 55 0.00222644748160850

2 56 0.00330328441308403

2 57 0.00283358610484452

2 58 0.00339703712635164

2 59 0.00441577488318234

2 60 0.00392038386851801

2 61 0.00328977592791235

2 62 0.00221251798718125

2 63 0.00241083689161759

2 64 0.00157730369451237

2 65 0.00227650683713748

2 66 0.00182036218275505

2 67 0.00142598346367706

2 68 0.00168466461589512

2 69 0.00208188232936446

2 70 0.00362281869743797

2 71 0.00372823151933732

2 72 0

2 73 0.000421692451299971

2 74 0.00343205505630151

2 75 0.0147903174140758

2 76 0.0175254892419352

3 1 0.00765489098394889

3 2 0.00650782271193640

3 3 0

3 4 0.00386637402227286

3 5 0.00737288824889004

3 6 0.00590579187993134

3 7 0.00627369731904826

3 8 0.0128860012982583

3 9 0.0110201648920232

3 10 0.00666001615298062

3 11 0.00733873972720345

3 12 0.00793709982658181

3 13 0.00712481100786000

3 14 0.00350805919779496

3 15 0.00733057934093540

3 16 0.00798322873148372

3 17 0.00680608376863812

3 18 0.00722069611031273

3 19 0.00733556863418583

3 20 0.00611798030148425

3 21 0.00603601961262745

3 22 0.00589705377620942

3 23 0.00601152959804341

3 24 0.00618547914050993

3 25 0.00677565857498907

3 26 0.00709219233361036

3 27 0.00629720962011637

3 28 0.00737978458930466

3 29 0.00730277112540112

3 30 0.00708186874599204

3 31 0.00747599026389556

3 32 0.00726323944877216

3 33 0.00653060715095410

3 34 0.00607189436548050

3 35 0.00677500363731876

3 36 0.00774423552310938

3 37 0.00769182494068904

3 38 0.00775090922200988

3 39 0.00694228005596764

3 40 0.0123132390096428

3 41 0.00934973658805782

3 42 0.00778764000449872

3 43 0.00859897228560547

3 44 0.00823426040956998

3 45 0.00780029747809485

3 46 0.00960056995845060

3 47 0.0104112258921425

3 48 0.00794564831728828

3 49 0.00976145619229107

3 50 0.00948396504132976

3 51 0.00759464184593750

3 52 0.00783875512562338

3 53 0.00707091316718267

3 54 0.00762663576337808

3 55 0.00701138147142411

3 56 0.00816770964742408

3 57 0.00771006102617000

3 58 0.00861634213862683

3 59 0.00950084284080710

3 60 0.00909357929671328

3 61 0.00841876164352751

3 62 0.00740151049378623

3 63 0.00764869820670988

3 64 0.00671270911019306

3 65 0.00733697829114988

3 66 0.00702957737976817

3 67 0.00646431137640535

3 68 0.00673040715213191

3 69 0.00711459251005087

3 70 0.00863168302688200

3 71 0.00894214889167633

3 72 0.00495850948389265

3 73 0.00544131106228907

3 74 0.00781004475520930

3 75 0.0199109689387378

3 76 0.0226526286792716

4 1 0.0101020414226622

4 2 0.00939119450296477

4 3 0.00709762804623071

4 4 0

4 5 0.00961465281470619

4 6 0.00786102738109129

4 7 0.00855743195751668

4 8 0.0152851088162689

4 9 0.0133756904954891

4 10 0.00914693113788001

4 11 0.00959817225183657

4 12 0.0102248230498495

4 13 0.00975764706055204

4 14 0.00627263174591242

4 15 0.00956369624079412

4 16 0.0103225541840537

4 17 0.00926775843761696

4 18 0.00965474756202556

4 19 0.00974799728015757

4 20 0.00842216472415491

4 21 0.00837747046685422

4 22 0.00842565719395771

4 23 0.00879632926602092

4 24 0.00882861210524433

4 25 0.00918337404505298

4 26 0.00939968510037759

4 27 0.00851007019432848

4 28 0.00975452393366150

4 29 0.00961861162154232

4 30 0.00937298388937324

4 31 0.00987656242046953

4 32 0.00962283329830915

4 33 0.00910413416283118

4 34 0.00859109872213437

4 35 0.00910843458963140

4 36 0.0101625743502899

4 37 0.0100404000667085

4 38 0.0101002951164190

4 39 0.00979527269870162

4 40 0.0159579262775258

4 41 0.0118322967130031

4 42 0.0102218636533835

4 43 0.0109781088730424

4 44 0.0105824547690282

4 45 0.0101097241732109

4 46 0.0119009022037575

4 47 0.0128162224799860

4 48 0.0103320488818307

4 49 0.0120695376680195

4 50 0.0117980915403169

4 51 0.0102709566490790

4 52 0.0107734422222443

4 53 0.00993674717297766

4 54 0.0104341404955177

4 55 0.00943425290769651

4 56 0.0105647658889149

4 57 0.0101255286764022

4 58 0.0109167971883181

4 59 0.0119491148843464

4 60 0.0115676870220804

4 61 0.0108627484092561

4 62 0.00983206483612686

4 63 0.0103931861902735

4 64 0.0101937118009273

4 65 0.00982122675368186

4 66 0.00960971426921453

4 67 0.00889567021279303

4 68 0.00912922855098108

4 69 0.00955560140735545

4 70 0.0109063440910111

4 71 0.0115058056112315

4 72 0.00740503319028085

4 73 0.00779567545634652

4 74 0.0104285001687345

4 75 0.0229040584942084

4 76 0.0256474870800147

5 1 0.00414090829559655

5 2 0.00387458850733580

5 3 0.00373446749121009

5 4 0.00310544209905950

5 5 0

5 6 0.00137676957382737

5 7 0.00227415169034084

5 8 0.00900881231595840

5 9 0.00704999981803611

5 10 0.00292720391610846

5 11 0.00354247466880453

5 12 0.00433797412900638

5 13 0.00441183038443427

5 14 0.00153517518320456

5 15 0.00352399051141727

5 16 0.00382790616988626

5 17 0.00262973699897240

5 18 0.00321590539032623

5 19 0.00351940175799481

5 20 0.00213791288065512

5 21 0.00207996916508535

5 22 0.00176716347274808

5 23 0.00196275648642907

5 24 0.00197027663963989

5 25 0.00288838321851403

5 26 0.00306075125033123

5 27 0.00204904634377989

5 28 0.00343718095769086

5 29 0.00333471272841301

5 30 0.00309690627854409

5 31 0.00350457237784718

5 32 0.00333637101313089

5 33 0.00264079836060116

5 34 0.00200744084737514

5 35 0.00281671358748625

5 36 0.00390273623720866

5 37 0.00381255838937356

5 38 0.00388453784966497

5 39 0.00243422038799856

5 40 0.00796036352192941

5 41 0.00565048198334839

5 42 0.00393674518082554

5 43 0.00485962528753592

5 44 0.00430497765787496

5 45 0.00380313888627115

5 46 0.00552772209729369

5 47 0.00644645716651116

5 48 0.00408198159870143

5 49 0.00593067084543819

5 50 0.00576530082958326

5 51 0.00412720269309519

5 52 0.00344411171145698

5 53 0.00245840649806617

5 54 0.00311853411273466

5 55 0.00276092881244550

5 56 0.00433894100849230

5 57 0.00384374183113634

5 58 0.00447429874271865

5 59 0.00572810389150735

5 60 0.00525721554117609

5 61 0.00460536389862987

5 62 0.00359491807888024

5 63 0.00430186546461664

5 64 0.00398690011139780

5 65 0.00362618486632799

5 66 0.00324986992418053

5 67 0.00276735783950988

5 68 0.00295277614138612

5 69 0.00326497421875094

5 70 0.00469542399159195

5 71 0.00510534873712953

5 72 0.000813000572265077

5 73 0.00129700019056300

5 74 0.00382374079554304

5 75 0.0159631774182791

5 76 0.0183421578096501

6 1 0.00484059399484793

6 2 0.00476751099502049

6 3 0.00471094862091803

6 4 0.00402394582746035

6 5 0.00357210577245914

6 6 0

6 7 0.00218406265571525

6 8 0.00963622946535103

6 9 0.00758641997018539

6 10 0.00328939292681285

6 11 0.00389445245106146

6 12 0.00479600147972759

6 13 0.00519612997673280

6 14 0.00239673060819612

6 15 0.00410936774577086

6 16 0.00435836536142409

6 17 0.00304878387994079

6 18 0.00362253555150216

6 19 0.00394437942450843

6 20 0.00259394090315057

6 21 0.00255016708002020

6 22 0.00225337333842490

6 23 0.00233534412268321

6 24 0.00242840694994184

6 25 0.00329313850433532

6 26 0.00345248296003309

6 27 0.00250508542181493

6 28 0.00384497203676437

6 29 0.00377217751777781

6 30 0.00349964701552896

6 31 0.00385950013987879

6 32 0.00377089975438183

6 33 0.00289643280741114

6 34 0.00232310135434077

6 35 0.00317482737799357

6 36 0.00429686028210824

6 37 0.00441796080278040

6 38 0.00445869599218213

6 39 0.00260240384045551

6 40 0.00786729941051345

6 41 0.00606368651520917

6 42 0.00445189646983268

6 43 0.00528040521688344

6 44 0.00495395972416457

6 45 0.00460880807714570

6 46 0.00609683711742037

6 47 0.00694865871510608

6 48 0.00454194865100999

6 49 0.00638956697611615

6 50 0.00622460719731965

6 51 0.00477138448226755

6 52 0.00422139468235883

6 53 0.00312961599883610

6 54 0.00394658753928789

6 55 0.00343480855985867

6 56 0.00482026086335718

6 57 0.00442888767951566

6 58 0.00529799078935556

6 59 0.00626666811182086

6 60 0.00570251403467925

6 61 0.00500873986523531

6 62 0.00402625983901189

6 63 0.00481091828607561

6 64 0.00470595204856539

6 65 0.00408742429456754

6 66 0.00373149516678006

6 67 0.00348898172850298

6 68 0.00377419084386310

6 69 0.00379933481169614

6 70 0.00512180292940889

6 71 0.00546718534187729

6 72 0.00184947386785628

6 73 0.00223083824828252

6 74 0.00454179231145946

6 75 0.0162810292391036

6 76 0.0182647661469733

7 1 0.00174839695636464

7 2 0.00139726716616317

7 3 0.00143938558785806

7 4 0.00128529211967210

7 5 0.000956996260803700

7 6 0

7 7 0

7 8 0.00647143401156025

7 9 0.00431265655609903

7 10 6.46771218264508e-05

7 11 0.00103990299162871

7 12 0.00184786909540208

7 13 0.00206394796593146

7 14 0

7 15 0.000817238674727716

7 16 0.00114559230113132

7 17 0

7 18 0.000388638715017819

7 19 0.000736555940648254

7 20 0

7 21 0

7 22 0

7 23 0

7 24 0

7 25 6.27622024803598e-05

7 26 0.000151834340113677

7 27 0

7 28 0.000585534850847114

7 29 0.000561577248217038

7 30 0.000287693425700564

7 31 0.000643081746989016

7 32 0.000541303493584389

7 33 0

7 34 0

7 35 0

7 36 0.00109845126716634

7 37 0.00103524665757992

7 38 0.00111806677582516

7 39 0

7 40 0.00460458622858240

7 41 0.00284061112342082

7 42 0.00125937195708414

7 43 0.00216986445393408

7 44 0.00176205781535688

7 45 0.00104536689277113

7 46 0.00274885238985134

7 47 0.00376073202614735

7 48 0.00146813917958777

7 49 0.00343414568847478

7 50 0.00305515438283244

7 51 0.00141267356530839

7 52 0.000685347314073992

7 53 0

7 54 0.000467444358744906

7 55 5.91607319511134e-06

7 56 0.00153074188761670

7 57 0.00106272944185615

7 58 0.00170200287011946

7 59 0.00292263095229806

7 60 0.00248047157673825

7 61 0.00181969617154165

7 62 0.000824900106490167

7 63 0.00156629650617335

7 64 0.000922256966154444

7 65 0.000925374042260507

7 66 0.000540223267214812

7 67 9.01118073427520e-05

7 68 0.000435097525644523

7 69 0.000637766666188977

7 70 0.00190874520291562

7 71 0.00226026301679716

7 72 0

7 73 0

7 74 0.00100357861131006

7 75 0.0132772998875967

7 76 0.0151271776857678

8 1 0

8 2 0

8 3 0

8 4 0

8 5 0

8 6 0

8 7 0

8 8 0.00466867323047498

8 9 0.00211695034795123

8 10 0

8 11 0

8 12 0

8 13 0

8 14 0

8 15 0

8 16 0

8 17 0

8 18 0

8 19 0

8 20 0

8 21 0

8 22 0

8 23 0

8 24 0

8 25 0

8 26 0

8 27 0

8 28 0

8 29 0

8 30 0

8 31 0

8 32 0

8 33 0

8 34 0

8 35 0

8 36 0

8 37 0

8 38 0

8 39 0

8 40 0.00222638548616583

8 41 0.000430407069316741

8 42 0

8 43 0

8 44 0

8 45 0

8 46 0.000366631365814829

8 47 0.00136490904280173

8 48 0

8 49 0.00109375775824472

8 50 0.000734887403707973

8 51 0

8 52 0

8 53 0

8 54 0

8 55 0

8 56 0

8 57 0

8 58 0

8 59 0.000563719776234928

8 60 9.98661834035186e-05

8 61 0

8 62 0

8 63 0

8 64 0

8 65 0

8 66 0

8 67 0

8 68 0

8 69 0

8 70 0

8 71 0

8 72 0

8 73 0

8 74 0

8 75 0.0111839812386698

8 76 0.0135420495395779

9 1 0.00151249990096181

9 2 0.00125838557089197

9 3 0.00152525120243707

9 4 0.00156168447836924

9 5 0.00119515837465745

9 6 0

9 7 0

9 8 0.00638659355365068

9 9 0.00284935037486400

9 10 0

9 11 0.000919037163646830

9 12 0.00189974723195285

9 13 0.00214924865297772

9 14 0

9 15 0.000847046525696116

9 16 0.00108337803766145

9 17 0

9 18 0.000427924604237184

9 19 0.000776635888810773

9 20 0

9 21 0

9 22 0

9 23 0

9 24 0

9 25 0.000137631664811733

9 26 0.000197521932056244

9 27 0

9 28 0.000632131770107902

9 29 0.000601991448004990

9 30 0.000316413300021634

9 31 0.000696831493143035

9 32 0.000612641974144346

9 33 0

9 34 0

9 35 2.23667436223263e-05

9 36 0.00112886318381800

9 37 0.00109781115635865

9 38 0.00115828813506363

9 39 0

9 40 0.00467255419568025

9 41 0.00286850030300101

9 42 0.00134159224361252

9 43 0.00221179197102372

9 44 0.00160946951611807

9 45 0.00105789888919405

9 46 0.00274133587195924

9 47 0.00370408997138860

9 48 0.00139023802890204

9 49 0.00326749634430645

9 50 0.00308456325691231

9 51 0.00140768005244762

9 52 0.000750876829556102

9 53 0

9 54 0.000558185244764253

9 55 0.000101533656983288

9 56 0.00158666767629945

9 57 0.00113760850435451

9 58 0.00173710555114559

9 59 0.00294530546999516

9 60 0.00249736042751336

9 61 0.00186183226581638

9 62 0.000891763098819942

9 63 0.00161242352302082

9 64 0.000875313323127736

9 65 0.000986984124910884

9 66 0.000524368479961401

9 67 9.90966565472817e-06

9 68 0.000241075189835294

9 69 0.000542837756359993

9 70 0.00197132422344681

9 71 0.00231156876648408

9 72 0

9 73 0

9 74 0.00108080777684549

9 75 0.0129488600882626

9 76 0.0149668192256136

10 1 0.00157669959434847

10 2 0.00135496392008494

10 3 0.00199971500433982

10 4 0.00302380308773098

10 5 0.00287493129878902

10 6 0.00209301467013834

10 7 0.00112025707223018

10 8 0.00671789612316820

10 9 0.00521856430645173

10 10 0

10 11 0.00185662643392459

10 12 0.00225364541028200

10 13 0.00247617627590480

10 14 0

10 15 0.00103731756086189

10 16 0.00125661845795733

10 17 0

10 18 0.000498722879504698

10 19 0.000891906492567207

10 20 0

10 21 0

10 22 0

10 23 0

10 24 0

10 25 0.000270162271266750

10 26 0.000348686028470824

10 27 0

10 28 0.000855993877992289

10 29 0.000727107985298403

10 30 0.000405742438606471

10 31 0.000913936707693797

10 32 0.000669802767859218

10 33 0

10 34 0

10 35 0.000144644031509711

10 36 0.00119830381616742

10 37 0.00121964465595731

10 38 0.00130934882243455

10 39 0

10 40 0.00517847045843556

10 41 0.00294526548592400

10 42 0.00135467705006220

10 43 0.00220957685389767

10 44 0.00167577898267546

10 45 0.00116506102601022

10 46 0.00286009431045131

10 47 0.00374974242780535

10 48 0.00150230845848676

10 49 0.00337714152805091

10 50 0.00335968689763455

10 51 0.00190760826010483

10 52 0.00156091967338423

10 53 0.000870975397402551

10 54 0.00155368729797756

10 55 0.000533881838416295

10 56 0.00190197798629665

10 57 0.00154770535075222

10 58 0.00224533782093517

10 59 0.00315722421542641

10 60 0.00261750768254043

10 61 0.00190243141452928

10 62 0.000902451571000662

10 63 0.00168585008514199

10 64 0.00143100584778000

10 65 0.00145365659165736

10 66 0.00156123757682280

10 67 0.000435923143214834

10 68 0.000214106303959238

10 69 0.000532374512610789

10 70 0.00207348655944206

10 71 0.00241918525106422

10 72 0

10 73 0

10 74 0.00156690177400831

10 75 0.0134209414877109

10 76 0.0153562919295329

11 1 0.00369656674756613

11 2 0.00336242428914169

11 3 0.00346984287744578

11 4 0.00318906213976022

11 5 0.00333743652041374

11 6 0.00163749816505143

11 7 0.00214089513358773

11 8 0.00854510383290658

11 9 0.00653697095998651

11 10 0.00250943651170849

11 11 0

11 12 0.00315796278265473

11 13 0.00383495033714532

11 14 0.000938078227279093

11 15 0.00311263041739940

11 16 0.00342158738734299

11 17 0.00223234680558504

11 18 0.00279983198900674

11 19 0.00310097160867728

11 20 0.00170288077768821

11 21 0.00166318365558471

11 22 0.00122701742466480

11 23 0.00150837294260409

11 24 0.00154653361096335

11 25 0.00243554542161362

11 26 0.00259705051133874

11 27 0.00160148872625776

11 28 0.00299944447555728

11 29 0.00293425300923911

11 30 0.00268430210675830

11 31 0.00310518923640318

11 32 0.00290607843235985

11 33 0.00209939787327573

11 34 0.00147626886110652

11 35 0.00235552832667019

11 36 0.00345634243055437

11 37 0.00339093896428172

11 38 0.00348716770462307

11 39 0.00179484288398979

11 40 0.00714788061395855

11 41 0.00514076985286432

11 42 0.00356549502558778

11 43 0.00444918522860782

11 44 0.00390780835323812

11 45 0.00341405005076539

11 46 0.00509968413773748

11 47 0.00603856780723561

11 48 0.00366670405517466

11 49 0.00550163380039648

11 50 0.00538152801350966

11 51 0.00380215424044716

11 52 0.00337274190611936

11 53 0.00245621262800344

11 54 0.00315848759934090

11 55 0.00249049256067224

11 56 0.00397122704463904

11 57 0.00352040519767416

11 58 0.00415641266783617

11 59 0.00529489384415705

11 60 0.00484995429112223

11 61 0.00418005265148236

11 62 0.00316783403944160

11 63 0.00387374406615809

11 64 0.00335703026330736

11 65 0.00319281406048705

11 66 0.00274353272387640

11 67 0.00227828870157637

11 68 0.00244169469706984

11 69 0.00279474800616036

11 70 0.00429470185319170

11 71 0.00461587760877436

11 72 0.000292778891839429

11 73 0.000883290971275152

11 74 0.00345923054843733

11 75 0.0154725053484537

11 76 0.0172866421362250

12 1 0.00289185013182613

12 2 0.00246376080842853

12 3 0.00243820147636709

12 4 0.00215270138192336

12 5 0.00242591993544639

12 6 0.000638245092343337

12 7 0.00129642851176071

12 8 0.00782645106190150

12 9 0.00594622035321574

12 10 0.00170423826598476

12 11 0.00152065858622136

12 12 0.000361023438553598

12 13 0.00265295447250208

12 14 0

12 15 0.00226195988094458

12 16 0.00264069591232152

12 17 0.00137558942048699

12 18 0.00198199292324103

12 19 0.00232478074855050

12 20 0.000952434272916691

12 21 0.000913277525164791

12 22 0.000564941061910629

12 23 0.000773539963535574

12 24 0.000892700660852674

12 25 0.00169191892749954

12 26 0.00182122399344387

12 27 0.000844732514915347

12 28 0.00224724135239490

12 29 0.00219163889238799

12 30 0.00192965615436158

12 31 0.00235406448008407

12 32 0.00217710412489652

12 33 0.00144041979686604

12 34 0.000897067427957365

12 35 0.00161733173649520

12 36 0.00268875759316800

12 37 0.00264487963412133

12 38 0.00272830058858076

12 39 0.000997757120227094

12 40 0.00627075852749026

12 41 0.00433940387134912

12 42 0.00285780745445574

12 43 0.00372828192563868

12 44 0.00321783095096406

12 45 0.00269119112357713

12 46 0.00440225342132522

12 47 0.00535607918956110

12 48 0.00291167339113019

12 49 0.00484029905688149

12 50 0.00474713434190432

12 51 0.00304032336270987

12 52 0.00280201372165922

12 53 0.00189199772606496

12 54 0.00272029039659627

12 55 0.00188896213879752

12 56 0.00321480994546008

12 57 0.00276954418765030

12 58 0.00355945414044545

12 59 0.00457020056384727

12 60 0.00411137943262629

12 61 0.00340225765768193

12 62 0.00238104144271617

12 63 0.00295503074147574

12 64 0.00220494816719996

12 65 0.00233342097029143

12 66 0.00183691473120362

12 67 0.00146270075661326

12 68 0.00169588149562261

12 69 0.00210019783382298

12 70 0.00356917432684561

12 71 0.00387926357032564

12 72 0

12 73 0

12 74 0.00271652103043007

12 75 0.0146348828916560

12 76 0.0167057208767465

13 1 0.00435855143631958

13 2 0.00379987090642031

13 3 0.00333950624499446

13 4 0.00305509556288341

13 5 0.00422339469097655

13 6 0.00269251372486323

13 7 0.00318334858806835

13 8 0.00968196371766472

13 9 0.00783195516605661

13 10 0.00350658629000555

13 11 0.00388803887126998

13 12 0.00443342326007268

13 13 0.000545916995511875

13 14 0

13 15 0.00383850329394209

13 16 0.00444385510443490

13 17 0.00338371757390554

13 18 0.00398885221544010

13 19 0.00415443132088217

13 20 0.00306141845595787

13 21 0.00298681057557348

13 22 0.00288368209077305

13 23 0.00302402098734489

13 24 0.00329266243428117

13 25 0.00358955132784378

13 26 0.00406396956966970

13 27 0.00342368686326422

13 28 0.00421113844698839

13 29 0.00418023821744551

13 30 0.00393959178992254

13 31 0.00437134527967831

13 32 0.00412793995479488

13 33 0.00337935960743341

13 34 0.00281666065151853

13 35 0.00356732320568554

13 36 0.00455839048144707

13 37 0.00452578167844520

13 38 0.00459341562296250

13 39 0.00377133572010757

13 40 0.00859869836352634

13 41 0.00621702100446431

13 42 0.00459088694726950

13 43 0.00546995946051021

13 44 0.00509202621835192

13 45 0.00460975435896249

13 46 0.00640550989614819

13 47 0.00719150626044307

13 48 0.00487326628395313

13 49 0.00665915069288126

13 50 0.00641588287552941

13 51 0.00473371322171390

13 52 0.00483075770910069

13 53 0.00378855446340531

13 54 0.00456713098477035

13 55 0.00390212256678701

13 56 0.00498785631784726

13 57 0.00458114594836478

13 58 0.00541897602354113

13 59 0.00629294091318278

13 60 0.00596793557858277

13 61 0.00524411584199547

13 62 0.00421002687207928

13 63 0.00473548543484448

13 64 0.00390896734452784

13 65 0.00415861683198121

13 66 0.00372935184769085

13 67 0.00329786339872329

13 68 0.00354597291638248

13 69 0.00390724402723941

13 70 0.00548226857076850

13 71 0.00572094416568436

13 72 0.00168375177286029

13 73 0.00211592360496737

13 74 0.00466120511461998

13 75 0.0165264486484193

13 76 0.0196817942036047

14 1 0.00527542304938078

14 2 0.00478738936784517

14 3 0.00384736313130474

14 4 0.00374303514912422

14 5 0.00527246843860163

14 6 0.00370729511605439

14 7 0.00398880734900975

14 8 0.0106502724704877

14 9 0.00866353202528591

14 10 0.00425831571398860

14 11 0.00479577384850094

14 12 0.00533241407181628

14 13 0.00407892051873404

14 14 0

14 15 0.00475751297362481

14 16 0.00534401233293902

14 17 0.00426472143190226

14 18 0.00478796492749500

14 19 0.00494146463498946

14 20 0.00390100826988249

14 21 0.00379355785446456

14 22 0.00473460433513906

14 23 0.00409768025450141

14 24 0.00486175498721575

14 25 0.00450528753717572

14 26 0.00533963226914536

14 27 0.00507189626394500

14 28 0.00541991676305886

14 29 0.00528087876396211

14 30 0.00504130052636143

14 31 0.00559516292194029

14 32 0.00510556963491549

14 33 0.00424986098520752

14 34 0.00379591971007309

14 35 0.00458574629316200

14 36 0.00555037328851238

14 37 0.00613857228477810

14 38 0.00624304633078054

14 39 0.00574920614564634

14 40 0.00976272287139712

14 41 0.00703899697647092

14 42 0.00548533840910737

14 43 0.00633187964132165

14 44 0.00625011302891132

14 45 0.00633981368370784

14 46 0.00788256976478097

14 47 0.00826076308337009

14 48 0.00594270895073479

14 49 0.00779129012991342

14 50 0.00733281220980042

14 51 0.00554906343774031

14 52 0.00585739960456499

14 53 0.00492739880956306

14 54 0.00550543395593772

14 55 0.00538694247834393

14 56 0.00605821981155541

14 57 0.00567785703175738

14 58 0.00635109405015233

14 59 0.00732512390579310

14 60 0.00675292604786193

14 61 0.00601762173792209

14 62 0.00498788488148949

14 63 0.00573739316360866

14 64 0.00477428826465454

14 65 0.00499498825791289

14 66 0.00457311722177478

14 67 0.00416605714219909

14 68 0.00452615715966798

14 69 0.00487413941081194

14 70 0.00641463788771057

14 71 0.00647894799590365

14 72 0.00326042754623868

14 73 0.00343839237728560

14 74 0.00592307277793147

14 75 0.0175316636889364

14 76 0.0213939687582996

15 1 0

15 2 0

15 3 0.000320452439358188

15 4 0.000100165383471351

15 5 2.65076945599185e-05

15 6 0

15 7 0

15 8 0.00500512420693211

15 9 0.00320837335086244

15 10 0

15 11 0

15 12 0.000670051787794834

15 13 0.000487319066497127

15 14 0

15 15 0

15 16 0

15 17 0

15 18 0

15 19 0

15 20 0

15 21 0

15 22 0

15 23 0

15 24 0

15 25 0

15 26 0

15 27 0

15 28 0

15 29 0

15 30 0

15 31 0

15 32 0

15 33 0

15 34 0

15 35 0

15 36 0

15 37 0

15 38 0

15 39 0

15 40 0.00343036765726601

15 41 0.00161669567840694

15 42 0.000115763316160922

15 43 0.00101158663937340

15 44 0.000429141676920541

15 45 0

15 46 0.00163762369502440

15 47 0.00247440923796027

15 48 0.000212122448124852

15 49 0.00211926112790850

15 50 0.00194194627849598

15 51 0.000295506477670404

15 52 0

15 53 0

15 54 0

15 55 0

15 56 0.000336257866195622

15 57 0

15 58 0.000780036058252165

15 59 0.00167757529432888

15 60 0.00127207560413056

15 61 0.000566014196861087

15 62 0

15 63 0.000154383162511706

15 64 0

15 65 0

15 66 0

15 67 0

15 68 0

15 69 0

15 70 0.000867744641196433

15 71 0.00109682184351323

15 72 0

15 73 0

15 74 0.000161185781475171

15 75 0.0117736682537744

15 76 0.0140004602841444

16 1 0

16 2 0

16 3 0

16 4 0

16 5 0

16 6 0

16 7 0

16 8 0.00388869350148069

16 9 0.00217970881561058

16 10 0

16 11 0

16 12 0

16 13 0

16 14 0

16 15 0

16 16 0

16 17 0

16 18 0

16 19 0

16 20 0

16 21 0

16 22 0

16 23 0

16 24 0

16 25 0

16 26 0

16 27 0

16 28 0

16 29 0

16 30 0

16 31 0

16 32 0

16 33 0

16 34 0

16 35 0

16 36 0

16 37 0

16 38 0

16 39 0

16 40 0.00238320797452340

16 41 0.000592983524272950

16 42 0

16 43 0.000132457088729332

16 44 0

16 45 0

16 46 0.000588424783086405

16 47 0.00139215529039749

16 48 0

16 49 0.00109475648591750

16 50 0.00107938406171515

16 51 0

16 52 0

16 53 0

16 54 0

16 55 0

16 56 0

16 57 0

16 58 0

16 59 0.000710744398433105

16 60 0.000251014599493837

16 61 0

16 62 0

16 63 0

16 64 0

16 65 0

16 66 0

16 67 0

16 68 0

16 69 0

16 70 0

16 71 2.32602081584599e-06

16 72 0

16 73 0

16 74 0

16 75 0.0113185999784362

16 76 0.0140225069165789

17 1 0

17 2 0

17 3 0

17 4 0

17 5 0

17 6 0

17 7 0

17 8 0.00282152942859359

17 9 0.00109042867568654

17 10 0

17 11 0

17 12 0

17 13 0

17 14 0

17 15 0

17 16 0

17 17 0

17 18 0

17 19 0

17 20 0

17 21 0

17 22 0

17 23 0

17 24 0

17 25 0

17 26 0

17 27 0

17 28 0

17 29 0

17 30 0

17 31 0

17 32 0

17 33 0

17 34 0

17 35 0

17 36 0

17 37 0

17 38 0

17 39 0

17 40 0.00137722701035681

17 41 0

17 42 0

17 43 0

17 44 0

17 45 0

17 46 0

17 47 0.000315617195917328

17 48 0

17 49 0

17 50 0

17 51 0

17 52 0

17 53 0

17 54 0

17 55 0

17 56 0

17 57 0

17 58 0

17 59 0

17 60 0

17 61 0

17 62 0

17 63 0

17 64 0

17 65 0

17 66 0

17 67 0

17 68 0

17 69 0

17 70 0

17 71 0

17 72 0

17 73 0

17 74 0

17 75 0.00984267600095135

17 76 0.0127769276810006

18 1 0

18 2 0

18 3 0

18 4 0

18 5 0

18 6 0

18 7 0

18 8 0.00347487125934709

18 9 0.00168822980476985

18 10 0

18 11 0

18 12 0

18 13 0

18 14 0

18 15 0

18 16 0

18 17 0

18 18 0

18 19 0

18 20 0

18 21 0

18 22 0

18 23 0

18 24 0

18 25 0

18 26 0

18 27 0

18 28 0

18 29 0

18 30 0

18 31 0

18 32 0

18 33 0

18 34 0

18 35 0

18 36 0

18 37 0

18 38 0

18 39 0

18 40 0.00195917918117361

18 41 0.000412046102439057

18 42 0

18 43 7.24510642136078e-05

18 44 0

18 45 0

18 46 0.000124535301368411

18 47 0.00114871555365148

18 48 0

18 49 0.000533380402273576

18 50 0.000639102559379157

18 51 0

18 52 0

18 53 0

18 54 0

18 55 0

18 56 0

18 57 0

18 58 0

18 59 0.000521815874488962

18 60 8.91450388752491e-05

18 61 0

18 62 0

18 63 0

18 64 0

18 65 0

18 66 0

18 67 0

18 68 0

18 69 0

18 70 0

18 71 0

18 72 0

18 73 0

18 74 0

18 75 0.0108709361768633

18 76 0.0136139142656835

19 1 0

19 2 0

19 3 0

19 4 0

19 5 0

19 6 0

19 7 0

19 8 0.00394598838575294

19 9 0.00213683149941546

19 10 0

19 11 0

19 12 0

19 13 0

19 14 0

19 15 0

19 16 0

19 17 0

19 18 0

19 19 0

19 20 0

19 21 0

19 22 0

19 23 0

19 24 0

19 25 0

19 26 0

19 27 0

19 28 0

19 29 0

19 30 0

19 31 0

19 32 0

19 33 0

19 34 0

19 35 0

19 36 0

19 37 0

19 38 0

19 39 0

19 40 0.00247036667631628

19 41 0.000793013913507834

19 42 0

19 43 3.99400456583265e-05

19 44 0

19 45 0

19 46 0.000604669045888406

19 47 0.00145451189460133

19 48 0

19 49 0.000968425137822526

19 50 0.000902902040951337

19 51 0

19 52 0

19 53 0

19 54 0

19 55 0

19 56 0

19 57 0

19 58 0

19 59 0.000845929048822081

19 60 0.000267874035418170

19 61 0

19 62 0

19 63 0

19 64 0

19 65 0

19 66 0

19 67 0

19 68 0

19 69 0

19 70 0

19 71 6.77950371485436e-05

19 72 0

19 73 0

19 74 0

19 75 0.0110305919105947

19 76 0.0133651611066203

20 1 0

20 2 0

20 3 0

20 4 0

20 5 0

20 6 0

20 7 0

20 8 0.00292538982430479

20 9 0.00106077625850987

20 10 0

20 11 0

20 12 0

20 13 0

20 14 0

20 15 0

20 16 0

20 17 0

20 18 0

20 19 0

20 20 0

20 21 0

20 22 0

20 23 0

20 24 0

20 25 0

20 26 0

20 27 0

20 28 0

20 29 0

20 30 0

20 31 0

20 32 0

20 33 0

20 34 0

20 35 0

20 36 0

20 37 0

20 38 0

20 39 0

20 40 0.00262615469602900

20 41 0.000663650636307467

20 42 0

20 43 0

20 44 0

20 45 0

20 46 0

20 47 0.000189309303208485

20 48 0

20 49 0.000786001273082038

20 50 0.00315294973114577

20 51 0.000830807591466254

20 52 0

20 53 0

20 54 0

20 55 0

20 56 0

20 57 0

20 58 0.000674249879243805

20 59 0.00183910129399023

20 60 0.000200993511703551

20 61 0

20 62 0

20 63 0

20 64 0

20 65 0

20 66 0

20 67 0

20 68 0

20 69 0

20 70 0

20 71 0

20 72 0

20 73 0.00453459631609532

20 74 0.00208792993125295

20 75 0.0111947592066399

20 76 0.0145727077478579

21 1 0

21 2 0

21 3 0

21 4 0

21 5 0

21 6 0

21 7 0

21 8 0.00308674320659075

21 9 0.00107608672063209

21 10 0

21 11 0

21 12 0

21 13 0

21 14 0

21 15 0

21 16 0

21 17 0

21 18 0

21 19 0

21 20 0

21 21 0

21 22 0

21 23 0

21 24 0

21 25 0

21 26 0

21 27 0

21 28 0

21 29 0

21 30 0

21 31 0

21 32 0

21 33 0

21 34 0

21 35 0

21 36 0

21 37 0

21 38 0

21 39 0

21 40 0.00280336279790205

21 41 0.000976387288602054

21 42 0

21 43 0

21 44 0

21 45 0

21 46 0

21 47 0.000314593271897423

21 48 0

21 49 0.000655877065261157

21 50 0.00264892449404841

21 51 0.000760667394597125

21 52 0

21 53 0

21 54 0

21 55 0

21 56 0

21 57 0

21 58 0.000808093469396254

21 59 0.00168881546766841

21 60 5.26420563737862e-05

21 61 0

21 62 0

21 63 0

21 64 0

21 65 0

21 66 0

21 67 0

21 68 0

21 69 0

21 70 0

21 71 0

21 72 0

21 73 0.00433363947754817

21 74 0.00149824146173261

21 75 0.0110985309768421

21 76 0.0141703142007030

22 1 0

22 2 0

22 3 0

22 4 0

22 5 0

22 6 0

22 7 0

22 8 0.00283123632227411

22 9 0.000526810080984763

22 10 0

22 11 0

22 12 0

22 13 0

22 14 0

22 15 0

22 16 0

22 17 0

22 18 0

22 19 0

22 20 0.00196630882323756

22 21 0.00206792257494992

22 22 0

22 23 0.00622748663347694

22 24 0.00280572699700721

22 25 0.00216216046692153

22 26 0.00424705339083387

22 27 0.00608582337954078

22 28 0.00406176709875539

22 29 0

22 30 0

22 31 0

22 32 0

22 33 0

22 34 0

22 35 0

22 36 0

22 37 0

22 38 0

22 39 0.00410500849209927

22 40 0.00593872018896424

22 41 0.00279307996713030

22 42 0

22 43 0

22 44 0

22 45 0.00136527013321941

22 46 0.000821268122914387

22 47 0.000173616408573718

22 48 0

22 49 0.00128713959851823

22 50 0.00402709140298763

22 51 0.00238497944871008

22 52 0.00813775515878179

22 53 0.00186421776144319

22 54 0.00404313655184940

22 55 0.00118987649001223

22 56 0.00518298471851775

22 57 0.00491705345956250

22 58 0.00600326512819493

22 59 0.00449410172358511

22 60 0

22 61 0

22 62 0

22 63 0

22 64 0

22 65 0

22 66 0

22 67 0

22 68 0

22 69 0

22 70 0

22 71 0

22 72 0.0100674276167425

22 73 0.0171894319458028

22 74 0.00458268875391465

22 75 0.0119340537982016

22 76 0.0190112834821152

23 1 0

23 2 0

23 3 0

23 4 0

23 5 0

23 6 0

23 7 0

23 8 0.00406240391003510

23 9 0.00171033203537652

23 10 0

23 11 0

23 12 0

23 13 0

23 14 0

23 15 0

23 16 0

23 17 0

23 18 0

23 19 0

23 20 0.000770968163627095

23 21 0.000800783799360549

23 22 0.00468027794976811

23 23 0

23 24 0.00342765984891202

23 25 0.000941144448840969

23 26 0.00374632482246151

23 27 0.00533851034538868

23 28 0.00312758071014929

23 29 0

23 30 0

23 31 0

23 32 0

23 33 0

23 34 0

23 35 0

23 36 0

23 37 0.00214733830382086

23 38 0.00227087381976543

23 39 0.00892686388552855

23 40 0.00791552730701650

23 41 0.00193477265763609

23 42 0

23 43 0

23 44 0.00155436230854422

23 45 0.00346755703658419

23 46 0.00231060458128085

23 47 0.00166014474594411

23 48 0

23 49 0.00411534804695612

23 50 0.00710036419619775

23 51 0.00236674886737798

23 52 0.00656868747049821

23 53 0.00956577333648001

23 54 0.00827155253819756

23 55 0.00645494235940425

23 56 0.00592010714316760

23 57 0.00552849787274756

23 58 0.00512807177456065

23 59 0.00529005017279682

23 60 0.000608571507903166

23 61 0

23 62 0

23 63 0

23 64 0

23 65 0

23 66 0

23 67 0

23 68 0

23 69 0

23 70 0

23 71 0

23 72 0.0134411977404365

23 73 0.0157369373797587

23 74 0.00799862144324692

23 75 0.0149824787672750

23 76 0.0244029659998397

24 1 0

24 2 0

24 3 0

24 4 0

24 5 0

24 6 0

24 7 0

24 8 0.00332775667813234

24 9 0.00102815548090351

24 10 0

24 11 0

24 12 0

24 13 0

24 14 0

24 15 0

24 16 0

24 17 0

24 18 0

24 19 0

24 20 0.000831022716887953

24 21 0.00103209956003980

24 22 0

24 23 0.00280741282504693

24 24 0

24 25 0.000938609897921161

24 26 0.00288513488611530

24 27 0.00386247231108416

24 28 0.00272263468481559

24 29 0

24 30 0

24 31 0

24 32 0

24 33 0

24 34 0

24 35 0

24 36 0

24 37 0

24 38 0

24 39 0.00404728024959045

24 40 0.00667141943871696

24 41 0.00366351551275057

24 42 0

24 43 0

24 44 0

24 45 0.00125873939281063

24 46 0.000899060813685071

24 47 0.000557220896991617

24 48 0

24 49 0.00189916472290286

24 50 0.00524274854540929

24 51 0.00377117704191154

24 52 0.00727435013119226

24 53 0.00229497996040173

24 54 0.00406383404296928

24 55 0.00208405260183930

24 56 0.00488413868725324

24 57 0.00462895896610904

24 58 0.00578212802775935

24 59 0.00507645136595813

24 60 0.000234588424585014

24 61 0

24 62 0

24 63 0

24 64 0

24 65 0

24 66 0

24 67 0

24 68 0

24 69 0

24 70 0

24 71 0

24 72 0.00896773339397661

24 73 0.0170198299797749

24 74 0.00487210645043346

24 75 0.0121558948506943

24 76 0.0189021387989232

25 1 0

25 2 0

25 3 0

25 4 0

25 5 0

25 6 0

25 7 0

25 8 0.00306457839279561

25 9 0.00107744003801302

25 10 0

25 11 0

25 12 0

25 13 0

25 14 0

25 15 0

25 16 0

25 17 0

25 18 0

25 19 0

25 20 0

25 21 0

25 22 0

25 23 0

25 24 0

25 25 0

25 26 0

25 27 0

25 28 0

25 29 0

25 30 0

25 31 0

25 32 0

25 33 0

25 34 0

25 35 0

25 36 0

25 37 0

25 38 0

25 39 0

25 40 0.00288866923813225

25 41 0.00269104165994605

25 42 0

25 43 0

25 44 0

25 45 0

25 46 0

25 47 0.000396426178408071

25 48 0

25 49 0.000145145815426462

25 50 0.00123660740220444

25 51 0.00115099307970168

25 52 0.00116848239641931

25 53 0

25 54 0

25 55 0

25 56 0.000165063418154587

25 57 0

25 58 0.00185102267491022

25 59 0.00220264889228972

25 60 2.97434560720955e-05

25 61 0

25 62 0

25 63 0

25 64 0

25 65 0

25 66 0

25 67 0

25 68 0

25 69 0

25 70 0

25 71 0

25 72 0.000113354729771586

25 73 0.00448323668016182

25 74 0.00119679349128254

25 75 0.0105982984592179

25 76 0.0126801830083323

26 1 0

26 2 0

26 3 0

26 4 0

26 5 0

26 6 0

26 7 0

26 8 0.00347257528228540

26 9 0.00127418287777314

26 10 0

26 11 0

26 12 0

26 13 0

26 14 0

26 15 0

26 16 0

26 17 0

26 18 0

26 19 0

26 20 0

26 21 0

26 22 0

26 23 4.71163865756186e-05

26 24 0

26 25 0

26 26 0

26 27 0

26 28 0

26 29 0

26 30 0

26 31 0

26 32 0

26 33 0

26 34 0

26 35 0

26 36 0

26 37 0

26 38 0

26 39 0

26 40 0.00230295457707785

26 41 0.000350725450631306

26 42 0

26 43 0

26 44 0

26 45 0

26 46 0.000712985178676018

26 47 0.000867883100711953

26 48 0

26 49 0.000683431942630319

26 50 0.00105010477710865

26 51 0

26 52 0.000784803559506608

26 53 0.000853060999087552

26 54 0.00130775537354655

26 55 0.000405641355151087

26 56 0.000300122146248416

26 57 0

26 58 0.000190860064362752

26 59 0.000967263197107826

26 60 0

26 61 0

26 62 0

26 63 0

26 64 0

26 65 0

26 66 0

26 67 0

26 68 0

26 69 0

26 70 0

26 71 0

26 72 0.00150392598403593

26 73 0.00308107640779487

26 74 4.24969181596069e-05

26 75 0.0108424848005002

26 76 0.0161056535895567

27 1 0

27 2 0

27 3 0

27 4 0

27 5 0

27 6 0

27 7 0

27 8 0.00302980001523667

27 9 0.000642185149296592

27 10 0

27 11 0

27 12 0

27 13 0

27 14 0

27 15 0

27 16 0

27 17 0

27 18 0

27 19 0

27 20 0

27 21 0

27 22 0.00153565530057709

27 23 0.00422182637427626

27 24 0.00219206404835848

27 25 0

27 26 0.00104006567733039

27 27 0

27 28 0.000426461687600188

27 29 0

27 30 0

27 31 0

27 32 0

27 33 0

27 34 0

27 35 0

27 36 0

27 37 0

27 38 0

27 39 0.00244689333191273

27 40 0.00296067135763056

27 41 4.77491087915727e-05

27 42 0

27 43 0

27 44 0

27 45 0

27 46 0.000513419121583203

27 47 0.000346794390151617

27 48 0

27 49 0.00120884229930018

27 50 0.00271548889622741

27 51 0

27 52 0.00422974381196828

27 53 0.00568581703836624

27 54 0.00599007462400669

27 55 0.00380874920699181

27 56 0.00241408026985213

27 57 0.00200688212076805

27 58 0.00204244914699769

27 59 0.00172471440841926

27 60 0

27 61 0

27 62 0

27 63 0

27 64 0

27 65 0

27 66 0

27 67 0

27 68 0

27 69 0

27 70 0

27 71 0

27 72 0.00751592380369925

27 73 0.0119095882930803

27 74 0.00227945834097199

27 75 0.0117741673770078

27 76 0.0207602663508153

28 1 0

28 2 0

28 3 0

28 4 0

28 5 0

28 6 0

28 7 0

28 8 0.00364726173597602

28 9 0.00149199061830585

28 10 0

28 11 0

28 12 0

28 13 0

28 14 0

28 15 0

28 16 0

28 17 0

28 18 0

28 19 0

28 20 0

28 21 0

28 22 0

28 23 0

28 24 0

28 25 0

28 26 0

28 27 0

28 28 0

28 29 0

28 30 0

28 31 0

28 32 0

28 33 0

28 34 0

28 35 0

28 36 0

28 37 0

28 38 0

28 39 0

28 40 0.00250098343220839

28 41 0.00134658594339132

28 42 0

28 43 0

28 44 0

28 45 5.19004050314464e-06

28 46 0.000663332751871137

28 47 0.000934272028122629

28 48 0

28 49 0.000455500127429009

28 50 0.000615260295057651

28 51 0

28 52 0.00167994916973213

28 53 0

28 54 0.000641458802797756

28 55 0

28 56 0.000598640280007001

28 57 0.000420567161775187

28 58 0.00152963358476010

28 59 0.00155306701983571

28 60 0

28 61 0

28 62 0

28 63 0

28 64 0

28 65 0

28 66 0

28 67 0

28 68 0

28 69 0

28 70 0

28 71 0

28 72 0.000708430383974279

28 73 0.00209580449879787

28 74 0.000562571649168953

28 75 0.0106932592450002

28 76 0.0142834871259749

29 1 0.00112239305849737

29 2 0.000911194005722260

29 3 0.00119009450391905

29 4 0.00112348638836846

29 5 0.000730499511702676

29 6 0

29 7 0

29 8 0.00568733174165048

29 9 0.00387990973708852

29 10 0

29 11 0.000583351217266159

29 12 0.00140870086385309

29 13 0.00173805987049125

29 14 0

29 15 0.000405516833228980

29 16 0.000699804946843075

29 17 0

29 18 0

29 19 0.000206467961117840

29 20 0

29 21 0

29 22 0

29 23 0

29 24 0

29 25 0

29 26 0

29 27 0

29 28 0.000277635077823579

29 29 0

29 30 0

29 31 0.000617781912205795

29 32 0

29 33 0

29 34 0

29 35 0

29 36 0.000593919998487680

29 37 0.000514701700581877

29 38 0.000577473372383230

29 39 0

29 40 0.00418814065053730

29 41 0.00229085716945676

29 42 0.000856277566242958

29 43 0.00167271104065503

29 44 0.00101328525971989

29 45 0.000541979345461408

29 46 0.00233360354710721

29 47 0.00317464887143304

29 48 0.000824993193451973

29 49 0.00268683378782808

29 50 0.00259623724789726

29 51 0.000961783974338326

29 52 0.000240622398149037

29 53 0

29 54 0

29 55 0

29 56 0.00102329696271297

29 57 0.000549432977180908

29 58 0.00120977861599902

29 59 0.00243014814679965

29 60 0.00196696158392329

29 61 0.00128862730973456

29 62 0.000293480530804635

29 63 0.00107114450340084

29 64 0.000369769968356026

29 65 0.000313115993726743

29 66 0

29 67 0

29 68 0

29 69 0

29 70 0.00144271211429947

29 71 0.00175477898118825

29 72 0

29 73 0

29 74 0.000457813544339936

29 75 0.0124474770500453

29 76 0.0149706666660641

30 1 0.000910325979010085

30 2 0.000741056017312003

30 3 0.000994844745118462

30 4 0.000917809723054441

30 5 0.000517310626907297

30 6 0

30 7 0

30 8 0.00545712391745035

30 9 0.00368425628675972

30 10 0

30 11 0.000418131791955045

30 12 0.00137348123599879

30 13 0.00154531030236638

30 14 0

30 15 0.000209077865037766

30 16 0.000521896390798693

30 17 0

30 18 0

30 19 2.72094482395846e-05

30 20 0

30 21 0

30 22 0

30 23 0

30 24 0

30 25 0

30 26 0

30 27 0

30 28 0

30 29 0

30 30 0

30 31 0.000402015984297344

30 32 0

30 33 0

30 34 0

30 35 0

30 36 0.000392735230726915

30 37 0.000315967246531490

30 38 0.000411226416751176

30 39 0

30 40 0.00394176898170651

30 41 0.00208603102787219

30 42 0.000480859986754734

30 43 0.00140247322779274

30 44 0.000842334431635949

30 45 0.000370303584246168

30 46 0.00214895810291083

30 47 0.00294634733178667

30 48 0.000660249167128701

30 49 0.00248649739629769

30 50 0.00235780033673938

30 51 0.000746566630760115

30 52 0.000192126845706020

30 53 0

30 54 0.000169121890531754

30 55 0

30 56 0.000930671552099360

30 57 0.000511366444770900

30 58 0.00133521206667608

30 59 0.00223236236684987

30 60 0.00172226226236760

30 61 0.00106589055748085

30 62 5.68311744786332e-05

30 63 0.000879961397317164

30 64 0.000270216310436000

30 65 0.000103232242061768

30 66 0

30 67 0

30 68 0

30 69 0

30 70 0.00130853114527885

30 71 0.00152714628245088

30 72 0

30 73 0

30 74 0.000324367530525560

30 75 0.0123472778429459

30 76 0.0151124131717924

31 1 0

31 2 0

31 3 0

31 4 0

31 5 0

31 6 0

31 7 0

31 8 0.00386524695989449

31 9 0.00188369921567921

31 10 0

31 11 0

31 12 0

31 13 0

31 14 0

31 15 0

31 16 0

31 17 0

31 18 0

31 19 0

31 20 0

31 21 0

31 22 0

31 23 0

31 24 0

31 25 0

31 26 0

31 27 0

31 28 0

31 29 0

31 30 0

31 31 0

31 32 0

31 33 0

31 34 0

31 35 0

31 36 0

31 37 0

31 38 0

31 39 0

31 40 0.00216729760947643

31 41 0.000474797160277096

31 42 0

31 43 0

31 44 0

31 45 0

31 46 0.000756862989439278

31 47 0.00126110295715332

31 48 0

31 49 0.000744685599389117

31 50 0.000600200222452552

31 51 0

31 52 0

31 53 0

31 54 0

31 55 0

31 56 0

31 57 0

31 58 0

31 59 0.000452366193316789

31 60 0

31 61 0

31 62 0

31 63 0

31 64 0

31 65 0

31 66 0

31 67 0

31 68 0

31 69 0

31 70 0

31 71 0

31 72 0

31 73 0

31 74 0

31 75 0.0104415191166849

31 76 0.0138228987377294

32 1 0.00170849247850091

32 2 0.00143629354127783

32 3 0.00169433938535080

32 4 0.00164114464418430

32 5 0.00131140046799161

32 6 0

32 7 0

32 8 0.00625844944551734

32 9 0.00447592030859356

32 10 9.22200449299249e-05

32 11 0.00117169167232434

32 12 0.00201310228769391

32 13 0.00230956622120715

32 14 0

32 15 0.00100534642692207

32 16 0.00132699089292954

32 17 2.38810052949345e-05

32 18 0.000538605389173386

32 19 0.000819101096578012

32 20 0

32 21 0

32 22 0

32 23 0

32 24 0

32 25 0.000178423561322583

32 26 0.000335876477378982

32 27 0

32 28 0.000750051838901811

32 29 0.000515782111565055

32 30 0.000218080061799131

32 31 0.00104625842058370

32 32 0

32 33 0

32 34 0

32 35 0

32 36 0.00121200295355939

32 37 0.00113865832544113

32 38 0.00117896697168829

32 39 0

32 40 0.00479445355620312

32 41 0.00289872958215642

32 42 0.00129894464979918

32 43 0.00220096843754447

32 44 0.00165323587797184

32 45 0.00113152717303600

32 46 0.00283804519944153

32 47 0.00374368954483106

32 48 0.00144317152892792

32 49 0.00324646545577156

32 50 0.00314917973162265

32 51 0.00144666744989475

32 52 0.000854104851942372

32 53 0

32 54 0.000625647085316095

32 55 0.000100940616279410

32 56 0.00162695417708003

32 57 0.00118151539056155

32 58 0.00186535542611999

32 59 0.00304454440687774

32 60 0.00253713226868058

32 61 0.00187931894519611

32 62 0.000867636770034097

32 63 0.00163561263632050

32 64 0.000881119967633293

32 65 0.000893956404577734

32 66 0.000469522109460052

32 67 0

32 68 0.000138699730221736

32 69 0.000511369187432220

32 70 0.00198730808365954

32 71 0.00235219379944607

32 72 0

32 73 0

32 74 0.00111780349251511

32 75 0.0129491328481481

32 76 0.0150128497095379

33 1 0.00275205219862162

33 2 0.00250978511690569

33 3 0.00309357954486544

33 4 0.00345109122921017

33 5 0.00251343153906325

33 6 0.000691940487131282

33 7 0.000956281528783132

33 8 0.00734496508979543

33 9 0.00555904641359772

33 10 0.00127421155538843

33 11 0.00254278559174903

33 12 0.00415528071323112

33 13 0.00370947259478771

33 14 0.000845151779486297

33 15 0.00210711102739436

33 16 0.00243617267448959

33 17 0.00119443451102930

33 18 0.00164537942858833

33 19 0.00197385427250896

33 20 0.000644733906942863

33 21 0.000643385919449457

33 22 0.000247598896543688

33 23 0.000437131887097153

33 24 0.000526016506980587

33 25 0.00140823658820788

33 26 0.00152931491109709

33 27 0.000423814511643861

33 28 0.00186723436599312

33 29 0.00167353536712345

33 30 0.00151197715344131

33 31 0.00210111226115828

33 32 0.00155770624340912

33 33 0

33 34 0

33 35 0.00128342207340293

33 36 0.00227023070145305

33 37 0.00225847913204436

33 38 0.00239788794986467

33 39 0.000550668109510766

33 40 0.00647925313856879

33 41 0.00421084093558011

33 42 0.00246862108075585

33 43 0.00350844959409435

33 44 0.00278518749777223

33 45 0.00232614627201511

33 46 0.00391778380148322

33 47 0.00486238654181981

33 48 0.00255924417972941

33 49 0.00455499092826572

33 50 0.00464554743740364

33 51 0.00302812429089117

33 52 0.00262852712394812

33 53 0.00140585533143889

33 54 0.00254124862163185

33 55 0.00159346080551492

33 56 0.00282985345499631

33 57 0.00254292126820421

33 58 0.00358914669032162

33 59 0.00425225860562362

33 60 0.00368120671916083

33 61 0.00294901338981257

33 62 0.00195742921083558

33 63 0.00285191861176504

33 64 0.00265020341337963

33 65 0.00213736141481380

33 66 0.00168000958121795

33 67 0.00107142210623223

33 68 0.00128977390166130

33 69 0.00161926571101534

33 70 0.00304936230543429

33 71 0.00349486197025650

33 72 0

33 73 0.00170355922035859

33 74 0.00280643700403782

33 75 0.0153932380332564

33 76 0.0169152395265266

34 1 0.00361088631242812

34 2 0.00333781640491948

34 3 0.00376878839357320

34 4 0.00386916600547149

34 5 0.00319593740683177

34 6 0.00142705548706035

34 7 0.00168089764236512

34 8 0.00813544464058480

34 9 0.00634405898657398

34 10 0.00196009172788880

34 11 0.00312817388203723

34 12 0.00450278074424815

34 13 0.00429146611880904

34 14 0.00135915895610617

34 15 0.00289351846236718

34 16 0.00325535324467907

34 17 0.00198910951876863

34 18 0.00243282774554576

34 19 0.00273987415026000

34 20 0.00143105681524125

34 21 0.00139052113909999

34 22 0.000904141138945880

34 23 0.00105191666129345

34 24 0.00108758815307608

34 25 0.00218268735195237

34 26 0.00237572008036424

34 27 0.00129049902512790

34 28 0.00269186599167304

34 29 0.00247067241386278

34 30 0.00232914819974273

34 31 0.00299588124914363

34 32 0.00226450699896574

34 33 0.00116130050291718

34 34 0

34 35 0.00215890529451035

34 36 0.00305589782387899

34 37 0.00302253947626491

34 38 0.00316602986910558

34 39 0.00121504956570351

34 40 0.00677190139729667

34 41 0.00488538498355162

34 42 0.00313067718055848

34 43 0.00416324606444096

34 44 0.00356365751272858

34 45 0.00308126533282804

34 46 0.00472836928414200

34 47 0.00564882621000995

34 48 0.00322983690562151

34 49 0.00514778640169933

34 50 0.00509684016006895

34 51 0.00353397782894194

34 52 0.00318977449055247

34 53 0.00211121082125265

34 54 0.00324291669475496

34 55 0.00242845858936125

34 56 0.00364578642491797

34 57 0.00343293638027653

34 58 0.00459299239271438

34 59 0.00500392801343030

34 60 0.00443106661860959

34 61 0.00369376759102036

34 62 0.00270961066890008

34 63 0.00357267534802952

34 64 0.00307586360042755

34 65 0.00277787017240083

34 66 0.00234846328958616

34 67 0.00181764235137605

34 68 0.00204225144852443

34 69 0.00239504126600154

34 70 0.00381625088104187

34 71 0.00420342401118334

34 72 0.000821317133706589

34 73 0.00262895485370063

34 74 0.00311405604158388

34 75 0.0159168259205558

34 76 0.0177243951413719

35 1 0.000587695520850695

35 2 0.000406731122823190

35 3 0.000688480230651822

35 4 0.000690575411985361

35 5 0.000248309886484721

35 6 0

35 7 0

35 8 0.00519659121203175

35 9 0.00336131111664950

35 10 0

35 11 9.47132392256078e-05

35 12 0.00108759528327584

35 13 0.00118392841642467

35 14 0

35 15 0

35 16 0.000171342196939484

35 17 0

35 18 0

35 19 0

35 20 0

35 21 0

35 22 0

35 23 0

35 24 0

35 25 0

35 26 0

35 27 0

35 28 0

35 29 0

35 30 0

35 31 0

35 32 0

35 33 0

35 34 0

35 35 0

35 36 0.000166375540676889

35 37 2.99070808304491e-05

35 38 0.000111353490908905

35 39 0

35 40 0.00374332342837214

35 41 0.00182137061894261

35 42 0.000217174268649201

35 43 0.00112429879918663

35 44 0.000549270223430542

35 45 6.20146480869721e-05

35 46 0.00173819965445710

35 47 0.00264881618884583

35 48 0.000309957404562300

35 49 0.00218327092383053

35 50 0.00214521572190618

35 51 0.000459625238498074

35 52 0

35 53 0

35 54 0

35 55 0

35 56 0.000577417839956573

35 57 0.000207356775109524

35 58 0.00114676944235637

35 59 0.00198846540698083

35 60 0.00145187947103886

35 61 0.000778358282488290

35 62 0

35 63 0.000555440434292231

35 64 0

35 65 0

35 66 0

35 67 0

35 68 0

35 69 0

35 70 0.000867663353175785

35 71 0.00128502440142630

35 72 0

35 73 0

35 74 0.000177729013273176

35 75 0.0122226224563389

35 76 0.0144335135002733

36 1 0

36 2 0

36 3 0

36 4 0

36 5 0

36 6 0

36 7 0

36 8 0.00426562829501803

36 9 0.00245583899979929

36 10 0

36 11 0

36 12 0

36 13 0.000255066933155357

36 14 0

36 15 0

36 16 0

36 17 0

36 18 0

36 19 0

36 20 0

36 21 0

36 22 0

36 23 0

36 24 0

36 25 0

36 26 0

36 27 0

36 28 0

36 29 0

36 30 0

36 31 0

36 32 0

36 33 0

36 34 0

36 35 0

36 36 0

36 37 0

36 38 0

36 39 0

36 40 0.00288444283686551

36 41 0.000931521968203786

36 42 0

36 43 0.000196126154527077

36 44 0

36 45 0

36 46 0.000832123557841791

36 47 0.00177717000980993

36 48 0

36 49 0.00127765913147904

36 50 0.00116004432631334

36 51 0

36 52 0

36 53 0

36 54 0

36 55 0

36 56 0

36 57 0

36 58 0

36 59 0.000958054786533935

36 60 0.000525894919846115

36 61 0

36 62 0

36 63 0

36 64 0

36 65 0

36 66 0

36 67 0

36 68 0

36 69 0

36 70 2.97992036279449e-05

36 71 0.000361216103679396

36 72 0

36 73 0

36 74 0

36 75 0.0109401379864907

36 76 0.0132167717999481

37 1 0.000145164603807091

37 2 0.000462614632490588

37 3 7.10839198493352e-05

37 4 0

37 5 0

37 6 0

37 7 0

37 8 0.00431110532849144

37 9 0.00248011757849270

37 10 0

37 11 0

37 12 0

37 13 0.000743789957990793

37 14 0

37 15 0

37 16 0

37 17 0

37 18 0

37 19 0

37 20 0

37 21 0

37 22 0

37 23 0

37 24 9.24822547280213e-05

37 25 0

37 26 0.00107976279821970

37 27 0.00155550004803834

37 28 0.000442850811503748

37 29 0

37 30 0

37 31 0.00117081667719332

37 32 0

37 33 0

37 34 0

37 35 0

37 36 0

37 37 0

37 38 0

37 39 0

37 40 0.00317138655661342

37 41 0.00101071680148529

37 42 0

37 43 0.000250524395635832

37 44 0.000660092395525225

37 45 0.000969269168995035

37 46 0.00118123607030152

37 47 0.00197535873827626

37 48 0

37 49 0.00148217507992227

37 50 0.00129476277677065

37 51 0

37 52 0.000746916055438640

37 53 0.000684891809797805

37 54 0.00104302607067297

37 55 0

37 56 0.000165715683125234

37 57 0

37 58 8.19784363211840e-05

37 59 0.00120928932066156

37 60 0.000574129837473292

37 61 0

37 62 0

37 63 0.000198512815044183

37 64 0

37 65 0

37 66 0

37 67 0

37 68 0

37 69 0

37 70 0.000259831360634122

37 71 0.000367312472055736

37 72 0

37 73 0

37 74 0

37 75 0.0110516714852162

37 76 0.0146743159471241

38 1 0.000257254974604659

38 2 0.000739012599566824

38 3 0.000168218114490470

38 4 0

38 5 0

38 6 0

38 7 0

38 8 0.00433532349665716

38 9 0.00250846674625016

38 10 0

38 11 0

38 12 1.27930055425218e-05

38 13 0.000829088389310673

38 14 0

38 15 0

38 16 0.000111612376334835

38 17 0

38 18 0

38 19 0

38 20 0

38 21 0

38 22 0

38 23 0

38 24 1.24968810639725e-05

38 25 0

38 26 0.00148800351303402

38 27 0.00204174767043686

38 28 0.000705242293574410

38 29 0

38 30 0

38 31 0.00164340554050726

38 32 0

38 33 0

38 34 0

38 35 0

38 36 0

38 37 0

38 38 0

38 39 0

38 40 0.00315613170296847

38 41 0.00101656839093861

38 42 0

38 43 0.000384062790996897

38 44 0.000733981205986889

38 45 0.00105470390166434

38 46 0.00112127178946764

38 47 0.00194468643278539

38 48 0

38 49 0.00146747229791072

38 50 0.00127165385899597

38 51 0

38 52 0.000665946036187304

38 53 0.000694609569578342

38 54 0.00103733092390579

38 55 0

38 56 0.000164040146501343

38 57 0

38 58 0.000158367478348909

38 59 0.00119376950823830

38 60 0.000603923162576736

38 61 0

38 62 0

38 63 0.000250691682994741

38 64 0

38 65 0

38 66 0

38 67 0

38 68 0

38 69 0

38 70 0.000215392951620486

38 71 0.000435737813472448

38 72 0

38 73 0

38 74 0

38 75 0.0111173015262260

38 76 0.0146789753722103

39 1 0.00164775061388955

39 2 0.00260932117797452

39 3 0.00297947339854709

39 4 0.00348093016389417

39 5 0.00206484734731438

39 6 0

39 7 0

39 8 0.00605120836688755

39 9 0.00421539254929837

39 10 0

39 11 0.00117255354421864

39 12 0.00193926805535338

39 13 0.00325828743676770

39 14 0.000495358541563351

39 15 0.00101341708511993

39 16 0.00135935528263531

39 17 0

39 18 0.000447010079017929

39 19 0.00104535945600959

39 20 0.00257204535258260

39 21 0.00243279825169929

39 22 0.00731667580778916

39 23 0.00747248018123703

39 24 0.00765440485655833

39 25 0.00237493003300726

39 26 0.00511723491702132

39 27 0.00718010316329631

39 28 0.00402318813802560

39 29 0.00132514856481925

39 30 0.00143296142462868

39 31 0.00321522226324111

39 32 0.000458570110014378

39 33 0.000626997524547424

39 34 0.000244448503677441

39 35 0.000257922601330729

39 36 0.000971524992674833

39 37 0.00167392183235615

39 38 0.00171760229409396

39 39 0

39 40 0.00606792914560461

39 41 0.00278231499925286

39 42 0.00118915831255728

39 43 0.00197502441193509

39 44 0.00249157156286406

39 45 0.00267867276230327

39 46 0.00305513407811120

39 47 0.00380535030033957

39 48 0.00135671254839254

39 49 0.00464054176885498

39 50 0.00543180834202006

39 51 0.00230295554909021

39 52 0.00702163217265839

39 53 0.0104200062557425

39 54 0.0102034901069945

39 55 0.00648778433027908

39 56 0.00319568155846595

39 57 0.00283717994567512

39 58 0.00235229803030745

39 59 0.00317015374152685

39 60 0.00227952218996197

39 61 0.00175676962377924

39 62 0.000575240867003779

39 63 0.00242565265762196

39 64 0.00396493567152045

39 65 0.00102812718753376

39 66 0.000579883632781497

39 67 0

39 68 0

39 69 0.000341619072603994

39 70 0.00199184232770766

39 71 0.00228714583722300

39 72 0.00526878714775858

39 73 0.00384737126138879

39 74 0.00226030800577637

39 75 0.0156884158688921

39 76 0.0251153199171021

40 1 0.00675069936398265

40 2 0.00718271744628762

40 3 0.00877984430362511

40 4 0.00965353291077498

40 5 0.00738052562878211

40 6 0.00470159018289573

40 7 0.00486135690351652

40 8 0.0113858805954504

40 9 0.00948346606130457

40 10 0.00530060267494126

40 11 0.00652929059145291

40 12 0.00718414829846048

40 13 0.00840785609319850

40 14 0.00608939394054453

40 15 0.00612420362773225

40 16 0.00617835955714119

40 17 0.00500062354072883

40 18 0.00559417982195865

40 19 0.00589303817523912

40 20 0.00468359657632567

40 21 0.00471390226648127

40 22 0.00644104373272936

40 23 0.00686144622089646

40 24 0.00685975757837820

40 25 0.00566760176015024

40 26 0.00603983605575587

40 27 0.00600654887738761

40 28 0.00625794707715677

40 29 0.00589549879313178

40 30 0.00586918587527829

40 31 0.00625385322656713

40 32 0.00567701365188922

40 33 0.00621225376654822

40 34 0.00550508742663014

40 35 0.00517792400328365

40 36 0.00625001872926845

40 37 0.00670647706693894

40 38 0.00665049038884114

40 39 0.00598437363924043

40 40 0.00305566536819518

40 41 0.00833416096568151

40 42 0.00602298046278471

40 43 0.00701529519708799

40 44 0.00734648107957170

40 45 0.00763475899004873

40 46 0.00849196073090464

40 47 0.00905211487891888

40 48 0.00650798848542278

40 49 0.00857727817359011

40 50 0.00916783821992673

40 51 0.00796290805123201

40 52 0.00905991061287370

40 53 0.00859446228791794

40 54 0.00948552826527149

40 55 0.00746056915365956

40 56 0.00737622734359034

40 57 0.00702018803453253

40 58 0.00777939727436006

40 59 0.00857202044945371

40 60 0.00760484946876538

40 61 0.00707431911008993

40 62 0.00590817528040044

40 63 0.00768901411785838

40 64 0.00984668744630646

40 65 0.00644427791120605

40 66 0.00595974963513590

40 67 0.00504303667039885

40 68 0.00529922990559961

40 69 0.00570475683385652

40 70 0.00708452686986671

40 71 0.00819018285095308

40 72 0.00661227505339612

40 73 0.00472396676902398

40 74 0.00768661050999298

40 75 0.0210705336754660

40 76 0.0245978835871521

41 1 0.00260790138111466

41 2 0.00217434649605541

41 3 0.00238655493054030

41 4 0.00236081510472363

41 5 0.00225975610453877

41 6 0.000534353662478448

41 7 0.000841065874247815

41 8 0.00733000104761661

41 9 0.00545553999236115

41 10 0.00141827663966809

41 11 0.00206650417619214

41 12 0.00281897513425794

41 13 0.00307967009135002

41 14 0.000289447472466731

41 15 0.00185026922863152

41 16 0.00214579791894520

41 17 0.000827588489747400

41 18 0.00152067787851884

41 19 0.00182051508960429

41 20 0.000528115602727919

41 21 0.000447069636230757

41 22 0.000213667572190657

41 23 0.000465302937366241

41 24 0.000399748251992293

41 25 0.00115543941451135

41 26 0.00128563093059675

41 27 0.000546560884720604

41 28 0.00165991344198729

41 29 0.00164495017995914

41 30 0.00136556593343806

41 31 0.00168885772640420

41 32 0.00163650454464659

41 33 0.000886228990661930

41 34 0.000296156550091853

41 35 0.00100214464488813

41 36 0.00218950434658527

41 37 0.00267355257762109

41 38 0.00259297191295171

41 39 0.000789283659238693

41 40 0.00526299891701765

41 41 0.00128975504194301

41 42 0.00194178505281428

41 43 0.00294555899028193

41 44 0.00291146431663936

41 45 0.00352044727069567

41 46 0.00482483387304078

41 47 0.00489113359111604

41 48 0.00238006229341248

41 49 0.00419955725244847

41 50 0.00410303114411359

41 51 0.00287582609695802

41 52 0.00308022671089203

41 53 0.00131300536678691

41 54 0.00262838881133387

41 55 0.00175611539563281

41 56 0.00304159026011963

41 57 0.00276217568496340

41 58 0.00402083048633495

41 59 0.00466492938945584

41 60 0.00359349584057589

41 61 0.00288015068643510

41 62 0.00183575452079870

41 63 0.00246097760840081

41 64 0.00176489765083732

41 65 0.00186363119023342

41 66 0.00143667651792223

41 67 0.000968936202658810

41 68 0.00123439998037922

41 69 0.00159182755814002

41 70 0.00299799905020148

41 71 0.00335857228334324

41 72 0.00196878072677564

41 73 0.00196177933491670

41 74 0.00337004483462255

41 75 0.0146071368176617

41 76 0.0177012115499386

42 1 0.00513365666394128

42 2 0.00494259096293448

42 3 0.00508448497588854

42 4 0.00498154598911438

42 5 0.00475347926125813

42 6 0.00301554864016695

42 7 0.00328930912574676

42 8 0.00974930905670979

42 9 0.00791655219423082

42 10 0.00362569114370648

42 11 0.00463616470219375

42 12 0.00546197086927358

42 13 0.00575472649031672

42 14 0.00277238265262036

42 15 0.00433243567717656

42 16 0.00484355135475467

42 17 0.00328840171161071

42 18 0.00390008342170722

42 19 0.00428208471315139

42 20 0.00299983037534535

42 21 0.00287727631287760

42 22 0.00380423339524505

42 23 0.00474484923282348

42 24 0.00382813508365365

42 25 0.00368864132411195

42 26 0.00437836629692090

42 27 0.00407033154675862

42 28 0.00466206385856049

42 29 0.00403887731539099

42 30 0.00377564761356197

42 31 0.00436158169772427

42 32 0.00404592289104322

42 33 0.00348612449528007

42 34 0.00270874699286827

42 35 0.00345168327972478

42 36 0.00459357568056046

42 37 0.00467783838530278

42 38 0.00479697282916647

42 39 0.00334784350998663

42 40 0.00808682561522989

42 41 0.00617820182676787

42 42 0

42 43 0.00364284430248751

42 44 0.00485015088211160

42 45 0.00495484965669335

42 46 0.00631303178078568

42 47 0.00716576159384574

42 48 0.00514839765514219

42 49 0.00622864736957918

42 50 0.00612930264960754

42 51 0.00514463567292078

42 52 0.00622146764235265

42 53 0.00621881657589252

42 54 0.00690439553208333

42 55 0.00531245758724008

42 56 0.00554290213514441

42 57 0.00543772010729393

42 58 0.00635399765786593

42 59 0.00662957446169521

42 60 0.00602458650079063

42 61 0.00542862439164560

42 62 0.00431039227111185

42 63 0.00499468061941366

42 64 0.00431332365516912

42 65 0.00435943621884483

42 66 0.00389837476731081

42 67 0.00342548512199692

42 68 0.00374294929990404

42 69 0.00416309070091359

42 70 0.00492460800764150

42 71 0.00511314963634091

42 72 0.00323480361980377

42 73 0.00333757553442782

42 74 0.00612426317364612

42 75 0.0174720735401983

42 76 0.0201817310279977

43 1 0.00608355284342577

43 2 0.00577759057061655

43 3 0.00587800673565586

43 4 0.00582710598842839

43 5 0.00570433623773614

43 6 0.00394516314686189

43 7 0.00421861413008062

43 8 0.0107023113121398

43 9 0.00880162665066153

43 10 0.00454234584717850

43 11 0.00553931506817085

43 12 0.00637947476849665

43 13 0.00658951676967023

43 14 0.00360195235542893

43 15 0.00529316342806863

43 16 0.00573347098574828

43 17 0.00428459157034788

43 18 0.00485550756819175

43 19 0.00515081543795448

43 20 0.00383966017222559

43 21 0.00369968616415584

43 22 0.00381847627162157

43 23 0.00417889384987458

43 24 0.00394901292399308

43 25 0.00448852361800023

43 26 0.00486325316016889

43 27 0.00418980867368379

43 28 0.00514034054946189

43 29 0.00499164152986698

43 30 0.00470181930905678

43 31 0.00519511415018115

43 32 0.00496940947988966

43 33 0.00426245739259257

43 34 0.00355429674380947

43 35 0.00439226953647265

43 36 0.00551522783196751

43 37 0.00546613182897410

43 38 0.00552448160303848

43 39 0.00386307476796843

43 40 0.00902019466684123

43 41 0.00719206872056721

43 42 0.00323859525549963

43 43 0.000547150788298756

43 44 0.00589247101689683

43 45 0.00579862063660597

43 46 0.00719218126217880

43 47 0.00815186236193566

43 48 0.00624399580900736

43 49 0.00716145783033662

43 50 0.00678025887061795

43 51 0.00583026554683830

43 52 0.00624467905481896

43 53 0.00529510430840907

43 54 0.00614119120869505

43 55 0.00498920068645226

43 56 0.00608265845174483

43 57 0.00573119833314917

43 58 0.00673164771288526

43 59 0.00747204330883000

43 60 0.00697162706482557

43 61 0.00626713610169494

43 62 0.00523147979035010

43 63 0.00586809227238594

43 64 0.00511507249753518

43 65 0.00523541642744474

43 66 0.00476980399340066

43 67 0.00447742066424195

43 68 0.00479199549029163

43 69 0.00531993134310704

43 70 0.00589683725265400

43 71 0.00604843919060227

43 72 0.00358498202597568

43 73 0.00452858364930409

43 74 0.00685831324317832

43 75 0.0182086813015419

43 76 0.0208757079469843

44 1 0.00100963966165535

44 2 0.000803956779763637

44 3 0.000758812929324781

44 4 0.000678094897263670

44 5 0.000403199055787895

44 6 0

44 7 0

44 8 0.00545754744658544

44 9 0.00358072854695313

44 10 0

44 11 0.000282099900223098

44 12 0.00106134498094301

44 13 0.00141905114943086

44 14 0

44 15 5.97291498646291e-05

44 16 0.000374142382547626

44 17 0

44 18 0

44 19 1.38835558631811e-05

44 20 0

44 21 0

44 22 0.000204559466451926

44 23 0

44 24 0.000204835922860691

44 25 0

44 26 0.000457060271396803

44 27 0.000234665010760415

44 28 0.000465545290536995

44 29 0

44 30 0

44 31 0.000157427648983854

44 32 0

44 33 0

44 34 0

44 35 0

44 36 0.000384133640981388

44 37 0.00110158518755038

44 38 0.00127412411816136

44 39 6.83041358053860e-05

44 40 0.00406740530069671

44 41 0.00202444804140334

44 42 0.000374364241138236

44 43 0.00114867170245225

44 44 5.41308014251340e-06

44 45 0.000991103697411622

44 46 0.00269971365053801

44 47 0.00327475477840988

44 48 0.000760544421031240

44 49 0.00276464592016523

44 50 0.00269404881407587

44 51 0.000945794552999368

44 52 0.00102568050558971

44 53 4.36145025592971e-05

44 54 0.000646793793720457

44 55 0.000841764231051423

44 56 0.00135555265309883

44 57 0.000910179104510966

44 58 0.00126600357054829

44 59 0.00241081676706001

44 60 0.00169142232832664

44 61 0.000944253998707789

44 62 0

44 63 0.000871334576740268

44 64 0.000202353232996666

44 65 0

44 66 0

44 67 0

44 68 0

44 69 0

44 70 0.00117053213940521

44 71 0.00132920045584117

44 72 0

44 73 0

44 74 0.00138482831796360

44 75 0.0124380540568019

44 76 0.0162447963331367

45 1 0

45 2 0

45 3 0

45 4 0

45 5 0

45 6 0

45 7 0

45 8 0.00342467439506966

45 9 0.00162916611900010

45 10 0

45 11 0

45 12 0

45 13 0.000139957590562623

45 14 0

45 15 0

45 16 0

45 17 0

45 18 0

45 19 0

45 20 0

45 21 0

45 22 0.00222868704912416

45 23 0.000567559024023501

45 24 0.00200841190715584

45 25 0

45 26 0.00160678734895647

45 27 0.00339233808790673

45 28 0.000682869067801684

45 29 0

45 30 0

45 31 0

45 32 0

45 33 0

45 34 0

45 35 0

45 36 0

45 37 0.000540824555288755

45 38 0.000736220628579198

45 39 0

45 40 0.00222458921662838

45 41 0.000117436099769219

45 42 0

45 43 5.98995180083328e-05

45 44 0

45 45 0

45 46 0.00219748069640191

45 47 0.00177379328588301

45 48 0

45 49 0.000980127118564766

45 50 0.000693093766737651

45 51 0

45 52 0.00167716773334003

45 53 0.00104834497126793

45 54 0.00168762424801683

45 55 0.00186091402961830

45 56 0.000423750616861618

45 57 0.000329681426775474

45 58 0.000445629883012444

45 59 0.000724883450920255

45 60 0

45 61 0

45 62 0

45 63 0

45 64 0

45 65 0

45 66 0

45 67 0

45 68 0

45 69 0

45 70 0

45 71 0

45 72 0

45 73 0

45 74 0

45 75 0.0111759050554874

45 76 0.0174819480585282

46 1 0

46 2 0

46 3 0

46 4 0

46 5 0

46 6 0

46 7 0

46 8 0.00351672601750963

46 9 0.00172750427189627

46 10 0

46 11 0

46 12 0

46 13 1.35881219516243e-05

46 14 0

46 15 0

46 16 0

46 17 0

46 18 0

46 19 0

46 20 0

46 21 0

46 22 0

46 23 0

46 24 0

46 25 0

46 26 0

46 27 0.000171440693790492

46 28 0

46 29 0

46 30 0

46 31 0

46 32 0

46 33 0

46 34 0

46 35 0

46 36 0

46 37 0

46 38 0

46 39 0

46 40 0.00226899471869768

46 41 0.000186661842352609

46 42 0

46 43 0

46 44 0

46 45 0

46 46 0.00118423575245746

46 47 0.00160478615202120

46 48 0

46 49 0.000831939342510135

46 50 0.000611683346971037

46 51 0

46 52 0.000160664555371115

46 53 0

46 54 0.000189213880089634

46 55 0

46 56 0

46 57 0

46 58 0

46 59 0.000376113326860938

46 60 0

46 61 0

46 62 0

46 63 0

46 64 0

46 65 0

46 66 0

46 67 0

46 68 0

46 69 0

46 70 0

46 71 0

46 72 0

46 73 0

46 74 0

46 75 0.0104635067339696

46 76 0.0138776712332302

47 1 0

47 2 0

47 3 0

47 4 0

47 5 0

47 6 0

47 7 0

47 8 0.00412896351510605

47 9 0.00231531983465860

47 10 0

47 11 0

47 12 0

47 13 0.000164813140059450

47 14 0

47 15 0

47 16 0

47 17 0

47 18 0

47 19 0

47 20 0

47 21 0

47 22 0

47 23 0

47 24 0

47 25 0

47 26 0

47 27 0

47 28 0

47 29 0

47 30 0

47 31 0

47 32 0

47 33 0

47 34 0

47 35 0

47 36 0

47 37 0

47 38 0

47 39 0

47 40 0.00269095630937255

47 41 0.000788470281927967

47 42 0

47 43 0.000117401484286961

47 44 0

47 45 0

47 46 0.00117608641712896

47 47 0.00213958284640897

47 48 0

47 49 0.00147986829344371

47 50 0.00122775234576145

47 51 0

47 52 0

47 53 0

47 54 0

47 55 0

47 56 0

47 57 0

47 58 0

47 59 0.000819506084890787

47 60 0.000393422696854606

47 61 0

47 62 0

47 63 0

47 64 0

47 65 0

47 66 0

47 67 0

47 68 0

47 69 0

47 70 0

47 71 0.000224615389290084

47 72 0

47 73 0

47 74 0

47 75 0.0108981950394738

47 76 0.0132812277060912

48 1 0.00195524940732172

48 2 0.00164075724966628

48 3 0.00189211665602895

48 4 0.00187727248556879

48 5 0.00166022825266199

48 6 0

48 7 0.000237377303247466

48 8 0.00662684793976120

48 9 0.00484839505854773

48 10 0.000589083968692283

48 11 0.00151636183057347

48 12 0.00228961058216226

48 13 0.00253659265615969

48 14 0

48 15 0.00137672776893072

48 16 0.00165035719145890

48 17 0.000482925765718845

48 18 0.000880889171106958

48 19 0.00130547761002442

48 20 0

48 21 0

48 22 0

48 23 0

48 24 0

48 25 0.000561170244348097

48 26 0.000643243786902281

48 27 0

48 28 0.00114333538453104

48 29 0.00103428673821104

48 30 0.000767037561600681

48 31 0.00114359405978870

48 32 0.00101437162055762

48 33 0.000251110145575661

48 34 0

48 35 0.000442202940370007

48 36 0.00153973129892315

48 37 0.00156227287389654

48 38 0.00161870633592864

48 39 0

48 40 0.00508428213952550

48 41 0.00327180974681363

48 42 0.00158105887877102

48 43 0.00250350342267358

48 44 0.00215824920233909

48 45 0.00219687796348778

48 46 0.00321320575614925

48 47 0.00399365817489228

48 48 0

48 49 0.00380048354109686

48 50 0.00394888933582771

48 51 0.00222429995442486

48 52 0.00149959968616842

48 53 0.000430422812254028

48 54 0.00134839751450433

48 55 0.000863185176184667

48 56 0.00202965949562506

48 57 0.00162402500148917

48 58 0.00235518010187619

48 59 0.00345942313841241

48 60 0.00298361493632093

48 61 0.00226133263318784

48 62 0.00125875432220990

48 63 0.00194176171760707

48 64 0.00117703301964534

48 65 0.00126480849942978

48 66 0.000802704119381326

48 67 0.000339175676779657

48 68 0.000578700924070419

48 69 0.000978548024204162

48 70 0.00236687453852025

48 71 0.00273045176418929

48 72 0

48 73 0.000576224662923730

48 74 0.00240417296675777

48 75 0.0141073209020830

48 76 0.0164112616548773

49 1 0.00493709261078013

49 2 0.00465270953424890

49 3 0.00460219891303626

49 4 0.00448835586648178

49 5 0.00440803226502917

49 6 0.00264513221413709

49 7 0.00300435740827176

49 8 0.00944183296605616

49 9 0.00759273238643254

49 10 0.00329037949854483

49 11 0.00429412532008822

49 12 0.00505437003461656

49 13 0.00526956887692387

49 14 0.00230177992109382

49 15 0.00411714152909903

49 16 0.00441905166020840

49 17 0.00308137702468425

49 18 0.00357363572735891

49 19 0.00436696707370460

49 20 0.00280753637681364

49 21 0.00283005674341041

49 22 0.00399769813066908

49 23 0.00406276305656916

49 24 0.00401555056363812

49 25 0.00373981402046131

49 26 0.00395803274232065

49 27 0.00357930362762005

49 28 0.00433471082237691

49 29 0.00381575237290077

49 30 0.00362163648734093

49 31 0.00418395623898060

49 32 0.00379372498705421

49 33 0.00327333188575718

49 34 0.00259633750693356

49 35 0.00328066773008284

49 36 0.00429719454743721

49 37 0.00429556558240457

49 38 0.00440725975920303

49 39 0.00359396543846124

49 40 0.00813406117940652

49 41 0.00589764172213336

49 42 0.00374452816138926

49 43 0.00461360933529065

49 44 0.00520210631155305

49 45 0.00469341505998711

49 46 0.00605022432588065

49 47 0.00706513515531904

49 48 0.00482134260255407

49 49 0.00164740141731479

49 50 0.00583264789257088

49 51 0.00507095623871823

49 52 0.00522772429412743

49 53 0.00470621468810606

49 54 0.00521397317087247

49 55 0.00476651097318670

49 56 0.00529229047226154

49 57 0.00478553854017860

49 58 0.00553244963071542

49 59 0.00647857704679977

49 60 0.00582701176212863

49 61 0.00504424425212047

49 62 0.00399678852228691

49 63 0.00466699499255507

49 64 0.00392023672199149

49 65 0.00398028262926004

49 66 0.00352906532321318

49 67 0.00313304754955379

49 68 0.00346738177861328

49 69 0.00393152417371800

49 70 0.00516955809615394

49 71 0.00544215080104271

49 72 0.00206960197972905

49 73 0.00355689618830191

49 74 0.00611422498591385

49 75 0.0195003734962613

49 76 0.0212395232311133

50 1 0.00642386369934456

50 2 0.00619141917006771

50 3 0.00603879635498028

50 4 0.00592833363397971

50 5 0.00594977654942352

50 6 0.00415917615571093

50 7 0.00449619387918154

50 8 0.0110305408914223

50 9 0.00915882814873181

50 10 0.00490287497572839

50 11 0.00582512269343716

50 12 0.00662216318748154

50 13 0.00680771456927887

50 14 0.00383643466820449

50 15 0.00560025604066783

50 16 0.00602483800833842

50 17 0.00457701257681664

50 18 0.00517092300461408

50 19 0.00573991492137993

50 20 0.00427605072944515

50 21 0.00436930830627436

50 22 0.00648713787126498

50 23 0.00576349307783575

50 24 0.00643749651506120

50 25 0.00525053410947629

50 26 0.00574618326771381

50 27 0.00568880029412100

50 28 0.00604474743256211

50 29 0.00537728939278148

50 30 0.00512476015699415

50 31 0.00585873605653309

50 32 0.00533892522506163

50 33 0.00503628519346000

50 34 0.00426283702111996

50 35 0.00478378299876936

50 36 0.00587743015905617

50 37 0.00613922842493442

50 38 0.00628218300370886

50 39 0.00633898645820863

50 40 0.0101171471182515

50 41 0.00755377215219688

50 42 0.00549552831622391

50 43 0.00606247892460476

50 44 0.00777515455678191

50 45 0.00721286523359810

50 46 0.00785236075640028

50 47 0.00871906702835879

50 48 0.00705869884680066

50 49 0.00787952555884175

50 50 0.00149779693805246

50 51 0.00578618953998655

50 52 0.00727406201448599

50 53 0.00642096199856446

50 54 0.00651758487976517

50 55 0.00678998247331553

50 56 0.00719454104723105

50 57 0.00674991796168345

50 58 0.00795226341605670

50 59 0.00840304251688484

50 60 0.00753072062094962

50 61 0.00665546838423614

50 62 0.00555803283469813

50 63 0.00612287386083055

50 64 0.00548159983965268

50 65 0.00556107002617823

50 66 0.00513359636285182

50 67 0.00474181167697307

50 68 0.00512534694511269

50 69 0.00558836796331796

50 70 0.00680870580636517

50 71 0.00701413353673275

50 72 0.00423734202148607

50 73 0.00544666338159583

50 74 0.0102806691795065

50 75 0.0212424342118043

50 76 0.0237043112679640

51 1 0.00453879477379016

51 2 0.00441449252273207

51 3 0.00444274685298396

51 4 0.00455896697282365

51 5 0.00444611158416741

51 6 0.00260622227263674

51 7 0.00278950645592924

51 8 0.00918218748261335

51 9 0.00734738821193137

51 10 0.00308503272783878

51 11 0.00409682681848855

51 12 0.00479542089349760

51 13 0.00509073076019129

51 14 0.00237069525411737

51 15 0.00374233644626820

51 16 0.00420704452260168

51 17 0.00289601572648779

51 18 0.00350099920284308

51 19 0.00369360791269369

51 20 0.00246518246185867

51 21 0.00243679305675215

51 22 0.00416341989062086

51 23 0.00323649260202896

51 24 0.00441116915114015

51 25 0.00331950947834536

51 26 0.00362512990225916

51 27 0.00322117444913628

51 28 0.00393893360954656

51 29 0.00352065437292126

51 30 0.00328191428952895

51 31 0.00384331241415858

51 32 0.00349446043606039

51 33 0.00272850271924729

51 34 0.00220955362060515

51 35 0.00296159559753606

51 36 0.00420032410982829

51 37 0.00516899363693468

51 38 0.00524377247071373

51 39 0.00478005409580307

51 40 0.00919153701103037

51 41 0.00620588435424829

51 42 0.00442919958057364

51 43 0.00515652858825033

51 44 0.00563026616432116

51 45 0.00631354561286912

51 46 0.00680446145868674

51 47 0.00697072266668275

51 48 0.00476027148035951

51 49 0.00678477818208967

51 50 0.00579206914069030

51 51 0

51 52 0.00726594352688026

51 53 0.00651949715469447

51 54 0.00773371289711156

51 55 0.00493291635575610

51 56 0.00513368298834616

51 57 0.00491709956271336

51 58 0.00659472997378652

51 59 0.00659192928162655

51 60 0.00570942522256401

51 61 0.00492329036594230

51 62 0.00375768469598015

51 63 0.00426129527137475

51 64 0.00370716556854156

51 65 0.00378454455844901

51 66 0.00340392341668827

51 67 0.00298025097515475

51 68 0.00346349878591778

51 69 0.00364000623122762

51 70 0.00499358548491780

51 71 0.00539455149919232

51 72 0.00418769759670412

51 73 0.00505836976460106

51 74 0.00958758796557901

51 75 0.0172527423096751

51 76 0.0200854466727951

52 1 0.00132486352972716

52 2 0.00175804249795042

52 3 0.00143365607415991

52 4 0.00142821539408122

52 5 0.000935817919482274

52 6 0

52 7 0

52 8 0.00581397646193582

52 9 0.00384456525091226

52 10 0

52 11 0.000578006972635325

52 12 0.00134614480979667

52 13 0.00221424937799775

52 14 0.000948674950936201

52 15 0.000156772330452526

52 16 0.000487544735024326

52 17 0

52 18 0

52 19 0.00114410647700114

52 20 0.00300121539979437

52 21 0.00316151913653373

52 22 0.00668318469618834

52 23 0.00734832055410251

52 24 0.00724306421175314

52 25 0.00349217130470936

52 26 0.00655405817649157

52 27 0.00938237372737194

52 28 0.00630095939610242

52 29 0.000178790557610942

52 30 0

52 31 0.00222536476509871

52 32 0

52 33 0

52 34 0

52 35 0

52 36 0.000943910282913185

52 37 0.00490163557541212

52 38 0.00502855127847390

52 39 0.00973188615390175

52 40 0.00999124849479727

52 41 0.00467939303101494

52 42 0.00157846521603577

52 43 0.00187933421064712

52 44 0.00385170106994104

52 45 0.00793845761170897

52 46 0.00599297696012580

52 47 0.00392180218290805

52 48 0.000931565807320345

52 49 0.00447149317128504

52 50 0.00535897845782074

52 51 0.00351235616965573

52 52 0

52 53 0.00224393111075627

52 54 0.00122913477883491

52 55 0.00463252125116065

52 56 0.00726576979411753

52 57 0.00721948563369257

52 58 0.00639263112750332

52 59 0.00685560135648111

52 60 0.00252400010259390

52 61 0.00126801240837660

52 62 0.000237711200763990

52 63 0.00119653277048359

52 64 0.00176132707048682

52 65 0.000273184986368968

52 66 0

52 67 0

52 68 0.000934402414338065

52 69 0.00111864598500855

52 70 0.00194533363128158

52 71 0.00169520118115973

52 72 0.00983822620164498

52 73 0.0108712177826409

52 74 0.00818885068393604

52 75 0.0140484506143668

52 76 0.0215289173695823

53 1 0

53 2 0.000182764857288520

53 3 0

53 4 0

53 5 0

53 6 0

53 7 0

53 8 0.00438724309365224

53 9 0.00211173247948304

53 10 0

53 11 0

53 12 0

53 13 0.000604540784458152

53 14 0

53 15 0

53 16 0

53 17 0

53 18 0

53 19 0.000829287660625400

53 20 0.00675131295756115

53 21 0.00656478152086004

53 22 0.0137675932169881

53 23 0.0187444693882761

53 24 0.0148066357872061

53 25 0.00597319215842673

53 26 0.00961754123565783

53 27 0.0164622451329588

53 28 0.00935609622101019

53 29 0

53 30 0

53 31 0.00193543701144216

53 32 0

53 33 0

53 34 0

53 35 0

53 36 0

53 37 0.00308211034474792

53 38 0.00348818148281338

53 39 0.0120307059315212

53 40 0.00895689505454422

53 41 0.00270301535326434

53 42 0.000675246149783648

53 43 0.000176835563244770

53 44 0.00212980934117035

53 45 0.00545799633946986

53 46 0.00392912826819658

53 47 0.00205312508457101

53 48 0

53 49 0.00378760472288953

53 50 0.00510901486964355

53 51 0.00188631636449732

53 52 0.00970939810253113

53 53 0

53 54 0.00393385034583615

53 55 0.00947310628389819

53 56 0.00885250274340610

53 57 0.00928886159217890

53 58 0.00594784013602556

53 59 0.00520244363224487

53 60 0.000592983666286573

53 61 0

53 62 0

53 63 0

53 64 0.000562463283291459

53 65 0

53 66 0

53 67 0

53 68 0

53 69 0

53 70 0.000496448346585021

53 71 2.59049014952328e-05

53 72 0.0154861955843426

53 73 0.0136391010732846

53 74 0.00984564420159573

53 75 0.0141297247127705

53 76 0.0242307448799559

54 1 0.000489181022510055

54 2 0.00116457911287093

54 3 0.000410134082367009

54 4 0.000310908994315184

54 5 0

54 6 0

54 7 0

54 8 0.00507840123867087

54 9 0.00298426186259171

54 10 0

54 11 0

54 12 0.000386120416242308

54 13 0.00140431293701937

54 14 4.59775702728038e-05

54 15 0

54 16 0

54 17 0

54 18 0

54 19 0.000996096467248075

54 20 0.00557320960077234

54 21 0.00549804529157982

54 22 0.0123155791435523

54 23 0.0155241830736599

54 24 0.0132935913826462

54 25 0.00546314081992350

54 26 0.00995403261616756

54 27 0.0158407023353939

54 28 0.00922573098382584

54 29 0

54 30 0

54 31 0.00278521467783510

54 32 0

54 33 0

54 34 0

54 35 0

54 36 6.50702510351930e-06

54 37 0.00455001818660594

54 38 0.00476020538799438

54 39 0.0124941581375422

54 40 0.00965067766024808

54 41 0.00375898863395663

54 42 0.00136159526207957

54 43 0.000937054830181250

54 44 0.00355371119082082

54 45 0.00788162314370333

54 46 0.00537500037837702

54 47 0.00296563773693714

54 48 0.000107140732401811

54 49 0.00455274142318651

54 50 0.00553520467241353

54 51 0.00290945561165090

54 52 0.00865860690863141

54 53 0.00135048404311067

54 54 0

54 55 0.00790085273785435

54 56 0.00949624743082000

54 57 0.00980797314714277

54 58 0.00708101308516596

54 59 0.00658572399181234

54 60 0.00152862295553868

54 61 0.000360094748927953

54 62 0

54 63 0.000345493005375230

54 64 0.00116548102257363

54 65 0

54 66 0

54 67 0

54 68 0.000476872500057457

54 69 0.000705283051393391

54 70 0.00133157803346906

54 71 0.000788550969267998

54 72 0.0152607859201390

54 73 0.0130174236671763

54 74 0.0103205610184787

54 75 0.0145918530154940

54 76 0.0247628677300451

55 1 0

55 2 0

55 3 0

55 4 0

55 5 0

55 6 0

55 7 0

55 8 0.00314170371093880

55 9 0.000966179937433087

55 10 0

55 11 0

55 12 0

55 13 0

55 14 0

55 15 0

55 16 0

55 17 0

55 18 0

55 19 0

55 20 0.00126721235570992

55 21 0.00113032312664796

55 22 0.00331638810539014

55 23 0.00867142448603464

55 24 0.00588149765099844

55 25 0.00259991923232306

55 26 0.00652238833847074

55 27 0.0101750394121054

55 28 0.00622249884006343

55 29 0

55 30 0

55 31 0.000762967558756711

55 32 0

55 33 0

55 34 0

55 35 0

55 36 0

55 37 0.00143755463584971

55 38 0.00144271693323916

55 39 0.00540787079009908

55 40 0.00601896099959887

55 41 0.00307537781184342

55 42 0

55 43 0

55 44 0.00143409199509770

55 45 0.00560565132639035

55 46 0.00276763925469092

55 47 0.000762385424150192

55 48 0

55 49 0.00163413498779263

55 50 0.00306179132203921

55 51 0.00262859511949554

55 52 0.00818182384844257

55 53 0.00158897818502912

55 54 0.00254182825941607

55 55 0

55 56 0.00647529904112432

55 57 0.00619340377421995

55 58 0.00599481756320675

55 59 0.00557384543723516

55 60 0

55 61 0

55 62 0

55 63 0

55 64 0

55 65 0

55 66 0

55 67 0

55 68 0

55 69 0

55 70 0

55 71 0

55 72 0.0107463303410353

55 73 0.0122535209085248

55 74 0.00609905306364955

55 75 0.0119450895613273

55 76 0.0188188109244309

56 1 0

56 2 0

56 3 0

56 4 0

56 5 0

56 6 0

56 7 0

56 8 0.00309272069557198

56 9 0.00121195065169621

56 10 0

56 11 0

56 12 0

56 13 0

56 14 0

56 15 0

56 16 0

56 17 0

56 18 0

56 19 0

56 20 0

56 21 0

56 22 0

56 23 0

56 24 0

56 25 0

56 26 0.000475949326241731

56 27 0.000751990250231094

56 28 0.000680432956232879

56 29 0

56 30 0

56 31 0

56 32 0

56 33 0

56 34 0

56 35 0

56 36 0

56 37 0

56 38 0

56 39 0

56 40 0.00293632344291184

56 41 0.00121666782043572

56 42 0

56 43 0

56 44 0.000345935082815996

56 45 0.00193605553602427

56 46 0.00103248718951776

56 47 0.000742926398547450

56 48 0

56 49 0.000739515213616371

56 50 0.00112650473445974

56 51 5.37617307070359e-05

56 52 0.000390619791874158

56 53 0

56 54 0

56 55 0

56 56 3.17703448898810e-06

56 57 0

56 58 0

56 59 0.00224147301927846

56 60 0

56 61 0

56 62 0

56 63 0

56 64 0

56 65 0

56 66 0

56 67 0

56 68 0

56 69 0

56 70 0

56 71 0

56 72 0

56 73 0.000706926320990009

56 74 0.00172245992629461

56 75 0.0108483596076163

56 76 0.0136724121494458

57 1 0

57 2 0

57 3 0

57 4 0

57 5 0

57 6 0

57 7 0

57 8 0.00296286117747380

57 9 0.00104269617885666

57 10 0

57 11 0

57 12 0

57 13 0

57 14 0

57 15 0

57 16 0

57 17 0

57 18 0

57 19 0

57 20 0

57 21 0

57 22 0

57 23 0.000651288423051843

57 24 0

57 25 0

57 26 0.00212996165515911

57 27 0.00314452904039830

57 28 0.00221384735080121

57 29 0

57 30 0

57 31 0

57 32 0

57 33 0

57 34 0

57 35 0

57 36 0

57 37 0.000594980552514501

57 38 0.000707179653693602

57 39 0

57 40 0.00350665054614863

57 41 0.00206114333881624

57 42 0

57 43 0

57 44 0.00114580303859557

57 45 0.00418904828027555

57 46 0.00195849572645734

57 47 0.000710987695722531

57 48 0

57 49 0.000643259842640243

57 50 0.00127743885422449

57 51 0.000982322023560522

57 52 0.00272561427912177

57 53 0

57 54 0.000552384169882014

57 55 0

57 56 0.00218284805833890

57 57 0

57 58 0.00131236262550716

57 59 0.00368578340338288

57 60 0

57 61 0

57 62 0

57 63 0

57 64 0

57 65 0

57 66 0

57 67 0

57 68 0

57 69 0

57 70 0

57 71 0

57 72 0.00292788957870149

57 73 0.00375731409187607

57 74 0.00324364251312848

57 75 0.0107230388738813

57 76 0.0143549623709258

58 1 0

58 2 0

58 3 0

58 4 0

58 5 0

58 6 0

58 7 0

58 8 0.00221366289234304

58 9 0.000428284112252153

58 10 0

58 11 0

58 12 0

58 13 0

58 14 0

58 15 0

58 16 0

58 17 0

58 18 0

58 19 0

58 20 0

58 21 0

58 22 0.000559295030211571

58 23 0.000661894289123000

58 24 0.000864018481813722

58 25 0

58 26 0.00227712882278897

58 27 0.00424261157561934

58 28 0.00242583518769712

58 29 0

58 30 0

58 31 0

58 32 0

58 33 0

58 34 0

58 35 0

58 36 0

58 37 0.00200537008568119

58 38 0.00225059843281755

58 39 0.000615519026972211

58 40 0.00280546280564242

58 41 0.00143144314208632

58 42 0

58 43 0

58 44 0.00171171829516892

58 45 0.00669840163971980

58 46 0.00297084441764961

58 47 0.000490392401492290

58 48 0

58 49 0

58 50 0.000392839524268496

58 51 0.000352539559911436

58 52 0.00237218816364915

58 53 0

58 54 0

58 55 0

58 56 0.00194476326544779

58 57 0.00147123748707534

58 58 5.58974466927653e-05

58 59 0.00427497994041448

58 60 0

58 61 0

58 62 0

58 63 0

58 64 0

58 65 0

58 66 0

58 67 0

58 68 0

58 69 0

58 70 0

58 71 0

58 72 0.00332104939878075

58 73 0.00256509402867522

58 74 0.00321235998666980

58 75 0.00994799483101994

58 76 0.0155135334199878

59 1 0.000500735559476384

59 2 0.000372982454586390

59 3 0.000198929597904907

59 4 8.35148108085937e-05

59 5 0

59 6 0

59 7 0

59 8 0.00477419016091862

59 9 0.00303627373507298

59 10 0

59 11 0

59 12 0.000469448562276598

59 13 0.000807558765740901

59 14 0

59 15 0

59 16 0

59 17 0

59 18 0

59 19 0

59 20 0

59 21 0

59 22 0

59 23 0

59 24 0

59 25 0

59 26 3.44188788548028e-05

59 27 0

59 28 0.000233953980254609

59 29 0

59 30 0

59 31 0

59 32 0

59 33 0

59 34 0

59 35 0

59 36 3.55768522026789e-05

59 37 0.00156490050325947

59 38 0.00183030160666170

59 39 9.02245841327698e-05

59 40 0.00382060050890565

59 41 0.00164429613534045

59 42 7.36929779125983e-05

59 43 0.000842489729193652

59 44 0.00175964649001181

59 45 0.00288262720992749

59 46 0.00280136547830800

59 47 0.00270505640221175

59 48 0.000175902803650541

59 49 0.00245002186902732

59 50 0.00230514807677840

59 51 0.000593101355610526

59 52 0

59 53 0

59 54 0

59 55 0

59 56 0.000712195325992759

59 57 0.000260075007568639

59 58 0.00204471289821884

59 59 0.00133982252523535

59 60 0.00139946256956691

59 61 0.000447635253549294

59 62 0

59 63 8.93483973903875e-05

59 64 0

59 65 0

59 66 0

59 67 0

59 68 0

59 69 0

59 70 0.000642206970261516

59 71 0.000937540669652348

59 72 0

59 73 0

59 74 0.00278170218819662

59 75 0.0126121921558413

59 76 0.0158169763048195

60 1 3.56048325996783e-05

60 2 0

60 3 1.68850551609534e-05

60 4 3.51413125254663e-05

60 5 0

60 6 0

60 7 0

60 8 0.00468535516234381

60 9 0.00291260534060500

60 10 0

60 11 0

60 12 0.000438569515782139

60 13 0.000624131261239502

60 14 0

60 15 0

60 16 0

60 17 0

60 18 0

60 19 0

60 20 0

60 21 0

60 22 0

60 23 0

60 24 0

60 25 0

60 26 0

60 27 0

60 28 0

60 29 0

60 30 0

60 31 0

60 32 0

60 33 0

60 34 0

60 35 0

60 36 0

60 37 0

60 38 0.000101899039744557

60 39 0

60 40 0.00328673974145921

60 41 0.00140310050870052

60 42 0

60 43 0.000707949557707699

60 44 0.000413693934264048

60 45 0.000322245977690261

60 46 0.00160422751312950

60 47 0.00231297489474080

60 48 0

60 49 0.00188495674140776

60 50 0.00186561372899985

60 51 0.000376209100584668

60 52 0

60 53 0

60 54 0

60 55 0

60 56 0.000308965983240483

60 57 4.24826306902881e-05

60 58 0.00142522142693458

60 59 0.00200686834919928

60 60 0.000915582565547357

60 61 0.000408865376831713

60 62 0

60 63 0

60 64 0

60 65 0

60 66 0

60 67 0

60 68 0

60 69 0

60 70 0.000479706066327634

60 71 0.000858123219211304

60 72 0

60 73 0

60 74 0.00154824628738248

60 75 0.0120084707741053

60 76 0.0140781925245914

61 1 0

61 2 0

61 3 0

61 4 0

61 5 0

61 6 0

61 7 0

61 8 0.00421290127053953

61 9 0.00243314005645412

61 10 0

61 11 0

61 12 0

61 13 0.000194913019013421

61 14 0

61 15 0

61 16 0

61 17 0

61 18 0

61 19 0

61 20 0

61 21 0

61 22 0

61 23 0

61 24 0

61 25 0

61 26 0

61 27 0

61 28 0

61 29 0

61 30 0

61 31 0

61 32 0

61 33 0

61 34 0

61 35 0

61 36 0

61 37 0

61 38 0

61 39 0

61 40 0.00287615358317228

61 41 0.000890201130694601

61 42 0

61 43 0.000217391516637133

61 44 0

61 45 0

61 46 0.000765047536115060

61 47 0.00171586726981754

61 48 0

61 49 0.00124496816734743

61 50 0.00128337818518887

61 51 0

61 52 0

61 53 0

61 54 0

61 55 0

61 56 0

61 57 0

61 58 0.000160235333260705

61 59 0.00120588946076439

61 60 0.000625267392576401

61 61 0.000257233607025509

61 62 0

61 63 0

61 64 0

61 65 0

61 66 0

61 67 0

61 68 0

61 69 0

61 70 3.95948526554779e-06

61 71 0.000374767409903498

61 72 0

61 73 0

61 74 0.000355906278502127

61 75 0.0111654098339291

61 76 0.0132849806979467

62 1 0

62 2 0

62 3 0

62 4 0

62 5 0

62 6 0

62 7 0

62 8 0.00416168211202184

62 9 0.00235724944496940

62 10 0

62 11 0

62 12 0

62 13 2.75754054048227e-05

62 14 0

62 15 0

62 16 0

62 17 0

62 18 0

62 19 0

62 20 0

62 21 0

62 22 0

62 23 0

62 24 0

62 25 0

62 26 0

62 27 0

62 28 0

62 29 0

62 30 0

62 31 0

62 32 0

62 33 0

62 34 0

62 35 0

62 36 0

62 37 0

62 38 0

62 39 0

62 40 0.00258595497899683

62 41 0.000797814436850564

62 42 0

62 43 8.45987869654286e-05

62 44 0

62 45 0

62 46 0.000676802482846517

62 47 0.00162018960036514

62 48 0

62 49 0.00117799104317773

62 50 0.00108585111421011

62 51 0

62 52 0

62 53 0

62 54 0

62 55 0

62 56 0

62 57 0

62 58 0

62 59 0.000854220701905550

62 60 0.000488811091536423

62 61 6.01841722469576e-06

62 62 0

62 63 0

62 64 0

62 65 0

62 66 0

62 67 0

62 68 0

62 69 0

62 70 0

62 71 0.000245307380689730

62 72 0

62 73 0

62 74 0

62 75 0.0107914629374281

62 76 0.0125494468703122

63 1 0.00281415493498671

63 2 0.00225843195294972

63 3 0.00272949128647437

63 4 0.00302276187431438

63 5 0.00304486649766933

63 6 0.00123738617719926

63 7 0.00145765681708010

63 8 0.00790960665136620

63 9 0.00608755789400162

63 10 0.00172815231386725

63 11 0.00280060737043852

63 12 0.00354025045465195

63 13 0.00349697419798134

63 14 0.000374284484431220

63 15 0.00233402995436061

63 16 0.00269999591491210

63 17 0.00161763768572265

63 18 0.00218867342087736

63 19 0.00241614688045222

63 20 0.00119372151661257

63 21 0.00112988397148794

63 22 0.000783302205781045

63 23 0.00103618720258303

63 24 0.00109561509836165

63 25 0.00181422357579064

63 26 0.00201863731611224

63 27 0.00113445971533677

63 28 0.00236636552858827

63 29 0.00235551297315112

63 30 0.00209975357036551

63 31 0.00250327358274521

63 32 0.00231866254668922

63 33 0.00151359614755875

63 34 0.00100749033944503

63 35 0.00176806529682683

63 36 0.00279263196143298

63 37 0.00276573492609911

63 38 0.00281397733398320

63 39 0.00169793945150687

63 40 0.00669817498552738

63 41 0.00443463559624324

63 42 0.00292346668020171

63 43 0.00377000192237520

63 44 0.00326801989074299

63 45 0.00280097937509044

63 46 0.00450268380481489

63 47 0.00539854897484249

63 48 0.00306345170336864

63 49 0.00487900339436842

63 50 0.00462072816582704

63 51 0.00288639250892986

63 52 0.00263007955075334

63 53 0.00188707036816860

63 54 0.00258865705841682

63 55 0.00201775921471392

63 56 0.00322063028843167

63 57 0.00277335054732975

63 58 0.00358159342780806

63 59 0.00457331949845974

63 60 0.00416722103257960

63 61 0.00354934399018270

63 62 0.00254783580653367

63 63 0

63 64 0.000660697412472433

63 65 0.00220811820666694

63 66 0.00184975227718021

63 67 0.00157216986337649

63 68 0.00176723103042464

63 69 0.00215885086510098

63 70 0.00360922905530203

63 71 0.00393818844540783

63 72 0

63 73 0.000109660055391259

63 74 0.00295013998913574

63 75 0.0147141293150854

63 76 0.0175614152687584

64 1 0.00678677441845033

64 2 0.00617887896196789

64 3 0.00706280138934612

64 4 0.00803750250133162

64 5 0.00742808852829002

64 6 0.00555069369850569

64 7 0.00544277050581865

64 8 0.0118977665394110

64 9 0.0100574807037975

64 10 0.00567058784516172

64 11 0.00683146529799961

64 12 0.00747201055355085

64 13 0.00766621748158380

64 14 0.00454786220398817

64 15 0.00628578326670970

64 16 0.00684296672487816

64 17 0.00581599625534401

64 18 0.00613220372451484

64 19 0.00633292436088329

64 20 0.00549610166624492

64 21 0.00539285889101915

64 22 0.00579585574348784

64 23 0.00665119953756743

64 24 0.00660214280831195

64 25 0.00593189497041569

64 26 0.00616962043926150

64 27 0.00549525512419902

64 28 0.00646797191996074

64 29 0.00627397901298521

64 30 0.00603529773209599

64 31 0.00647045970256333

64 32 0.00623223330243583

64 33 0.00579905524154889

64 34 0.00523623027044595

64 35 0.00572702490924038

64 36 0.00676009847899572

64 37 0.00679594556296914

64 38 0.00683288921857073

64 39 0.00803623359152250

64 40 0.0134746453582822

64 41 0.00855764898212641

64 42 0.00689395002624893

64 43 0.00763348242753026

64 44 0.00724854886132142

64 45 0.00683776031764150

64 46 0.00860964845934464

64 47 0.00937565874563562

64 48 0.00706335023278315

64 49 0.00888412702985530

64 50 0.00871326914243542

64 51 0.00689967565304772

64 52 0.00797527968154266

64 53 0.00695598106437867

64 54 0.00756787605753906

64 55 0.00652568591153391

64 56 0.00731502481502611

64 57 0.00687663669167871

64 58 0.00760737466539707

64 59 0.00856455698977410

64 60 0.00817816628940571

64 61 0.00754004703933120

64 62 0.00653491755923241

64 63 0.00561082028672011

64 64 0

64 65 0.00624262429471523

64 66 0.00590560924842087

64 67 0.00548024236425571

64 68 0.00574265845216260

64 69 0.00612609444346091

64 70 0.00772659140688425

64 71 0.00809831952579765

64 72 0.00420972522740337

64 73 0.00424509969050912

64 74 0.00687021941585564

64 75 0.0196532717552309

64 76 0.0233229438731699

65 1 0.00128528242021259

65 2 0.000908723895695018

65 3 0.00119775909343400

65 4 0.00122336012222396

65 5 0.00109262547812372

65 6 0

65 7 0

65 8 0.00603480163363324

65 9 0.00422091426112009

65 10 0

65 11 0.000907513810904426

65 12 0.00165433653883057

65 13 0.00181104521321357

65 14 0

65 15 0.000632791488610729

65 16 0.000997574872481644

65 17 0

65 18 0.000265274494528511

65 19 0.000584966019662347

65 20 0

65 21 0

65 22 0

65 23 0

65 24 0

65 25 0

65 26 0.000106758711603927

65 27 0

65 28 0.000514933977374066

65 29 0.000399964135308017

65 30 0.000124304044291701

65 31 0.000572087297972201

65 32 0.000392357241659591

65 33 0

65 34 0

65 35 0

65 36 0.000959598977461423

65 37 0.000879725600730658

65 38 0.000965136069341177

65 39 0

65 40 0.00458549261188668

65 41 0.00267698215589623

65 42 0.00105490851321233

65 43 0.00191305659424734

65 44 0.00139112040951983

65 45 0.000901262135066749

65 46 0.00255273280855800

65 47 0.00348135772579128

65 48 0.00118039784826829

65 49 0.00302986376984815

65 50 0.00287901670503377

65 51 0.00115963955814979

65 52 0.000423031253498185

65 53 0

65 54 0.000225221789046159

65 55 0

65 56 0.00141113346133637

65 57 0.000958741393598639

65 58 0.00160176153613367

65 59 0.00285171730095157

65 60 0.00239121777271145

65 61 0.00168257015454443

65 62 0.000699382116659070

65 63 0.000982783292197809

65 64 0.000150750685339096

65 65 0

65 66 0

65 67 0

65 68 0

65 69 0.000317910213802175

65 70 0.00178036378839674

65 71 0.00209011735025161

65 72 0

65 73 0

65 74 0.000943393523472835

65 75 0.0129166984705860

65 76 0.0149921732196925

66 1 0.00178582364750557

66 2 0.00146381378231752

66 3 0.00194636912499213

66 4 0.00190379078963832

66 5 0.00165830850458748

66 6 0

66 7 0.000239233701607389

66 8 0.00655891133609721

66 9 0.00490074266045149

66 10 0.000416448853239215

66 11 0.00143807936319862

66 12 0.00216370622141909

66 13 0.00238239649320859

66 14 0

66 15 0.00118519600830291

66 16 0.00145042936769757

66 17 0.000249281560174564

66 18 0.000772829999990343

66 19 0.00112002071535167

66 20 0

66 21 0

66 22 0

66 23 0

66 24 0

66 25 0.000491663462400482

66 26 0.000615828915985883

66 27 0

66 28 0.00103268685094826

66 29 0.000957076248417454

66 30 0.000684864461828139

66 31 0.00117402065889799

66 32 0.000930878545836289

66 33 9.27872136453622e-05

66 34 0

66 35 0.000418724877119803

66 36 0.00149587040929428

66 37 0.00141449456342424

66 38 0.00150408509880706

66 39 0

66 40 0.00535141034513675

66 41 0.00323759792517875

66 42 0.00154445309418638

66 43 0.00242461869488764

66 44 0.00192979641404989

66 45 0.00142148056455027

66 46 0.00307363910131053

66 47 0.00401315954076897

66 48 0.00167398964918830

66 49 0.00354082589091720

66 50 0.00339183687225964

66 51 0.00169705631864125

66 52 0.00104744385245414

66 53 0.000190872475173576

66 54 0.000858979968947127

66 55 0.000420789920398179

66 56 0.00199626752945670

66 57 0.00152869938534050

66 58 0.00226212600014308

66 59 0.00341018832874018

66 60 0.00289482977050570

66 61 0.00219897541179248

66 62 0.00117974358759260

66 63 0.00159131440122151

66 64 0.000850045642526665

66 65 0.000605422482315599

66 66 0

66 67 0.000404419291310965

66 68 0.000533320574316099

66 69 0.000950391611169277

66 70 0.00230370348503761

66 71 0.00266958809272266

66 72 0

66 73 0

66 74 0.00164965032101339

66 75 0.0140462809489248

66 76 0.0160368295245714

67 1 0

67 2 0

67 3 0

67 4 0

67 5 0

67 6 0

67 7 0

67 8 0.00360938603879135

67 9 0.00180192155988257

67 10 0

67 11 0

67 12 0

67 13 0

67 14 0

67 15 0

67 16 0

67 17 0

67 18 0

67 19 0

67 20 0

67 21 0

67 22 0

67 23 0

67 24 0

67 25 0

67 26 0

67 27 0

67 28 0

67 29 0

67 30 0

67 31 0

67 32 0

67 33 0

67 34 0

67 35 0

67 36 0

67 37 0

67 38 0

67 39 0

67 40 0.00196936426808603

67 41 0.000186332535002665

67 42 0

67 43 0

67 44 0

67 45 0

67 46 0.000339809188117046

67 47 0.00126658715629557

67 48 0

67 49 0.000833326026044023

67 50 0.000505363762601796

67 51 0

67 52 0

67 53 0

67 54 0

67 55 0

67 56 0

67 57 0

67 58 0

67 59 0.000488074949915873

67 60 0

67 61 0

67 62 0

67 63 0

67 64 0

67 65 0

67 66 0

67 67 0

67 68 0

67 69 0

67 70 0

67 71 0

67 72 0

67 73 0

67 74 0

67 75 0.0107062973944814

67 76 0.0127528721886461

68 1 0

68 2 0

68 3 0

68 4 0

68 5 0

68 6 0

68 7 0

68 8 0.00309879167236760

68 9 0.00122974278599008

68 10 0

68 11 0

68 12 0

68 13 0

68 14 0

68 15 0

68 16 0

68 17 0

68 18 0

68 19 0

68 20 0

68 21 0

68 22 0

68 23 0

68 24 0

68 25 0

68 26 0

68 27 0

68 28 0

68 29 0

68 30 0

68 31 0

68 32 0

68 33 0

68 34 0

68 35 0

68 36 0

68 37 0

68 38 0

68 39 0

68 40 0.00138037778246924

68 41 0

68 42 0

68 43 0

68 44 0

68 45 0

68 46 3.15185942905138e-05

68 47 0.000862085782506794

68 48 0

68 49 0.000643682456678341

68 50 0.000232982275329086

68 51 0

68 52 0

68 53 0

68 54 0

68 55 0

68 56 0

68 57 0

68 58 0

68 59 0.000346477384671418

68 60 0

68 61 0

68 62 0

68 63 0

68 64 0

68 65 0

68 66 0

68 67 0

68 68 0

68 69 0

68 70 0

68 71 0

68 72 0

68 73 0

68 74 0

68 75 0.0108425930308134

68 76 0.0133788183423993

69 1 0

69 2 0

69 3 0

69 4 0

69 5 0

69 6 0

69 7 0

69 8 0.00323487663268696

69 9 0.00136723417171747

69 10 0

69 11 0

69 12 0

69 13 0

69 14 0

69 15 0

69 16 0

69 17 0

69 18 0

69 19 0

69 20 0

69 21 0

69 22 0

69 23 0

69 24 0

69 25 0

69 26 0

69 27 0

69 28 0

69 29 0

69 30 0

69 31 0

69 32 0

69 33 0

69 34 0

69 35 0

69 36 0

69 37 0

69 38 0

69 39 0

69 40 0.00166683891662622

69 41 0

69 42 0

69 43 0

69 44 0

69 45 0

69 46 1.07563374619479e-05

69 47 0.000827528338859906

69 48 0

69 49 0.00104328491400740

69 50 0.000573282544114262

69 51 0

69 52 0

69 53 0

69 54 0

69 55 0

69 56 0

69 57 0

69 58 0

69 59 0.000380914582722380

69 60 0

69 61 0

69 62 0

69 63 0

69 64 0

69 65 0

69 66 0

69 67 0

69 68 0

69 69 0

69 70 0

69 71 0

69 72 0

69 73 0

69 74 0

69 75 0.0105013887807932

69 76 0.0130495546185034

70 1 0.00117557703966487

70 2 0.000939987990850821

70 3 0.00136117121735246

70 4 0.00135734324610892

70 5 0.000949625058257575

70 6 0

70 7 0

70 8 0.00600320021454426

70 9 0.00405966482541154

70 10 0

70 11 0.000978519218980067

70 12 0.00165491699574982

70 13 0.00188577209704199

70 14 0

70 15 0.000519894708780333

70 16 0.000740371084970382

70 17 0

70 18 8.53815375591038e-05

70 19 0.000448556934248612

70 20 0

70 21 0

70 22 0

70 23 0

70 24 0

70 25 0

70 26 5.50882118148799e-05

70 27 0

70 28 0.000433478981443525

70 29 0.000267746943166669

70 30 3.57939415918107e-05

70 31 0.000383063932204331

70 32 0.000246472120002506

70 33 0

70 34 0

70 35 0

70 36 0.000765947440972525

70 37 0.000810212550851452

70 38 0.000856720167235647

70 39 0

70 40 0.00443837544789716

70 41 0.00296681878120608

70 42 0.000489306172910875

70 43 0.00127401098389091

70 44 0.00143980518235431

70 45 0.00147468262310879

70 46 0.00246534675129928

70 47 0.00335034254428501

70 48 0.00105718186573223

70 49 0.00287877218050514

70 50 0.00334466250852350

70 51 0.00203823618984877

70 52 0.00148052722990566

70 53 0

70 54 0.000763176803444399

70 55 0.000365090370165300

70 56 0.00197950226197485

70 57 0.00166680447208523

70 58 0.00261193142007365

70 59 0.00353352480367442

70 60 0.00231418557383012

70 61 0.00146905381515794

70 62 0.000485819048003155

70 63 0.00116730395660691

70 64 0.000473675053778821

70 65 0.000515743782643741

70 66 6.86117025331390e-05

70 67 0

70 68 0.000253782605895037

70 69 0.000723993717450466

70 70 0.000389548593423161

70 71 0.00131740030581751

70 72 0

70 73 0.00160087228608030

70 74 0.00167746134605484

70 75 0.0129886881249512

70 76 0.0161409387345881

71 1 0.00312790146831266

71 2 0.00283918958666585

71 3 0.00317874996278400

71 4 0.00324041160118249

71 5 0.00291691790100634

71 6 0.00113905917836321

71 7 0.00143478850413614

71 8 0.00789471883476767

71 9 0.00603151764127152

71 10 0.00170831591147791

71 11 0.00281388186237330

71 12 0.00355520363863870

71 13 0.00377617601867220

71 14 0.000801036910790587

71 15 0.00243740656348623

71 16 0.00289385023063216

71 17 0.00147193841436177

71 18 0.00206237476599425

71 19 0.00239104215504199

71 20 0.00113057206728340

71 21 0.00101258228606993

71 22 0.000738950058535193

71 23 0.00117737306477972

71 24 0.000954926700363235

71 25 0.00174453778113892

71 26 0.00194114576466387

71 27 0.00112133555919081

71 28 0.00232798399043022

71 29 0.00218503185224161

71 30 0.00193193907829925

71 31 0.00237280892884340

71 32 0.00216697816269418

71 33 0.00167937846028610

71 34 0.000932153200969177

71 35 0.00161838954661098

71 36 0.00275017283097806

71 37 0.00272703020167087

71 38 0.00280088020485436

71 39 0.00116860976660949

71 40 0.00651982480855773

71 41 0.00474914257394288

71 42 0.00211787113422290

71 43 0.00315589423773754

71 44 0.00311725313664057

71 45 0.00271996229855631

71 46 0.00439997632482270

71 47 0.00532934784510897

71 48 0.00303613597517782

71 49 0.00468752412448426

71 50 0.00477231643659415

71 51 0.00328517167216247

71 52 0.00247784907453297

71 53 0.00137978473186906

71 54 0.00215332746583119

71 55 0.00180218691166556

71 56 0.00332167069931122

71 57 0.00288412620306244

71 58 0.00337385591313966

71 59 0.00459701078841124

71 60 0.00414330515364880

71 61 0.00348419622127483

71 62 0.00244043311570286

71 63 0.00316402160437057

71 64 0.00272586490444005

71 65 0.00257030354949894

71 66 0.00207426832807511

71 67 0.00163327228274646

71 68 0.00186869108876031

71 69 0.00220200743439192

71 70 0.00280649925963006

71 71 0.000738797481443898

71 72 0

71 73 0.000598662399773020

71 74 0.00338025177978663

71 75 0.0148748269783692

71 76 0.0171395168250892

72 1 0

72 2 0

72 3 0

72 4 0

72 5 0

72 6 0

72 7 0

72 8 0.000672794663390208

72 9 0

72 10 0

72 11 0

72 12 0

72 13 0

72 14 0

72 15 0

72 16 0

72 17 0

72 18 0

72 19 0

72 20 0.00502236522274369

72 21 0.00546920246658589

72 22 0.0199933126112661

72 23 0.0177274496674922

72 24 0.0168248796047905

72 25 0.00445114685642589

72 26 0.00912240649102092

72 27 0.0180166365220984

72 28 0.00834755685139899

72 29 0

72 30 0

72 31 0.000706096394338163

72 32 0

72 33 0

72 34 0

72 35 0

72 36 0

72 37 0.00249982239231605

72 38 0.00322046476692317

72 39 0.00893580505067027

72 40 0.00251759652040429

72 41 0

72 42 0

72 43 0

72 44 0

72 45 0.00419395545733203

72 46 0.00210533566793436

72 47 0

72 48 0

72 49 0.000352444832225407

72 50 0.00141148563392646

72 51 0

72 52 0.0107333042169591

72 53 0.0196887001638562

72 54 0.0177270936416367

72 55 0.0165151675065109

72 56 0.00628901278330707

72 57 0.00782805544637721

72 58 0.00450410282325287

72 59 0.00144277789261826

72 60 0

72 61 0

72 62 0

72 63 0

72 64 0

72 65 0

72 66 0

72 67 0

72 68 0

72 69 0

72 70 0

72 71 0

72 72 0

72 73 0.00999260498078258

72 74 0.00852611586690943

72 75 0.0173901929863860

72 76 0.0342192751974040

73 1 0

73 2 0.000329554161353163

73 3 0

73 4 0

73 5 0

73 6 0

73 7 0

73 8 0.00244849441227968

73 9 0.000367633373679510

73 10 0

73 11 0

73 12 0

73 13 0

73 14 0

73 15 0

73 16 0

73 17 0

73 18 0

73 19 0

73 20 0

73 21 2.51598158125255e-05

73 22 0.0153519071143831

73 23 0.00951730358206160

73 24 0.0118811494775567

73 25 0.00336043352671689

73 26 0.00725192328532964

73 27 0.0132548910840179

73 28 0.00690458394435456

73 29 0

73 30 0

73 31 0.00271696698083368

73 32 0

73 33 0

73 34 0

73 35 0

73 36 0

73 37 0.00279809892517335

73 38 0.00364423953494109

73 39 0.00941612350049548

73 40 0.00382636999863140

73 41 0

73 42 0

73 43 0

73 44 0.00393435285596833

73 45 0.00495682127252117

73 46 0.00223444073900403

73 47 0.000654538277620409

73 48 0

73 49 0.00480177330530374

73 50 0.00383569361173464

73 51 0

73 52 0.00799581216400136

73 53 0.0162421761564755

73 54 0.0140701262463852

73 55 0.0116537449465786

73 56 0.00436499977507576

73 57 0.00478160671023531

73 58 0.00174999966182132

73 59 0.00162275374764331

73 60 0

73 61 0

73 62 0

73 63 0

73 64 0

73 65 0

73 66 0

73 67 0

73 68 0

73 69 0

73 70 0

73 71 0

73 72 0.0132415799490233

73 73 0

73 74 0.00936300029469495

73 75 0.0182577446486418

73 76 0.0294964771921502

74 1 0

74 2 0

74 3 0

74 4 0

74 5 0

74 6 0

74 7 0

74 8 0.00158941138952418

74 9 0

74 10 0

74 11 0

74 12 0

74 13 0

74 14 0

74 15 0

74 16 0

74 17 0

74 18 0

74 19 0

74 20 0

74 21 0

74 22 0

74 23 0

74 24 0

74 25 0

74 26 0

74 27 0

74 28 0

74 29 0

74 30 0

74 31 0

74 32 0

74 33 0

74 34 0

74 35 0

74 36 0

74 37 0

74 38 0

74 39 0

74 40 0.00232381544054749

74 41 0

74 42 0.00227971820780426

74 43 0.00100658975677081

74 44 0

74 45 0

74 46 0

74 47 0

74 48 0

74 49 0.000708594562551679

74 50 0.00403622281467886

74 51 0.00145324678304348

74 52 0

74 53 0

74 54 0

74 55 0

74 56 0

74 57 0

74 58 0

74 59 0.000541641393512871

74 60 0.000126922005302954

74 61 0

74 62 0

74 63 0

74 64 0

74 65 0

74 66 0

74 67 0

74 68 0

74 69 0

74 70 0

74 71 0

74 72 0

74 73 0.000447098495661935

74 74 0

74 75 0.0132849279821319

74 76 0.0192946532190779

75 1 0

75 2 0

75 3 0

75 4 0

75 5 0

75 6 0

75 7 0

75 8 0.000161626867922982

75 9 0

75 10 0

75 11 0

75 12 0

75 13 0

75 14 0

75 15 0

75 16 0

75 17 0

75 18 0

75 19 0

75 20 0

75 21 0

75 22 0

75 23 0

75 24 0

75 25 0

75 26 0

75 27 0

75 28 0

75 29 0

75 30 0

75 31 0

75 32 0

75 33 0

75 34 0

75 35 0

75 36 0

75 37 0

75 38 0

75 39 0

75 40 0.000203354283407453

75 41 0

75 42 0

75 43 0

75 44 0

75 45 0

75 46 0

75 47 0

75 48 0

75 49 0

75 50 0

75 51 0

75 52 0

75 53 0

75 54 0

75 55 0

75 56 0

75 57 0

75 58 0

75 59 0

75 60 0

75 61 0

75 62 0

75 63 0

75 64 0

75 65 0

75 66 0

75 67 0

75 68 0

75 69 0

75 70 0

75 71 0

75 72 0

75 73 0

75 74 0

75 75 0.0111258911886890

75 76 0.0109900643775780

76 1 0

76 2 0

76 3 0

76 4 0

76 5 0

76 6 0

76 7 0

76 8 0

76 9 0

76 10 0

76 11 0

76 12 0

76 13 0

76 14 0

76 15 0

76 16 0

76 17 0

76 18 0

76 19 0

76 20 0

76 21 0

76 22 0.00208397881604272

76 23 0

76 24 0.000548032610852234

76 25 0

76 26 8.41550225560983e-05

76 27 0.00374121519073367

76 28 0

76 29 0

76 30 0

76 31 0

76 32 0

76 33 0

76 34 0

76 35 0

76 36 0

76 37 0

76 38 0

76 39 0

76 40 0.00101670214774896

76 41 0

76 42 0

76 43 0

76 44 0

76 45 0

76 46 0

76 47 0

76 48 0

76 49 0

76 50 5.92253055851089e-05

76 51 0

76 52 0.00147811008041265

76 53 0.00358234206172559

76 54 0.00402307509461819

76 55 0.000172882771009464

76 56 0

76 57 0

76 58 0

76 59 0

76 60 0

76 61 0

76 62 0

76 63 0

76 64 0

76 65 0

76 66 0

76 67 0

76 68 0

76 69 0

76 70 0

76 71 0

76 72 0.0126858979953359

76 73 0.00534511617119682

76 74 0

76 75 0.0110726994521388

76 76 0.0127778047531453

Data for Figs 4 and 5

Entropy transfer from individual residues to others. The values for drawing the figures in the six panels can be extracted from the following data, where the first two columns are residue indices, the third column is entropy transfer.

1 1 0

1 2 0

1 3 0

1 4 0

1 5 0

1 6 0

1 7 0

1 8 0.00499195359501925

1 9 0.00327547472128253

1 10 0

1 11 0

1 12 0.000380027474992994

1 13 0.000171211310904251

1 14 0

1 15 0

1 16 0.000142796658508138

1 17 0

1 18 0

1 19 0

1 20 0

1 21 0

1 22 0

1 23 0

1 24 0

1 25 0

1 26 0

1 27 0

1 28 0

1 29 0

1 30 0

1 31 0

1 32 0

1 33 0

1 34 0

1 35 0

1 36 0

1 37 7.26430846265669e-05

1 38 0.000193911291881510

1 39 0

1 40 0.00101138271575241

1 41 0.000467014129495680

1 42 0

1 43 0

1 44 0.000134135727098084

1 45 0

1 46 0.00147701165521896

1 47 0.00209535285171381

1 48 0.000117952516724640

1 49 0.00118840522045660

1 50 0.000241779103676065

1 51 0

1 52 0

1 53 0

1 54 0

1 55 0

1 56 9.95155175600448e-05

1 57 0

1 58 0.000243932270069247

1 59 0.000898182610748921

1 60 0.000787918282209699

1 61 0.000426036113230999

1 62 0

1 63 0

1 64 0

1 65 0

1 66 0

1 67 0

1 68 0

1 69 0

1 70 0.000128523031241778

1 71 0

1 72 0

1 73 0

1 74 0.000441289284994650

1 75 0.0127082131612990

1 76 0.0153160714225080

2 1 0.00212529229554581

2 2 0

2 3 0.00136575642512493

2 4 0.00128372085954198

2 5 0.00190334711206230

2 6 0.000629260472910231

2 7 0.00116512858432150

2 8 0.00736410052901249

2 9 0.00563835075069374

2 10 0.00150418333211033

2 11 0.00202195439970332

2 12 0.00270515288920059

2 13 0.00238883112921828

2 14 0.000723844290482334

2 15 0.00172606070051939

2 16 0.00239010767184766

2 17 0.00143373974024896

2 18 0.00175505286772315

2 19 0.00198927822603834

2 20 0.00127874454367205

2 21 0.00120962299738070

2 22 0.000941498389051332

2 23 0.00125804736800061

2 24 0.00130804068162971

2 25 0.00163345804600801

2 26 0.00208535015865274

2 27 0.00160190896889167

2 28 0.00238922325665458

2 29 0.00183521323357205

2 30 0.00165025815026454

2 31 0.00208786711525288

2 32 0.00184285600106349

2 33 0.00121088385655477

2 34 0.000695167350572623

2 35 0.00130484415295906

2 36 0.00225650499559310

2 37 0.00244462122658540

2 38 0.00257563857517817

2 39 0.00187633999030623

2 40 0.00374839461695908

2 41 0.00280949677910158

2 42 0.00139360925889587

2 43 0.00198408895636204

2 44 0.00244532131792363

2 45 0.00246342755667495

2 46 0.00398253170616603

2 47 0.00445568302554367

2 48 0.00243492948749580

2 49 0.00348577874098344

2 50 0.00256428088541949

2 51 0.00123671989644825

2 52 0.00138799775298848

2 53 0.000556596645222118

2 54 0.000998580409344418

2 55 0.00138673966831249

2 56 0.00278917039842508

2 57 0.00239467797858817

2 58 0.00261413647441366

2 59 0.00331649673342394

2 60 0.00310386170329136

2 61 0.00285219802344305

2 62 0.00197272011725669

2 63 0.00199560151942257

2 64 0.000916807541992415

2 65 0.00185612064785001

2 66 0.00128565640240519

2 67 0.00124080896424861

2 68 0.00152000462218271

2 69 0.00180301262215110

2 70 0.00246780841053618

2 71 0.00229113260396774

2 72 0.000218135128412067

2 73 0.000657576872329035

2 74 0.00362071338405090

2 75 0.0150769727701436

2 76 0.0179250294530626

3 1 0.00431409918708092

3 2 0.00353714832091800

3 3 0

3 4 0.00173123187279878

3 5 0.00395796320468766

3 6 0.00265172249117374

3 7 0.00339499656901487

3 8 0.00968679431120756

3 9 0.00788602939919569

3 10 0.00365721694969212

3 11 0.00401797502152501

3 12 0.00486417765624281

3 13 0.00368369180991868

3 14 0.00267785486243155

3 15 0.00379697808683332

3 16 0.00436735608409744

3 17 0.00376721424619031

3 18 0.00416049251589135

3 19 0.00418456573404169

3 20 0.00316571546782296

3 21 0.00300915946230951

3 22 0.00231346468414628

3 23 0.00257668309243930

3 24 0.00260010598071236

3 25 0.00358502045155362

3 26 0.00398432119776526

3 27 0.00312923990553993

3 28 0.00413809097731166

3 29 0.00405720921418040

3 30 0.00382312889193726

3 31 0.00428118319452164

3 32 0.00404835976125784

3 33 0.00330753760035929

3 34 0.00289306611756357

3 35 0.00356210641175903

3 36 0.00449792250821629

3 37 0.00447645001832508

3 38 0.00454213221381228

3 39 0.00350161203909993

3 40 0.00679453998402813

3 41 0.00521935550409847

3 42 0.00377992782947323

3 43 0.00435866852548561

3 44 0.00452092175068763

3 45 0.00447317024850957

3 46 0.00590152181501979

3 47 0.00664307169374834

3 48 0.00460182301524892

3 49 0.00563877816995573

3 50 0.00481777446468035

3 51 0.00341262047692115

3 52 0.00336406056279226

3 53 0.00282835546628246

3 54 0.00325376784608689

3 55 0.00313705429453992

3 56 0.00456330780949610

3 57 0.00420836674485714

3 58 0.00486687786655449

3 59 0.00544295928441607

3 60 0.00537404223065896

3 61 0.00510516688409002

3 62 0.00422983757153805

3 63 0.00453220833465939

3 64 0.00360923366573473

3 65 0.00412121066588156

3 66 0.00355022835251795

3 67 0.00348843607365823

3 68 0.00371560049325081

3 69 0.00393282596968603

3 70 0.00467078057768544

3 71 0.00457062654714990

3 72 0.00232082633401309

3 73 0.00277405618981830

3 74 0.00501468805970373

3 75 0.0178619076811049

3 76 0.0205280150341705

4 1 0.00526909853039792

4 2 0.00481340060288926

4 3 0.00351680929734710

4 4 0

4 5 0.00483932717465407

4 6 0.00338792479958527

4 7 0.00443579433480024

4 8 0.0107216501337432

4 9 0.00888421556876728

4 10 0.00469927120420999

4 11 0.00495952787423326

4 12 0.00597018044222319

4 13 0.00454552133021058

4 14 0.00387496511493080

4 15 0.00479566376636909

4 16 0.00534034510183190

4 17 0.00491967480184696

4 18 0.00532126350556661

4 19 0.00526849870424073

4 20 0.00409062499280066

4 21 0.00397260824696488

4 22 0.00337198483012369

4 23 0.00379742158066565

4 24 0.00374135969120526

4 25 0.00465286171065671

4 26 0.00491641908838747

4 27 0.00390698808646017

4 28 0.00514989997965787

4 29 0.00506624932554145

4 30 0.00483116373278325

4 31 0.00535009932398689

4 32 0.00510566555961445

4 33 0.00444071956187475

4 34 0.00399708552281008

4 35 0.00463388463853598

4 36 0.00555841231638976

4 37 0.00548017103694976

4 38 0.00551789533119484

4 39 0.00459181050883017

4 40 0.00882212555460160

4 41 0.00642783991066220

4 42 0.00489108076565514

4 43 0.00549012479769329

4 44 0.00554709613098747

4 45 0.00544962680803818

4 46 0.00686358012444099

4 47 0.00769770025260486

4 48 0.00563924052839304

4 49 0.00671249247692429

4 50 0.00600889308027430

4 51 0.00480587910857633

4 52 0.00488788341371604

4 53 0.00429099978402459

4 54 0.00467309347810230

4 55 0.00416911611703585

4 56 0.00563256107696353

4 57 0.00526015366635935

4 58 0.00588165083403047

4 59 0.00647915567660429

4 60 0.00646994480263119

4 61 0.00621927422771684

4 62 0.00529815410839818

4 63 0.00570337472759763

4 64 0.00548021979476110

4 65 0.00524552945164380

4 66 0.00458752801988416

4 67 0.00460533992214185

4 68 0.00484495933026397

4 69 0.00499589220316221

4 70 0.00559406770731841

4 71 0.00568107124872075

4 72 0.00354322059522372

4 73 0.00378443204413748

4 74 0.00625788316292808

4 75 0.0199963081556125

4 76 0.0226890131934763

5 1 0.00154378744454697

5 2 0.00112829511413992

5 3 0.000946109079379309

5 4 0.000354021523416126

5 5 0

5 6 0

5 7 0.000800035133349364

5 8 0.00695806307447511

5 9 0.00514722506006615

5 10 0.000982105740595096

5 11 0.00137999987085358

5 12 0.00211223835127250

5 13 0.00144089293504313

5 14 0

5 15 0.00109342861072426

5 16 0.00145052206668761

5 17 0.000769516544604443

5 18 0.00129754942545113

5 19 0.00151827618649342

5 20 0.000297375436514757

5 21 0.000185796805416749

5 22 0

5 23 0

5 24 0

5 25 0.000926053716465369

5 26 0.00112246216676026

5 27 0.000205651784198846

5 28 0.00138438698107768

5 29 0.00133474245068754

5 30 0.00108655878400388

5 31 0.00152123674224303

5 32 0.00133765046199541

5 33 0.000573473316381801

5 34 8.28419628431387e-05

5 35 0.000866843034761922

5 36 0.00183144563389298

5 37 0.00176334035590420

5 38 0.00180836636453013

5 39 0.000218650459769210

5 40 0.00330568637632767

5 41 0.00239607637722616

5 42 0.000782752774591944

5 43 0.00146338877489927

5 44 0.00178190193355265

5 45 0.00169322486676626

5 46 0.00299638173527994

5 47 0.00386773252774308

5 48 0.00190429595626318

5 49 0.00288459819224962

5 50 0.00199308370788442

5 51 0.000793273453645882

5 52 0.000420413786161866

5 53 0

5 54 0.000272148557254326

5 55 0.000352671293645024

5 56 0.00190411623798770

5 57 0.00152681396568388

5 58 0.00204395100213450

5 59 0.00276762177703760

5 60 0.00269177243083585

5 61 0.00241393961865033

5 62 0.00154639400104506

5 63 0.00163717168473576

5 64 0.000867596406067817

5 65 0.00145416503293283

5 66 0.000879287629600989

5 67 0.000812356613920651

5 68 0.00106949371729903

5 69 0.00122053278199008

5 70 0.00185471703287032

5 71 0.00181453330145531

5 72 0

5 73 0

5 74 0.00205943438397871

5 75 0.0147661843672247

5 76 0.0169636925515451

6 1 0.00371493863822592

6 2 0.00341443432723565

6 3 0.00336691618476892

6 4 0.00270698437097316

6 5 0.00306945877357123

6 6 0

6 7 0.00350350936787225

6 8 0.00926666772720342

6 9 0.00718393967953646

6 10 0.00306699017809942

6 11 0.00334467108386638

6 12 0.00421874902056496

6 13 0.00373062048338757

6 14 0.00204430036466519

6 15 0.00322925438405541

6 16 0.00355945414137215

6 17 0.00274902121562048

6 18 0.00327955633016408

6 19 0.00357513265011566

6 20 0.00246390215858139

6 21 0.00236129425427356

6 22 0.00158695323333280

6 23 0.00187070080491214

6 24 0.00186428457202814

6 25 0.00292930494826615

6 26 0.00313323509007923

6 27 0.00219834252574724

6 28 0.00338867890750838

6 29 0.00339635198187194

6 30 0.00313135914568552

6 31 0.00352115263519603

6 32 0.00340614535790318

6 33 0.00261310213521504

6 34 0.00212411348799069

6 35 0.00289492568442062

6 36 0.00385442731518243

6 37 0.00388211100602565

6 38 0.00390120494657581

6 39 0.00222271548921194

6 40 0.00514471381278392

6 41 0.00442831366609242

6 42 0.00288907313592712

6 43 0.00355000707674191

6 44 0.00398350133412306

6 45 0.00393373247368312

6 46 0.00513249093862689

6 47 0.00598559201758842

6 48 0.00397952225417964

6 49 0.00497277706824628

6 50 0.00406929820554380

6 51 0.00294490411655834

6 52 0.00261248327449959

6 53 0.00202664070631897

6 54 0.00253507198399161

6 55 0.00246818561300888

6 56 0.00395483005046904

6 57 0.00361222934792216

6 58 0.00430991328673969

6 59 0.00487316630878854

6 60 0.00475366912393493

6 61 0.00445451362050053

6 62 0.00362911877422334

6 63 0.00361419725987688

6 64 0.00295717748355639

6 65 0.00350397454215512

6 66 0.00297538763219385

6 67 0.00301951864565531

6 68 0.00336624440036071

6 69 0.00336649952276535

6 70 0.00390872122662633

6 71 0.00385631663504304

6 72 0.00200345014087455

6 73 0.00209659348943014

6 74 0.00435092830516493

6 75 0.0165675098849076

6 76 0.0182299756923618

7 1 0

7 2 0

7 3 0

7 4 0

7 5 0

7 6 0

7 7 0

7 8 0.00405345131776025

7 9 0.00219151555696262

7 10 0

7 11 0

7 12 0

7 13 0

7 14 0

7 15 0

7 16 0

7 17 0

7 18 0

7 19 0

7 20 0

7 21 0

7 22 0

7 23 0

7 24 0

7 25 0

7 26 0

7 27 0

7 28 0

7 29 0

7 30 0

7 31 0

7 32 0

7 33 0

7 34 0

7 35 0

7 36 0

7 37 0

7 38 0

7 39 0

7 40 0

7 41 0

7 42 0

7 43 0

7 44 0

7 45 0

7 46 2.34190096006781e-05

7 47 0.000946598268330545

7 48 0

7 49 0.000109174475455665

7 50 0

7 51 0

7 52 0

7 53 0

7 54 0

7 55 0

7 56 0

7 57 0

7 58 0

7 59 0

7 60 0

7 61 0

7 62 0

7 63 0

7 64 0

7 65 0

7 66 0

7 67 0

7 68 0

7 69 0

7 70 0

7 71 0

7 72 0

7 73 0

7 74 0

7 75 0.0116704449016816

7 76 0.0132800382808624

8 1 0

8 2 0

8 3 0

8 4 0

8 5 0

8 6 0

8 7 0

8 8 0.00470427103882015

8 9 0.00178592504578978

8 10 0

8 11 0

8 12 0

8 13 0

8 14 0

8 15 0

8 16 0

8 17 0

8 18 0

8 19 0

8 20 0

8 21 0

8 22 0

8 23 0

8 24 0

8 25 0

8 26 0

8 27 0

8 28 0

8 29 0

8 30 0

8 31 0

8 32 0

8 33 0

8 34 0

8 35 0

8 36 0

8 37 0

8 38 0

8 39 0

8 40 0

8 41 0

8 42 0

8 43 0

8 44 0

8 45 0

8 46 0

8 47 6.21458493101912e-05

8 48 0

8 49 0

8 50 0

8 51 0

8 52 0

8 53 0

8 54 0

8 55 0

8 56 0

8 57 0

8 58 0

8 59 0

8 60 0

8 61 0

8 62 0

8 63 0

8 64 0

8 65 0

8 66 0

8 67 0

8 68 0

8 69 0

8 70 0

8 71 0

8 72 0

8 73 0

8 74 0

8 75 0.0111528446617495

8 76 0.0131820345850358

9 1 0

9 2 0

9 3 0

9 4 0

9 5 0

9 6 0

9 7 0

9 8 0.00478884853192219

9 9 0.00298912336015789

9 10 0

9 11 0

9 12 0

9 13 0

9 14 0

9 15 0

9 16 0

9 17 0

9 18 0

9 19 0

9 20 0

9 21 0

9 22 0

9 23 0

9 24 0

9 25 0

9 26 0

9 27 0

9 28 0

9 29 0

9 30 0

9 31 0

9 32 0

9 33 0

9 34 0

9 35 0

9 36 0

9 37 0

9 38 0

9 39 0

9 40 0.000355489231214090

9 41 0

9 42 0

9 43 0

9 44 0

9 45 0

9 46 0.000430121192885324

9 47 0.00131342268217649

9 48 0

9 49 0.000370344530640998

9 50 0

9 51 0

9 52 0

9 53 0

9 54 0

9 55 0

9 56 0

9 57 0

9 58 0

9 59 0.000208161233017590

9 60 9.91186911006858e-05

9 61 0

9 62 0

9 63 0

9 64 0

9 65 0

9 66 0

9 67 0

9 68 0

9 69 0

9 70 0

9 71 0

9 72 0

9 73 0

9 74 0

9 75 0.0116844997888046

9 76 0.0137122419658742

10 1 0

10 2 0

10 3 0

10 4 0

10 5 0

10 6 0

10 7 0

10 8 0.00380151984745825

10 9 0.00241537657469315

10 10 0

10 11 0

10 12 0

10 13 0

10 14 0

10 15 0

10 16 0

10 17 0

10 18 0

10 19 0

10 20 0

10 21 0

10 22 0

10 23 0

10 24 0

10 25 0

10 26 0

10 27 0

10 28 0

10 29 0

10 30 0

10 31 0

10 32 0

10 33 0

10 34 0

10 35 0

10 36 0

10 37 0

10 38 0

10 39 0

10 40 0

10 41 0

10 42 0

10 43 0

10 44 0

10 45 0

10 46 0

10 47 0.000449997582747885

10 48 0

10 49 0

10 50 0

10 51 0

10 52 0

10 53 0

10 54 0

10 55 0

10 56 0

10 57 0

10 58 0

10 59 0

10 60 0

10 61 0

10 62 0

10 63 0

10 64 0

10 65 0

10 66 0

10 67 0

10 68 0

10 69 0

10 70 0

10 71 0

10 72 0

10 73 0

10 74 0

10 75 0.0112421649227668

10 76 0.0129375583150424

11 1 0.00128552575080643

11 2 0.000890406312384573

11 3 0.000884433376877936

11 4 0.000502886369253108

11 5 0.00107956337824233

11 6 0

11 7 0.000565728041316160

11 8 0.00665217962935893

11 9 0.00487115421314155

11 10 0.000785730801620421

11 11 0

11 12 0.00161267803736531

11 13 0.00116725098123116

11 14 0

11 15 0.000900665032368520

11 16 0.00118961638957960

11 17 0.000506212016247121

11 18 0.00102696606705865

11 19 0.00128514031751925

11 20 9.16102857461132e-05

11 21 0

11 22 0

11 23 0

11 24 0

11 25 0.000635687312817601

11 26 0.000833819695069793

11 27 0

11 28 0.00112177764148408

11 29 0.00112563416609685

11 30 0.000890577502078549

11 31 0.00130032913180789

11 32 0.00113064474958308

11 33 0.000309119771633615

11 34 0

11 35 0.000605502217632337

11 36 0.00156812369363005

11 37 0.00152205910589653

11 38 0.00158711446334761

11 39 0

11 40 0.00284584286512002

11 41 0.00210349739696769

11 42 0.000587385700391852

11 43 0.00128491779539675

11 44 0.00157765317583114

11 45 0.00147756460430998

11 46 0.00277412728496856

11 47 0.00364269024502140

11 48 0.00169030401498926

11 49 0.00266265904980401

11 50 0.00184242708034843

11 51 0.000623656829442876

11 52 0.000309428175073290

11 53 0

11 54 0.000146173057345322

11 55 0.000143808195998796

11 56 0.00168350886242274

11 57 0.00131065588226231

11 58 0.00182300426019877

11 59 0.00256251796808282

11 60 0.00246009667389713

11 61 0.00215407253029720

11 62 0.00132853911758352

11 63 0.00130580310959527

11 64 0.000419341392755124

11 65 0.00118142122086667

11 66 0.000603836068084895

11 67 0.000584277033801983

11 68 0.000755061152075520

11 69 0.000960711215156784

11 70 0.00164571256278401

11 71 0.00158038008005224

11 72 0

11 73 0

11 74 0.00182942979846268

11 75 0.0142224716902380

11 76 0.0159075316666804

12 1 6.93993927775027e-05

12 2 0

12 3 0

12 4 0

12 5 0

12 6 0

12 7 0

12 8 0.00537469060471840

12 9 0.00366884059769007

12 10 0

12 11 0

12 12 5.72014410995525e-05

12 13 0.000215801426835138

12 14 0

12 15 0

12 16 0

12 17 0

12 18 0

12 19 1.58363421411956e-05

12 20 0

12 21 0

12 22 0

12 23 0

12 24 0

12 25 0

12 26 0

12 27 0

12 28 0

12 29 0

12 30 0

12 31 5.45954510182134e-05

12 32 0

12 33 0

12 34 0

12 35 0

12 36 0.000324563263181421

12 37 0.000288164241372013

12 38 0.000346536047449914

12 39 0

12 40 0.00167082154299636

12 41 0.000862315097586219

12 42 0

12 43 2.29979392356583e-05

12 44 0.000351740834718173

12 45 0.000243096253180974

12 46 0.00155571387457326

12 47 0.00244732561568106

12 48 0.000435804314407706

12 49 0.00145333380376300

12 50 0.000666877297638702

12 51 0

12 52 0

12 53 0

12 54 0

12 55 0

12 56 0.000429643806464874

12 57 6.58567294750667e-05

12 58 0.000677978204333463

12 59 0.00131147875522208

12 60 0.00120504406071775

12 61 0.000908281739848138

12 62 5.65710291883237e-05

12 63 6.30165961117424e-05

12 64 0

12 65 0

12 66 0

12 67 0

12 68 0

12 69 0

12 70 0.000386747752414096

12 71 0.000313514676547499

12 72 0

12 73 0

12 74 0.000584465536134005

12 75 0.0128855979251346

12 76 0.0147737063170950

13 1 0.00468003708107001

13 2 0.00425058083967522

13 3 0.00326492305533888

13 4 0.00257200245320477

13 5 0.00406159299384745

13 6 0.00282703192146649

13 7 0.00369851264028176

13 8 0.00991815951137753

13 9 0.00817877217458118

13 10 0.00393055962210587

13 11 0.00402409399095749

13 12 0.00505395351120652

13 13 0

13 14 0.00309368215542671

13 15 0.00415470132662554

13 16 0.00463446331708772

13 17 0.00385027145458561

13 18 0.00438778961907105

13 19 0.00445800644128069

13 20 0.00355216864131369

13 21 0.00337800995872029

13 22 0.00285544318894970

13 23 0.00309155487356805

13 24 0.00314171100886407

13 25 0.00386886687304078

13 26 0.00445762434308461

13 27 0.00373067870720956

13 28 0.00447875716442137

13 29 0.00437632836145430

13 30 0.00411665585539489

13 31 0.00468716424808957

13 32 0.00436153451008647

13 33 0.00356663314073380

13 34 0.00311661240056826

13 35 0.00379631144656045

13 36 0.00479395297878898

13 37 0.00478990603990309

13 38 0.00485255139009561

13 39 0.00387651718633741

13 40 0.00636923940737444

13 41 0.00540874237211586

13 42 0.00394985938107650

13 43 0.00461919946228773

13 44 0.00485236252698207

13 45 0.00479405505879971

13 46 0.00617125924896900

13 47 0.00691345686839429

13 48 0.00494149402797495

13 49 0.00592241033937668

13 50 0.00503216035963083

13 51 0.00371814278825289

13 52 0.00388816694713312

13 53 0.00321653622315354

13 54 0.00378083106442961

13 55 0.00357614732228340

13 56 0.00485783545676932

13 57 0.00452276755069769

13 58 0.00518329313164123

13 59 0.00567694723365675

13 60 0.00567111943488885

13 61 0.00538669629521382

13 62 0.00451799033147082

13 63 0.00495076024958641

13 64 0.00382299703414879

13 65 0.00442276315812767

13 66 0.00373396386995395

13 67 0.00376825336918529

13 68 0.00398446665436403

13 69 0.00418965245210434

13 70 0.00494986646463869

13 71 0.00475615879527913

13 72 0.00260473328851574

13 73 0.00283306937976602

13 74 0.00524337834759880

13 75 0.0175065896007092

13 76 0.0207506128043944

14 1 0

14 2 0

14 3 0

14 4 0

14 5 0

14 6 0

14 7 0

14 8 0.00461633869079359

14 9 0.00287160576884926

14 10 0

14 11 0

14 12 0

14 13 0.000244079249420937

14 14 0

14 15 0

14 16 0

14 17 0

14 18 0

14 19 0

14 20 0

14 21 0

14 22 0

14 23 0

14 24 0

14 25 0

14 26 0

14 27 0

14 28 0

14 29 0

14 30 0

14 31 0

14 32 0

14 33 0

14 34 0

14 35 0

14 36 0

14 37 0.000320939620020888

14 38 0.000466608628969523

14 39 0

14 40 0.00151397988158830

14 41 0.000110916305965936

14 42 0

14 43 0

14 44 0

14 45 0.000421699043405388

14 46 0.00146182852692067

14 47 0.00171070686960340

14 48 0

14 49 0.000895497962634217

14 50 0

14 51 0

14 52 0

14 53 0

14 54 0

14 55 0

14 56 0

14 57 0

14 58 0

14 59 0.000588877941740673

14 60 0.000301351954570150

14 61 0

14 62 0

14 63 0

14 64 0

14 65 0

14 66 0

14 67 0

14 68 0

14 69 0

14 70 0

14 71 0

14 72 0

14 73 0

14 74 0.000336552679486735

14 75 0.0120870866014247

14 76 0.0157921621218167

15 1 0.000135583003511019

15 2 0

15 3 0

15 4 0

15 5 0

15 6 0

15 7 0

15 8 0.00522780131930800

15 9 0.00352334082365069

15 10 0

15 11 0

15 12 0.000575031712824359

15 13 0.000251919350155450

15 14 0

15 15 0

15 16 0.000411386454263418

15 17 0

15 18 0

15 19 0

15 20 0

15 21 0

15 22 0

15 23 0

15 24 0

15 25 0

15 26 0

15 27 0

15 28 0

15 29 0

15 30 0

15 31 0

15 32 0

15 33 0

15 34 0

15 35 0

15 36 0.000124281371446600

15 37 6.51039486886940e-05

15 38 0.000147372294942438

15 39 0

15 40 0.00125036402412648

15 41 0.000656379552078423

15 42 0

15 43 0

15 44 0.000174832385908208

15 45 2.85993666601092e-05

15 46 0.00145306133551282

15 47 0.00219437753255580

15 48 0.000254325967666724

15 49 0.00122833563781199

15 50 0.000334647017136591

15 51 0

15 52 0

15 53 0

15 54 0

15 55 0

15 56 0.000214829334143407

15 57 0

15 58 0.000559963178395018

15 59 0.00105523704655264

15 60 0.00100080952222803

15 61 0.000671125649040483

15 62 0

15 63 0

15 64 0

15 65 0

15 66 0

15 67 0

15 68 0

15 69 0

15 70 0.000350760471881206

15 71 0.000143900368440475

15 72 0

15 73 0

15 74 0.000620781788234548

15 75 0.0127064011333873

15 76 0.0145990517345660

16 1 0

16 2 0

16 3 0

16 4 0

16 5 0

16 6 0

16 7 0

16 8 0.00401785386312936

16 9 0.00237588282549139

16 10 0

16 11 0

16 12 0

16 13 0

16 14 0

16 15 0

16 16 0

16 17 0

16 18 0

16 19 0

16 20 0

16 21 0

16 22 0

16 23 0

16 24 0

16 25 0

16 26 0

16 27 0

16 28 0

16 29 0

16 30 0

16 31 0

16 32 0

16 33 0

16 34 0

16 35 0

16 36 0

16 37 0

16 38 0

16 39 0

16 40 8.66392404512695e-05

16 41 0

16 42 0

16 43 0

16 44 0

16 45 0

16 46 0.000375101809235723

16 47 0.00101736247777506

16 48 0

16 49 0.000165612503676638

16 50 0

16 51 0

16 52 0

16 53 0

16 54 0

16 55 0

16 56 0

16 57 0

16 58 0

16 59 0

16 60 0

16 61 0

16 62 0

16 63 0

16 64 0

16 65 0

16 66 0

16 67 0

16 68 0

16 69 0

16 70 0

16 71 0

16 72 0

16 73 0

16 74 0.000134317465450340

16 75 0.0120301098401212

16 76 0.0145205745789843

17 1 0

17 2 0

17 3 0

17 4 0

17 5 0

17 6 0

17 7 0

17 8 0.00266103111083571

17 9 0.00103693882410216

17 10 0

17 11 0

17 12 0

17 13 0

17 14 0

17 15 0

17 16 0

17 17 0

17 18 0

17 19 0

17 20 0

17 21 0

17 22 0

17 23 0

17 24 0

17 25 0

17 26 0

17 27 0

17 28 0

17 29 0

17 30 0

17 31 0

17 32 0

17 33 0

17 34 0

17 35 0

17 36 0

17 37 0

17 38 0

17 39 0

17 40 0

17 41 0

17 42 0

17 43 0

17 44 0

17 45 0

17 46 0

17 47 0

17 48 0

17 49 0

17 50 0

17 51 0

17 52 0

17 53 0

17 54 0

17 55 0

17 56 0

17 57 0

17 58 0

17 59 0

17 60 0

17 61 0

17 62 0

17 63 0

17 64 0

17 65 0

17 66 0

17 67 0

17 68 0

17 69 0

17 70 0

17 71 0

17 72 0

17 73 0

17 74 0

17 75 0.0104101938233307

17 76 0.0131779559647669

18 1 0

18 2 0

18 3 0

18 4 0

18 5 0

18 6 0

18 7 0

18 8 0.00363772046981892

18 9 0.00194669933208091

18 10 0

18 11 0

18 12 0

18 13 0

18 14 0

18 15 0

18 16 0

18 17 0

18 18 0

18 19 0

18 20 0

18 21 0

18 22 0

18 23 0

18 24 0

18 25 0

18 26 0

18 27 0

18 28 0

18 29 0

18 30 0

18 31 0

18 32 0

18 33 0

18 34 0

18 35 0

18 36 0

18 37 0

18 38 0

18 39 0

18 40 0

18 41 0

18 42 0

18 43 0

18 44 0

18 45 0

18 46 0

18 47 0.000782000831707486

18 48 0

18 49 0

18 50 0

18 51 0

18 52 0

18 53 0

18 54 0

18 55 0

18 56 0

18 57 0

18 58 0

18 59 0

18 60 0

18 61 0

18 62 0

18 63 0

18 64 0

18 65 0

18 66 0

18 67 0

18 68 0

18 69 0

18 70 0

18 71 0

18 72 0

18 73 0

18 74 0

18 75 0.0117834563433622

18 76 0.0145287006376433

19 1 0

19 2 0

19 3 0

19 4 0

19 5 0

19 6 0

19 7 0

19 8 0.00405292222132925

19 9 0.00234591890956093

19 10 0

19 11 0

19 12 0

19 13 0

19 14 0

19 15 0

19 16 0

19 17 0

19 18 0

19 19 0

19 20 0

19 21 0

19 22 0

19 23 0

19 24 0

19 25 0

19 26 0

19 27 0

19 28 0

19 29 0

19 30 0

19 31 0

19 32 0

19 33 0

19 34 0

19 35 0

19 36 0

19 37 0

19 38 0

19 39 0

19 40 0.000159583454135026

19 41 0

19 42 0

19 43 0

19 44 0

19 45 0

19 46 0.000308055185322620

19 47 0.00106179945805618

19 48 0

19 49 7.78675093598968e-05

19 50 0

19 51 0

19 52 0

19 53 0

19 54 0

19 55 0

19 56 0

19 57 0

19 58 0

19 59 0.000132878826226035

19 60 0

19 61 0

19 62 0

19 63 0

19 64 0

19 65 0

19 66 0

19 67 0

19 68 0

19 69 0

19 70 0

19 71 0

19 72 0

19 73 0

19 74 0

19 75 0.0117846059695017

19 76 0.0140784802864706

20 1 0

20 2 0

20 3 0

20 4 0

20 5 0

20 6 0

20 7 0

20 8 0.00463175704411545

20 9 0.00282289150287052

20 10 0

20 11 0

20 12 0

20 13 0

20 14 0

20 15 0

20 16 0

20 17 0

20 18 0

20 19 0

20 20 0

20 21 0

20 22 0

20 23 0

20 24 0

20 25 0

20 26 2.34061279060782e-05

20 27 0

20 28 6.42279013637892e-05

20 29 0

20 30 0

20 31 0

20 32 0

20 33 0

20 34 0

20 35 0

20 36 0

20 37 0

20 38 0

20 39 0

20 40 0.00160074597479498

20 41 0.00137481880471646

20 42 0

20 43 1.13781003874269e-05

20 44 0

20 45 0

20 46 0.000652137320250934

20 47 0.00136956338560723

20 48 0

20 49 0.00167161481248757

20 50 0.00348296411876436

20 51 0.00144412895348223

20 52 9.41878440806621e-05

20 53 0

20 54 0

20 55 0

20 56 0.00136617771283898

20 57 0.000707185220482409

20 58 0.00238729650175251

20 59 0.00310103470193546

20 60 0.00158560994446499

20 61 0.000322996729095970

20 62 0

20 63 0

20 64 0

20 65 0

20 66 0

20 67 0

20 68 0

20 69 0

20 70 0

20 71 0

20 72 0.00127927077334378

20 73 0.00784626495132745

20 74 0.00472555785741824

20 75 0.0140375161697253

20 76 0.0170783266845154

21 1 0

21 2 0

21 3 0

21 4 0

21 5 0

21 6 0

21 7 0

21 8 0.00480079786856646

21 9 0.00286733086657365

21 10 0

21 11 0

21 12 0

21 13 0

21 14 0

21 15 0

21 16 0

21 17 0

21 18 0

21 19 0

21 20 0

21 21 0

21 22 0

21 23 0

21 24 0

21 25 0

21 26 0.000222802317990323

21 27 0

21 28 0.000285725605132958

21 29 0

21 30 0

21 31 0

21 32 0

21 33 0

21 34 0

21 35 0

21 36 0

21 37 0

21 38 0

21 39 0

21 40 0.00180020187409657

21 41 0.00171814031621298

21 42 0

21 43 6.61199544799151e-05

21 44 0.000235791524736073

21 45 0.000147674311218315

21 46 0.000784102476785398

21 47 0.00148951287264187

21 48 0

21 49 0.00156870968681133

21 50 0.00309632902901957

21 51 0.00148772414736797

21 52 0.000361728783587090

21 53 0

21 54 0

21 55 0

21 56 0.00127814340195020

21 57 0.000623346655703849

21 58 0.00265857927770652

21 59 0.00302155448854868

21 60 0.00148391611335241

21 61 0.000275211355099003

21 62 0

21 63 0

21 64 0

21 65 0

21 66 0

21 67 0

21 68 0

21 69 0

21 70 0

21 71 0

21 72 0.00157675181249251

21 73 0.00794159308659448

21 74 0.00453575968653075

21 75 0.0139952968376997

21 76 0.0168180762950929

22 1 0.00123832667175927

22 2 0.00118869375015851

22 3 0.000808049260293764

22 4 0.000759361090010291

22 5 0.00102079621456463

22 6 0.000100978318869238

22 7 0.000289899176345587

22 8 0.00689164264087105

22 9 0.00474706120238100

22 10 0.000577042086877699

22 11 0.00113943280007001

22 12 0.00184104630373594

22 13 0.00149829688394554

22 14 0.000714601955043470

22 15 0.000672409017085873

22 16 0.00114338501913425

22 17 0.000282628868948587

22 18 0.00135487235768372

22 19 0.00324737222537364

22 20 0.00819913754892021

22 21 0.00841509548697861

22 22 0

22 23 0.0100454117158473

22 24 0.00607430324329361

22 25 0.00836955963364705

22 26 0.0101336450222062

22 27 0.0121832070182734

22 28 0.0102780689924168

22 29 0.000974564257619992

22 30 0.000692624227100702

22 31 0.00384320964189600

22 32 0.000871377231048021

22 33 0.000733363396551390

22 34 0.000374606543172784

22 35 0.000535122605495819

22 36 0.00154187505574643

22 37 0.00426611077988437

22 38 0.00411087773416197

22 39 0.0109330259365423

22 40 0.00760352354996319

22 41 0.00628042054010580

22 42 0.00132012293924411

22 43 0.00130493512706154

22 44 0.00436452368083118

22 45 0.00682930555768690

22 46 0.00505277354771372

22 47 0.00398636061517721

22 48 0.00170533389170680

22 49 0.00519367406061255

22 50 0.00760039845207738

22 51 0.00575039121553034

22 52 0.0107542751242145

22 53 0.00481722941364504

22 54 0.00630323993884985

22 55 0.00497614709905236

22 56 0.0111731292357006

22 57 0.0109877499772680

22 58 0.0114335130355532

22 59 0.00908548057244918

22 60 0.00377208270333140

22 61 0.00193492644386428

22 62 0.00106166608133140

22 63 0.00112470594475200

22 64 0.000724280068877814

22 65 0.000941733838557779

22 66 0.000471316471197247

22 67 0.00103678867693158

22 68 0.00325507733524288

22 69 0.00329051085005172

22 70 0.00240225551196416

22 71 0.00154065480797461

22 72 0.0169988716729443

22 73 0.0238532083038545

22 74 0.0112604008052719

22 75 0.0175546223480090

22 76 0.0243349280836715

23 1 0.00269020866532710

23 2 0.00294605992443242

23 3 0.00206574407722548

23 4 0.00177998368677812

23 5 0.00205910741941395

23 6 0.000889410131854396

23 7 0.00128554059452135

23 8 0.00794860963687016

23 9 0.00573987289239375

23 10 0.00161124509250876

23 11 0.00214579570733953

23 12 0.00286850864741306

23 13 0.00281028738640299

23 14 0.00206675409798796

23 15 0.00184024547203532

23 16 0.00236358621642174

23 17 0.00131710332087187

23 18 0.00220085815936644

23 19 0.00372146056171674

23 20 0.00654442769268715

23 21 0.00665731732517849

23 22 0.00842719296897410

23 23 0

23 24 0.00654357956291862

23 25 0.00662865974182691

23 26 0.00918737349567367

23 27 0.0105519410840493

23 28 0.00883242498221770

23 29 0.00196351921058868

23 30 0.00170375864318340

23 31 0.00400441732963297

23 32 0.00193218207062085

23 33 0.00226529325488090

23 34 0.00193246466249808

23 35 0.00179517314382760

23 36 0.00277446929858427

23 37 0.00702550066712893

23 38 0.00700142424113226

23 39 0.0152916769138176

23 40 0.00958824907742950

23 41 0.00519651753559791

23 42 0.00238934902610688

23 43 0.00213498850576266

23 44 0.00641061650879027

23 45 0.00873750244807803

23 46 0.00643037720346973

23 47 0.00538726934734191

23 48 0.00269954127882865

23 49 0.00811066275037897

23 50 0.0102224580269029

23 51 0.00559994562500488

23 52 0.00993584350963439

23 53 0.0132885006892505

23 54 0.0111712485815529

23 55 0.0112852801040209

23 56 0.0116655469515801

23 57 0.0115154637974935

23 58 0.0106555337366332

23 59 0.00990760012669611

23 60 0.00460696981398900

23 61 0.00293573782051371

23 62 0.00213407126161924

23 63 0.00237502880658080

23 64 0.00224092552662258

23 65 0.00196804056752542

23 66 0.00153737324225178

23 67 0.00178173302235785

23 68 0.00381191345678766

23 69 0.00398692482280272

23 70 0.00278705059860374

23 71 0.00245915261307117

23 72 0.0204851078571926

23 73 0.0225195115792476

23 74 0.0142529523110043

23 75 0.0201229879616200

23 76 0.0288626035575355

24 1 0.00214599927776482

24 2 0.00198056444978356

24 3 0.00168050816533161

24 4 0.00150974020837380

24 5 0.00190152175071023

24 6 0.000998415774148720

24 7 0.00119764270368705

24 8 0.00779965917560954

24 9 0.00562629246600455

24 10 0.00147118024243054

24 11 0.00200869611540988

24 12 0.00273109674773520

24 13 0.00232834564796758

24 14 0.00157909779763976

24 15 0.00162365709694756

24 16 0.00199533297591681

24 17 0.00118279316414227

24 18 0.00214616827150149

24 19 0.00376980076088329

24 20 0.00761037556271094

24 21 0.00774450790554204

24 22 0.00308589150447802

24 23 0.00627762772519080

24 24 0

24 25 0.00727823852758047

24 26 0.00930596389294236

24 27 0.00988805574372342

24 28 0.00928852318145212

24 29 0.00182373292488358

24 30 0.00155151766974260

24 31 0.00409984941185426

24 32 0.00179855472646451

24 33 0.00161874472870238

24 34 0.00121134605336504

24 35 0.00142148620628513

24 36 0.00248913469208389

24 37 0.00519098865940326

24 38 0.00493253591350162

24 39 0.0114076393820757

24 40 0.00903555799131972

24 41 0.00757277639413878

24 42 0.00208435473663826

24 43 0.00232457262589925

24 44 0.00505767182430628

24 45 0.00717261190921759

24 46 0.00563961739648033

24 47 0.00479967660268976

24 48 0.00250133980555578

24 49 0.00619947597499526

24 50 0.00904080472808744

24 51 0.00730750440527173

24 52 0.00988994516978514

24 53 0.00543909077101601

24 54 0.00649755245975148

24 55 0.00667939909079807

24 56 0.0113004787410623

24 57 0.0111072587158746

24 58 0.0117203665887196

24 59 0.0100709122547547

24 60 0.00479644539095703

24 61 0.00281597487347784

24 62 0.00200025497512035

24 63 0.00203534124147764

24 64 0.00160561779605162

24 65 0.00182768030547664

24 66 0.00135065970160098

24 67 0.00193261639067621

24 68 0.00396526825295662

24 69 0.00404783924889274

24 70 0.00308519706226296

24 71 0.00240561672303941

24 72 0.0167890743713972

24 73 0.0243679435052268

24 74 0.0119714497649139

24 75 0.0182133963583653

24 76 0.0248246748744407

25 1 0

25 2 0

25 3 0

25 4 0

25 5 0

25 6 0

25 7 0

25 8 0.00444773494182704

25 9 0.00256356702665206

25 10 0

25 11 0

25 12 0

25 13 0

25 14 0

25 15 0

25 16 0

25 17 0

25 18 0

25 19 0

25 20 0

25 21 0

25 22 0

25 23 0

25 24 0

25 25 0

25 26 0.000117803934326366

25 27 0

25 28 0.000363054387014505

25 29 0

25 30 0

25 31 0

25 32 0

25 33 0

25 34 0

25 35 0

25 36 0

25 37 0

25 38 0

25 39 0

25 40 0.00205257963773753

25 41 0.00321028201456841

25 42 0

25 43 0

25 44 0.000191264208843633

25 45 0.000886240609676292

25 46 0.000941404465383267

25 47 0.00127151513494450

25 48 0

25 49 0.000640939542984609

25 50 0.00127141550119214

25 51 0.00173243690116009

25 52 0.00172910221250600

25 53 0

25 54 0

25 55 0

25 56 0.00175011499682076

25 57 0.00119789673954085

25 58 0.00320485472527654

25 59 0.00325172683010955

25 60 0.00111572375036306

25 61 0

25 62 0

25 63 0

25 64 0

25 65 0

25 66 0

25 67 0

25 68 0

25 69 0

25 70 0

25 71 0

25 72 0.00261470340777747

25 73 0.00724147690283838

25 74 0.00366467985187324

25 75 0.0127545706700039

25 76 0.0147631241643484

26 1 0

26 2 0

26 3 0

26 4 0

26 5 0

26 6 0

26 7 0

26 8 0.00462485636414556

26 9 0.00261686919533710

26 10 0

26 11 0

26 12 0

26 13 0

26 14 0

26 15 0

26 16 0

26 17 0

26 18 0

26 19 0

26 20 0.000324220054171098

26 21 0.000108646190308259

26 22 0.000173365103293022

26 23 0.00122398395498025

26 24 0.000514648659880157

26 25 0

26 26 0

26 27 0

26 28 0.000209405594103318

26 29 0

26 30 0

26 31 0

26 32 0

26 33 0

26 34 0

26 35 0

26 36 0

26 37 0

26 38 0

26 39 0.00119434634270921

26 40 0.00110284860130216

26 41 0.000492770343572979

26 42 0

26 43 0

26 44 0.000160951095173001

26 45 0.00100208895742004

26 46 0.00160998366214948

26 47 0.00167344680706771

26 48 0

26 49 0.00116421117553345

26 50 0.000676218514191973

26 51 0

26 52 0.00149209476727485

26 53 0.00194244066453453

26 54 0.00208284941661563

26 55 0.00168524506313650

26 56 0.00169918617541376

26 57 0.00133150443141650

26 58 0.00143379422768886

26 59 0.00161529766462054

26 60 0.000342432551937777

26 61 0

26 62 0

26 63 0

26 64 0

26 65 0

26 66 0

26 67 0

26 68 0

26 69 0

26 70 0

26 71 0

26 72 0.00333229196055029

26 73 0.00489171641080088

26 74 0.00200853547801094

26 75 0.0128607533027076

26 76 0.0175259219487063

27 1 0.00124621185370488

27 2 0.00103516101744616

27 3 0.000830530721993461

27 4 0.000715408507537374

27 5 0.00103188816110977

27 6 0

27 7 0.000287693114698118

27 8 0.00691216896368874

27 9 0.00476731153010357

27 10 0.000599799866085160

27 11 0.00111473354503255

27 12 0.00181353887321500

27 13 0.00145407511744611

27 14 0

27 15 0.000730710926483025

27 16 0.00112383077425160

27 17 0.000326894088622409

27 18 0.000970989643414910

27 19 0.00193248454639849

27 20 0.00521095159702922

27 21 0.00502610098281986

27 22 0.00552563270437434

27 23 0.00769521027972386

27 24 0.00576559650920028

27 25 0.00395633283598329

27 26 0.00556622855624056

27 27 0

27 28 0.00538259737657987

27 29 0.00106674417331143

27 30 0.000759966520359701

27 31 0.00248807480697444

27 32 0.000951005838348951

27 33 0.000410123373539850

27 34 6.07566578181284e-06

27 35 0.000579931691307878

27 36 0.00151285740102991

27 37 0.00328370648136561

27 38 0.00307660339655175

27 39 0.00859521686996034

27 40 0.00480434148871622

27 41 0.00314158438728462

27 42 0.00145442086238445

27 43 0.00125658029982834

27 44 0.00306847500534457

27 45 0.00448702209608720

27 46 0.00438975195049274

27 47 0.00401243001687746

27 48 0.00160297998069980

27 49 0.00495352371024049

27 50 0.00566430234375259

27 51 0.00248457090356591

27 52 0.00770349211755628

27 53 0.00942731320307910

27 54 0.00941280623807361

27 55 0.00842847053668339

27 56 0.00741757713946800

27 57 0.00714598102201924

27 58 0.00694974292815220

27 59 0.00564558327635023

27 60 0.00284108088295021

27 61 0.00196475066825785

27 62 0.00114217421906648

27 63 0.00123608381975904

27 64 0.000370561862779173

27 65 0.000968126901077726

27 66 0.000492990001910054

27 67 0.000618772850051741

27 68 0.00221105938023314

27 69 0.00245880866391957

27 70 0.00206154493968924

27 71 0.00168235944826789

27 72 0.0131360938505063

27 73 0.0174165018199411

27 74 0.00795837316115466

27 75 0.0169347706831904

27 76 0.0250581775852856

28 1 0

28 2 0

28 3 0

28 4 0

28 5 0

28 6 0

28 7 0

28 8 0.00443550527015352

28 9 0.00243482979123549

28 10 0

28 11 0

28 12 0

28 13 0

28 14 0

28 15 0

28 16 0

28 17 0

28 18 0

28 19 0

28 20 0.000159543119458871

28 21 4.11142843913526e-05

28 22 0

28 23 0.000953561106356804

28 24 0

28 25 0

28 26 0

28 27 0

28 28 0

28 29 0

28 30 0

28 31 0

28 32 0

28 33 0

28 34 0

28 35 0

28 36 0

28 37 0

28 38 0

28 39 0

28 40 0.00104017080014629

28 41 0.00119641650505486

28 42 0

28 43 0

28 44 8.45932788691162e-05

28 45 0.00117370573630587

28 46 0.00115326161879004

28 47 0.00128621145827196

28 48 0

28 49 0.000439307643506415

28 50 0

28 51 0

28 52 0.00208999415672306

28 53 0.000572692236880279

28 54 0.00104340836045003

28 55 0.000627866509371522

28 56 0.00160786176055483

28 57 0.00141472469345361

28 58 0.00233153223437543

28 59 0.00187562987071788

28 60 0.000233850248136380

28 61 0

28 62 0

28 63 0

28 64 0

28 65 0

28 66 0

28 67 0

28 68 0

28 69 0

28 70 0

28 71 0

28 72 0.00239202977908426

28 73 0.00375827000576023

28 74 0.00217352000766669

28 75 0.0122528428619574

28 76 0.0154315631775115

29 1 0

29 2 0

29 3 0

29 4 0

29 5 0

29 6 0

29 7 0

29 8 0.00377774474425552

29 9 0.00208153815925838

29 10 0

29 11 0

29 12 0

29 13 0

29 14 0

29 15 0

29 16 0

29 17 0

29 18 0

29 19 0

29 20 0

29 21 0

29 22 0

29 23 0

29 24 0

29 25 0

29 26 0

29 27 0

29 28 0

29 29 0

29 30 0

29 31 0

29 32 0

29 33 0

29 34 0

29 35 0

29 36 0

29 37 0

29 38 0

29 39 0

29 40 0

29 41 0

29 42 0

29 43 0

29 44 0

29 45 0

29 46 1.81272463417592e-06

29 47 0.000790689571880110

29 48 0

29 49 0

29 50 0

29 51 0

29 52 0

29 53 0

29 54 0

29 55 0

29 56 0

29 57 0

29 58 0

29 59 0

29 60 0

29 61 0

29 62 0

29 63 0

29 64 0

29 65 0

29 66 0

29 67 0

29 68 0

29 69 0

29 70 0

29 71 0

29 72 0

29 73 0

29 74 0

29 75 0.0112132615217231

29 76 0.0132467907950506

30 1 0

30 2 0

30 3 0

30 4 0

30 5 0

30 6 0

30 7 0

30 8 0.00362862470652825

30 9 0.00197959833848194

30 10 0

30 11 0

30 12 0

30 13 0

30 14 0

30 15 0

30 16 0

30 17 0

30 18 0

30 19 0

30 20 0

30 21 0

30 22 0

30 23 0

30 24 0

30 25 0

30 26 0

30 27 0

30 28 0

30 29 0

30 30 0

30 31 0

30 32 0

30 33 0

30 34 0

30 35 0

30 36 0

30 37 0

30 38 0

30 39 0

30 40 0

30 41 0

30 42 0

30 43 0

30 44 0

30 45 0

30 46 0

30 47 0.000645807689958478

30 48 0

30 49 0

30 50 0

30 51 0

30 52 0

30 53 0

30 54 0

30 55 0

30 56 0

30 57 0

30 58 0

30 59 0

30 60 0

30 61 0

30 62 0

30 63 0

30 64 0

30 65 0

30 66 0

30 67 0

30 68 0

30 69 0

30 70 0

30 71 0

30 72 0

30 73 0

30 74 0

30 75 0.0111968703857865

30 76 0.0133267876331651

31 1 0

31 2 0

31 3 0

31 4 0

31 5 0

31 6 0

31 7 0

31 8 0.00410845779011032

31 9 0.00227744472815500

31 10 0

31 11 0

31 12 0

31 13 0

31 14 0

31 15 0

31 16 0

31 17 0

31 18 0

31 19 0

31 20 0

31 21 0

31 22 0

31 23 0

31 24 0

31 25 0

31 26 0

31 27 0

31 28 0

31 29 0

31 30 0

31 31 0

31 32 0

31 33 0

31 34 0

31 35 0

31 36 0

31 37 0

31 38 0

31 39 0

31 40 2.00348273542428e-05

31 41 0

31 42 0

31 43 0

31 44 0

31 45 0

31 46 0.000643473907297909

31 47 0.00109010146009042

31 48 0

31 49 4.02084164501382e-05

31 50 0

31 51 0

31 52 0

31 53 0

31 54 0

31 55 0

31 56 0

31 57 0

31 58 0

31 59 0

31 60 0

31 61 0

31 62 0

31 63 0

31 64 0

31 65 0

31 66 0

31 67 0

31 68 0

31 69 0

31 70 0

31 71 0

31 72 0

31 73 0

31 74 0

31 75 0.0115136938715718

31 76 0.0144566736438381

32 1 0

32 2 0

32 3 0

32 4 0

32 5 0

32 6 0

32 7 0

32 8 0.00379703504908713

32 9 0.00210989295333797

32 10 0

32 11 0

32 12 0

32 13 0

32 14 0

32 15 0

32 16 0

32 17 0

32 18 0

32 19 0

32 20 0

32 21 0

32 22 0

32 23 0

32 24 0

32 25 0

32 26 0

32 27 0

32 28 0

32 29 0

32 30 0

32 31 0

32 32 0

32 33 0

32 34 0

32 35 0

32 36 0

32 37 0

32 38 0

32 39 0

32 40 0

32 41 0

32 42 0

32 43 0

32 44 0

32 45 0

32 46 0

32 47 0.000789324785065215

32 48 0

32 49 0

32 50 0

32 51 0

32 52 0

32 53 0

32 54 0

32 55 0

32 56 0

32 57 0

32 58 0

32 59 0

32 60 0

32 61 0

32 62 0

32 63 0

32 64 0

32 65 0

32 66 0

32 67 0

32 68 0

32 69 0

32 70 0

32 71 0

32 72 0

32 73 0

32 74 0

32 75 0.0111664501620030

32 76 0.0129488771403092

33 1 0

33 2 0

33 3 0

33 4 0

33 5 0

33 6 0

33 7 0

33 8 0.00317695468534673

33 9 0.00148889043998279

33 10 0

33 11 0

33 12 0

33 13 0

33 14 0

33 15 0

33 16 0

33 17 0

33 18 0

33 19 0

33 20 0

33 21 0

33 22 0

33 23 0

33 24 0

33 25 0

33 26 0

33 27 0

33 28 0

33 29 0

33 30 0

33 31 0

33 32 0

33 33 0

33 34 0

33 35 0

33 36 0

33 37 0

33 38 0

33 39 0

33 40 0

33 41 0

33 42 0

33 43 0

33 44 0

33 45 0

33 46 0

33 47 0.000206707772097969

33 48 0

33 49 0

33 50 0

33 51 0

33 52 0

33 53 0

33 54 0

33 55 0

33 56 0

33 57 0

33 58 0

33 59 0

33 60 0

33 61 0

33 62 0

33 63 0

33 64 0

33 65 0

33 66 0

33 67 0

33 68 0

33 69 0

33 70 0

33 71 0

33 72 0

33 73 0

33 74 0

33 75 0.0122154915120165

33 76 0.0135906228817415

34 1 0

34 2 0

34 3 0

34 4 0

34 5 0

34 6 0

34 7 0

34 8 0.00338840366195203

34 9 0.00170219670304628

34 10 0

34 11 0

34 12 0

34 13 0

34 14 0

34 15 0

34 16 0

34 17 0

34 18 0

34 19 0

34 20 0

34 21 0

34 22 0

34 23 0

34 24 0

34 25 0

34 26 0

34 27 0

34 28 0

34 29 0

34 30 0

34 31 0

34 32 0

34 33 0

34 34 0

34 35 0

34 36 0

34 37 0

34 38 0

34 39 0

34 40 0

34 41 0

34 42 0

34 43 0

34 44 0

34 45 0

34 46 0

34 47 0.000412948253382006

34 48 0

34 49 0

34 50 0

34 51 0

34 52 0

34 53 0

34 54 0

34 55 0

34 56 0

34 57 0

34 58 0

34 59 0

34 60 0

34 61 0

34 62 0

34 63 0

34 64 0

34 65 0

34 66 0

34 67 0

34 68 0

34 69 0

34 70 0

34 71 0

34 72 0

34 73 0

34 74 0

34 75 0.0118867764505533

34 76 0.0135720348498585

35 1 0

35 2 0

35 3 0

35 4 0

35 5 0

35 6 0

35 7 0

35 8 0.00380744027255586

35 9 0.00209411597698317

35 10 0

35 11 0

35 12 0

35 13 0

35 14 0

35 15 0

35 16 0

35 17 0

35 18 0

35 19 0

35 20 0

35 21 0

35 22 0

35 23 0

35 24 0

35 25 0

35 26 0

35 27 0

35 28 0

35 29 0

35 30 0

35 31 0

35 32 0

35 33 0

35 34 0

35 35 0

35 36 0

35 37 0

35 38 0

35 39 0

35 40 0

35 41 0

35 42 0

35 43 0

35 44 0

35 45 0

35 46 0

35 47 0.000786084940702536

35 48 0

35 49 0

35 50 0

35 51 0

35 52 0

35 53 0

35 54 0

35 55 0

35 56 0

35 57 0

35 58 0

35 59 0

35 60 0

35 61 0

35 62 0

35 63 0

35 64 0

35 65 0

35 66 0

35 67 0

35 68 0

35 69 0

35 70 0

35 71 0

35 72 0

35 73 0

35 74 0

35 75 0.0115273866361032

35 76 0.0134079084580394

36 1 0

36 2 0

36 3 0

36 4 0

36 5 0

36 6 0

36 7 0

36 8 0.00441100376824061

36 9 0.00270618009372575

36 10 0

36 11 0

36 12 0

36 13 0

36 14 0

36 15 0

36 16 0

36 17 0

36 18 0

36 19 0

36 20 0

36 21 0

36 22 0

36 23 0

36 24 0

36 25 0

36 26 0

36 27 0

36 28 0

36 29 0

36 30 0

36 31 0

36 32 0

36 33 0

36 34 0

36 35 0

36 36 0

36 37 0

36 38 0

36 39 0

36 40 0.000544629629529170

36 41 0

36 42 0

36 43 0

36 44 0

36 45 0

36 46 0.000588626611326359

36 47 0.00144590394268873

36 48 0

36 49 0.000455101450147211

36 50 0

36 51 0

36 52 0

36 53 0

36 54 0

36 55 0

36 56 0

36 57 0

36 58 0

36 59 0.000263450607268401

36 60 0.000197042091685695

36 61 0

36 62 0

36 63 0

36 64 0

36 65 0

36 66 0

36 67 0

36 68 0

36 69 0

36 70 0

36 71 0

36 72 0

36 73 0

36 74 0

36 75 0.0117979526417948

36 76 0.0138764455317084

37 1 0

37 2 0.000102150465897621

37 3 0

37 4 0

37 5 0

37 6 0

37 7 0

37 8 0.00435875420073340

37 9 0.00264397877894473

37 10 0

37 11 0

37 12 0

37 13 0

37 14 0

37 15 0

37 16 0

37 17 0

37 18 0

37 19 0

37 20 0

37 21 0

37 22 0.000331486007474902

37 23 0

37 24 0.000585869516387505

37 25 0

37 26 0.00147276191688134

37 27 0.00214960544121506

37 28 0.000728827507224805

37 29 0

37 30 0

37 31 0.00129442788515044

37 32 0

37 33 0

37 34 0

37 35 0

37 36 0

37 37 0

37 38 0

37 39 0

37 40 0.000642892536376172

37 41 0

37 42 0

37 43 0

37 44 0.000500756365338362

37 45 0.00113963506602194

37 46 0.000915177335160833

37 47 0.00158354396353799

37 48 0

37 49 0.000596033871360036

37 50 0

37 51 0

37 52 9.25327088633354e-05

37 53 0.000355308630077600

37 54 0.000432960639149549

37 55 0

37 56 8.48518741585758e-05

37 57 0

37 58 0

37 59 0.000508783275139146

37 60 0.000171570600883131

37 61 0

37 62 0

37 63 0

37 64 0

37 65 0

37 66 0

37 67 0

37 68 0

37 69 0

37 70 0

37 71 0

37 72 0

37 73 0

37 74 0

37 75 0.0118017784753530

37 76 0.0151616690033680

38 1 0

38 2 0.000426322845944072

38 3 0

38 4 0

38 5 0

38 6 0

38 7 0

38 8 0.00441413529624640

38 9 0.00269141094466419

38 10 0

38 11 0

38 12 0

38 13 0.000139864536923384

38 14 0

38 15 0

38 16 9.64338865311332e-05

38 17 0

38 18 0

38 19 0

38 20 0

38 21 0

38 22 0.000510283885040597

38 23 0

38 24 0.000715365520085221

38 25 0

38 26 0.00192898335650904

38 27 0.00272325879202479

38 28 0.00104762739406322

38 29 0

38 30 0

38 31 0.00178796233884557

38 32 0

38 33 0

38 34 0

38 35 0

38 36 0

38 37 0

38 38 0

38 39 0

38 40 0.000661346400785079

38 41 0

38 42 0

38 43 0

38 44 0.000675881994479877

38 45 0.00130236559889374

38 46 0.000889295707721727

38 47 0.00158497988690914

38 48 0

38 49 0.000651283098329181

38 50 0

38 51 0

38 52 0.000183442858963989

38 53 0.000474179708047973

38 54 0.000478077347155659

38 55 4.66258228779548e-05

38 56 8.37929461834364e-05

38 57 0

38 58 0

38 59 0.000500517275545231

38 60 0.000217176787845941

38 61 0

38 62 0

38 63 0

38 64 0

38 65 0

38 66 0

38 67 0

38 68 0

38 69 0

38 70 0

38 71 0

38 72 0

38 73 0

38 74 0

38 75 0.0119019190794513

38 76 0.0152603152992008

39 1 0

39 2 0.000415619382039378

39 3 0

39 4 0

39 5 0

39 6 0

39 7 0

39 8 0.00428100740396142

39 9 0.00255778672162277

39 10 0

39 11 0

39 12 0

39 13 0.000420264686935012

39 14 0

39 15 0

39 16 0

39 17 0

39 18 0

39 19 0

39 20 0.00214922634196058

39 21 0.00214164331247813

39 22 0.00684305677977570

39 23 0.00606659666312814

39 24 0.00687998517474886

39 25 0.00157746355365485

39 26 0.00453002869808861

39 27 0.00702839344361639

39 28 0.00330387459563530

39 29 0

39 30 0

39 31 0.00184944364466288

39 32 0

39 33 0

39 34 0

39 35 0

39 36 0

39 37 0.000154186118709987

39 38 0.000197455786242806

39 39 0

39 40 0.00188653725865140

39 41 0

39 42 0

39 43 0

39 44 0.000846197810464355

39 45 0.00144538273871420

39 46 0.00113623136084684

39 47 0.00163221966794558

39 48 0

39 49 0.00217536616616199

39 50 0.00196613266446732

39 51 0

39 52 0.00573302542172116

39 53 0.00995058341711297

39 54 0.00962002568017084

39 55 0.00621545665997392

39 56 0.00210999822433555

39 57 0.00199651835682924

39 58 0.00113412168759142

39 59 0.00124824979392946

39 60 0.000155437402418857

39 61 0

39 62 0

39 63 0

39 64 0.000385770399716767

39 65 0

39 66 0

39 67 0

39 68 0

39 69 0

39 70 0

39 71 0

39 72 0.00463462740734721

39 73 0.00352499170237375

39 74 0.00140184822635447

39 75 0.0142125646176166

39 76 0.0231825506403194

40 1 0.000997313403028222

40 2 0.00140425730782112

40 3 0.00215267619168458

40 4 0.00256516742287005

40 5 0.000905759410561924

40 6 0

40 7 0

40 8 0.00577306965956392

40 9 0.00398641748863082

40 10 0

40 11 0.000635798173549973

40 12 0.00123532861506637

40 13 0.00213127542855851

40 14 0.000373636657784893

40 15 0.000331315463206638

40 16 0.000361768857497724

40 17 0

40 18 0.000112158925520300

40 19 0.000425174903724068

40 20 0

40 21 0

40 22 0.000965873839552156

40 23 0.000742289111748784

40 24 0.00126885966403545

40 25 0.000135702676721805

40 26 0.000852153139134004

40 27 0.000935498569404114

40 28 0.000831071951929552

40 29 0.000283385713048312

40 30 0.000179583804866379

40 31 0.000888510009836918

40 32 0.000155706361362418

40 33 1.69933287663682e-06

40 34 0

40 35 0

40 36 0.000636894105893515

40 37 0.00110298669454889

40 38 0.00103120455854111

40 39 0.000532694208381646

40 40 0.000715672071278650

40 41 0.00186960557526406

40 42 0

40 43 0.000685774534727290

40 44 0.00149772914389501

40 45 0.00223083200579255

40 46 0.00251811694266524

40 47 0.00281173945504476

40 48 0.000757616503871295

40 49 0.00240451072432557

40 50 0.00287197469668510

40 51 0.00243129518301122

40 52 0.00351125590101042

40 53 0.00412133169056683

40 54 0.00432007706567816

40 55 0.00215904014603963

40 56 0.00161297981909936

40 57 0.00130976734661326

40 58 0.00171027078106167

40 59 0.00264257791395195

40 60 0.00155870722529594

40 61 0.00126615251879092

40 62 0.000326169421784872

40 63 0.00140435102199898

40 64 0.00290943551848932

40 65 0.000498565387313632

40 66 0

40 67 0

40 68 0

40 69 8.23852061682118e-06

40 70 0.000823859716559738

40 71 0.00109717442851920

40 72 0.00214938971422707

40 73 0

40 74 0.00211191918732689

40 75 0.0163886351805060

40 76 0.0205808281931159

41 1 0

41 2 0

41 3 0

41 4 0

41 5 0

41 6 0

41 7 0

41 8 0.00400317896909042

41 9 0.00227200629689883

41 10 0

41 11 0

41 12 0

41 13 0

41 14 0

41 15 0

41 16 0

41 17 0

41 18 0

41 19 0

41 20 0

41 21 0

41 22 0

41 23 0

41 24 0

41 25 0

41 26 0

41 27 0

41 28 0

41 29 0

41 30 0

41 31 0

41 32 0

41 33 0

41 34 0

41 35 0

41 36 0

41 37 0

41 38 0

41 39 0

41 40 0.000267437056007180

41 41 0.000136460930074689

41 42 0

41 43 0

41 44 0

41 45 0.000258586431042041

41 46 0.00107846815398249

41 47 0.00101838377331509

41 48 0

41 49 0.000141719530515227

41 50 0

41 51 0

41 52 0

41 53 0

41 54 0

41 55 0

41 56 0

41 57 0

41 58 0.000189869144848642

41 59 0.000608356037708591

41 60 0

41 61 0

41 62 0

41 63 0

41 64 0

41 65 0

41 66 0

41 67 0

41 68 0

41 69 0

41 70 0

41 71 0

41 72 0

41 73 0

41 74 0.000148336594121834

41 75 0.0120858651453255

41 76 0.0147867935000829

42 1 0

42 2 0

42 3 0

42 4 0

42 5 0

42 6 0

42 7 0

42 8 0.00432012887200151

42 9 0.00256327971053172

42 10 0

42 11 0

42 12 0

42 13 0

42 14 0

42 15 0

42 16 0

42 17 0

42 18 0

42 19 0

42 20 0

42 21 0

42 22 0

42 23 0

42 24 0

42 25 0

42 26 0

42 27 0

42 28 0

42 29 0

42 30 0

42 31 0

42 32 0

42 33 0

42 34 0

42 35 0

42 36 0

42 37 0

42 38 0

42 39 0

42 40 0.000747238574318243

42 41 0.000159210948457500

42 42 0

42 43 0.000356836894653600

42 44 0

42 45 0

42 46 0.000534116420024322

42 47 0.00132441859359167

42 48 0

42 49 0.000736326769879336

42 50 9.37165636007986e-05

42 51 0

42 52 0

42 53 0

42 54 0.000216515916693139

42 55 0

42 56 0

42 57 0

42 58 0.000499684210082951

42 59 0.000330542294154856

42 60 0.000131493910010771

42 61 0

42 62 0

42 63 0

42 64 0

42 65 0

42 66 0

42 67 0

42 68 0

42 69 0

42 70 0.000132256149547594

42 71 0.000215181623716698

42 72 0

42 73 0

42 74 0.00107250224356736

42 75 0.0130552316828103

42 76 0.0152599289123727

43 1 0

43 2 0

43 3 0

43 4 0

43 5 0

43 6 0

43 7 0

43 8 0.00305208908446053

43 9 0.00128287931672411

43 10 0

43 11 0

43 12 0

43 13 0

43 14 0

43 15 0

43 16 0

43 17 0

43 18 0

43 19 0

43 20 0

43 21 0

43 22 0

43 23 0

43 24 0

43 25 0

43 26 0

43 27 0

43 28 0

43 29 0

43 30 0

43 31 0

43 32 0

43 33 0

43 34 0

43 35 0

43 36 0

43 37 0

43 38 0

43 39 0

43 40 0

43 41 0

43 42 0

43 43 0

43 44 0

43 45 0

43 46 0

43 47 0

43 48 0

43 49 0

43 50 0

43 51 0

43 52 0

43 53 0

43 54 0

43 55 0

43 56 0

43 57 0

43 58 0

43 59 0

43 60 0

43 61 0

43 62 0

43 63 0

43 64 0

43 65 0

43 66 0

43 67 0

43 68 0

43 69 0

43 70 0

43 71 0

43 72 0

43 73 0

43 74 0

43 75 0.0113279337485832

43 76 0.0136531619726117

44 1 0

44 2 0

44 3 0

44 4 0

44 5 0

44 6 0

44 7 0

44 8 0.00480960257486529

44 9 0.00305249226406468

44 10 0

44 11 0

44 12 7.54048957460896e-05

44 13 0

44 14 0

44 15 0

44 16 0

44 17 0

44 18 0

44 19 0

44 20 0

44 21 0

44 22 0.000577946016252051

44 23 0

44 24 0.000496540703353365

44 25 0

44 26 0.000549772098809909

44 27 0.000653647844152228

44 28 0.000314993632242744

44 29 0

44 30 0

44 31 0

44 32 0

44 33 0

44 34 0

44 35 0

44 36 0

44 37 0.000768667838646930

44 38 0.000929893860869224

44 39 0.000231842060423704

44 40 0.00102591592024825

44 41 0.000291215242221310

44 42 0

44 43 0

44 44 0

44 45 5.58515313142660e-05

44 46 0.00169418443445857

44 47 0.00210074698712481

44 48 8.50822236753146e-05

44 49 0.00136904095646684

44 50 0.000431096826392086

44 51 0

44 52 0.000135825989643457

44 53 0

44 54 0

44 55 0.000597071618016476

44 56 0.000717513005723869

44 57 0.000399325740555123

44 58 0.000491104644781748

44 59 0.00111028590722517

44 60 0.000618120003141653

44 61 0.000217013833554880

44 62 0

44 63 0

44 64 0

44 65 0

44 66 0

44 67 0

44 68 0

44 69 0

44 70 0

44 71 0

44 72 0

44 73 0

44 74 0.00102714032167761

44 75 0.0124683834724975

44 76 0.0161872782995136

45 1 0

45 2 0

45 3 0

45 4 0

45 5 0

45 6 0

45 7 0

45 8 0.00383793391542975

45 9 0.00217396351084487

45 10 0

45 11 0

45 12 0

45 13 0

45 14 0

45 15 0

45 16 0

45 17 0

45 18 0

45 19 0

45 20 0

45 21 0

45 22 0.00346778556319416

45 23 0.00198717126740333

45 24 0.00347977008627742

45 25 0.000184326157517956

45 26 0.00264871350235962

45 27 0.00472480373488182

45 28 0.00164250840689240

45 29 0

45 30 0

45 31 0.000165051812703210

45 32 0

45 33 0

45 34 0

45 35 0

45 36 0

45 37 0.00124189295446303

45 38 0.00138405873298325

45 39 0.00133816170418832

45 40 0.000141484330230868

45 41 0

45 42 0

45 43 0

45 44 0

45 45 0

45 46 0.00225330543440067

45 47 0.00175508508392452

45 48 0

45 49 0.000353834002881803

45 50 0

45 51 0

45 52 0.00242956427799901

45 53 0.00210559649488973

45 54 0.00238847667265252

45 55 0.00265142084534398

45 56 0.00103555196187111

45 57 0.00104302502746090

45 58 0.000891866631474114

45 59 0.000549055259250308

45 60 0

45 61 0

45 62 0

45 63 0

45 64 0

45 65 0

45 66 0

45 67 0

45 68 0

45 69 0

45 70 0

45 71 0

45 72 0

45 73 0

45 74 0.000405336297906844

45 75 0.0123466396986149

45 76 0.0184071099086232

46 1 0

46 2 0

46 3 0

46 4 0

46 5 0

46 6 0

46 7 0

46 8 0.00353743309730814

46 9 0.00187130565466898

46 10 0

46 11 0

46 12 0

46 13 0

46 14 0

46 15 0

46 16 0

46 17 0

46 18 0

46 19 0

46 20 0

46 21 0

46 22 0

46 23 0

46 24 0

46 25 0

46 26 0.000119642655916685

46 27 0.000646621347182741

46 28 0

46 29 0

46 30 0

46 31 0

46 32 0

46 33 0

46 34 0

46 35 0

46 36 0

46 37 0

46 38 0

46 39 0

46 40 0

46 41 0

46 42 0

46 43 0

46 44 0

46 45 0

46 46 0.000815262020597951

46 47 0.00108361118145794

46 48 0

46 49 0

46 50 0

46 51 0

46 52 0

46 53 0

46 54 0

46 55 0

46 56 0

46 57 0

46 58 0

46 59 0

46 60 0

46 61 0

46 62 0

46 63 0

46 64 0

46 65 0

46 66 0

46 67 0

46 68 0

46 69 0

46 70 0

46 71 0

46 72 0

46 73 0

46 74 0

46 75 0.0111334505936531

46 76 0.0143286122954513

47 1 0

47 2 0

47 3 0

47 4 0

47 5 0

47 6 0

47 7 0

47 8 0.00372461453988671

47 9 0.00201931658209775

47 10 0

47 11 0

47 12 0

47 13 0

47 14 0

47 15 0

47 16 0

47 17 0

47 18 0

47 19 0

47 20 0

47 21 0

47 22 0

47 23 0

47 24 0

47 25 0

47 26 0

47 27 0

47 28 0

47 29 0

47 30 0

47 31 0

47 32 0

47 33 0

47 34 0

47 35 0

47 36 0

47 37 0

47 38 0

47 39 0

47 40 0

47 41 0

47 42 0

47 43 0

47 44 0

47 45 0

47 46 0.000277708533386156

47 47 0.00167939781947329

47 48 0

47 49 0

47 50 0

47 51 0

47 52 0

47 53 0

47 54 0

47 55 0

47 56 0

47 57 0

47 58 0

47 59 0

47 60 0

47 61 0

47 62 0

47 63 0

47 64 0

47 65 0

47 66 0

47 67 0

47 68 0

47 69 0

47 70 0

47 71 0

47 72 0

47 73 0

47 74 0

47 75 0.0111517952713518

47 76 0.0134089549477432

48 1 0.000454879207005043

48 2 2.09056040277877e-05

48 3 0.000104484995675280

48 4 0

48 5 0.000243559874022692

48 6 0

48 7 0

48 8 0.00568179364582311

48 9 0.00401268226234119

48 10 0

48 11 0.000362346934705049

48 12 0.00100479281104948

48 13 0.000677285680633522

48 14 0

48 15 4.54632335288308e-05

48 16 0.000426697308516522

48 17 0

48 18 3.72165325615015e-05

48 19 0.000418250920301122

48 20 0

48 21 0

48 22 0

48 23 0

48 24 0

48 25 0

48 26 0

48 27 0

48 28 0.000228176366286914

48 29 0.000172264829270685

48 30 0

48 31 0.000320844075790139

48 32 0.000188580767106261

48 33 0

48 34 0

48 35 0

48 36 0.000607781337913838

48 37 0.000606794215617934

48 38 0.000649832394032490

48 39 0

48 40 0.00176213021723537

48 41 0.00119153227253466

48 42 0

48 43 0.000578429027234484

48 44 0.000857769147845300

48 45 0.00117336438319915

48 46 0.00183085490204549

48 47 0.00272982330136640

48 48 0

48 49 0.00177089564815758

48 50 0.00144568929225941

48 51 9.90421611650749e-05

48 52 0

48 53 0

48 54 0

48 55 0

48 56 0.000715408876879264

48 57 0.000410432654147974

48 58 0.00104861611449958

48 59 0.00166855011952349

48 60 0.00155853756975932

48 61 0.00120637088046183

48 62 0.000347871618408435

48 63 0.000300617316910001

48 64 0

48 65 0.000184852323373175

48 66 0

48 67 0

48 68 0

48 69 8.69724224977775e-05

48 70 0.000793735304178700

48 71 0.000736871699666519

48 72 0

48 73 5.56720782618481e-06

48 74 0.00158278857876570

48 75 0.0137702394595420

48 76 0.0159093165335047

49 1 0.000349466013625510

49 2 0

49 3 0

49 4 0

49 5 0

49 6 0

49 7 0

49 8 0.00538609608699725

49 9 0.00369255392943357

49 10 0

49 11 9.09603982726104e-05

49 12 0.000720279926512579

49 13 0.000438184683341225

49 14 0

49 15 0

49 16 9.44044989953374e-05

49 17 0

49 18 0

49 19 0.000466999289255310

49 20 0

49 21 0

49 22 0.000592698892000088

49 23 0.000425455286887755

49 24 0.000615906675736633

49 25 7.64565158961617e-06

49 26 0.000294191693441803

49 27 0.000105317127608640

49 28 0.000504836730525038

49 29 0

49 30 0

49 31 0.000351740459474170

49 32 0

49 33 0

49 34 0

49 35 0

49 36 0.000311725952833686

49 37 0.000332541733216063

49 38 0.000448095775646473

49 39 5.78239207243980e-05

49 40 0.00228188999978118

49 41 0.00101226367304574

49 42 0

49 43 0.000505164295104610

49 44 0.00106750162770619

49 45 0.000552903778698322

49 46 0.00163814293281472

49 47 0.00252961678845542

49 48 0.000533228174657840

49 49 0.000695011705463822

49 50 0.00174574905835545

49 51 0.000596217030260249

49 52 0.000719501641179621

49 53 0.000557506341411829

49 54 0.000751584134461192

49 55 0.000767272816059705

49 56 0.00106302841664119

49 57 0.000628661702920419

49 58 0.00114421451246116

49 59 0.00168253752353686

49 60 0.00135270282389521

49 61 0.000901908914891836

49 62 4.88510666977193e-05

49 63 8.33849918677743e-05

49 64 0

49 65 0

49 66 0

49 67 0

49 68 0

49 69 0

49 70 0.000936418486726764

49 71 0.000634081796567432

49 72 0

49 73 0

49 74 0.00173763043252650

49 75 0.0158406600542553

49 76 0.0176553741146341

50 1 0.000166168590560623

50 2 6.88238574463318e-05

50 3 0

50 4 0

50 5 0

50 6 0

50 7 0

50 8 0.00512257833004681

50 9 0.00345781043266169

50 10 0

50 11 0

50 12 0.000474878360651188

50 13 0.000290626373243397

50 14 0

50 15 0

50 16 0

50 17 0

50 18 0

50 19 6.01324839104578e-05

50 20 0

50 21 0

50 22 0.00104685178890307

50 23 0

50 24 0.00104486168509477

50 25 0

50 26 0.000318224552124935

50 27 0.000380324302616364

50 28 0.000458256875290441

50 29 0

50 30 0

50 31 0.000216280029171512

50 32 0

50 33 0

50 34 0

50 35 0

50 36 4.91231966830430e-05

50 37 0.000425929892175048

50 38 0.000566338066084637

50 39 0.000962528728716494

50 40 0.00307198563165401

50 41 0.00100465984153619

50 42 0

50 43 0.000577115951182705

50 44 0.00175550218645082

50 45 0.00141823390745166

50 46 0.00174422829729071

50 47 0.00237990248780262

50 48 0.000956998265005504

50 49 0.00284958999102236

50 50 0

50 51 0.000476997381457345

50 52 0.00120737252226721

50 53 0.000138893540871488

50 54 0

50 55 0.000747927545816429

50 56 0.000976430255605565

50 57 0.000605545379110173

50 58 0.00117285806842748

50 59 0.00157200384971090

50 60 0.00117899110733888

50 61 0.000659645777165707

50 62 0

50 63 0

50 64 0

50 65 0

50 66 0

50 67 0

50 68 0

50 69 0

50 70 0.000714195466650969

50 71 0.000456774081081357

50 72 0

50 73 0

50 74 0.00360517074686118

50 75 0.0158307580553256

50 76 0.0186206708432888

51 1 0.00280911845908605

51 2 0.00281476344526554

51 3 0.00259775534663231

51 4 0.00240271848398788

51 5 0.00241797888384476

51 6 0.00103397771135194

51 7 0.00157286836997750

51 8 0.00776296905881491

51 9 0.00603449868923933

51 10 0.00210463603834488

51 11 0.00244807665136593

51 12 0.00306133544896248

51 13 0.00304842110807024

51 14 0.00148165319901472

51 15 0.00211085607318795

51 16 0.00245242068759333

51 17 0.00164608021655310

51 18 0.00219278747674367

51 19 0.00244362982929447

51 20 0.00161497068974970

51 21 0.00155184691884047

51 22 0.00252631250872692

51 23 0.00113898116065636

51 24 0.00282107664390185

51 25 0.00231373356805953

51 26 0.00278090955589283

51 27 0.00236605997191308

51 28 0.00300466872271954

51 29 0.00215527050099773

51 30 0.00196020361641436

51 31 0.00275293682428079

51 32 0.00216119086843303

51 33 0.00139296149729784

51 34 0.00101448280718341

51 35 0.00168879110559983

51 36 0.00286237717537174

51 37 0.00410478334909936

51 38 0.00423502717290458

51 39 0.00401053788644812

51 40 0.00571109628294852

51 41 0.00374091432787604

51 42 0.00201242380068933

51 43 0.00280972773489041

51 44 0.00420790315993846

51 45 0.00543183212141085

51 46 0.00525125124298287

51 47 0.00511481917271328

51 48 0.00318575315659508

51 49 0.00485762823868496

51 50 0.00388648237280642

51 51 0

51 52 0.00512659559714535

51 53 0.00324571553377506

51 54 0.00512091575080453

51 55 0.00251054162969799

51 56 0.00327515046248295

51 57 0.00309832066717286

51 58 0.00408777402776317

51 59 0.00429955092243783

51 60 0.00374050083407984

51 61 0.00331462521869208

51 62 0.00236889701500764

51 63 0.00248119826967741

51 64 0.00214245308684391

51 65 0.00223256464401123

51 66 0.00167883477465125

51 67 0.00168107663375117

51 68 0.00217210479462437

51 69 0.00229497024682612

51 70 0.00300275218766088

51 71 0.00281690243331822

51 72 0.00309198398475052

51 73 0.00375140201317437

51 74 0.00837263802338184

51 75 0.0164333810709267

51 76 0.0198790735025276

52 1 0.00428388428170834

52 2 0.00463588525513048

52 3 0.00347361838115123

52 4 0.00311150672283889

52 5 0.00337394933095991

52 6 0.00219316343097620

52 7 0.00255947086517649

52 8 0.00887810604879857

52 9 0.00715459777037708

52 10 0.00307789406362424

52 11 0.00339353118965435

52 12 0.00417227501369388

52 13 0.00445320985598197

52 14 0.00476086141551990

52 15 0.00311120899526673

52 16 0.00353042659134561

52 17 0.00247788844807517

52 18 0.00317957290401727

52 19 0.00471830938727513

52 20 0.00781286292548145

52 21 0.00795686942198148

52 22 0.00954815729156522

52 23 0.0105958602411913

52 24 0.00998387850491533

52 25 0.00850713135362657

52 26 0.0113781309445657

52 27 0.0142047263306242

52 28 0.0114549394171779

52 29 0.00326933447957622

52 30 0.00304104153297557

52 31 0.00584304349141240

52 32 0.00317049350008969

52 33 0.00291069443907555

52 34 0.00240534759707489

52 35 0.00277678968776507

52 36 0.00425133225144159

52 37 0.00891995520274191

52 38 0.00904549523272624

52 39 0.0150389597919329

52 40 0.0106965587570622

52 41 0.00726038334074808

52 42 0.00361070459063284

52 43 0.00359672713776638

52 44 0.00781159758298378

52 45 0.0124880435778666

52 46 0.00929426722060711

52 47 0.00675610028114304

52 48 0.00404455781703250

52 49 0.00729703934539061

52 50 0.00766884150340719

52 51 0.00591638920781779

52 52 0

52 53 0.00223248454501168

52 54 0.00145575692393107

52 55 0.00730424059356594

52 56 0.0119200349714708

52 57 0.0120128259484368

52 58 0.0106873945187027

52 59 0.0104594452618652

52 60 0.00550971998859695

52 61 0.00426172098735544

52 62 0.00341855233557409

52 63 0.00370372768643357

52 64 0.00375590901176048

52 65 0.00324674302504357

52 66 0.00267624667751065

52 67 0.00299096717248193

52 68 0.00469430992374209

52 69 0.00507964052423282

52 70 0.00433156729532858

52 71 0.00370515349256118

52 72 0.0164593627418190

52 73 0.0168514688524231

52 74 0.0130353851644101

52 75 0.0182245683434981

52 76 0.0261547063127647

53 1 0.00388049212548935

53 2 0.00438897437797947

53 3 0.00295819234601358

53 4 0.00269666049786377

53 5 0.00302154304776514

53 6 0.00192623714602336

53 7 0.00213215130056077

53 8 0.00869959147426203

53 9 0.00670511869899904

53 10 0.00254421463631060

53 11 0.00306588763991944

53 12 0.00383520472476784

53 13 0.00407807555815198

53 14 0.00398897436745904

53 15 0.00273774925682257

53 16 0.00339478875571508

53 17 0.00235708770842735

53 18 0.00334345101285993

53 19 0.00590805519489357

53 20 0.0138981941365055

53 21 0.0139187911405831

53 22 0.0189412159824838

53 23 0.0246119496942285

53 24 0.0202581157875714

53 25 0.0130555663537497

53 26 0.0158486618695317

53 27 0.0232835805952039

53 28 0.0161234303901571

53 29 0.00291320452640531

53 30 0.00266079403925579

53 31 0.00686895475468252

53 32 0.00273299214406153

53 33 0.00292899687077131

53 34 0.00254146743268624

53 35 0.00256424797475785

53 36 0.00359840482449625

53 37 0.00858638429220848

53 38 0.00895643180611083

53 39 0.0197906704582431

53 40 0.0111355233134762

53 41 0.00662090139149152

53 42 0.00412516517215633

53 43 0.00314023530749274

53 44 0.00751667666254963

53 45 0.0113872361167588

53 46 0.00856354666113912

53 47 0.00627445340507427

53 48 0.00356225308157865

53 49 0.00798751262321806

53 50 0.00865926664257555

53 51 0.00532417315474820

53 52 0.0121839151093535

53 53 0

53 54 0.00499318850882413

53 55 0.0144917897929295

53 56 0.0155681808468700

53 57 0.0161293244403256

53 58 0.0122826098751505

53 59 0.0102353251839968

53 60 0.00496257944053191

53 61 0.00385824194188245

53 62 0.00296349377238347

53 63 0.00331426690874470

53 64 0.00365577645928905

53 65 0.00287865897977191

53 66 0.00240098615390139

53 67 0.00268945840990997

53 68 0.00492972694062588

53 69 0.00525048927363825

53 70 0.00397553850608978

53 71 0.00345480557926026

53 72 0.0238565889511384

53 73 0.0208220635910150

53 74 0.0162940134976952

53 75 0.0198175878799081

53 76 0.0297931339525359

54 1 0.00397180297775357

54 2 0.00474823975951977

54 3 0.00299885272345124

54 4 0.00257766674630378

54 5 0.00284070361419575

54 6 0.00167398886263936

54 7 0.00203088002912089

54 8 0.00849394909358070

54 9 0.00667388555421766

54 10 0.00256761656052862

54 11 0.00292799973899971

54 12 0.00367387031254962

54 13 0.00418972209583890

54 14 0.00457560779130861

54 15 0.00266726767941772

54 16 0.00326555475553958

54 17 0.00210847178779883

54 18 0.00300534613173320

54 19 0.00524138933936524

54 20 0.0117094525687196

54 21 0.0118398311648493

54 22 0.0167592827583147

54 23 0.0206383488355899

54 24 0.0180063040045981

54 25 0.0115660839375096

54 26 0.0153339965513117

54 27 0.0219215524375818

54 28 0.0152014144073475

54 29 0.00285029637316070

54 30 0.00258821817649824

54 31 0.00681052996818354

54 32 0.00262593400312605

54 33 0.00277910400586556

54 34 0.00235413372683713

54 35 0.00242147616650623

54 36 0.00373220293975718

54 37 0.00913668919733501

54 38 0.00936199404217220

54 39 0.0192748271764371

54 40 0.0110308802138213

54 41 0.00688555168137883

54 42 0.00390756269955794

54 43 0.00310275563880702

54 44 0.00818539990422695

54 45 0.0130692301017601

54 46 0.00919757803958032

54 47 0.00629218107770702

54 48 0.00358844023756721

54 49 0.00792873159945629

54 50 0.00824897951435477

54 51 0.00579357319679041

54 52 0.0113682317635750

54 53 0.00293644147773220

54 54 0

54 55 0.0118961370661077

54 56 0.0152402144167227

54 57 0.0157953255260050

54 58 0.0123326121438152

54 59 0.0108469017180317

54 60 0.00501850758982037

54 61 0.00376527056122566

54 62 0.00285463864863134

54 63 0.00330900474351870

54 64 0.00369741243268718

54 65 0.00275544704518427

54 66 0.00228133464811520

54 67 0.00256065758598267

54 68 0.00485185986093017

54 69 0.00522280652010565

54 70 0.00391159428534560

54 71 0.00335540331520390

54 72 0.0231324574567382

54 73 0.0197260672250954

54 74 0.0160538527576406

54 75 0.0193792470288101

54 76 0.0297421032013967

55 1 0.000942772104557621

55 2 0.00151292288509408

55 3 0.000118525740273823

55 4 0

55 5 0

55 6 0

55 7 0

55 8 0.00579714158673472

55 9 0.00386962094951493

55 10 0

55 11 0.000129056776248637

55 12 0.000889175219090488

55 13 0.000953914468193040

55 14 0.00114960542289522

55 15 0

55 16 0.000456425269377725

55 17 0

55 18 0.000165000666101589

55 19 0.00210962216479937

55 20 0.00607051704538475

55 21 0.00606754291994582

55 22 0.00619506171506123

55 23 0.0125140574867775

55 24 0.00945839020334083

55 25 0.00734872786233776

55 26 0.0109283994422053

55 27 0.0152583204300386

55 28 0.0109643046146201

55 29 0.000180084820887827

55 30 0

55 31 0.00405840197692486

55 32 0

55 33 0

55 34 0

55 35 0

55 36 0.000932278681018972

55 37 0.00515661445278792

55 38 0.00510605155215016

55 39 0.0113878752965209

55 40 0.00677462708728038

55 41 0.00523761197810924

55 42 0.000760022005242389

55 43 0.000310258033826338

55 44 0.00516263776287873

55 45 0.00967882801428022

55 46 0.00568697208251323

55 47 0.00320298035698852

55 48 0.000784466083868951

55 49 0.00426528887542910

55 50 0.00500247985587521

55 51 0.00451116261023055

55 52 0.0100341735056191

55 53 0.00355303496775239

55 54 0.00309096302751821

55 55 0

55 56 0.0109347526323867

55 57 0.0105734913959371

55 58 0.00961996783241981

55 59 0.00866320860894643

55 60 0.00259827342696739

55 61 0.000954946134764212

55 62 0.000113831790412355

55 63 0.000512513253502878

55 64 0.000409956691982627

55 65 0

55 66 0

55 67 3.94961160221641e-06

55 68 0.00239213454626464

55 69 0.00240360336583079

55 70 0.00126516742255833

55 71 0.000598113040489290

55 72 0.0163161475495013

55 73 0.0174887566803960

55 74 0.0108094489804388

55 75 0.0159475137636433

55 76 0.0230541992857154

56 1 0

56 2 0

56 3 0

56 4 0

56 5 0

56 6 0

56 7 0

56 8 0.00420233915134649

56 9 0.00245198321252105

56 10 0

56 11 0

56 12 0

56 13 0

56 14 0

56 15 0

56 16 0

56 17 0

56 18 0

56 19 0

56 20 0

56 21 0

56 22 0

56 23 0.000496040510218343

56 24 0

56 25 0.000452178845735785

56 26 0.00242823147586224

56 27 0.00304679929281093

56 28 0.00261170570211511

56 29 0

56 30 0

56 31 0.000578062064976681

56 32 0

56 33 0

56 34 0

56 35 0

56 36 0

56 37 0.00111714811784375

56 38 0.00116614950558963

56 39 0.00101652579994571

56 40 0.00174111296430834

56 41 0.00151627387021036

56 42 0

56 43 0

56 44 0.00179644658341671

56 45 0.00366090539256458

56 46 0.00202463137692732

56 47 0.00146380466418938

56 48 0

56 49 0.00121871879953994

56 50 0.000695374308694041

56 51 5.69195791992883e-05

56 52 0.000703557866776117

56 53 0

56 54 0

56 55 0

56 56 0

56 57 0

56 58 0.000790816847768161

56 59 0.00278072027172338

56 60 0.000452436219560815

56 61 0

56 62 0

56 63 0

56 64 0

56 65 0

56 66 0

56 67 0

56 68 0

56 69 0

56 70 0

56 71 0

56 72 0.00212963062096505

56 73 0.00311697210790585

56 74 0.00387206467634238

56 75 0.0128972527955371

56 76 0.0155650876626214

57 1 0

57 2 0

57 3 0

57 4 0

57 5 0

57 6 0

57 7 0

57 8 0.00410166208705265

57 9 0.00233411081645585

57 10 0

57 11 0

57 12 0

57 13 0

57 14 0

57 15 0

57 16 0

57 17 0

57 18 0

57 19 0

57 20 0

57 21 0

57 22 0.000715599256694510

57 23 0.00268830684085997

57 24 0.00179349526961425

57 25 0.00164858587742001

57 26 0.00431884701950214

57 27 0.00596376285188072

57 28 0.00444742724594738

57 29 0

57 30 0

57 31 0.00136177187818876

57 32 0

57 33 0

57 34 0

57 35 0

57 36 0

57 37 0.00231443634028156

57 38 0.00227692749580471

57 39 0.00253717582464552

57 40 0.00252285510350925

57 41 0.00248764539254465

57 42 0

57 43 0

57 44 0.00289662499758769

57 45 0.00612703339139364

57 46 0.00302041640249295

57 47 0.00150893532363594

57 48 0

57 49 0.00130740536996643

57 50 0.000956828604527837

57 51 0.00114759700529277

57 52 0.00321077885852328

57 53 0.000399239238020543

57 54 0.000561982123083116

57 55 0

57 56 0.00287841251788534

57 57 0

57 58 0.00235799981323215

57 59 0.00441883167044144

57 60 0.000601512120319558

57 61 0

57 62 0

57 63 0

57 64 0

57 65 0

57 66 0

57 67 0

57 68 0.000142468448451227

57 69 8.09897676011806e-05

57 70 0

57 71 0

57 72 0.00543954519073431

57 73 0.00626250883801915

57 74 0.00565129754140803

57 75 0.0128445981964536

57 76 0.0164304638874317

58 1 0

58 2 0.000196275868060480

58 3 0

58 4 0

58 5 0

58 6 0

58 7 0

58 8 0.00323010631129483

58 9 0.00160115572476216

58 10 0

58 11 0

58 12 0

58 13 0

58 14 0

58 15 0

58 16 0

58 17 0

58 18 0

58 19 0

58 20 0

58 21 0

58 22 0.00296388642162615

58 23 0.00319053417918047

58 24 0.00341861958345036

58 25 0.00124059602883309

58 26 0.00443845196767667

58 27 0.00715962826090855

58 28 0.00464162605124852

58 29 0

58 30 0

58 31 0.00133951428251122

58 32 0

58 33 0

58 34 0

58 35 0

58 36 0

58 37 0.00359010424970041

58 38 0.00371429599889517

58 39 0.00357097753932945

58 40 0.00168348704862042

58 41 0.00184417176083163

58 42 0

58 43 0

58 44 0.00360954181099449

58 45 0.00874083985832708

58 46 0.00394109586895919

58 47 0.00122779268853446

58 48 0

58 49 0.000487624747192328

58 50 0

58 51 0.000364029576847780

58 52 0.00321865830715795

58 53 0.000107124689349813

58 54 0.000265475998162179

58 55 0.000149567900831427

58 56 0.00356017905768802

58 57 0.00291456702926041

58 58 0

58 59 0.00468368797811258

58 60 0

58 61 0

58 62 0

58 63 0

58 64 0

58 65 0

58 66 0

58 67 0

58 68 0

58 69 0

58 70 0

58 71 0

58 72 0.00562563519035098

58 73 0.00473999188757479

58 74 0.00540268627599649

58 75 0.0121373466235231

58 76 0.0176900036192071

59 1 0

59 2 0

59 3 0

59 4 0

59 5 0

59 6 0

59 7 0

59 8 0.00423272999119217

59 9 0.00263400787823387

59 10 0

59 11 0

59 12 0

59 13 0

59 14 0

59 15 0

59 16 0

59 17 0

59 18 0

59 19 0

59 20 0

59 21 0

59 22 0

59 23 0

59 24 0

59 25 0

59 26 0.000119837733130290

59 27 0.000227260897152526

59 28 0.000192200245313967

59 29 0

59 30 0

59 31 0

59 32 0

59 33 0

59 34 0

59 35 0

59 36 0

59 37 0.00132984269652553

59 38 0.00157055986323046

59 39 0.000462411668846308

59 40 0.000922475716937599

59 41 0

59 42 0

59 43 0

59 44 0.00134977055075103

59 45 0.00277357885466645

59 46 0.00206337021956937

59 47 0.00179653723683926

59 48 0

59 49 0.00119270801455473

59 50 0

59 51 0

59 52 0

59 53 0

59 54 0

59 55 0

59 56 0

59 57 0

59 58 0.000482037123816670

59 59 0.000533765399236552

59 60 0.000505054938294225

59 61 0

59 62 0

59 63 0

59 64 0

59 65 0

59 66 0

59 67 0

59 68 0

59 69 0

59 70 0

59 71 0

59 72 0

59 73 0

59 74 0.00251962404922934

59 75 0.0127076144316991

59 76 0.0158368264247375

60 1 0

60 2 0

60 3 0

60 4 0

60 5 0

60 6 0

60 7 0

60 8 0.00380462243477064

60 9 0.00210972860993353

60 10 0

60 11 0

60 12 0

60 13 0

60 14 0

60 15 0

60 16 0

60 17 0

60 18 0

60 19 0

60 20 0

60 21 0

60 22 0

60 23 0

60 24 0

60 25 0

60 26 0

60 27 0

60 28 0

60 29 0

60 30 0

60 31 0

60 32 0

60 33 0

60 34 0

60 35 0

60 36 0

60 37 0

60 38 0

60 39 0

60 40 0

60 41 0

60 42 0

60 43 0

60 44 0

60 45 0

60 46 0.000379941086052993

60 47 0.00102043451412470

60 48 0

60 49 0.000119807211305001

60 50 0

60 51 0

60 52 0

60 53 0

60 54 0

60 55 0

60 56 0

60 57 0

60 58 0

60 59 0.000356578135562002

60 60 0.000474552826635555

60 61 0

60 62 0

60 63 0

60 64 0

60 65 0

60 66 0

60 67 0

60 68 0

60 69 0

60 70 0

60 71 0

60 72 0

60 73 0

60 74 0.000785542344009182

60 75 0.0119082813229014

60 76 0.0138178686003423

61 1 0

61 2 0

61 3 0

61 4 0

61 5 0

61 6 0

61 7 0

61 8 0.00359216439559773

61 9 0.00191405940148004

61 10 0

61 11 0

61 12 0

61 13 0

61 14 0

61 15 0

61 16 0

61 17 0

61 18 0

61 19 0

61 20 0

61 21 0

61 22 0

61 23 0

61 24 0

61 25 0

61 26 0

61 27 0

61 28 0

61 29 0

61 30 0

61 31 0

61 32 0

61 33 0

61 34 0

61 35 0

61 36 0

61 37 0

61 38 0

61 39 0

61 40 0

61 41 0

61 42 0

61 43 0

61 44 0

61 45 0

61 46 0

61 47 0.000599809054842138

61 48 0

61 49 0

61 50 0

61 51 0

61 52 0

61 53 0

61 54 0

61 55 0

61 56 0

61 57 0

61 58 0

61 59 0

61 60 0

61 61 0.000189040776159721

61 62 0

61 63 0

61 64 0

61 65 0

61 66 0

61 67 0

61 68 0

61 69 0

61 70 0

61 71 0

61 72 0

61 73 0

61 74 0

61 75 0.0113238075082123

61 76 0.0131603733446832

62 1 0

62 2 0

62 3 0

62 4 0

62 5 0

62 6 0

62 7 0

62 8 0.00451617019229911

62 9 0.00281738435892320

62 10 0

62 11 0

62 12 0

62 13 0

62 14 0

62 15 0

62 16 0

62 17 0

62 18 0

62 19 0

62 20 0

62 21 0

62 22 0

62 23 0

62 24 0

62 25 0

62 26 0

62 27 0

62 28 0

62 29 0

62 30 0

62 31 0

62 32 0

62 33 0

62 34 0

62 35 0

62 36 0

62 37 0

62 38 0

62 39 0

62 40 0.000570395822777403

62 41 0

62 42 0

62 43 0

62 44 0

62 45 0

62 46 0.000631675479708604

62 47 0.00147131375435539

62 48 0

62 49 0.000547593691854975

62 50 0

62 51 0

62 52 0

62 53 0

62 54 0

62 55 0

62 56 0

62 57 0

62 58 0

62 59 0.000365847352217119

62 60 0.000339465812314255

62 61 0.000203268591844941

62 62 0

62 63 0

62 64 0

62 65 0

62 66 0

62 67 0

62 68 0

62 69 0

62 70 0

62 71 0

62 72 0

62 73 0

62 74 0

62 75 0.0118116290417786

62 76 0.0133381261251913

63 1 0.00322797520446327

63 2 0.00263449275779870

63 3 0.00310193581132856

63 4 0.00307778790887514

63 5 0.00320571195296537

63 6 0.00180610300826611

63 7 0.00226163234098420

63 8 0.00843748707053438

63 9 0.00670230978225894

63 10 0.00250766039639350

63 11 0.00315983007252341

63 12 0.00385025998010935

63 13 0.00373337826180664

63 14 0.00169066142377183

63 15 0.00273191279802065

63 16 0.00311162697802203

63 17 0.00238916573015036

63 18 0.00287438410452529

63 19 0.00304186966929298

63 20 0.00203519153974274

63 21 0.00188280134140340

63 22 0.00110235023402971

63 23 0.00145617576345169

63 24 0.00139799343318392

63 25 0.00239646748689759

63 26 0.00274420515080143

63 27 0.00179238867320508

63 28 0.00295110247811109

63 29 0.00292851923988691

63 30 0.00267333804159686

63 31 0.00316492452799411

63 32 0.00292192162493732

63 33 0.00214356336589527

63 34 0.00171809999726447

63 35 0.00238578262449807

63 36 0.00335103667851677

63 37 0.00335353061144955

63 38 0.00339608278226411

63 39 0.00228000048811583

63 40 0.00499837754214083

63 41 0.00389338337807910

63 42 0.00239114277559027

63 43 0.00309585912696675

63 44 0.00336931763389081

63 45 0.00334052921373551

63 46 0.00467409695189769

63 47 0.00547199027955847

63 48 0.00345778359654225

63 49 0.00445278565838836

63 50 0.00360167860359395

63 51 0.00229647047164849

63 52 0.00212317549630270

63 53 0.00170600609424842

63 54 0.00214199983836005

63 55 0.00196927104196931

63 56 0.00341646721635069

63 57 0.00303395942518114

63 58 0.00363415208670836

63 59 0.00428807732089020

63 60 0.00420282607200606

63 61 0.00397635869875723

63 62 0.00311131912353013

63 63 0

63 64 0.000397684036634316

63 65 0.00254798232152070

63 66 0.00215339701590966

63 67 0.00234605137377997

63 68 0.00252287544288443

63 69 0.00276285529763343

63 70 0.00342082681019729

63 71 0.00331839983726956

63 72 0.000861924926766733

63 73 0.00119104842327378

63 74 0.00381632005110177

63 75 0.0161482247950511

63 76 0.0191164931198073

64 1 0.00604917574118025

64 2 0.00567625553988205

64 3 0.00659805268368618

64 4 0.00701912631014723

64 5 0.00643265488711253

64 6 0.00494621323648059

64 7 0.00516766520979561

64 8 0.0113422958257194

64 9 0.00959165323140820

64 10 0.00540890753127721

64 11 0.00614759534387521

64 12 0.00690778574423323

64 13 0.00705552824430522

64 14 0.00522141246151520

64 15 0.00559895360357832

64 16 0.00601723076444727

64 17 0.00553932739035656

64 18 0.00581588864848315

64 19 0.00590864103443334

64 20 0.00523539878705326

64 21 0.00504482162935227

64 22 0.00441100565003039

64 23 0.00542118326352703

64 24 0.00517251929428264

64 25 0.00549801684127316

64 26 0.00584860424054112

64 27 0.00498260721880706

64 28 0.00601524492203487

64 29 0.00579638249669356

64 30 0.00555848643438595

64 31 0.00603908872260706

64 32 0.00579197254864405

64 33 0.00524405856619292

64 34 0.00482520780571560

64 35 0.00532762545604848

64 36 0.00626703645686622

64 37 0.00636111220432845

64 38 0.00638404743904586

64 39 0.00670898212470084

64 40 0.0105241527314131

64 41 0.00700667498555951

64 42 0.00568845655550088

64 43 0.00623911458978255

64 44 0.00635983811005614

64 45 0.00635192628571235

64 46 0.00773150912792653

64 47 0.00842312097669618

64 48 0.00637754969713866

64 49 0.00756396600245879

64 50 0.00707185802784682

64 51 0.00564822150705902

64 52 0.00600677462840538

64 53 0.00520118092566491

64 54 0.00553685665936476

64 55 0.00513111887077322

64 56 0.00649726989898658

64 57 0.00613456066485585

64 58 0.00660845296368706

64 59 0.00723726368682875

64 60 0.00712512593124237

64 61 0.00692444240065815

64 62 0.00605044779579445

64 63 0.00471796983223549

64 64 0

64 65 0.00541411920648061

64 66 0.00489551040800873

64 67 0.00524051193250541

64 68 0.00547358904935913

64 69 0.00565745913191640

64 70 0.00652729911862948

64 71 0.00642703436112690

64 72 0.00470336758977563

64 73 0.00424650262780046

64 74 0.00663379282596843

64 75 0.0207092319819098

64 76 0.0247382844087274

65 1 0.00232149391558750

65 2 0.00183998651711415

65 3 0.00206794986714642

65 4 0.00191343032208491

65 5 0.00223695851694472

65 6 0.000886918132960157

65 7 0.00152830830121747

65 8 0.00762617294555917

65 9 0.00589555631899508

65 10 0.00168029988983576

65 11 0.00231659232522624

65 12 0.00300903082462184

65 13 0.00264002554084780

65 14 0.000679710318081450

65 15 0.00189461947159131

65 16 0.00225278309711430

65 17 0.00157099457647569

65 18 0.00200488114328640

65 19 0.00228448947162407

65 20 0.00107008717722912

65 21 0.000944552867111814

65 22 0.000186956330607169

65 23 0.000458333959693635

65 24 0.000435075137413921

65 25 0.00164778003700200

65 26 0.00185824211190483

65 27 0.000863252607701037

65 28 0.00213021724195206

65 29 0.00209073598260817

65 30 0.00181647628100906

65 31 0.00227825741234633

65 32 0.00209776235692705

65 33 0.00131538624965921

65 34 0.000807425467672540

65 35 0.00157687883718882

65 36 0.00255968808942519

65 37 0.00249637145635906

65 38 0.00256634001203221

65 39 0.000849803539705873

65 40 0.00376846040244716

65 41 0.00310480786487916

65 42 0.00158029241508761

65 43 0.00223560830925995

65 44 0.00254699985681944

65 45 0.00244721635293987

65 46 0.00372500486979327

65 47 0.00460013339370302

65 48 0.00265117584738928

65 49 0.00364464697873546

65 50 0.00277259767287108

65 51 0.00155100254486662

65 52 0.00108658360374625

65 53 0.000612565779248953

65 54 0.000984690931538967

65 55 0.00107568603729624

65 56 0.00265615135148706

65 57 0.00230407325245180

65 58 0.00278267704798063

65 59 0.00354937687763934

65 60 0.00347073476105797

65 61 0.00318002065093026

65 62 0.00234479911275920

65 63 0.00184585194160447

65 64 0.000583774311816332

65 65 0

65 66 0.000661630345673014

65 67 0.00157906217311832

65 68 0.00176119237030015

65 69 0.00198614278387321

65 70 0.00262970216362574

65 71 0.00253949027152567

65 72 0

65 73 0.000430279476748896

65 74 0.00280892248356268

65 75 0.0152100766797540

65 76 0.0172000036642463

66 1 0.00278084663722689

66 2 0.00230532250882287

66 3 0.00255671408715363

66 4 0.00223554945818716

66 5 0.00262910224581747

66 6 0.00124375901906859

66 7 0.00205823109336234

66 8 0.00805536951910169

66 9 0.00646720378447330

66 10 0.00214504728691090

66 11 0.00277546427854170

66 12 0.00343857978183815

66 13 0.00302770852339307

66 14 0.00108307631655258

66 15 0.00234586381548274

66 16 0.00269984055550265

66 17 0.00197671063948757

66 18 0.00242361154294674

66 19 0.00270732950098085

66 20 0.00155428133732194

66 21 0.00141516739101644

66 22 0.000765787212971714

66 23 0.00112142678534533

66 24 0.00102315230687688

66 25 0.00210806084399573

66 26 0.00232348276142491

66 27 0.00134850544656329

66 28 0.00257890877219269

66 29 0.00252959446175238

66 30 0.00228032752741658

66 31 0.00277097135173587

66 32 0.00254854025336160

66 33 0.00176014242490308

66 34 0.00125350743439945

66 35 0.00204888251247248

66 36 0.00301536967582317

66 37 0.00295152987176783

66 38 0.00303835433136579

66 39 0.00141120731830402

66 40 0.00425875775278994

66 41 0.00355793468466559

66 42 0.00199446734419961

66 43 0.00266096914799452

66 44 0.00300882125105229

66 45 0.00291443734966823

66 46 0.00415188379281395

66 47 0.00502318752240072

66 48 0.00309050614015671

66 49 0.00407573723495680

66 50 0.00323181967221375

66 51 0.00199789679608464

66 52 0.00170205709192861

66 53 0.00126627244919253

66 54 0.00165287545241943

66 55 0.00165828751190689

66 56 0.00320144552959634

66 57 0.00284999796596652

66 58 0.00337505912890423

66 59 0.00407926882605914

66 60 0.00391496553940529

66 61 0.00358450710024694

66 62 0.00277160214723643

66 63 0.00242641949185118

66 64 0.00117462819242464

66 65 0.00163018918693580

66 66 0

66 67 0.00210170788569053

66 68 0.00226964441092936

66 69 0.00251419842651390

66 70 0.00304091358890490

66 71 0.00299062632963820

66 72 0.000746680728036964

66 73 0.00113765143609212

66 74 0.00341627298611968

66 75 0.0161904947263101

66 76 0.0179114814082380

67 1 0

67 2 0

67 3 0

67 4 0

67 5 0

67 6 0

67 7 0

67 8 0.00415009239134356

67 9 0.00240064134881435

67 10 0

67 11 0

67 12 0

67 13 0

67 14 0

67 15 0

67 16 0

67 17 0

67 18 0

67 19 0

67 20 0

67 21 0

67 22 0

67 23 0

67 24 0

67 25 0

67 26 0

67 27 0

67 28 0

67 29 0

67 30 0

67 31 0

67 32 0

67 33 0

67 34 0

67 35 0

67 36 0

67 37 0

67 38 0

67 39 0

67 40 0.000135849597765136

67 41 0

67 42 0

67 43 0

67 44 0

67 45 0

67 46 0.000461501757106131

67 47 0.00127040162770742

67 48 0

67 49 0.000318941417188356

67 50 0

67 51 0

67 52 0

67 53 0

67 54 0

67 55 0

67 56 0

67 57 0

67 58 0

67 59 0.000158588632695800

67 60 0

67 61 0

67 62 0

67 63 0

67 64 0

67 65 0

67 66 0

67 67 0

67 68 0

67 69 0

67 70 0

67 71 0

67 72 0

67 73 0

67 74 0

67 75 0.0119509764258126

67 76 0.0137859119338530

68 1 0

68 2 0

68 3 0

68 4 0

68 5 0

68 6 0

68 7 0

68 8 0.00353251832304435

68 9 0.00173450559693777

68 10 0

68 11 0

68 12 0

68 13 0

68 14 0

68 15 0

68 16 0

68 17 0

68 18 0

68 19 0

68 20 0

68 21 0

68 22 0

68 23 0

68 24 0

68 25 0

68 26 0

68 27 0

68 28 0

68 29 0

68 30 0

68 31 0

68 32 0

68 33 0

68 34 0

68 35 0

68 36 0

68 37 0

68 38 0

68 39 0

68 40 0

68 41 0

68 42 0

68 43 0

68 44 0

68 45 0

68 46 9.53717315860247e-05

68 47 0.000815255192077258

68 48 0

68 49 4.17280542182041e-05

68 50 0

68 51 0

68 52 0

68 53 0

68 54 0

68 55 0

68 56 0

68 57 0

68 58 0

68 59 0

68 60 0

68 61 0

68 62 0

68 63 0

68 64 0

68 65 0

68 66 0

68 67 0

68 68 0

68 69 0

68 70 0

68 71 0

68 72 0

68 73 0

68 74 0

68 75 0.0121442043980413

68 76 0.0145815597920691

69 1 0

69 2 0

69 3 0

69 4 0

69 5 0

69 6 0

69 7 0

69 8 0.00350518894536422

69 9 0.00171770392442672

69 10 0

69 11 0

69 12 0

69 13 0

69 14 0

69 15 0

69 16 0

69 17 0

69 18 0

69 19 0

69 20 0

69 21 0

69 22 0

69 23 0

69 24 0

69 25 0

69 26 0

69 27 0

69 28 0

69 29 0

69 30 0

69 31 0

69 32 0

69 33 0

69 34 0

69 35 0

69 36 0

69 37 0

69 38 0

69 39 0

69 40 0

69 41 0

69 42 0

69 43 0

69 44 0

69 45 0

69 46 0

69 47 0.000599809400400497

69 48 0

69 49 0.000241155309882668

69 50 0

69 51 0

69 52 0

69 53 0

69 54 0

69 55 0

69 56 0

69 57 0

69 58 0

69 59 0

69 60 0

69 61 0

69 62 0

69 63 0

69 64 0

69 65 0

69 66 0

69 67 0

69 68 0

69 69 0

69 70 0

69 71 0

69 72 0

69 73 0

69 74 1.01834626327069e-05

69 75 0.0115932490542736

69 76 0.0139956858933503

70 1 0

70 2 0

70 3 0

70 4 0

70 5 0

70 6 0

70 7 0

70 8 0.00281577225464735

70 9 0.00101947245685941

70 10 0

70 11 0

70 12 0

70 13 0

70 14 0

70 15 0

70 16 0

70 17 0

70 18 0

70 19 0

70 20 0

70 21 0

70 22 0

70 23 0

70 24 0

70 25 0

70 26 0

70 27 0

70 28 0

70 29 0

70 30 0

70 31 0

70 32 0

70 33 0

70 34 0

70 35 0

70 36 0

70 37 0

70 38 0

70 39 0

70 40 0

70 41 0

70 42 0

70 43 0

70 44 0

70 45 0

70 46 0

70 47 0

70 48 0

70 49 0

70 50 0

70 51 0

70 52 0

70 53 0

70 54 0

70 55 0

70 56 0

70 57 0

70 58 0

70 59 0

70 60 0

70 61 0

70 62 0

70 63 0

70 64 0

70 65 0

70 66 0

70 67 0

70 68 0

70 69 0

70 70 0

70 71 0

70 72 0

70 73 0

70 74 0

70 75 0.0103510626521766

70 76 0.0128518775088446

71 1 0

71 2 0

71 3 0

71 4 0

71 5 0

71 6 0

71 7 0

71 8 0.00372918332228733

71 9 0.00195801735450651

71 10 0

71 11 0

71 12 0

71 13 0

71 14 0

71 15 0

71 16 0

71 17 0

71 18 0

71 19 0

71 20 0

71 21 0

71 22 0

71 23 0

71 24 0

71 25 0

71 26 0

71 27 0

71 28 0

71 29 0

71 30 0

71 31 0

71 32 0

71 33 0

71 34 0

71 35 0

71 36 0

71 37 0

71 38 0

71 39 0

71 40 0.000101963114094716

71 41 0

71 42 0

71 43 0

71 44 0

71 45 0

71 46 0

71 47 0.000662153807768906

71 48 0

71 49 0

71 50 0

71 51 0

71 52 0

71 53 0

71 54 0

71 55 0

71 56 0

71 57 0

71 58 0

71 59 0

71 60 0

71 61 0

71 62 0

71 63 0

71 64 0

71 65 0

71 66 0

71 67 0

71 68 0

71 69 0

71 70 0

71 71 0

71 72 0

71 73 0

71 74 0

71 75 0.0117548210599019

71 76 0.0136280897541732

72 1 0

72 2 0

72 3 0

72 4 0

72 5 0

72 6 0

72 7 0

72 8 0.00154973919503687

72 9 0

72 10 0

72 11 0

72 12 0

72 13 0

72 14 0

72 15 0

72 16 0

72 17 0

72 18 0

72 19 0

72 20 0.00838730847512781

72 21 0.00916068994492614

72 22 0.0245141965443445

72 23 0.0213664778455505

72 24 0.0219377873485049

72 25 0.00801993961531000

72 26 0.0110991707874087

72 27 0.0214459100446747

72 28 0.0110143864859555

72 29 0

72 30 0

72 31 0.00169109707525117

72 32 0

72 33 0

72 34 0

72 35 0

72 36 0

72 37 0.00400470068699099

72 38 0.00467998356290389

72 39 0.0135090541980059

72 40 8.78056000348515e-05

72 41 0

72 42 0

72 43 0

72 44 0.00118057170235764

72 45 0.00610437568429323

72 46 0.00287259452700495

72 47 1.01660948698123e-05

72 48 0

72 49 0.000892909442975265

72 50 0.000489958301529647

72 51 0

72 52 0.0143463718952455

72 53 0.0232722645264758

72 54 0.0208562654918314

72 55 0.0202565677656366

72 56 0.00911961312017628

72 57 0.0109936773323779

72 58 0.00695516709466215

72 59 0.00249548464650684

72 60 0

72 61 0

72 62 0

72 63 0

72 64 0

72 65 0

72 66 0

72 67 0

72 68 0

72 69 0

72 70 0

72 71 0

72 72 0

72 73 0.0121778415197371

72 74 0.0111681009397304

72 75 0.0189406187308530

72 76 0.0325100797311391

73 1 0.000674415349993462

73 2 0.00161511593930819

73 3 0

73 4 0

73 5 0

73 6 0

73 7 0

73 8 0.00284100568699275

73 9 0.00101031695938891

73 10 0

73 11 0

73 12 0

73 13 0

73 14 0

73 15 0

73 16 0.000316226150599119

73 17 0

73 18 0

73 19 0.000666085808482930

73 20 0.00132874335361810

73 21 0.00207143661825970

73 22 0.0172487498541327

73 23 0.0110367290727658

73 24 0.0144732638690639

73 25 0.00526320923808610

73 26 0.00824261332521159

73 27 0.0152131066406244

73 28 0.00828469555933908

73 29 0

73 30 0

73 31 0.00304044941711401

73 32 0

73 33 0

73 34 0

73 35 0

73 36 0

73 37 0.00359564127763662

73 38 0.00440611054309997

73 39 0.0116291885974366

73 40 0.00142180653189583

73 41 0

73 42 0

73 43 0

73 44 0.00590311690707768

73 45 0.00611633234187614

73 46 0.00252761873458018

73 47 0.000999196185325735

73 48 0.00103533638563202

73 49 0.00553105439567925

73 50 0.00271481837878484

73 51 0

73 52 0.00942004406846786

73 53 0.0167557057271581

73 54 0.0142952872208526

73 55 0.0130483078855556

73 56 0.00593349673468757

73 57 0.00637710550532944

73 58 0.00272309926202086

73 59 0.00219284739578529

73 60 0

73 61 0

73 62 0

73 63 0

73 64 0

73 65 0

73 66 0

73 67 0

73 68 0

73 69 0

73 70 0

73 71 0

73 72 0.0129106718445632

73 73 0

73 74 0.0104683350061193

73 75 0.0187813243835695

73 76 0.0296969074122861

74 1 0

74 2 0

74 3 0

74 4 0

74 5 0

74 6 0

74 7 0

74 8 0.00221998115080435

74 9 0.000699525521283051

74 10 0

74 11 0

74 12 0

74 13 0

74 14 0

74 15 0

74 16 0

74 17 0

74 18 0

74 19 0

74 20 0

74 21 0

74 22 0

74 23 0

74 24 0

74 25 0

74 26 0

74 27 0.00128969293469994

74 28 0

74 29 0

74 30 0

74 31 0

74 32 0

74 33 0

74 34 0

74 35 0

74 36 0

74 37 0

74 38 0

74 39 0

74 40 0

74 41 0

74 42 0.00102946981689833

74 43 0

74 44 0.000469009212262117

74 45 0.000210509512959089

74 46 0

74 47 0

74 48 0

74 49 0.000422034756034750

74 50 0.00222707029085412

74 51 0.00150524923343853

74 52 0

74 53 0

74 54 0

74 55 0

74 56 0

74 57 0

74 58 0

74 59 0.000681190474491333

74 60 0.000495415315858949

74 61 0

74 62 0

74 63 0

74 64 0

74 65 0

74 66 0

74 67 0

74 68 0

74 69 0

74 70 0

74 71 0

74 72 0

74 73 0.00142167316858743

74 74 0

74 75 0.0143344961566536

74 76 0.0209531721833336

75 1 0

75 2 0

75 3 0

75 4 0

75 5 0

75 6 0

75 7 0

75 8 0.00117302289814636

75 9 0

75 10 0

75 11 0

75 12 0

75 13 0

75 14 0

75 15 0

75 16 0

75 17 0

75 18 0

75 19 0

75 20 0

75 21 0

75 22 0

75 23 0

75 24 0

75 25 0

75 26 0

75 27 0

75 28 0

75 29 0

75 30 0

75 31 0

75 32 0

75 33 0

75 34 0

75 35 0

75 36 0

75 37 0

75 38 0

75 39 0

75 40 0

75 41 0

75 42 0

75 43 0

75 44 0

75 45 0

75 46 0

75 47 0

75 48 0

75 49 0

75 50 0

75 51 0

75 52 0

75 53 0

75 54 0

75 55 0

75 56 0

75 57 0

75 58 0

75 59 0

75 60 0

75 61 0

75 62 0

75 63 0

75 64 0

75 65 0

75 66 0

75 67 0

75 68 0

75 69 0

75 70 0

75 71 0

75 72 0

75 73 0

75 74 0.000531674950098759

75 75 0.0118771360816483

75 76 0.0132236016562384

76 1 0

76 2 0

76 3 0

76 4 0

76 5 0

76 6 0

76 7 0

76 8 0

76 9 0

76 10 0

76 11 0

76 12 0

76 13 0

76 14 0

76 15 0

76 16 0

76 17 0

76 18 0

76 19 0

76 20 0

76 21 0

76 22 0.00465623706237128

76 23 0.00199236174580952

76 24 0.00356318833468117

76 25 0

76 26 0.00127500096809841

76 27 0.00525495243326080

76 28 4.75645120794344e-05

76 29 0

76 30 0

76 31 0

76 32 0

76 33 0

76 34 0

76 35 0

76 36 0

76 37 0

76 38 0

76 39 0

76 40 0

76 41 0

76 42 0

76 43 0

76 44 0

76 45 0

76 46 0

76 47 0

76 48 0

76 49 0

76 50 0

76 51 0

76 52 0.00236163283436319

76 53 0.00664818337993411

76 54 0.00695327204585472

76 55 0.00261798689397019

76 56 0

76 57 0

76 58 0.000651774757050028

76 59 0

76 60 0

76 61 0

76 62 0

76 63 0

76 64 0

76 65 0

76 66 0

76 67 0

76 68 0

76 69 0

76 70 0

76 71 0

76 72 0.0119058606168243

76 73 0.00618849312123881

76 74 0.000793845531229964

76 75 0.0143030792453120

76 76 0.0133402829232523

**Data for Figure 6**

First column is the residue index, second column is the net entropy transfer from the corresponding residue

1 -1.635758E-02

2 1.228610E-01

3 2.915981E-01

4 3.828480E-01

5 8.043786E-02

6 2.626340E-01

7 -7.661046E-03

8 -3.547890E-01

9 -2.166658E-01

10 -1.407498E-02

11 6.234863E-02

12 -1.936423E-02

13 2.972078E-01

14 1.959931E-03

15 1.820618E-03

16 -2.386536E-02

17 -1.388468E-02

18 -2.037728E-02

19 -3.736372E-02

20 -3.217397E-02

21 -3.096435E-02

22 2.210194E-01

23 2.958753E-01

24 2.527936E-01

25 -4.445705E-02

26 -9.565479E-02

27 8.338998E-02

28 -1.052748E-01

29 -2.107753E-02

30 -1.621835E-02

31 -5.856135E-02

32 -2.036949E-02

33 -1.084693E-02

34 -2.390692E-03

35 -1.116367E-02

36 -2.901689E-02

37 -6.886830E-02

38 -6.877866E-02

39 -3.332093E-02

40 -4.632157E-02

41 -8.289941E-02

42 -1.087303E-02

43 -3.045048E-02

44 -6.403597E-02

45 -8.880086E-02

46 -1.288770E-01

47 -1.412868E-01

48 1.411277E-03

49 -7.139419E-02

50 -5.557634E-02

51 1.762653E-01

52 3.514414E-01

53 4.867972E-01

54 4.581209E-01

55 1.882254E-01

56 -1.019386E-01

57 -4.778506E-02

58 -4.194484E-02

59 -1.244472E-01

60 -7.047095E-02

61 -4.852518E-02

62 -1.902509E-02

63 1.962002E-01

64 4.510747E-01

65 1.357314E-01

66 1.841600E-01

67 -9.449440E-03

68 -3.076391E-02

69 -3.593860E-02

70 -4.476139E-02

71 -3.360720E-02

72 1.338524E-01

73 2.674402E-02

74 -1.806233E-01

75 -1.025620

76 -1.237860

**Data for Fig. 8**

First column is time in ns. Second column is autocorrelation for residue 7, third column is for autocorrelation for residue 71

0.1 0.86416 0.88133

0.2 0.64081 0.65982

0.3 0.60269 0.63016

0.4 0.57739 0.60952

0.5 0.55802 0.59594

0.6 0.54172 0.58493

0.7 0.5286 0.57419

0.8 0.5174 0.56431

0.9 0.5078 0.55485

1 0.49845 0.54674

1.1 0.48893 0.5397

1.2 0.48159 0.53421

1.3 0.47299 0.52873

1.4 0.46445 0.52291

1.5 0.45865 0.51717

1.6 0.45352 0.5135

1.7 0.44687 0.5092

1.8 0.44085 0.50446

1.9 0.4375 0.49864

2 0.43242 0.4957

2.1 0.42512 0.49159

2.2 0.42041 0.48883

2.3 0.41379 0.48349

2.4 0.40923 0.48058

2.5 0.40538 0.47825

2.6 0.40297 0.4745

2.7 0.39869 0.47071

2.8 0.39503 0.46842

2.9 0.39123 0.46845

3 0.38682 0.46777

3.1 0.38197 0.46535

3.2 0.37948 0.46426

3.3 0.37733 0.46364

3.4 0.37589 0.46143

3.5 0.37254 0.46028

3.6 0.36858 0.45679

3.7 0.36435 0.45393

3.8 0.36083 0.4498

3.9 0.35834 0.44656

4 0.35707 0.44535

4.1 0.3549 0.44403

4.2 0.35565 0.44376

4.3 0.35466 0.4425

4.4 0.35412 0.44268

4.5 0.35323 0.44346

4.6 0.35406 0.44309

4.7 0.35262 0.44399

4.8 0.3496 0.44423

4.9 0.34896 0.44412

5 0.34584 0.44261

5.1 0.34313 0.44275

5.2 0.34203 0.44402

5.3 0.33802 0.44254

5.4 0.33263 0.44

5.5 0.32725 0.43933

5.6 0.32347 0.4384

5.7 0.31955 0.43714

5.8 0.31514 0.43659

5.9 0.31041 0.43517

6 0.30633 0.43479

6.1 0.30478 0.43356

6.2 0.30056 0.43081

6.3 0.29903 0.42903

6.4 0.29977 0.42681

6.5 0.3004 0.42505

6.6 0.3004 0.42297

6.7 0.29914 0.42159

6.8 0.29768 0.4207

6.9 0.29677 0.41931

7 0.29615 0.41752

7.1 0.29536 0.41512

7.2 0.29322 0.41316

7.3 0.2914 0.41249

7.4 0.28933 0.41211

7.5 0.28798 0.41167

7.6 0.2857 0.40844

7.7 0.28389 0.40535

7.8 0.28406 0.40224

7.9 0.28096 0.40013

8 0.27817 0.39959

8.1 0.27711 0.4002

8.2 0.27566 0.3997

8.3 0.27239 0.39857

8.4 0.27018 0.39828

8.5 0.26767 0.39644

8.6 0.26416 0.39351

8.7 0.26245 0.39208

8.8 0.25912 0.3919

8.9 0.25672 0.39078

9 0.25524 0.38885

9.1 0.25476 0.38534

9.2 0.25388 0.38409

9.3 0.25277 0.38382

9.4 0.25347 0.38382

9.5 0.25253 0.38404

9.6 0.25085 0.38325

9.7 0.24982 0.38193

9.8 0.25016 0.37947

9.9 0.24924 0.37732

10 0.2482 0.37756

10.1 0.24621 0.37706

10.2 0.24791 0.37715

10.3 0.24709 0.37779

10.4 0.24532 0.37908

10.5 0.24479 0.3773

10.6 0.24502 0.37667

10.7 0.24269 0.37576

10.8 0.24069 0.37526

10.9 0.23988 0.37502

11 0.23822 0.37416

11.1 0.23894 0.37185

11.2 0.239 0.37045

11.3 0.23901 0.36892

11.4 0.23952 0.36922

11.5 0.23977 0.36898

11.6 0.24003 0.3694

11.7 0.23919 0.37016

11.8 0.23665 0.36862

11.9 0.23517 0.36812

12 0.23381 0.36942

12.1 0.23323 0.36848

12.2 0.2353 0.36925

12.3 0.23519 0.36639

12.4 0.2338 0.36464

12.5 0.22999 0.36424

12.6 0.22838 0.36354

12.7 0.22651 0.36282

12.8 0.22729 0.36221

12.9 0.22843 0.36195

13 0.2277 0.3612

13.1 0.22777 0.36222

13.2 0.22673 0.36223

13.3 0.22523 0.36306

13.4 0.22519 0.36394

13.5 0.22454 0.36299

13.6 0.22496 0.36203

13.7 0.22274 0.36002

13.8 0.22369 0.35949

13.9 0.2223 0.36024

14 0.22071 0.35976

14.1 0.21943 0.35982

14.2 0.21733 0.35988

14.3 0.21715 0.35919

14.4 0.21756 0.35669

14.5 0.21611 0.35425

14.6 0.2171 0.35322

14.7 0.21824 0.35361

14.8 0.21782 0.35303

14.9 0.21644 0.35347

15 0.2146 0.35113

15.1 0.21271 0.3495

15.2 0.20912 0.3487

15.3 0.20724 0.34974

15.4 0.2057 0.34932

15.5 0.20579 0.34791

15.6 0.20528 0.34869

15.7 0.20537 0.34583

15.8 0.20374 0.34438

15.9 0.20244 0.34322

16 0.20042 0.34363

16.1 0.20038 0.34538

16.2 0.19944 0.34648

16.3 0.19803 0.34627

16.4 0.19735 0.34646

16.5 0.19564 0.34638

16.6 0.19511 0.34702

16.7 0.19657 0.34927

16.8 0.197 0.35024

16.9 0.19712 0.35083

17 0.19533 0.35052

17.1 0.19547 0.34787

17.2 0.19322 0.34472

17.3 0.19303 0.34421

17.4 0.1922 0.34343

17.5 0.19079 0.34227

17.6 0.19123 0.3421

17.7 0.18833 0.34009

17.8 0.18645 0.33922

17.9 0.18402 0.33813

18 0.18257 0.33673

18.1 0.18368 0.33521

18.2 0.1828 0.33308

18.3 0.18316 0.33105

18.4 0.18173 0.32918

18.5 0.18005 0.32775

18.6 0.18042 0.32649

18.7 0.17955 0.32589

18.8 0.17833 0.32498

18.9 0.17957 0.32444

19 0.17741 0.32531

19.1 0.17517 0.32576

19.2 0.17533 0.32367

19.3 0.17653 0.3238

19.4 0.17635 0.32236

19.5 0.17717 0.31943

19.6 0.17529 0.31794

19.7 0.17702 0.31704

19.8 0.17834 0.31861

19.9 0.17975 0.3175

20 0.17891 0.31863

20.1 0.17776 0.31818

20.2 0.17853 0.31936

20.3 0.17627 0.31929

20.4 0.17498 0.31761

20.5 0.17311 0.31646

20.6 0.17228 0.31642

20.7 0.16947 0.31743

20.8 0.16591 0.31479

20.9 0.16359 0.31381

21 0.16367 0.31312

21.1 0.16204 0.31415

21.2 0.15986 0.31385

21.3 0.15867 0.31406

21.4 0.16127 0.31311

21.5 0.16279 0.31414

21.6 0.16151 0.31435

21.7 0.16002 0.31234

21.8 0.15968 0.30982

21.9 0.162 0.30979

22 0.16288 0.3094

22.1 0.16064 0.30795

22.2 0.16184 0.309

22.3 0.15806 0.30856

22.4 0.15647 0.3085

22.5 0.15699 0.30747

22.6 0.15761 0.30667

22.7 0.15892 0.30572

22.8 0.1586 0.30541

22.9 0.15763 0.30515

23 0.15794 0.30532

23.1 0.15729 0.30575

23.2 0.15675 0.30501

23.3 0.15565 0.30385

23.4 0.15653 0.30506

23.5 0.1581 0.30346

23.6 0.15774 0.30192

23.7 0.15826 0.29993

23.8 0.15974 0.29848

23.9 0.16272 0.29938

24 0.16575 0.29919

24.1 0.16805 0.30047

24.2 0.16843 0.30183

24.3 0.16798 0.30182

24.4 0.16641 0.30315

24.5 0.16552 0.30237

24.6 0.16572 0.30022

24.7 0.16638 0.30033

24.8 0.16693 0.30128

24.9 0.1666 0.30066

25 0.16476 0.30042

25.1 0.16484 0.29874

25.2 0.16365 0.29617

25.3 0.16444 0.29615

25.4 0.16368 0.29414

25.5 0.16267 0.29338

25.6 0.16013 0.29057

25.7 0.15745 0.28802

25.8 0.15629 0.287

25.9 0.15563 0.28562

26 0.15359 0.28248

26.1 0.15399 0.28092

26.2 0.15196 0.2798

26.3 0.15051 0.27857

26.4 0.1489 0.2774

26.5 0.14931 0.27441

26.6 0.14803 0.27163

26.7 0.14929 0.27235

26.8 0.1494 0.27211

26.9 0.14906 0.27225

27 0.15119 0.27266

27.1 0.15046 0.27089

27.2 0.14751 0.26961

27.3 0.14662 0.26812

27.4 0.14764 0.26806

27.5 0.14643 0.2667

27.6 0.14448 0.26456

27.7 0.14298 0.26202

27.8 0.14476 0.2607

27.9 0.14317 0.25866

28 0.14408 0.25645

28.1 0.14294 0.25645

28.2 0.14303 0.25624

28.3 0.14144 0.25714

28.4 0.14143 0.25581

28.5 0.14183 0.25556

28.6 0.14526 0.25775

28.7 0.14567 0.25797

28.8 0.14746 0.25843

28.9 0.14818 0.25905

29 0.14987 0.25925

29.1 0.14821 0.25861

29.2 0.1475 0.25806

29.3 0.14688 0.25615

29.4 0.14696 0.25521

29.5 0.14687 0.25627

29.6 0.14696 0.25398

29.7 0.14737 0.25314

29.8 0.14852 0.25164

29.9 0.14998 0.25078

30 0.15256 0.25025

30.1 0.1515 0.25105

30.2 0.15083 0.25048

30.3 0.15125 0.24952

30.4 0.15173 0.2493

30.5 0.15053 0.24935

30.6 0.15 0.24937

30.7 0.15028 0.2479

30.8 0.14812 0.24625

30.9 0.14558 0.24591

31 0.14341 0.24622

31.1 0.14203 0.24469

31.2 0.13923 0.24197

31.3 0.13652 0.24058

31.4 0.13484 0.23947

31.5 0.13694 0.23947

31.6 0.13667 0.23914

31.7 0.13262 0.23654

31.8 0.13176 0.23423

31.9 0.1323 0.23397

32 0.13218 0.23267

32.1 0.13365 0.23057

32.2 0.13316 0.22934

32.3 0.13242 0.22893

32.4 0.1301 0.22843

32.5 0.12896 0.22589

32.6 0.12744 0.22416

32.7 0.12609 0.22195

32.8 0.1239 0.22103

32.9 0.12167 0.22051

33 0.12201 0.2207

33.1 0.12012 0.22113

33.2 0.11734 0.22177

33.3 0.11646 0.2212

33.4 0.11516 0.22118

33.5 0.1154 0.22253

33.6 0.11087 0.22002

33.7 0.11041 0.21783

33.8 0.11068 0.21582

33.9 0.10987 0.21427

34 0.10913 0.21525

34.1 0.11104 0.21281

34.2 0.11002 0.21253

34.3 0.10717 0.21053

34.4 0.10702 0.20959

34.5 0.10532 0.20935

34.6 0.10511 0.21072

34.7 0.10362 0.2118

34.8 0.10194 0.21081

34.9 0.10305 0.21105

35 0.10234 0.2111

35.1 0.10134 0.21014

35.2 0.10088 0.20857

35.3 0.09936 0.20972

35.4 0.0998 0.2101

35.5 0.09907 0.2098

35.6 0.09781 0.20849

35.7 0.09648 0.20721

35.8 0.0959 0.20648

35.9 0.09503 0.20537

36 0.09766 0.20428

36.1 0.09822 0.20265

36.2 0.09813 0.20246

36.3 0.09823 0.20212

36.4 0.09691 0.20089

36.5 0.0972 0.20024

36.6 0.09771 0.20176

36.7 0.09624 0.20166

36.8 0.09585 0.20139

36.9 0.09531 0.20145

37 0.0966 0.20254

37.1 0.09656 0.2011

37.2 0.09787 0.20047

37.3 0.09799 0.1979

37.4 0.09822 0.19612

37.5 0.09963 0.19579

37.6 0.09916 0.19542

37.7 0.09779 0.19369

37.8 0.09549 0.19367

37.9 0.0965 0.19086

38 0.09381 0.18922

38.1 0.09272 0.18998

38.2 0.09217 0.19195

38.3 0.09267 0.19302

38.4 0.09398 0.19208

38.5 0.09578 0.19185

38.6 0.0976 0.19279

38.7 0.09848 0.19147

38.8 0.09909 0.18946

38.9 0.09914 0.18975

39 0.10015 0.18955

39.1 0.10177 0.1897

39.2 0.10238 0.18972

39.3 0.10308 0.18911

39.4 0.10347 0.18852

39.5 0.10329 0.18956

39.6 0.10334 0.18826

39.7 0.10498 0.18749

39.8 0.10477 0.18712

39.9 0.1045 0.18705

40 0.10472 0.18727

40.1 0.1025 0.18571

40.2 0.1002 0.1846

40.3 0.10038 0.18522

40.4 0.0984 0.18453

40.5 0.09616 0.18305

40.6 0.09272 0.17995

40.7 0.08949 0.17498

40.8 0.08793 0.17171

40.9 0.08591 0.16944

41 0.08288 0.16886

41.1 0.08232 0.16877

41.2 0.08091 0.1665

41.3 0.0814 0.16644

41.4 0.08149 0.16564

41.5 0.08247 0.16488

41.6 0.08399 0.16631

41.7 0.08222 0.16708

41.8 0.0803 0.16681

41.9 0.07753 0.16712

42 0.07405 0.1676

42.1 0.072 0.16669

42.2 0.06855 0.16648

42.3 0.06753 0.16604

42.4 0.06597 0.1659

42.5 0.06656 0.16553

42.6 0.063 0.16399

42.7 0.06308 0.1638

42.8 0.06365 0.16305

42.9 0.06234 0.16233

43 0.06387 0.16265

43.1 0.06343 0.16042

43.2 0.0619 0.15907

43.3 0.06236 0.15901

43.4 0.06226 0.15849

43.5 0.06188 0.15897

43.6 0.06146 0.15914

43.7 0.05855 0.16056

43.8 0.05841 0.1593

43.9 0.05825 0.15897

44 0.05856 0.15928

44.1 0.0602 0.15947

44.2 0.06109 0.16087

44.3 0.06255 0.1608

44.4 0.06252 0.16166

44.5 0.06227 0.1611

44.6 0.06232 0.15947

44.7 0.0623 0.15846

44.8 0.06118 0.15824

44.9 0.06281 0.15933

45 0.06318 0.15938

45.1 0.05976 0.16007

45.2 0.05538 0.15963

45.3 0.05329 0.15971

45.4 0.05012 0.15832

45.5 0.04752 0.15794

45.6 0.04505 0.1584

45.7 0.0441 0.15611

45.8 0.04452 0.15419

45.9 0.04551 0.1518

46 0.04357 0.14994

46.1 0.04318 0.14882

46.2 0.04352 0.14704

46.3 0.04308 0.14402

46.4 0.04498 0.14292

46.5 0.04721 0.14203

46.6 0.04828 0.14122

46.7 0.0506 0.13931

46.8 0.05286 0.13839

46.9 0.05379 0.13709

47 0.0557 0.13608

47.1 0.05555 0.13329

47.2 0.05614 0.13318

47.3 0.05527 0.13313

47.4 0.05507 0.13317

47.5 0.05435 0.13241

47.6 0.05443 0.13144

47.7 0.0528 0.13157

47.8 0.05124 0.13044

47.9 0.05125 0.12987

48 0.04995 0.1291

48.1 0.04813 0.12837

48.2 0.04855 0.12851

48.3 0.05006 0.12777

48.4 0.04901 0.12904

48.5 0.04864 0.13031

48.6 0.04949 0.13169

48.7 0.04861 0.13224

48.8 0.04929 0.13225

48.9 0.05033 0.13276

49 0.05142 0.13314

49.1 0.05278 0.13161

49.2 0.05434 0.13273

49.3 0.05489 0.13151

49.4 0.0532 0.13055

49.5 0.05342 0.13009

49.6 0.05135 0.12868

49.7 0.05215 0.13049

49.8 0.05081 0.12916

49.9 0.05032 0.12902

50 0.04951 0.12841

50.1 0.05056 0.12935

50.2 0.04854 0.12815

50.3 0.04663 0.12746

50.4 0.04822 0.12614

50.5 0.04805 0.12353

50.6 0.04775 0.12457

50.7 0.04806 0.12309

50.8 0.05054 0.1218

50.9 0.05032 0.12116

51 0.05155 0.12153

51.1 0.04991 0.12345

51.2 0.04967 0.12291

51.3 0.04986 0.12404

51.4 0.0476 0.12365

51.5 0.04781 0.12347

51.6 0.04889 0.12436

51.7 0.04712 0.12227

51.8 0.04734 0.12227

51.9 0.04663 0.12085

52 0.04768 0.11999

52.1 0.04716 0.12043

52.2 0.0466 0.1194

52.3 0.04305 0.11875

52.4 0.04139 0.12051

52.5 0.04266 0.12035

52.6 0.04288 0.11959

52.7 0.0421 0.11865

52.8 0.04221 0.11879

52.9 0.04074 0.117

53 0.03778 0.11704

53.1 0.03724 0.11737

53.2 0.03722 0.11539

53.3 0.03862 0.11631

53.4 0.03946 0.11621

53.5 0.03881 0.11695

53.6 0.03892 0.1172

53.7 0.03924 0.11831

53.8 0.04064 0.11794

53.9 0.04059 0.11763

54 0.04158 0.11564

54.1 0.0406 0.11484

54.2 0.04086 0.11323

54.3 0.0432 0.11261

54.4 0.04357 0.11178

54.5 0.04189 0.11155

54.6 0.04365 0.11036

54.7 0.04483 0.10939

54.8 0.04429 0.1072

54.9 0.04304 0.10589

55 0.041 0.1035

55.1 0.04156 0.10095

55.2 0.03972 0.09944

55.3 0.04079 0.09813

55.4 0.03979 0.09708

55.5 0.0399 0.09437

55.6 0.04127 0.09396

55.7 0.03928 0.09181

55.8 0.0377 0.09086

55.9 0.03801 0.08945

56 0.03777 0.08811

56.1 0.03845 0.08536

56.2 0.03716 0.08389

56.3 0.03915 0.08424

56.4 0.04023 0.08401

56.5 0.03909 0.08204

56.6 0.03816 0.08017

56.7 0.0373 0.07758

56.8 0.03782 0.07669

56.9 0.03531 0.07621

57 0.03487 0.07511

57.1 0.0345 0.07435

57.2 0.0351 0.07338

57.3 0.03216 0.0698

57.4 0.03037 0.06817

57.5 0.02719 0.06665

57.6 0.02429 0.06102

57.7 0.02347 0.05968

57.8 0.02137 0.05879

57.9 0.01903 0.05886

58 0.01664 0.05789

58.1 0.01579 0.05534

58.2 0.01421 0.05397

58.3 0.01274 0.05303

58.4 0.01124 0.05313

58.5 0.01188 0.05391

58.6 0.01193 0.05322

58.7 0.0126 0.05331

58.8 0.01479 0.0536

58.9 0.0158 0.0529

59 0.01301 0.05006

59.1 0.01256 0.04934

59.2 0.01234 0.04857

59.3 0.01171 0.04912

59.4 0.01035 0.04777

59.5 0.00833 0.04734

59.6 0.00824 0.04842

59.7 0.00605 0.04849

59.8 0.00446 0.04803

59.9 0.00271 0.04765

60 -8.4417E-4 0.04907

60.1 -0.00199 0.0485

60.2 -0.00319 0.04758

60.3 -0.00513 0.04819

60.4 -0.00492 0.04928

60.5 -0.005 0.04826

60.6 -0.00571 0.04751

60.7 -0.00559 0.04625

60.8 -0.00569 0.04496

60.9 -0.00717 0.04186

61 -0.00953 0.03959

61.1 -0.01142 0.03817

61.2 -0.00975 0.03851

61.3 -0.00867 0.03708

61.4 -0.00936 0.03603

61.5 -0.00903 0.03503

61.6 -0.00801 0.03593

61.7 -0.00934 0.0344

61.8 -0.00908 0.03483

61.9 -0.00889 0.03655

62 -0.01089 0.03565

62.1 -0.01287 0.03469

62.2 -0.01321 0.0336

62.3 -0.01584 0.03303

62.4 -0.01578 0.03142

62.5 -0.01606 0.02968

62.6 -0.01662 0.02805

62.7 -0.0165 0.02676

62.8 -0.01754 0.02558

62.9 -0.02047 0.02465

63 -0.02033 0.0223

63.1 -0.01972 0.02184

63.2 -0.02194 0.0205

63.3 -0.02247 0.02057

63.4 -0.02433 0.02043

63.5 -0.02511 0.01819

63.6 -0.02771 0.01605

63.7 -0.02818 0.01379

63.8 -0.02911 0.01372

63.9 -0.02854 0.01238

64 -0.02707 0.01408

64.1 -0.02766 0.01244

64.2 -0.02823 0.01057

64.3 -0.02811 0.01044

64.4 -0.03009 0.00829

64.5 -0.02862 0.00762

64.6 -0.03022 0.00583

64.7 -0.03187 0.00636

64.8 -0.03558 0.00566

64.9 -0.03583 0.00473

65 -0.03722 0.00342

65.1 -0.03679 0.00299

65.2 -0.03462 0.00437

65.3 -0.03442 0.00561

65.4 -0.03549 0.00655

65.5 -0.03449 0.00752

65.6 -0.0332 0.00615

65.7 -0.03194 0.0056

65.8 -0.03149 0.00376

65.9 -0.03307 0.00163

66 -0.03632 9.94259E-4

66.1 -0.03839 -1.50558E-4

66.2 -0.03951 -1.81965E-4

66.3 -0.03782 4.48048E-4

66.4 -0.03632 -0.00125

66.5 -0.03601 -0.00352

66.6 -0.03651 -0.00505

66.7 -0.03712 -0.00565

66.8 -0.03565 -0.00544

66.9 -0.03455 -0.00624

67 -0.03562 -0.00757

67.1 -0.03523 -0.00717

67.2 -0.0348 -0.00707

67.3 -0.03581 -0.00873

67.4 -0.03591 -0.00872

67.5 -0.0363 -0.0083

67.6 -0.03662 -0.00851

67.7 -0.03603 -0.00979

67.8 -0.03515 -0.0093

67.9 -0.03497 -0.00962

68 -0.0323 -0.00968

68.1 -0.03093 -0.00875

68.2 -0.02924 -0.00818

68.3 -0.02784 -0.01036

68.4 -0.02692 -0.01172

68.5 -0.02346 -0.01202

68.6 -0.02168 -0.01076

68.7 -0.02129 -0.01092

68.8 -0.02042 -0.01084

68.9 -0.01783 -0.00909

69 -0.01695 -0.01036

69.1 -0.01621 -0.01128

69.2 -0.01875 -0.01212

69.3 -0.0213 -0.011

69.4 -0.02259 -0.01023

69.5 -0.02254 -0.00989

69.6 -0.02421 -0.01035

69.7 -0.02163 -0.01101

69.8 -0.0219 -0.01167

69.9 -0.01967 -0.01147

70 -0.0188 -0.01389

70.1 -0.01812 -0.01599

70.2 -0.01721 -0.01539

70.3 -0.0157 -0.01406

70.4 -0.01527 -0.01324

70.5 -0.01772 -0.01338

70.6 -0.01599 -0.01467

70.7 -0.01701 -0.01469

70.8 -0.0174 -0.01519

70.9 -0.01662 -0.01656

71 -0.01574 -0.01843

71.1 -0.01631 -0.01747

71.2 -0.01713 -0.01967

71.3 -0.01672 -0.01956

71.4 -0.01604 -0.02024

71.5 -0.01601 -0.02187

71.6 -0.01538 -0.02511

71.7 -0.01511 -0.02584

71.8 -0.01577 -0.02731

71.9 -0.01671 -0.0292

72 -0.0179 -0.02869

72.1 -0.01883 -0.02904

72.2 -0.01804 -0.03021

72.3 -0.01732 -0.031

72.4 -0.01966 -0.03201

72.5 -0.01942 -0.03134

72.6 -0.0175 -0.03042

72.7 -0.01751 -0.02859

72.8 -0.01531 -0.0284

72.9 -0.01123 -0.02954

73 -0.00973 -0.02999

73.1 -0.00998 -0.03103

73.2 -0.0129 -0.03071

73.3 -0.01385 -0.03055

73.4 -0.01214 -0.02941

73.5 -0.00962 -0.02861

73.6 -0.0095 -0.02749

73.7 -0.00954 -0.02696

73.8 -0.00995 -0.0275

73.9 -0.01273 -0.02763

74 -0.0125 -0.02713

74.1 -0.01215 -0.02786

74.2 -0.01077 -0.02712

74.3 -0.009 -0.02615

74.4 -0.0071 -0.0251

74.5 -0.00569 -0.02549

74.6 -0.00365 -0.02571

74.7 -0.00335 -0.02501

74.8 -0.00309 -0.02461

74.9 -0.00434 -0.02448

75 -0.00494 -0.02324

75.1 -0.00663 -0.02516

75.2 -0.00699 -0.02812

75.3 -0.00657 -0.02989

75.4 -0.00702 -0.02949

75.5 -0.00726 -0.03088

75.6 -0.00422 -0.03183

75.7 -0.00399 -0.03375

75.8 -0.00646 -0.03366

75.9 -0.00839 -0.03227

76 -0.00923 -0.03237

76.1 -0.00558 -0.03313

76.2 -0.00683 -0.036

76.3 -0.00657 -0.0379

76.4 -0.00628 -0.03764

76.5 -0.00676 -0.03929

76.6 -0.00949 -0.03941

76.7 -0.01074 -0.04065

76.8 -0.01232 -0.04164

76.9 -0.0132 -0.04244

77 -0.01562 -0.04319

77.1 -0.01634 -0.04168

77.2 -0.01558 -0.04241

77.3 -0.01232 -0.0422

77.4 -0.00916 -0.04463

77.5 -0.00719 -0.04438

77.6 -0.00725 -0.04452

77.7 -0.00853 -0.04602

77.8 -0.00916 -0.04691

77.9 -0.00753 -0.04712

78 -0.00614 -0.04816

78.1 -0.00589 -0.04907

78.2 -0.00443 -0.0502

78.3 -0.00584 -0.05196

78.4 -0.0058 -0.05331

78.5 -0.00525 -0.05359

78.6 -0.00169 -0.05193

78.7 2.07447E-4 -0.05149

78.8 -1.13084E-4 -0.04836

78.9 0.00227 -0.04876

79 0.00584 -0.04855

79.1 0.00626 -0.04766

79.2 0.00888 -0.04647

79.3 0.01083 -0.04784

79.4 0.01071 -0.04752

79.5 0.01196 -0.04886

79.6 0.01448 -0.04905

79.7 0.01739 -0.05141

79.8 0.0156 -0.05168

79.9 0.01571 -0.05372

80 0.01627 -0.05552

80.1 0.01499 -0.05615

80.2 0.01421 -0.05738

80.3 0.01452 -0.06008

80.4 0.0144 -0.06066

80.5 0.01152 -0.06117

80.6 0.01025 -0.06172

80.7 0.01083 -0.06248

80.8 0.00998 -0.06345

80.9 0.00829 -0.0649

81 0.00916 -0.06646

81.1 0.00733 -0.06716

81.2 0.00693 -0.07056

81.3 0.00727 -0.07196

81.4 0.00597 -0.07244

81.5 0.00452 -0.07188

81.6 0.00176 -0.07138

81.7 -0.00138 -0.07241

81.8 -0.00172 -0.07362

81.9 -0.00117 -0.07448

82 -7.74945E-4 -0.07441

82.1 0.00117 -0.07436

82.2 -1.5353E-4 -0.0747

82.3 -0.00323 -0.07456

82.4 -0.00562 -0.0752

82.5 -0.00618 -0.07567

82.6 -0.00741 -0.07482

82.7 -0.00796 -0.07421

82.8 -0.00924 -0.07529

82.9 -0.0083 -0.07561

83 -0.01032 -0.07678

83.1 -0.0104 -0.07706

83.2 -0.01008 -0.07945

83.3 -0.0088 -0.08162

83.4 -0.01191 -0.08345

83.5 -0.01482 -0.08305

83.6 -0.0161 -0.08463

83.7 -0.01762 -0.08381

83.8 -0.01508 -0.08228

83.9 -0.01595 -0.08473

84 -0.01524 -0.08331

84.1 -0.01719 -0.08278

84.2 -0.01639 -0.08221

84.3 -0.01349 -0.08024

84.4 -0.01319 -0.07872

84.5 -0.01172 -0.07735

84.6 -0.0082 -0.07456

84.7 -0.0105 -0.07309

84.8 -0.0097 -0.07175

84.9 -0.00769 -0.07307

85 -0.00779 -0.07328

85.1 -0.00566 -0.07271

85.2 -0.00584 -0.07256

85.3 -0.00418 -0.07245

85.4 -0.00197 -0.07174

85.5 -0.00125 -0.06997

85.6 -6.52919E-4 -0.07073

85.7 -8.12048E-4 -0.07127

85.8 -3.00034E-4 -0.07536

85.9 8.74004E-6 -0.07498

86 0.00172 -0.07621

86.1 0.00181 -0.07605

86.2 0.00264 -0.07518

86.3 0.00354 -0.07763

86.4 0.0063 -0.07755

86.5 0.00982 -0.07822

86.6 0.01341 -0.07749

86.7 0.01281 -0.07704

86.8 0.01281 -0.07613

86.9 0.01167 -0.07585

87 0.01385 -0.07757

87.1 0.01399 -0.07773

87.2 0.01494 -0.0781

87.3 0.01491 -0.07793

87.4 0.01609 -0.07775

87.5 0.01508 -0.07635

87.6 0.01343 -0.07901

87.7 0.01655 -0.07883

87.8 0.01485 -0.07893

87.9 0.01303 -0.08008

88 0.00993 -0.07902

88.1 0.00739 -0.07893

88.2 0.00513 -0.08017

88.3 0.00471 -0.08158

88.4 0.00368 -0.0822

88.5 0.00194 -0.08211

88.6 0.00387 -0.08324

88.7 0.00553 -0.08513

88.8 0.00617 -0.08561

88.9 0.00611 -0.08798

89 0.00541 -0.08861

89.1 0.00519 -0.09021

89.2 0.00484 -0.08814

89.3 0.00419 -0.08822

89.4 0.00474 -0.08944

89.5 0.00528 -0.09052

89.6 0.00624 -0.08968

89.7 0.00593 -0.08965

89.8 0.00696 -0.09166

89.9 0.00394 -0.09169

90 0.00199 -0.09204

90.1 8.33583E-4 -0.0892

90.2 0.00214 -0.08897

90.3 0.00156 -0.08715

90.4 6.34327E-4 -0.08365

90.5 -3.75908E-4 -0.08359

90.6 -7.66332E-5 -0.0855

90.7 -0.00241 -0.0862

90.8 -0.0038 -0.08735

90.9 -0.00224 -0.08791

91 1.23802E-4 -0.08602

91.1 3.79002E-4 -0.08695

91.2 0.00137 -0.08453

91.3 8.78376E-4 -0.08467

91.4 -0.00107 -0.08713

91.5 -0.00136 -0.0891

91.6 -0.0026 -0.08816

91.7 -0.00335 -0.0905

91.8 -0.00169 -0.09072

91.9 -0.00151 -0.09004

92 -0.00164 -0.08875

92.1 -0.0028 -0.0895

92.2 -0.00325 -0.09154

92.3 -0.00335 -0.09127

92.4 -0.00414 -0.09146

92.5 -0.00362 -0.09134

92.6 -0.00447 -0.09105

92.7 -0.00689 -0.09183

92.8 -0.00892 -0.09297

92.9 -0.01122 -0.09313

93 -0.01088 -0.09219

93.1 -0.01029 -0.09313

93.2 -0.01075 -0.09325

93.3 -0.01125 -0.09428

93.4 -0.01296 -0.09321

93.5 -0.01287 -0.09248

93.6 -0.01521 -0.09112

93.7 -0.01665 -0.09082

93.8 -0.01547 -0.09197

93.9 -0.01826 -0.08996

94 -0.01803 -0.08962

94.1 -0.01794 -0.08856

94.2 -0.01738 -0.08869

94.3 -0.0146 -0.08795

94.4 -0.01485 -0.08932

94.5 -0.01691 -0.0901

94.6 -0.01746 -0.09058

94.7 -0.01886 -0.09207

94.8 -0.02098 -0.0914

94.9 -0.02005 -0.09262

95 -0.02138 -0.0944

95.1 -0.0227 -0.09532

95.2 -0.02298 -0.09711

95.3 -0.02284 -0.09834

95.4 -0.02414 -0.09914

95.5 -0.02189 -0.09917

95.6 -0.01852 -0.09738

95.7 -0.01726 -0.09587

95.8 -0.01812 -0.09774

95.9 -0.01907 -0.09966

96 -0.01874 -0.09814

96.1 -0.01662 -0.09689

96.2 -0.01669 -0.09633

96.3 -0.01793 -0.09561

96.4 -0.01806 -0.09661

96.5 -0.01724 -0.09819

96.6 -0.01842 -0.09818

96.7 -0.01797 -0.09714

96.8 -0.02117 -0.09684

96.9 -0.02371 -0.0987

97 -0.02387 -0.09888

97.1 -0.02561 -0.10072

97.2 -0.02573 -0.10247

97.3 -0.02718 -0.10266

97.4 -0.02849 -0.10234

97.5 -0.03038 -0.10146

97.6 -0.0272 -0.1008

97.7 -0.02873 -0.10004

97.8 -0.02679 -0.09978

97.9 -0.02501 -0.09947

98 -0.02627 -0.09888

98.1 -0.02633 -0.09917

98.2 -0.02652 -0.0995

98.3 -0.02336 -0.10047

98.4 -0.02295 -0.09971

98.5 -0.02356 -0.10001

98.6 -0.02379 -0.10045

98.7 -0.02454 -0.10085

98.8 -0.02276 -0.10019

98.9 -0.0237 -0.09853

99 -0.02301 -0.09541

99.1 -0.02365 -0.09436

99.2 -0.02557 -0.09335

99.3 -0.02567 -0.09326

99.4 -0.02657 -0.09093

99.5 -0.02403 -0.09215

99.6 -0.02393 -0.09143

99.7 -0.02569 -0.09027

99.8 -0.02644 -0.08862

99.9 -0.02752 -0.08856

100 -0.02648 -0.08888

100.1 -0.02734 -0.09011

**Data for Figure 9**

The first column is time, the second column is cross correlation for residue 7-71, the third column is for cross correlation for residue 71-7

1 -0.16178 -0.1617

2 -0.13522 -0.1362

3 -0.13033 -0.1316

4 -0.12772 -0.1304

5 -0.12501 -0.1292

6 -0.12454 -0.128

7 -0.12208 -0.124

8 -0.12285 -0.1216

9 -0.12462 -0.1186

10 -0.12597 -0.1168

11 -0.12615 -0.1145

12 -0.12712 -0.1123

13 -0.12683 -0.1102

14 -0.12852 -0.1082

15 -0.13016 -0.1074

16 -0.13057 -0.1068

17 -0.12817 -0.105

18 -0.12641 -0.1025

19 -0.1271 -0.1023

20 -0.12803 -0.1012

21 -0.12679 -0.0998

22 -0.12706 -0.0987

23 -0.12581 -0.0979

24 -0.12445 -0.0965

25 -0.12362 -0.0981

26 -0.12417 -0.0979

27 -0.12332 -0.0992

28 -0.12408 -0.1005

29 -0.12379 -0.1003

30 -0.12441 -0.0997

31 -0.12386 -0.0998

32 -0.12383 -0.1013

33 -0.12529 -0.1021

34 -0.12542 -0.1025

35 -0.12282 -0.1004

36 -0.12226 -0.0991

37 -0.12194 -0.0991

38 -0.12081 -0.0985

39 -0.12133 -0.0964

40 -0.12229 -0.0956

41 -0.12215 -0.0957

42 -0.12471 -0.0946

43 -0.12444 -0.0943

44 -0.12392 -0.0947

45 -0.12329 -0.0968

46 -0.12235 -0.0979

47 -0.12046 -0.0985

48 -0.12003 -0.099

49 -0.1204 -0.0995

50 -0.12073 -0.0964

51 -0.12082 -0.0951

Data for Fig 10

First column is time in nanoseconds, second column for cross correlation of 14-53, third column is for cross correlation of 53-14

0 -0.16858 -0.16858

0.1 -0.15329 -0.14972

0.2 -0.14594 -0.1429

0.3 -0.14288 -0.13784

0.4 -0.14034 -0.13345

0.5 -0.13969 -0.12909

0.6 -0.13818 -0.12542

0.7 -0.13673 -0.12346

0.8 -0.13584 -0.1213

0.9 -0.13496 -0.11963

1 -0.13403 -0.11647

1.1 -0.13383 -0.11508A

1.2 -0.13348 -0.11253

1.3 -0.1325 -0.11095

1.4 -0.13435 -0.10879

1.5 -0.13356 -0.10689

1.6 -0.13183 -0.10425

1.7 -0.13252 -0.10229

1.8 -0.13222 -0.10175

1.9 -0.13152 -0.09953

2 -0.12963 -0.09747

2.1 -0.12845 -0.09618

2.2 -0.12739 -0.09465

2.3 -0.1277 -0.09362

2.4 -0.12788 -0.09313

2.5 -0.12757 -0.09303

2.6 -0.12778 -0.09244

2.7 -0.12684 -0.09298

2.8 -0.12556 -0.09222

2.9 -0.12484 -0.09103

3 -0.12581 -0.09039

3.1 -0.12561 -0.09094

3.2 -0.12653 -0.09085

3.3 -0.12683 -0.08887

3.4 -0.12636 -0.08715

3.5 -0.12501 -0.08562

3.6 -0.12403 -0.08478

3.7 -0.12475 -0.08479

3.8 -0.12162 -0.08297

3.9 -0.11895 -0.081

4 -0.11706 -0.08191

4.1 -0.11649 -0.08234

4.2 -0.11522 -0.08155

4.3 -0.11356 -0.08083

4.4 -0.11245 -0.08028

4.5 -0.1122 -0.0796

4.6 -0.11284 -0.07903

4.7 -0.11382 -0.07831

4.8 -0.11276 -0.07601

4.9 -0.11327 -0.07607

5 -0.1122 -0.07644

5.1 -0.11244 -0.07612

5.2 -0.11131 -0.07643

5.3 -0.10898 -0.07511

5.4 -0.10709 -0.07355

5.5 -0.10552 -0.07337

5.6 -0.10441 -0.07314

5.7 -0.10457 -0.07178

5.8 -0.10256 -0.07161

5.9 -0.10191 -0.07011

6 -0.10026 -0.07008

6.1 -0.09937 -0.06955

6.2 -0.09764 -0.06915

6.3 -0.09787 -0.06955

6.4 -0.09761 -0.07018

6.5 -0.09679 -0.07098

6.6 -0.09785 -0.06891

6.7 -0.0987 -0.06891

6.8 -0.10034 -0.06844

6.9 -0.10099 -0.06781

7 -0.10102 -0.06712

7.1 -0.10019 -0.06727

7.2 -0.10016 -0.06932

7.3 -0.10039 -0.06973

7.4 -0.10058 -0.06816

7.5 -0.10049 -0.06723

7.6 -0.10095 -0.06704

7.7 -0.10041 -0.06783

7.8 -0.0995 -0.0673

7.9 -0.09901 -0.06762

8 -0.09877 -0.0673

8.1 -0.09738 -0.06672

8.2 -0.09662 -0.06639

8.3 -0.0952 -0.06715

8.4 -0.09361 -0.06795

8.5 -0.09065 -0.06633

8.6 -0.08909 -0.06593

8.7 -0.08764 -0.06504

8.8 -0.08523 -0.06644

8.9 -0.08628 -0.06664

9 -0.08607 -0.06606

9.1 -0.08498 -0.0657

9.2 -0.08357 -0.06419

9.3 -0.08311 -0.06415

9.4 -0.08282 -0.06332

9.5 -0.08168 -0.06307

9.6 -0.07995 -0.06232

9.7 -0.08009 -0.06356

9.8 -0.08058 -0.06395

9.9 -0.08104 -0.06408

10 -0.08069 -0.06344

10.1 -0.07981 -0.06538

10.2 -0.07974 -0.06705

10.3 -0.07828 -0.06728

10.4 -0.07974 -0.06731

10.5 -0.07864 -0.06672

10.6 -0.07798 -0.06639

10.7 -0.07798 -0.06659

10.8 -0.07799 -0.06649

10.9 -0.0779 -0.06627

11 -0.07797 -0.0641

11.1 -0.07737 -0.06211

11.2 -0.07912 -0.06089

11.3 -0.07978 -0.06149

11.4 -0.0794 -0.06202

11.5 -0.07865 -0.06204

11.6 -0.07784 -0.06299

11.7 -0.07865 -0.06213

11.8 -0.07901 -0.06131

11.9 -0.07825 -0.06217

12 -0.07767 -0.061

12.1 -0.07805 -0.06135

12.2 -0.07863 -0.06078

12.3 -0.0794 -0.06068

12.4 -0.07817 -0.06086

12.5 -0.07803 -0.06056

12.6 -0.0781 -0.06017

12.7 -0.07827 -0.05979

12.8 -0.07861 -0.05905

12.9 -0.07784 -0.0588

13 -0.07705 -0.05754

13.1 -0.07721 -0.0573

13.2 -0.07743 -0.05661

13.3 -0.07664 -0.05751

13.4 -0.07652 -0.05761

13.5 -0.07479 -0.0586

13.6 -0.07432 -0.05923

13.7 -0.07387 -0.0596

13.8 -0.07479 -0.05978

13.9 -0.07572 -0.06114

14 -0.07505 -0.06068

14.1 -0.07444 -0.06003

14.2 -0.07263 -0.05959

14.3 -0.07147 -0.05963

14.4 -0.07117 -0.05919

14.5 -0.07067 -0.06004

14.6 -0.07169 -0.06039

14.7 -0.071 -0.05929

14.8 -0.0706 -0.05905

14.9 -0.06747 -0.0587

15 -0.06889 -0.05778

15.1 -0.06866 -0.05954

15.2 -0.06754 -0.05835

15.3 -0.06799 -0.05824

15.4 -0.06877 -0.05796

15.5 -0.07018 -0.05802

15.6 -0.0711 -0.0567

15.7 -0.07124 -0.05626

15.8 -0.07133 -0.05506

15.9 -0.07012 -0.05588

16 -0.07019 -0.05479

16.1 -0.07008 -0.05528

16.2 -0.06825 -0.05479

16.3 -0.06815 -0.05546

16.4 -0.06596 -0.05377

16.5 -0.06459 -0.05252

16.6 -0.06572 -0.05265

16.7 -0.06658 -0.0517

16.8 -0.06549 -0.0515

16.9 -0.06527 -0.05116

17 -0.06448 -0.04956

17.1 -0.06267 -0.04867

17.2 -0.05981 -0.04864

17.3 -0.05936 -0.04874

17.4 -0.05879 -0.04792

17.5 -0.05835 -0.04749

17.6 -0.05694 -0.04624

17.7 -0.05583 -0.04589

17.8 -0.05541 -0.04658

17.9 -0.05632 -0.04604

18 -0.05461 -0.04538

18.1 -0.0538 -0.04535

18.2 -0.05401 -0.0451

18.3 -0.05459 -0.04599

18.4 -0.05341 -0.04636

18.5 -0.0534 -0.04507

18.6 -0.0529 -0.0452

18.7 -0.05339 -0.04524

18.8 -0.05436 -0.04525

18.9 -0.05415 -0.04634

19 -0.05449 -0.04588

19.1 -0.05399 -0.04455

19.2 -0.05362 -0.04494

19.3 -0.05483 -0.04547

19.4 -0.05532 -0.04543

19.5 -0.05551 -0.04433

19.6 -0.05549 -0.04277

19.7 -0.05621 -0.04151

19.8 -0.05732 -0.04094

19.9 -0.05697 -0.0404

20 -0.05766 -0.03911

20.1 -0.05805 -0.03904

20.2 -0.05759 -0.03869

20.3 -0.05813 -0.03901

20.4 -0.05887 -0.03871

20.5 -0.05887 -0.03771

20.6 -0.05854 -0.03594

20.7 -0.05843 -0.03423

20.8 -0.05883 -0.03624

20.9 -0.05881 -0.03613

21 -0.05887 -0.03614

21.1 -0.05927 -0.03647

21.2 -0.05812 -0.03583

21.3 -0.05807 -0.03508

21.4 -0.05776 -0.03543

21.5 -0.05778 -0.03609

21.6 -0.05698 -0.03607

21.7 -0.05733 -0.03723

21.8 -0.0564 -0.03851

21.9 -0.05634 -0.03786

22 -0.05602 -0.03792

22.1 -0.0553 -0.03705

22.2 -0.05436 -0.03808

22.3 -0.05265 -0.03776

22.4 -0.0531 -0.03721

22.5 -0.0531 -0.03888

22.6 -0.05197 -0.03989

22.7 -0.05109 -0.0401

22.8 -0.05075 -0.04118

22.9 -0.04986 -0.0418

23 -0.05019 -0.04138

23.1 -0.04892 -0.04078

23.2 -0.04887 -0.04106

23.3 -0.04985 -0.04194

23.4 -0.05039 -0.04264

23.5 -0.05085 -0.04337

23.6 -0.05132 -0.04292

23.7 -0.04997 -0.04269

23.8 -0.04994 -0.04202

23.9 -0.05035 -0.0419

24 -0.05085 -0.04213

24.1 -0.05264 -0.04338

24.2 -0.05142 -0.04314

24.3 -0.05108 -0.0432

24.4 -0.05224 -0.04288

24.5 -0.05306 -0.04085

24.6 -0.05371 -0.04053

24.7 -0.054 -0.03821

24.8 -0.05356 -0.03711

24.9 -0.05385 -0.0354

25 -0.05419 -0.03382

25.1 -0.05182 -0.03258

25.2 -0.05232 -0.0336

25.3 -0.05252 -0.03402

25.4 -0.05256 -0.03375

25.5 -0.05278 -0.03327

25.6 -0.0518 -0.0339

25.7 -0.05085 -0.03356

25.8 -0.05014 -0.0337

25.9 -0.04975 -0.03341

26 -0.04806 -0.03295

26.1 -0.04786 -0.0327

26.2 -0.04707 -0.03312

26.3 -0.04626 -0.0324

26.4 -0.04636 -0.03112

26.5 -0.0452 -0.03063

26.6 -0.04566 -0.03066

26.7 -0.04694 -0.02995

26.8 -0.04812 -0.03062

26.9 -0.0482 -0.02874

27 -0.048 -0.02782

27.1 -0.04845 -0.02727

27.2 -0.04667 -0.02697

27.3 -0.0475 -0.0274

27.4 -0.04653 -0.02673

27.5 -0.04621 -0.02495

27.6 -0.04781 -0.02412

27.7 -0.04762 -0.02463

27.8 -0.04744 -0.02349

27.9 -0.04788 -0.02388

28 -0.04891 -0.02329

28.1 -0.04699 -0.02298

28.2 -0.04727 -0.02282

28.3 -0.04735 -0.02247

28.4 -0.04677 -0.02116

28.5 -0.04712 -0.02124

28.6 -0.04921 -0.02122

28.7 -0.04926 -0.02002

28.8 -0.05025 -0.01973

28.9 -0.05057 -0.01803

29 -0.05133 -0.01676

29.1 -0.05134 -0.01599

29.2 -0.05207 -0.01584

29.3 -0.0535 -0.0154

29.4 -0.05285 -0.01626

29.5 -0.05222 -0.01512

29.6 -0.05435 -0.01354

29.7 -0.05546 -0.01379

29.8 -0.05463 -0.01181

29.9 -0.05487 -0.01246

30 -0.05343 -0.01161

30.1 -0.05106 -0.01158

30.2 -0.05158 -0.01303

30.3 -0.05136 -0.01235

30.4 -0.05259 -0.01036

30.5 -0.0514 -0.00914

30.6 -0.05183 -0.00971

30.7 -0.05165 -0.00858

30.8 -0.05142 -0.00766

30.9 -0.05089 -0.00681

31 -0.05186 -0.00536

31.1 -0.05172 -0.00456

31.2 -0.05177 -0.00391

31.3 -0.05244 -0.00361

31.4 -0.05366 -0.00494

31.5 -0.05479 -0.00506

31.6 -0.05323 -0.00448

31.7 -0.0534 -0.0046

31.8 -0.05345 -0.00468

31.9 -0.05305 -0.00596

32 -0.05397 -0.00513

32.1 -0.05256 -0.00546

32.2 -0.0546 -0.00525

32.3 -0.05528 -0.00469

32.4 -0.0553 -0.00434

32.5 -0.05698 -0.00423

32.6 -0.05634 -0.00316

32.7 -0.05732 -0.00352

32.8 -0.05593 -0.00205

32.9 -0.05566 -0.00247

33 -0.05495 -0.00178

33.1 -0.05251 -0.0011

33.2 -0.05262 -9.19611E-4

33.3 -0.05264 -0.00127

33.4 -0.05305 -0.00236

33.5 -0.05272 -0.00197

33.6 -0.05294 -0.00127

33.7 -0.0535 -0.00159

33.8 -0.05243 -0.00108

33.9 -0.05276 -0.00179

34 -0.05319 -8.75896E-4

34.1 -0.05245 -3.6954E-4

34.2 -0.052 -3.57798E-4

34.3 -0.05046 7.79295E-4

34.4 -0.05064 -5.03387E-4

34.5 -0.05034 9.37325E-5

34.6 -0.04988 0.00176

34.7 -0.04951 8.63495E-4

34.8 -0.04984 0.0015

34.9 -0.04976 0.00218

35 -0.04875 0.00269

35.1 -0.04858 0.00144

35.2 -0.04712 0.00106

35.3 -0.04742 0.00153

35.4 -0.04609 0.00166

35.5 -0.04577 0.0027

35.6 -0.04504 0.00164

35.7 -0.04518 3.28711E-4

35.8 -0.04516 1.11892E-5

35.9 -0.0468 8.07717E-4

36 -0.04613 9.51197E-4

36.1 -0.04561 0.00229

36.2 -0.0443 8.93296E-4

36.3 -0.0431 0.00251

36.4 -0.04271 0.004

36.5 -0.04266 0.00412

36.6 -0.04261 0.00388

36.7 -0.04426 0.00346

36.8 -0.04429 0.00408

36.9 -0.04528 0.00403

37 -0.04532 0.00413

37.1 -0.04431 0.00519

37.2 -0.04395 0.00432

37.3 -0.04442 0.00359

37.4 -0.04526 0.00263

37.5 -0.04541 0.00349

37.6 -0.04563 0.00117

37.7 -0.04606 0.00266

37.8 -0.04582 0.00406

37.9 -0.04566 0.00623

38 -0.04517 0.00612

38.1 -0.04533 0.00779

38.2 -0.04567 0.00968

38.3 -0.04599 0.01085

38.4 -0.04653 0.01175

38.5 -0.04733 0.01296

38.6 -0.04699 0.01289

38.7 -0.04785 0.01158

38.8 -0.04756 0.01219

38.9 -0.04692 0.01282

39 -0.04621 0.01294

39.1 -0.04512 0.0125

39.2 -0.04621 0.01253

39.3 -0.04424 0.01413

39.4 -0.0435 0.01446

39.5 -0.04324 0.01532

39.6 -0.04295 0.01446

39.7 -0.04175 0.01411

39.8 -0.04199 0.01358

39.9 -0.04299 0.01347

40 -0.04192 0.01236

40.1 -0.04025 0.01161

40.2 -0.03989 0.01071

40.3 -0.04093 0.01072

40.4 -0.041 0.01147

40.5 -0.03935 0.01369

40.6 -0.03882 0.0151

40.7 -0.03831 0.01499

40.8 -0.03871 0.01576

40.9 -0.03702 0.0145

41 -0.0362 0.01563

41.1 -0.0354 0.01647

41.2 -0.03568 0.01719

41.3 -0.03564 0.01891

41.4 -0.03593 0.0204

41.5 -0.03537 0.02008

41.6 -0.03322 0.02216

41.7 -0.03372 0.02341

41.8 -0.03263 0.02412

41.9 -0.03297 0.02481

42 -0.03207 0.02455

42.1 -0.03165 0.02496

42.2 -0.02992 0.02476

42.3 -0.02839 0.02649

42.4 -0.02819 0.02635

42.5 -0.02721 0.02791

42.6 -0.02652 0.0293

42.7 -0.0264 0.03019

42.8 -0.02569 0.03072

42.9 -0.025 0.03238

43 -0.02333 0.03286

43.1 -0.02276 0.03129

43.2 -0.02248 0.03204

43.3 -0.02312 0.03225

43.4 -0.02211 0.03191

43.5 -0.02146 0.03296

43.6 -0.02088 0.03409

43.7 -0.02052 0.036

43.8 -0.02049 0.03646

43.9 -0.02048 0.03506

44 -0.02113 0.03408

44.1 -0.02039 0.03359

44.2 -0.02058 0.03384

44.3 -0.02036 0.03326

44.4 -0.01969 0.03292

44.5 -0.01945 0.03084

44.6 -0.01863 0.03191

44.7 -0.01839 0.03164

44.8 -0.019 0.0304

44.9 -0.01958 0.02943

45 -0.01843 0.02897

45.1 -0.0177 0.03052

45.2 -0.01724 0.02957

45.3 -0.01619 0.02949

45.4 -0.01452 0.02935

45.5 -0.01352 0.02934

45.6 -0.01327 0.02864

45.7 -0.01192 0.02867

45.8 -0.01177 0.02857

45.9 -0.01143 0.02725

46 -0.0121 0.02531

46.1 -0.01232 0.02454

46.2 -0.01065 0.0261

46.3 -0.01029 0.02666

46.4 -0.01047 0.02727

46.5 -0.01047 0.02673

46.6 -0.01024 0.02764

46.7 -0.00999 0.0276

46.8 -0.0097 0.02758

46.9 -0.00987 0.02745

47 -0.00947 0.02864

47.1 -0.00883 0.02795

47.2 -0.00887 0.02825

47.3 -0.00767 0.0278

47.4 -0.00828 0.02772

47.5 -0.00791 0.02826

47.6 -0.00787 0.02744

47.7 -0.00764 0.02847

47.8 -0.01004 0.02771

47.9 -0.00943 0.02796

48 -0.00927 0.02605

48.1 -0.00948 0.02442

48.2 -0.01019 0.02485

48.3 -0.00991 0.02283

48.4 -0.01028 0.0219

48.5 -0.01065 0.02189

48.6 -0.01023 0.02342

48.7 -0.01004 0.02257

48.8 -0.00982 0.02192

48.9 -0.0098 0.0212

49 -0.00898 0.02204

49.1 -0.01039 0.02229

49.2 -0.00761 0.02244

49.3 -0.00772 0.02226

49.4 -0.00818 0.02169

49.5 -0.00654 0.02003

49.6 -0.00871 0.0183

49.7 -0.00884 0.01732

49.8 -0.00826 0.01546

49.9 -0.00795 0.01386

50 -0.00687 0.01407

50.1 -0.00698 0.01557

50.2 -0.00593 0.01756

50.3 -0.00727 0.01682

50.4 -0.00611 0.01673

50.5 -0.0067 0.01544

50.6 -0.00586 0.01712

50.7 -0.00465 0.01885

50.8 -0.00395 0.02041

50.9 -0.00388 0.0221

51 -0.00362 0.02182

51.1 -0.00284 0.02264

51.2 -0.00247 0.02313

51.3 -0.00262 0.02251

51.4 -0.00494 0.0229

51.5 -0.00454 0.02316

51.6 -0.00243 0.02394

51.7 -0.00186 0.02423

51.8 -0.0013 0.02474

51.9 -3.96006E-4 0.02479

52 0.00145 0.02672

52.1 6.9765E-4 0.02847

52.2 8.18171E-4 0.02792

52.3 6.37093E-4 0.02626

52.4 -2.47281E-4 0.0281

52.5 -1.5908E-4 0.0303

52.6 -0.00123 0.03072

52.7 2.05998E-4 0.03204

52.8 3.64826E-4 0.0335

52.9 4.31966E-4 0.03448

53 6.15566E-4 0.03608

53.1 0.00102 0.0357

53.2 9.19363E-4 0.03567

53.3 3.8277E-4 0.03474

53.4 0.0017 0.03496

53.5 0.00357 0.03552

53.6 0.00503 0.03573

53.7 0.00604 0.03525

53.8 0.00539 0.03476

53.9 0.00676 0.03537

54 0.00659 0.03583

54.1 0.00772 0.03728

54.2 0.00681 0.0376

54.3 0.00791 0.03847

54.4 0.0079 0.03912

54.5 0.00867 0.03881

54.6 0.00867 0.03872

54.7 0.00833 0.03852

54.8 0.00858 0.03898

54.9 0.00895 0.03985

55 0.00879 0.04001

55.1 0.00761 0.0402

55.2 0.00764 0.03957

55.3 0.00737 0.04038

55.4 0.00763 0.0425

55.5 0.00732 0.04146

55.6 0.00661 0.04124

55.7 0.00702 0.04156

55.8 0.00778 0.04335

55.9 0.00654 0.04445

56 0.00627 0.04464

56.1 0.00622 0.044

56.2 0.0064 0.04392

56.3 0.00461 0.04406

56.4 0.00582 0.04364

56.5 0.00654 0.04466

56.6 0.00702 0.04477

56.7 0.00821 0.04456

56.8 0.00803 0.04477

56.9 0.00917 0.04343

57 0.00857 0.04341

57.1 0.00864 0.04301

57.2 0.00831 0.04407

57.3 0.00842 0.04465

57.4 0.00771 0.04414

57.5 0.00787 0.04402

57.6 0.00784 0.0436

57.7 0.00742 0.04217

57.8 0.00859 0.04208

57.9 0.00904 0.04263

58 0.00849 0.04247

58.1 0.0096 0.04395

58.2 0.00976 0.04505

58.3 0.01043 0.0467

58.4 0.01184 0.04838

58.5 0.01169 0.04926

58.6 0.01292 0.05019

58.7 0.01395 0.05145

58.8 0.01413 0.05198

58.9 0.01407 0.05246

59 0.01381 0.05352

59.1 0.01434 0.05506

59.2 0.01311 0.05648

59.3 0.01476 0.05716

59.4 0.01518 0.05714

59.5 0.01485 0.0577

59.6 0.0153 0.05811

59.7 0.01528 0.05802

59.8 0.01587 0.05721

59.9 0.01538 0.05674

60 0.01654 0.05685

60.1 0.01441 0.05633

60.2 0.01231 0.05518

60.3 0.01064 0.05447

60.4 0.01134 0.05471

60.5 0.01098 0.05533

60.6 0.01153 0.05532

60.7 0.0105 0.05568

60.8 0.01189 0.05583

60.9 0.01247 0.05673

61 0.01339 0.05782

61.1 0.01468 0.05867

61.2 0.01415 0.05788

61.3 0.01618 0.05853

61.4 0.01586 0.05723

61.5 0.0161 0.05637

61.6 0.01701 0.05454

61.7 0.016 0.05428

61.8 0.01644 0.05502

61.9 0.01913 0.0542

62 0.01998 0.05532

62.1 0.01939 0.05496

62.2 0.02123 0.05643

62.3 0.02054 0.05683

62.4 0.02006 0.05694

62.5 0.02085 0.05862

62.6 0.02029 0.0577

62.7 0.02074 0.05766

62.8 0.02117 0.0559

62.9 0.02101 0.05651

63 0.02026 0.05592

63.1 0.02067 0.05546

63.2 0.02056 0.05418

63.3 0.0201 0.05428

63.4 0.022 0.05319

63.5 0.02187 0.05219

63.6 0.0204 0.05156

63.7 0.02133 0.05246

63.8 0.02375 0.05259

63.9 0.02374 0.05213

64 0.02482 0.05303

64.1 0.02538 0.05366

64.2 0.02695 0.05364

64.3 0.02774 0.0537

64.4 0.02616 0.05338

64.5 0.02685 0.0519

64.6 0.0279 0.05131

64.7 0.02997 0.05079

64.8 0.03025 0.04957

64.9 0.03046 0.04852

65 0.03077 0.0466

65.1 0.03179 0.04593

65.2 0.03313 0.04428

65.3 0.03373 0.04277

65.4 0.03443 0.0408

65.5 0.03445 0.03991

65.6 0.03547 0.03951

65.7 0.03622 0.03859

65.8 0.03604 0.03772

65.9 0.03594 0.03605

66 0.03638 0.0348

66.1 0.03586 0.03389

66.2 0.03616 0.03312

66.3 0.03668 0.03293

66.4 0.03587 0.03144

66.5 0.03614 0.03117

66.6 0.03618 0.03011

66.7 0.0353 0.02943

66.8 0.03478 0.02813

66.9 0.03575 0.0282

67 0.03671 0.02874

67.1 0.03765 0.02983

67.2 0.03734 0.03098

67.3 0.03682 0.03005

67.4 0.03665 0.02982

67.5 0.03626 0.0306

67.6 0.03789 0.0319

67.7 0.03727 0.0309

67.8 0.03631 0.0295

67.9 0.03547 0.02831

68 0.0353 0.02793

68.1 0.03541 0.02826

68.2 0.03488 0.02787

68.3 0.0337 0.0267

68.4 0.03317 0.02549

68.5 0.03228 0.02601

68.6 0.03396 0.02557

68.7 0.03302 0.02278

68.8 0.03329 0.02369

68.9 0.03315 0.0243

69 0.03342 0.02504

69.1 0.03391 0.02518

69.2 0.03454 0.02551

69.3 0.03472 0.02502

69.4 0.03546 0.02416

69.5 0.0371 0.02363

69.6 0.03673 0.02246

69.7 0.03655 0.02211

69.8 0.03527 0.02082

69.9 0.03423 0.02156

70 0.0331 0.02199

70.1 0.0322 0.02135

70.2 0.03153 0.02252

70.3 0.03063 0.02256

70.4 0.03189 0.02513

70.5 0.0326 0.02684

70.6 0.03182 0.02752

70.7 0.03161 0.02859

70.8 0.03224 0.02805

70.9 0.03172 0.02585

71 0.03236 0.02474

71.1 0.0329 0.02438

71.2 0.03294 0.02377

71.3 0.03156 0.02443

71.4 0.03084 0.02398

71.5 0.03084 0.02626

71.6 0.03155 0.02441

71.7 0.03166 0.02312

71.8 0.03211 0.02424

71.9 0.03084 0.02428

72 0.03029 0.02452

72.1 0.03141 0.02296

72.2 0.03325 0.02352

72.3 0.03516 0.02329

72.4 0.03464 0.0242

72.5 0.03442 0.02333

72.6 0.03273 0.02245

72.7 0.03248 0.02222

72.8 0.03229 0.02188

72.9 0.03295 0.02065

73 0.03298 0.02032

73.1 0.03252 0.02204

73.2 0.03379 0.02155

73.3 0.0337 0.02067

73.4 0.03368 0.0189

73.5 0.0338 0.01953

73.6 0.03284 0.01903

73.7 0.03176 0.01897

73.8 0.03284 0.01851

73.9 0.03231 0.01733

74 0.03202 0.01862

74.1 0.03358 0.01809

74.2 0.03383 0.0182

74.3 0.03439 0.01795

74.4 0.03394 0.01847

74.5 0.03199 0.01854

74.6 0.03389 0.0186

74.7 0.03444 0.02006

74.8 0.03552 0.02131

74.9 0.03664 0.02292

75 0.03749 0.02281

75.1 0.03856 0.02508

75.2 0.03939 0.02525

75.3 0.03964 0.02554

75.4 0.04038 0.02524

75.5 0.03998 0.02496

75.6 0.04076 0.02592

75.7 0.03927 0.02689

75.8 0.03873 0.02783

75.9 0.0378 0.02696

76 0.03847 0.02556

76.1 0.03822 0.02435

76.2 0.03865 0.0235

76.3 0.03944 0.02316

76.4 0.03884 0.02243

76.5 0.03995 0.02183

76.6 0.04059 0.0211

76.7 0.0391 0.02113

76.8 0.03875 0.02106

76.9 0.03943 0.02135

77 0.04023 0.02143

77.1 0.03922 0.02067

77.2 0.03836 0.01943

77.3 0.03663 0.01752

77.4 0.03679 0.01705

77.5 0.03792 0.01792

77.6 0.03868 0.01899

77.7 0.03902 0.01789

77.8 0.03879 0.01748

77.9 0.04032 0.01798

78 0.03923 0.01915

78.1 0.03977 0.01782

78.2 0.03921 0.01781

78.3 0.03821 0.01587

78.4 0.03969 0.01536

78.5 0.03956 0.01259

78.6 0.03927 0.01117

78.7 0.0383 0.00993

78.8 0.04034 0.00969

78.9 0.04012 0.00791

79 0.04059 0.00792

79.1 0.04017 0.0077

79.2 0.03859 0.00756

79.3 0.03942 0.00726

79.4 0.04032 0.00839

79.5 0.04003 0.00767

79.6 0.038 0.00671

79.7 0.03835 0.00756

79.8 0.03792 0.00727

79.9 0.03793 0.00784

80 0.03759 0.00868

80.1 0.0345 0.00832

80.2 0.03404 0.00832

80.3 0.03189 0.00742

80.4 0.03187 0.00721

80.5 0.03101 0.00623

80.6 0.03014 0.00786

80.7 0.02944 0.00762

80.8 0.02776 0.00799

80.9 0.02773 0.00859

81 0.02702 0.00887

81.1 0.02636 0.00899

81.2 0.0259 0.00814

81.3 0.0249 0.00932

81.4 0.02493 0.01067

81.5 0.02346 0.01293

81.6 0.02377 0.01186

81.7 0.02291 0.01134

81.8 0.0217 0.00906

81.9 0.02219 0.00714

82 0.0238 0.00676

82.1 0.02455 0.00694

82.2 0.02511 0.00821

82.3 0.02689 0.00829

82.4 0.02649 0.00877

82.5 0.02901 0.00911

82.6 0.02925 0.00956

82.7 0.02907 0.01179

82.8 0.02902 0.01224

82.9 0.03075 0.0113

83 0.03132 0.01028

83.1 0.03242 0.01137

83.2 0.03359 0.01204

83.3 0.03287 0.01278

83.4 0.03445 0.01261

83.5 0.0357 0.01176

83.6 0.03582 0.0106

83.7 0.0357 0.01023

83.8 0.03646 0.01008

83.9 0.03623 0.0097

84 0.03793 0.01

84.1 0.0373 0.00907

84.2 0.03638 0.00911

84.3 0.03559 0.00826

84.4 0.03336 0.00852

84.5 0.03248 0.00805

84.6 0.03241 0.00907

84.7 0.03165 0.00891

84.8 0.02973 0.00878

84.9 0.03019 0.00761

85 0.03115 0.00748

85.1 0.03149 0.00815

85.2 0.03006 0.00971

85.3 0.02876 0.01097

85.4 0.02775 0.01152

85.5 0.02692 0.01146

85.6 0.02459 0.01293

85.7 0.02448 0.01341

85.8 0.02426 0.01401

85.9 0.02264 0.01463

86 0.02148 0.01413

86.1 0.02248 0.01434

86.2 0.02329 0.0147

86.3 0.02341 0.01265

86.4 0.02297 0.01195

86.5 0.0233 0.01184

86.6 0.02288 0.01164

86.7 0.02122 0.01198

86.8 0.02073 0.01101

86.9 0.01988 0.01081

87 0.018 0.01068

87.1 0.0174 0.01092
[truncated: 6,145 more chars]
